# Important Medicinal and Food Taxa (Orders and Families) in Kenya, Based on Three Quantitative Approaches

**DOI:** 10.3390/plants12051145

**Published:** 2023-03-02

**Authors:** Fredrick Munyao Mutie, Yuvenalis Morara Mbuni, Peninah Cheptoo Rono, Elijah Mbandi Mkala, John Mulinge Nzei, Methee Phumthum, Guang-Wan Hu, Qing-Feng Wang

**Affiliations:** 1 CAS Key Laboratory of Plant Germplasm and Specialty Agriculture, Wuhan Botanical Garden, Chinese Academy of Sciences, Wuhan 430074, China; 2 Sino-Africa Joint Research Center, Chinese Academy of Sciences, Wuhan 430074, China; 3 University of Chinese Academy of Sciences, Beijing 100049, China; 4 East African Herbarium, Nairobi National Museums, P.O. Box 45166, Nairobi 00100, Kenya; 5 Department of Pharmaceutical Botany, Faculty of Pharmacy, Mahidol University, Bangkok 10400, Thailand

**Keywords:** Bayesian analysis, binomial analysis, linear regression, outlier

## Abstract

Globally, food and medicinal plants have been documented, but their use patterns are poorly understood. Useful plants are non-random subsets of flora, prioritizing certain taxa. This study evaluates orders and families prioritized for medicine and food in Kenya, using three statistical models: Regression, Binomial, and Bayesian approaches. An extensive literature search was conducted to gather information on indigenous flora, medicinal and food plants. Regression residuals, obtained using LlNEST linear regression function, were used to quantify if taxa had unexpectedly high number of useful species relative to the overall proportion in the flora. Bayesian analysis, performed using BETA.INV function, was used to obtain superior and inferior 95% probability credible intervals for the whole flora and for all taxa. To test for the significance of individual taxa departure from the expected number, binomial analysis using BINOMDIST function was performed to obtain *p*-values for all taxa. The three models identified 14 positive outlier medicinal orders, all with significant values (*p* < 0.05). Fabales had the highest (66.16) regression residuals, while Sapindales had the highest (1.1605) R-value. Thirty-eight positive outlier medicinal families were identified; 34 were significant outliers (*p* < 0.05). Rutaceae (1.6808) had the highest R-value, while Fabaceae had the highest regression residuals (63.2). Sixteen positive outlier food orders were recovered; 13 were significant outliers (*p* < 0.05). Gentianales (45.27) had the highest regression residuals, while Sapindales (2.3654) had the highest R-value. Forty-two positive outlier food families were recovered by the three models; 30 were significant outliers (*p* < 0.05). Anacardiaceae (5.163) had the highest R-value, while Fabaceae had the highest (28.72) regression residuals. This study presents important medicinal and food taxa in Kenya, and adds useful data for global comparisons.

## 1. Introduction

Scientific literature documents that traditional herbs are as old as mankind and are utilized three to four times compared to modern medicines [1]. Globally, between 35,000 and 70,000 plant species, including monocots, dicots, gymnosperms, pteridophytes, and lichens, have been used for medicine in different cultures at some point in time [2]. In Africa, over 5400 species are medicinal, and about 10% have been, to some extent, developed commercially [3]. Many modern medicine prescriptions are plant-based [1], with estimates indicating that 67% of chemotherapy drugs are derived from natural products [4], whereas higher plants contribute over 25% of all drugs in clinical use [5]. Over 50,000 secondary metabolites have been discovered in the plant kingdom alone [6]. The potential for discovering more compounds is higher in the tropics since about 1% of tropical plants have been studied for pharmaceutical potential, although the tropics harbor nearly half of all flowering plants [5]. If medicinal plants were not efficacious, they would not have developed into a primary means of treatment in many societies [7]. This argument was justified when a test involving medicinal and non-medicinal plants was carried out against the measles virus: non-medicinal plants failed to decrease replication of the virus below control levels in infected U937 cells after 48–72 h, while extracts from medicinal plants had a neutralization effect [8]. Successful treatments using plant-based medicines include the use of artemisinin as an antimalarial drug [9] and the application of neem shampoo to eradicate head lice [10].

Food plants are also important resources, and globally, over 77% of rural households collect them, and this proportion is higher in Africa (88%). Wild food plants are harvested for both commercial and subsistence purposes [11]. Generally, diversity of wild food plants is higher than that of cultivated food crops [11,12]. Native flora is an important resource for many communities in Kenya [13,14,15]. Its exploitation can be a buffer against periodical famines [15,16], since it can improve the nutrition of many people through the provision of vitamins, minerals, etc. Deficiencies of vitamin A, which can be obtained from wild fruits, are among the major health concerns in many children, especially in developing countries [17]. Many wild plants eaten in Kenya have been demonstrated to contain energy, fats, proteins and other nutrients [18].

People are very selective and rely heavily on certain taxa for food and medicine [19]. The selection of medicinal plants is based on diverse reasons, i.e., a tradition passed from one generation to another, the efficacy of the plants, cross-cultural acquisition of knowledge, the abundance of plants, the symbolic resemblance of plant parts to certain body organs, and the taxonomic affiliation of plants to known medicinal taxa [20]. However, plant-based medicine is not just symbolic and placebo [21]. To find consistent patterns of plant uses, ethnobotanical studies have applied various metrics, such as informant consensus factor, the relative frequency of citation, use reports, and fidelity levels [22,23,24]. However, those indices are not independent from each other, and thus behave similarly. They are also not grounded in well-supported statistical approaches, and thus not robust enough to lead into discovery of new drugs [25,26]. Understanding plant use patterns cannot be achieved through species counts since they tend to overemphasize large families, while percentages of medicinal species overemphasize small families [27]. If medicinal plants were selected at random, the proportion of plants selected from each family would be equal [28]. Quantitative analysis based on least squares regression found that some families comprise a higher number of medicinal species than would be expected from a null model of a linear relationship between the total number of species in a family and the observed medicinal species [29]. This model was applied by Moerman [30], when analyzing patterns of plant uses by native Americans, where bias towards uses of certain taxa was identified, demonstrating that medicinal properties were not randomly distributed in plants. Plants used for the treatment of diseases portray a phylogenetic clustering [31], and this has been supported by the detection of phylogenetic signals in seed plants due to the presence of related secondary metabolites [32]. A number of studies carried out worldwide support the perspective that certain plant families are heavily relied on for medicinal uses, e.g., in North America [19], Mexico [33], South Africa, Nepal and New Zealand [34], South Africa [28], Southern Belize [35], and Thailand [29].

Linear regression, binomial, and Bayesian models have been widely applied in detecting outliers in floras [19,20,21,29,30,36,37]. Linear regression approach evaluates outliers by regressing the number of useful species against the total number of species per taxon, then interpreting the resultant least squares line as a measure of average relationship between taxa sizes and the number of useful plants. Regression residuals obtained are used to quantify the extent to which taxa have unexpectedly high or low number of useful species relative to the overall proportion of species in a flora [19,20,21,36]. However, this approach lacks tests of statistical hypotheses and prowess to measure uncertainties for estimates or control error rates associated with over- and under-use of taxa. Regression approach is also subjective in assessing what constitutes a large or a small residual [20], thus a new metric was coined to be used alongside the residuals [29]. Regression model is also biased towards large families, assumes a linear relationship between useful species and total species in taxa [37], and it is also unclear whether more outliers would result from the overall proportion in a given flora than one would expect by chance [20]. Binomial analysis was thus introduced, and was first used in highlighting outstanding medicinal taxa in Ecuadorian Shuar flora using contingency tables. This model provides tests of statistical hypotheses by assessing whether distribution of useful plants among taxa would depart significantly from a homogenous null distribution, which is analogous to analyzing overall significance of an ANOVA model, then testing for the significance of individual parameters. Binomial model relies on a test statistic with a binomial distribution, that assumes that the proportion of useful plants in taxa equals the proportion of useful plants in the whole flora, and treats each species within taxa as a random trial with a probability of being utilized or not [20]. Although binomial approach considers the stochastic process of the number of species used, it treats the proportion of useful species in the flora as a fixed number, which is in fact an estimated value with inherent uncertainties, since it is obtained from sampled data. To accommodate these uncertainties, Bayesian model treats the proportion of useful species in taxa as a random variable, with values ranging from 0–1, and with a certain probability known as prior probability. These values are assumed to be uniformly distributed prior to sampling since there is no sufficient ground to believe that some taxa would have a lower or higher probability of being selected. The prior probability is updated to posterior probability, after sampling and analyzing the total and useful species in each taxon. Since proportion of all useful species in the local flora is a random variable, it is also assigned a posterior probability. Posterior probability distributions of useful species in each taxon and in the whole flora are used to judge if certain taxa depart from the expected proportion of useful species [37]. The combination of the three approaches is thus recommended to avoid biases when dealing with small to medium-sized samples [29,37]. These approaches are important when exploring higher taxonomic groups to identify important taxa and in comparing unrelated cultures by discounting ineffective taxa [34].

If medicinal plants are selected independently more than once, their case for efficacy is stronger, and in this perspective, common patterns have been found in different ethnomedicinal floras pertaining to diverse cultures [34]. However, close cultures may undergo transmission of ethnomedicinal knowledge, thus distorting the independent cultural consensus on the use of those plants and, in turn, affecting anticipated biological activities [34]. Understanding why different plants produce different secondary metabolites is paramount for bioprospecting [28]. The high cost of research and development of novel drugs necessitates optimization of the drug bioprospecting phase to help identify ethnomedicinal taxa possessing biological activities, especially when the resource base is extensive and diverse [28,34]. One such extensive resource base is the flora of Kenya, which is distributed in seven floral regions (K1–K7) [38]. Medicinal and food plants are diverse and important resources in Kenya [39,40]. Plants found in dry areas are perceived to have better medicinal qualities [41], while wild fruits are most important to people living in arid areas [42]. This suggests that Kenya, 80% of whose landmass is under arid and semi-arid conditions, which are also some of the most botanically diverse habitats with over 5000 indigenous species [43,44], is an important region for evaluating the selection behavior of useful plants. Kenya is also endowed with culturally diverse ethnic minorities [45], who have potentially played a role in shaping her present traditional plant use patterns. Plant selection behavior pertaining to medicinal and food taxa is not well understood in Kenya. At a global scale, studies have mostly focused on unraveling relationships between total floras and ethnomedicinal floras, thus paying little attention to comparative analyses between medicinal and food floras. Based on three quantitative approaches: Regression, Bayesian, and Binomial analyses, the objective of this study was to test whether medicinal and food taxa in Kenya are selected randomly by the users, since the flora of Kenya has never been explored in this perspective.

## 2. Results

### 2.1. Medicinal Orders

**Figure 1:**
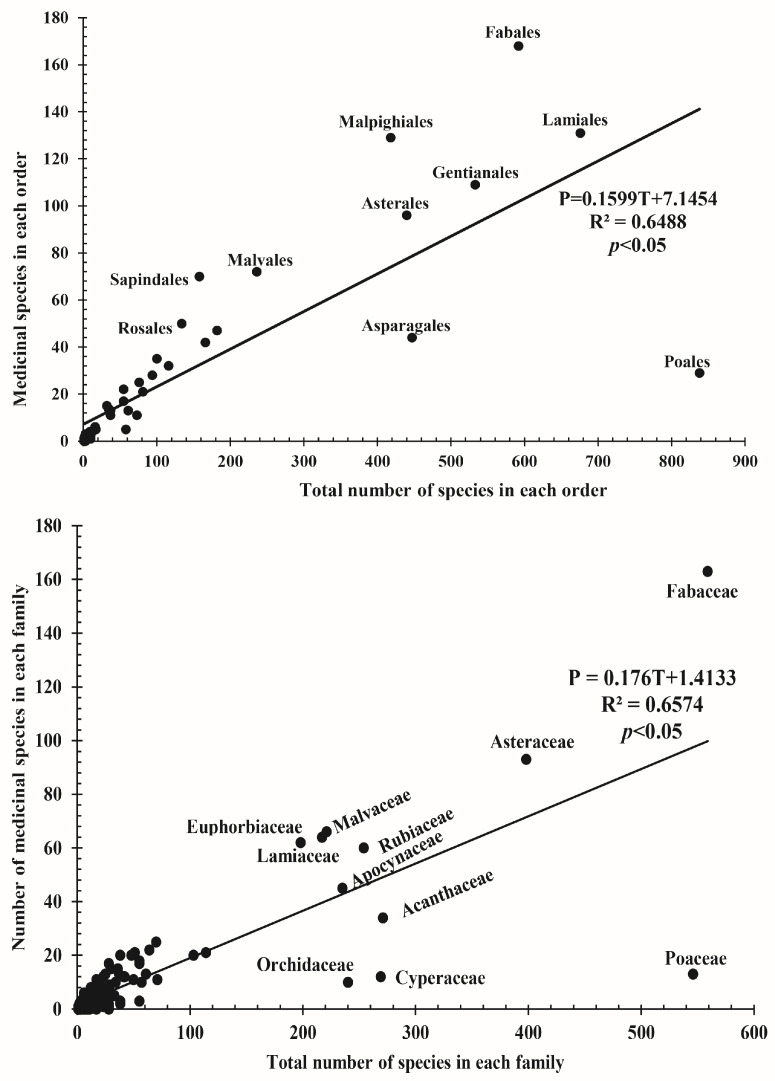
Linear regression model showing the relationship between the number of medicinal species and the total number of species for orders and families.

The modeled linear relationship between predicted medicinal taxa and the total number of species was given as P = 0.1599T + 7.1454 (for orders) and P = 0.176T + 1.4133 (for families). The two equations explained 64.88% and 65.74% of the variation in y-values, respectively, and the total number of species in the flora was a significant predictor of medicinal species in both orders and families (*p* < 0.05; Figure 1). Cumulatively, the three models identified 14 important medicinal orders; only six of those orders (Fabales, Malpighiales, Malvales, Sapindales, Mrytales, and Rosales) were recovered under all models. The regression model recovered six positive outliers, while binomial and Bayesian models identified 14 and 12 orders, respectively. Fabales had the highest residuals (66.16), followed by Malpighiales (55). Under the regression model, Sapindales had the highest R-value (1.1605), followed by Rosales (0.7507). All the 14 orders identified by the three models were recovered under the binomial model with significant values (*p* < 0.05; Table 1).

### 2.2. Medicinal Families

The three models identified 38 positive outliers, whereas 31 outliers were identified by all the models. The bayesian approach identified 31 families, while the binomial approach identified all 34 positive outliers (*p* < 0.05). The regression approach identified 32 positive outliers, but it did not recover Canellaceae, Peraceae, Phytolaccaceae, Pittosporaceae, Salvadoraceae, and Typhaceae, which were identified by the other two models. Rutaceae (1.6808) had the highest R-value, followed by Oleaceae (1.497). Fabaceae had the highest (63.2) residuals, followed by Lamiaceae 25.74 (Table 2).

### 2.3. Food Orders

**Figure 2:**
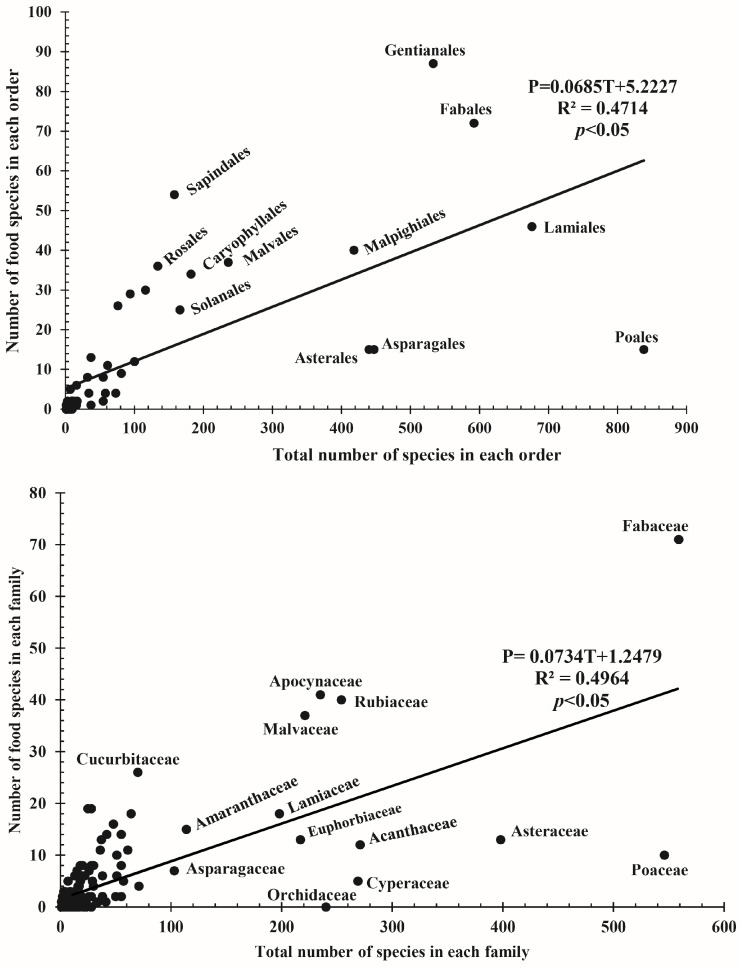
Linear regression model showing the relationship between the number of food species and the total number of species for orders and families.

Under the regression model, the predicted number of food taxa was recovered under the equations: P = 0.0685T + 5.2227 (for orders) and P = 0.0734T + 1.2479 (for families). The two models explained 47.14% and 49.64% of the variation in y-values, respectively, and the number of total species in the flora was a significant predictor of food species in both orders and families (*p* < 0.05; Figure 2). Sixteen positive outliers were recovered by the three approaches, where seven of them were recovered by all three models. Both binomial and Bayesian models recovered 13 positive outliers each. The linear regression model recovered 11 positive outliers, where Sapindales (2.3654) had the highest R-value, followed by Rosales (1.4997). Solanales and Fabales were identified by the regression model only. Gentianales (45.27) and Sapindales (37.95) had the highest regression residuals (Table 3).

### 2.4. Food Families

The three models recovered 42 positive outliers; 27 families were recovered by all three models (*p* < 0.05). The regression model identified 35 positive outliers, where Anacardiaceae (5.163) had the highest R-value, followed by Sapotaceae (4.7522). Fabaceae had the highest regression residual (28.72), and was only recovered as an outlier under regression approach. Binomial and Bayesian models identified 30 (*p* < 0.05) and 34 positive outliers, respectively (Table 4).

### 2.5. Taxa Shared between Medicinal and Food Uses

Of the 21 orders recovered, only Celastrales, Cucurbitales, Ericales, Magnoliales, Malvales, Rosales, and Sapindales were significant outliers for both food and medicinal categories. Fabales was shared between the two categories, although it was not a significant positive outlier food taxon. In total, 59 families were recovered, with 21 common positive outliers, of which 14 (Amaranthaceae, Anacardiaceae, Annonaceae, Capparaceae, Celastraceae, Cucurbitaceae, Malvaceae, Moraceae, Olacaceae, Rhamnaceae, Rutaceae, Salicaceae, Salvadoraceae, Sapindaceae) were significant positive outliers.

## 3. Discussion

Malphigiales and Fabales, which contain Euphorbiaceae and Fabaceae, respectively, were recovered as positive medicinal outliers with significant values. In South Africa, Malphigiales was also recovered as a positive outlier, with Euphorbiaceae influencing its outlier status most [28]. Euphorbiaceae is an important medicinal taxon since its members contain terpenoids and derivatives, aliphatics, and alkaloids as abundant compounds and at least 1.7% of all major compounds [28]. In our study, Malphigiales comprised four significant positive outlier medicinal families (Salicaceae, Euphorbaceae, Hypericaceae, and Phyllanthaceae), and all of them were recovered with positive regression residuals in South Africa [28]. Three Sapindales families, i.e., Sapindaceae, Meliaceae, and Anacardiaceae, which were significant positive medicinal outliers, were also recovered with positive residuals in South Africa [28]. Anacardiaceae comprise flavonoids, aliphatics, and terpenoids as the major compounds [28]. Rutaceae was also a significant positive outlier in our study, and previous studies support its importance in traditional medicine, e.g., alkaloid nitidine extracted from *Zanthoxylum asiaticum* (L.) Appelhans, Groppo & J. Wen root bark has a high antimalarial activity and is the main chemical component in many Rutaceae species [46]. Nitidine was first isolated from *Zanthoxylum zanthoxyloides* (Lam.) Zepern. & Timler, and received considerable attention as a potential anticancer drug after it was discovered to be potent against leukemia in mice [47]. To date, over 400 secondary metabolites have been discovered in *Zanthoxylum* species, with alkaloids and terpenoids being the major compounds [48]. In *Fagaropsis*, 18 main phytocompounds are known, and flavonoids, terpenoids, and alkaloids are among the compounds responsible for its medicinal properties [49]. Isolation and characterization of 21 secondary metabolites from *Clausena anisata* (Willd.) Hook.f. ex Benth. revealed that imperatorin had the highest anti-bacterial activity. Some *C. anisata* extracts such as murrayamine-A, gravelliferone, and excavatin had activities against HeLa cells (LC50 = 1.14–3.26 μg/mL), with good selectivity index values (SI 38.20–231.58) and those compounds are not toxic to normal Vero cells (LC50 = 69.15–434.78 μg/mL), indicating their potential in the development of anticancer drugs [50]. Related medicinal plants portray efficacy against human diseases due to inheritable similarities in the secondary metabolites [28], endorsing the idea of phylogenetic based bioprospecting. Similar to this study, Malvales was recovered as a positive outlier medicinal taxon in South Africa. Moreover, Rosales was recovered as a positive medicinal outlier in our study, while Solanales, Asterales, and Gentianales were not recovered as significant positive outliers, unlike in South Africa [28]. Orders and families shared for both food and medicine suggest possibility of generalist taxa. Moerman [19] also found a substantial overlap between drug and food plants in North America. However, the observed differences suggest that the two categories have independent applications.

Fabaceae, Euphorbiaceae, Malvaceae and Lamiaceae, which are among the largest medicinal families in Kenya [14,51], were recovered as positive outliers, although Fabaceae was not a significant outlier, which agrees with another study in South Africa [34]. However, Fabaceae is an important medicinal family in China and Nepal [34,52]. All the medicinal families recovered as important in our study were also reported in Thailand [29]. This relationship might be due to shared traditional knowledge between the two regions or due to the presence of the same important lineages [52]. However, Euphorbiaceae, which was a significant positive outlier in our study, was not recovered as a significant outlier in South Africa [34]. This is expected because the two regions comprise different floras and cultures. Each family may also have substantially different species compositions in different regions [34], thus influencing how its species are picked. Asteraceae, the second-largest medicinal family in Kenya, was not recovered as a positive outlier by any of the three models. Based on floral studies in Kenya [53,54], Asteraceae is abundant in the highlands, where it is moist. Lowland plants, mostly found in arid areas, are perceived to have better medicinal properties [41]. A previous study supported that the accumulation of chemical compounds in plants is influenced by edaphic factors [52]. Although water stress may play an important role in increasing the quantities of secondary metabolites in many plants [55], the scale of our study is not sufficient to address this perspective. However, Asteraceae is an important medicinal family in North America and South Africa [27,28]. Rutaceae had the highest R-value, which agrees with results obtained from Thailand, where it was among the families with the highest R-values [29]. Some medicinal families recovered here as positive medicinal outliers have been reported elsewhere in the world, e.g., Rutaceae, Lamiaceae, Araliaceae, and Cucurbitaceae in Daodi pharmacopeia in China [52]; Amaranthaceae in Ecuador [20]; Anacardiaceae in South Africa [28,34]; Malvaceae and Solanaceae in Nepal, New Zealand, and South Africa [28,34]; Lamiaceae in Mexico, Nepal, New Zealand, North America, Korea and Ecuador [19,20,33,34,36]; Fabaceae in Mexico, Nepal, New Zealand, and South Africa [28,33,34]; Euphorbiaceae in Mexico, Nepal, and South Africa [28,33,34]; and Rutaceae in Nepal [34]. Acanthaceae, Cactaceae and Juncaceae were among the negative outlier medicinal families in North America [27], which agrees with the results of our study. In Southern Belize and China, Cyperaceae and Orchidaceae were also among the negative outlier medicinal families [35,52]. Orchidaceae is the largest family globally, but most orchids are rare epiphytes and thus not easily accessible for medicinal uses [35]. However, there are differences in the identification of important medicinal taxa globally. For example, in North America, Rosaceae was identified as a positive outlier, while Euphorbiaceae, Malvaceae, and Fabaceae were identified as negative outliers using regression residuals, contrary to our results [27]. This convergent uses of medicinal families in different regions by unrelated cultures strongly indicates their efficacy and further highlights their importance in different cultures suggesting the need to prioritize them in bioscreening and conservation since they present a potential for the discovery of new or overlooked medicinal species [28,34].

Since previous studies have largely focused on important medicinal taxa, Saslis–Lagoudakis et al. [34] recommended analyses of floras to identify other important taxa, such as food plants. Here, 14 positive outlier food orders and 27 positive outlier food families were recovered with significant values. In North America, Rosaceae, Fabaceae and Poaceae, which produce human foods, were avoided as medicinal sources, but Solanaceae was important in both uses since most of its members are important foods globally [19]. However, Fabaceae and Solanaceae were only recovered as positive outliers under the regression model and thus were not significantly used for food. Moerman [27] argued that since grasses do not produce significant quantities of secondary metabolites, they comprise the majority of the food plants that humans and animals depend on in North America. Though the two floras are different, our study does not support this perspective since Poaceae was a negative outlier. Cyperaceae and Scrophulariaceae, which were among the negative outlier food taxa, were also ranked among the least important food families in North America using regression residuals [19]. Polygonaceae and Rosaceae were ranked among the top food families in North America, while Rubiaceae and Malvaceae were ranked among the least. Those four families were recovered as positive outliers by all three models. Lamiaceae was only recovered as a positive outlier under the regression model, but it was not significantly used for food, unlike in North America, where most of the aromatic plants were used for tisanes [19]. Unlike in North America, Apiaceae, Cactaceae, and Liliaceae were not recovered as positive outliers in Kenya, while similar to North America, Euphorbiaceae, Asteraceae, and Campanulaceae are not important food families in Kenya.

## 4. Materials and Methods

### 4.1. Study Area

**Figure 3:**
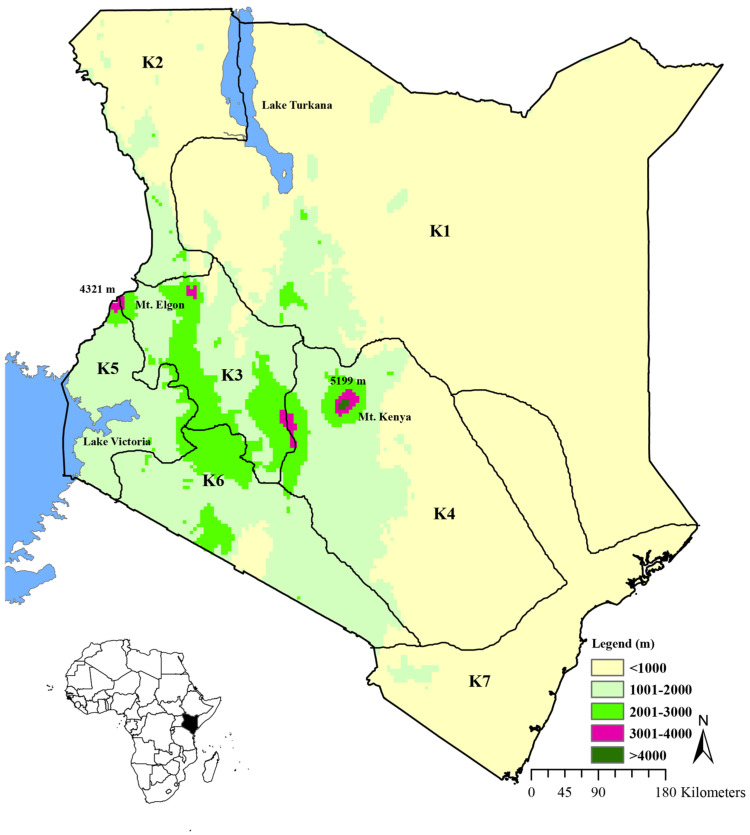
Map of Kenya (study area) (K1–K7 represents the seven floral regions of Kenya).

Kenyan landmass covers an area of about 582,000 km^2^ (Figure 3), with an altitude raising from sea level through a series of plateaus to the highest point in Mount Kenya, about 5200 m above sea level [56]. The low-lying areas receive low rainfall and are thus hotter [57]. Kenya is divided into 7 floral regions [38]. The climate of Kenya is of tropical monsoon, belonging to tropical savanna climate, where the annual temperatures are in the range of 22–26 °C, while the annual minimum temperatures are in the range of 10–14 °C [58]. The highest temperatures are experienced in northwest and northern Kenya, where night temperatures can be 29 °C and the mean annual temperatures can reach above 34 °C [56]. Rainfall patterns in Kenya vary according to different seasons throughout the year, where most regions experience high rainfall from March to June, and October to December, with the annual rainfall decreasing from 2000–250 mm from southwest to northeast Kenya [58]. Habitat types in Kenya include wooded grasslands, bushlands, grasslands, semi-deserts, afro-alpine, mangroves, inland wetlands, etc. [59,60]. There are 3 vegetation types in Kenya: forest types, grasslands, and semi-arid grasslands [60]. Most of the area in Kenya is arid to semi-arid, where the climax vegetation comprises bushland, wooded grassland and desert scrub, while the transitional zones with higher rainfall comprise woodlands [44,56]. The vegetation of many dry areas in Kenya is characterized by occasional baobab trees and low, stunted, dense thorn bushes with thick undergrowth [57], consisting of scrublands and woodlands [61]. The Kenyan coast comprises about 50% of forest-dependent plant species, which are nationally threatened, making it the most important area for conservation despite its small area [56]. Most of the arid areas in Kenya lack forests except on hills [57], where species endemism is high [56,62]. Dry forests in Kenya consist of *Drypetes* spp., *Combretum* spp., *Vepris* spp., *Croton* spp., *Euphorbia* spp., *Dombeya* spp., and *Commiphora* spp. [62,63].

In Kenya, most of the plants used locally for traditional medicine and food are obtained from the forests, proving the significance of biodiversity to the livelihoods of local inhabitants [51,64]. Many forests in dry environments are affected by harsh conditions and are thus deciduous, with low species diversity and a reduced canopy, and may have low tree density under extreme droughts. Many forests have been depleted over the years due to human activities; hence their species diversity is lower compared to the previous decades [63]. This destruction may escalate in the future due to difficult climate manifestation in East Africa, where warming is likely to lead to dryness in some areas and higher precipitation in others [65]. Based on these arguments, the utilization of forest resources needs to be well documented, understood, and properly managed if future generations are expected to benefit from them. Kenya comprises 42+ tribes, and some groups are composed of subtribes [45,66]. Those groups have renown traditional knowledge regarding the use of plants [51,67,68]. Since over 80% of the Kenyan land mass is under arid and semiarid conditions [44], the majority of the people live in arid areas, depending on livestock farming, and their lives may be constrained by frequent droughts [69], hence influencing their economic lifestyles; thus, some of them have commercialized useful plants [51,70].

### 4.2. Floral and Ethnobotanical Data Collection

An elaborate independent literature search in google scholar, web of science, semantic scholar, science direct, PubMed, Scopus, Scientific Electronic Library Online, and Google search was conducted to gather information on medicinal and food plants of Kenya. Keywords like ethnobotany, useful plants, traditional knowledge, medicinal plants, and food plants, combined with Kenya, were used to aid in the data search. Where possible, we added studies cited by already retrieved studies if the reported ethnobotanical work was carried out in Kenya. Since some ethnomedicinal materials collected data from both traditional healers/herbalists and non-specialists of traditional medicine, we merged all the materials into 1 data set. Ethnomedicinal data were obtained from 74 articles [7,14,51,64,66,67,68,71,72,73,74,75,76,77,78,79,80,81,82,83,84,85,86,87,88,89,90,91,92,93,94,95,96,97,98,99,100,101,102,103,104,105,106,107,108,109,110,111,112,113,114,115,116,117,118,119,120,121,122,123,124,125,126,127,128,129,130,131,132,133,134,135,136,137], 4 ethnobotanical reports [138,139,140,141], 3 books [142,143,144], and 11 dissertations (includes MSc. and Ph.D.) [41,145,146,147,148,149,150,151,152,153,154]. Data on food plants were obtained from 19 articles [12,13,14,16,18,100,114,134,155,156,157,158,159,160,161,162,163,164,165,166] and 2 books [142,167]. The materials obtained were published between 1966 and 2021. We traced all the use reports to their original sources to remove repeated publications. Retrieved studies were entered in Microsoft Excel and screened manually to remove those with taxonomic errors. Where possible, we verified all the scientific names to ensure that they existed in the floral regions where the studies were carried out. Although our survey may not cover all the food and medicinal plant uses in Kenya, it represents a reliable sample due to its extensive geographical coverage. Data obtained were treated as a census rather than a sample of medicinal and food uses since it is more useful in understanding human behaviors compared to analyses of sample sizes [30]. Data on the flora of Kenya were obtained from Zhou et al. [38], and updated according to Plants of The World Online (https://powo.science.kew.org/ accessed on 1 February–31 March 2021). Plant orders were obtained from the Global Biodiversity Information Facility (https://www.gbif.org/ accessed on 1–30 April 2021). Plants not determined to species level and introduced species were removed during analyses. Infraspecific taxa were merged with the parent species. Fifty orders, 188 families, and 5883 species represented the total flora of Kenya. In total, 1298 medicinal plants (Appendix A) and 664 food plants (Appendix B) were subjected to quantitative analysis.

### 4.3. Quantitative Methods

#### 4.3.1. Regression Analysis

Regression analysis was carried out using Moerman’s LINEST linear regression function approach in MS Excel 2016, according to Bennett and Husby [20]. The expected numbers of medical and food species were recovered under the equation: y = bx + m; both b and m were obtained using the LINEST function. Regression residuals were used to analyze a simple plot of the number of medicinal and food species against the total number of species in each category [29]. Since this approach violates the statistical assumptions of homoscedasticity and normality when used for analyzing useful floras [20], we applied the metric ‘relative regression residual (R)’ [29]; where R is the relative difference between the observed number of medicinal/food species (O) and predicted number (P) of medicinal/food species under the model (P): R = (O − P)/P. We used the critical range of R-values to filter random noise, which could result from data sets assembled from many sources, and thereafter identified positive outliers as those with R > 0.5 [29].

#### 4.3.2. Binomial Analysis

To test for the significance of individual taxa departure from the expected number of medicinal/food plants, we conducted a binomial analysis to obtain *p*-values for the positive outliers using the BINOMDIST function in MS Excel 2016 [20]. To obtain the significant values, we calculated 2 probabilities:

(a) The probability that the number of medicinal and food plants in each category is equal to the expected;
(b) The probability that the number of medicinal and food species in each category is equal to or more than the expected value.

The 95% interval probability that the number of medicinal/food species is higher than expected was calculated as *p* = (1 + a) − b [20].

#### 4.3.3. Bayesian Analysis

We conducted a Bayesian analysis using BETA.INV function in MS Excel 2016 [37]. Here, we calculated the inferior (inf.) 95% probability credible intervals and the superior (sup.) 95% probability credible intervals. Taxa with inferior 95% probability credible intervals higher than the superior 95% probability credible intervals obtained for all species were considered important (Bayesian inferior and superior 95% probability credible interval values obtained for all categories analyzed are enclosed in brackets in the results).

## 5. Conclusions

Using linear regression, Binomial and Bayesian analyses, our results add information to the literature regarding outstanding medicinal and food plant taxa in Kenya. By comparing our results with those from other studies elsewhere in the world, we have contributed towards the synthesis of taxa that might prove to be important candidates for future bioscreening of useful chemical compounds. The comparison of outlier medicinal and food taxa further highlights that some taxa are more important in Kenya than previously known. In the future, we suggest a phylogenetic evaluation of orders and families identified as important for medicinal uses to clearly understand the relationships between individual taxa. We also suggest comparative analyses of plants used by different ethnic minorities in Kenya and between different regions, especially in tropical Africa, to unravel the cultural interrelationships in those regions.

## Figures and Tables

**Table 1:** Quantitative analysis of medicinal species in orders. T—total number of species in the flora; M—medicinal species; ↑/↓—positive/negative outliers based on residuals; P_a_—predicted medicinal species under regression model; Res.—regression residuals; P_b_—predicted medicinal species under binomial model; inf.—Bayesian inferior 95% probability credible intervals; sup.—Bayesian superior 95% probability credible intervals; *—positive outliers.

			Regression Analysis			Binomial Analysis		Bayesian Analysis	
Order	T	M	P_a_	Res.	R-Value	P_b_	*p*-Value	inf. (0.2102)	sup. (0.2314)
Fabales	592	168 ↑	101.84	66.16	0.6497 *	130.59	0.0002 *	0.249 *	0.3214
Malpighiales	418	129 ↑	74	55	0.7433 *	92.21	0 *	0.2663 *	0.3545
Sapindales	158	70 ↑	32.4	37.6	1.1605 *	34.85	0 *	0.3678 *	0.5211
Malvales	236	72 ↑	44.88	27.12	0.6043 *	52.06	0.0016 *	0.2499 *	0.3667
Rosales	134	50 ↑	28.56	21.44	0.7507 *	29.56	0 *	0.2958 *	0.4577
Asterales	440	96 ↑	77.52	18.48	0.2384	97.06	0.5672	0.1822	0.2592
Gentianales	533	109 ↑	92.4	16.6	0.1797	117.58	0.8283	0.1725	0.2408
Lamiales	676	131 ↑	115.28	15.72	0.1364	149.12	0.9596	0.1658	0.2253
Myrtales	100	35 ↑	23.12	11.88	0.5139 *	22.06	0.0021 *	0.2636 *	0.4477
Caryophyllales	182	47 ↑	36.24	10.76	0.2969	40.15	0.129	0.2002	0.3265
Solanales	166	42 ↑	33.68	8.32	0.2471	36.62	0.1796	0.193	0.3244
Brassicales	116	32 ↑	25.68	6.32	0.2461	25.59	0.0951	0.2028	0.3636
Apiales	55	22 ↑	15.92	6.08	0.382	12.13	0.0021 *	0.281 *	0.5325
Ericales	94	28 ↑	22.16	5.84	0.2636	20.74	0.0498 *	0.2149	0.3971
Cucurbitales	76	25 ↑	19.28	5.72	0.2967	16.77	0.0194 *	0.2338 *	0.441
Magnoliales	32	15 ↑	12.24	2.76	0.2256	7.06	0.0016 *	0.308 *	0.6365
Ranunculales	34	14 ↑	12.56	1.44	0.1147	7.5	0.0096 *	0.2632 *	0.5789
Vitales	55	17 ↑	15.92	1.08	0.0679	12.13	0.0814	0.2029	0.441
Santalales	81	21 ↑	20.08	0.92	0.0459	17.87	0.2365	0.1764	0.3644
Celastrales	37	13 ↓	13.04	−0.04	−0.003	8.16	0.0479 *	0.2181	0.5135
Saxifragales	37	11 ↓	13.04	−2.04	−0.1564	8.16	0.1753	0.175	0.459
Zygophyllales	16	6 ↓	9.68	−3.68	−0.3801	3.53	0.1201	0.1844	0.6167
Boraginales	61	13 ↓	16.88	−3.88	−0.2298	13.46	0.6057	0.1293	0.3318
Zingiberales	9	4 ↓	8.56	−4.56	−0.5327	1.99	0.1153	0.1871	0.7376
Icacinales	3	3 ↓	7.6	−4.6	−0.6052	0.66	0.0107 *	0.3976 *	0.9937
Geraniales	16	5 ↓	9.68	−4.68	−0.4834	3.53	0.2672	0.1421	0.5596
Piperales	17	5 ↓	9.84	−4.84	−0.4918	3.75	0.315	0.1334	0.5348
Oxalidales	12	4 ↓	9.04	−5.04	−0.5575	2.65	0.2627	0.1386	0.6143
Laurales	8	3 ↓	8.4	−5.4	−0.6428	1.76	0.25	0.137	0.7007
Canellales	2	2 ↓	7.44	−5.44	−0.7312	0.44	0.0487 *	0.2924 *	0.9916
Arecales	7	2 ↓	8.24	−6.24	−0.7573	1.54	0.4791	0.0852	0.6509
Proteales	7	2 ↓	8.24	−6.24	−0.7573	1.54	0.4791	0.0852	0.6509
Aquifoliales	1	1 ↓	7.28	−6.28	−0.8626	0.22	0.2206	0.1581	0.9874
Buxales	1	1 ↓	7.28	−6.28	−0.8626	0.22	0.2206	0.1581	0.9874
Liliales	8	2 ↓	8.4	−6.4	−0.7619	1.76	0.5555	0.0749	0.6001
Dilleniales	2	1 ↓	7.44	−6.44	−0.8656	0.44	0.3925	0.0943	0.9057
Gunnerales	2	1 ↓	7.44	−6.44	−0.8656	0.44	0.3925	0.0943	0.9057
Nymphaeales	3	1 ↓	7.6	−6.6	−0.8684	0.66	0.5265	0.0676	0.8059
Dioscoreales	10	2 ↓	8.72	−6.72	−0.7706	2.21	0.6832	0.0602	0.5178
Fagales	4	1 ↓	7.76	−6.76	−0.8711	0.88	0.631	0.0527	0.7164
Cornales	5	1 ↓	7.92	−6.92	−0.8737	1.1	0.7124	0.0433	0.6412
Pandanales	5	1 ↓	7.92	−6.92	−0.8737	1.1	0.7124	0.0433	0.6412
Metteniusales	1	0 ↓	7.28	−7.28	−1	0.22	0.7794	0.0126	0.8419
Ceratophyllales	2	0 ↓	7.44	−7.44	−1	0.44	1	0.0084	0.7076
Vahliales	3	0 ↓	7.6	−7.6	−1	0.66	1	0.0063	0.6024
Dipsacales	10	1 ↓	8.72	−7.72	−0.8853	2.21	0.9173	0.0228	0.4128
Commelinales	73	11 ↓	18.8	−7.8	−0.4149	16.1	0.9486	0.0867	0.2504
Alismatales	58	5 ↓	16.4	−11.4	−0.6951	12.79	0.9981	0.0382	0.1868
Asparagales	447	44 ↓	78.64	−34.64	−0.4405	98.61	1	0.0742	0.1296
Poales	838	29 ↓	141.2	−112.2	−0.7946	184.86	1	0.0243	0.0493

**Table 2:** Quantitative analysis of medicinal species in families. T—total number of species in the flora; M—medicinal species; P_a_—predicted medicinal species under the regression model; ↑/↓—positive/negative outliers based on residuals; Res.—regression residuals; P_b_—predicted medicinal species under binomial model; inf.—Bayesian inferior 95% probability credible intervals; sup.—Bayesian superior 95% probability credible intervals; *—positive outliers.

			Regression Analysis			Binomial Analysis		Bayesian Analysis	
Family	T	M	P_a_	Res.	R-Value	P_b_	*p*-Value	inf. (0.2102)	sup. (0.2314)
Fabaceae	559	163 ↑	99.8	63.2	0.6333 *	123.34	0.0001 *	0.2555 *	0.3306
Lamiaceae	198	62 ↑	36.26	25.74	0.7098 *	43.69	0.0016 *	0.2526 *	0.3809
Malvaceae	221	66 ↑	40.31	25.69	0.6373 *	48.76	0.0042 *	0.2422 *	0.3621
Euphorbiaceae	217	64 ↑	39.61	24.39	0.6159 *	47.88	0.0065 *	0.2383 *	0.3589
Asteraceae	398	93 ↑	71.46	21.54	0.3014	87.81	0.2829	0.1948	0.2777
Rubiaceae	254	60 ↑	46.12	13.88	0.301	56.04	0.2969	0.1882	0.2922
Combretaceae	38	20 ↑	8.1	11.9	1.4687 *	8.38	0 *	0.3718 *	0.6758
Cucurbitaceae	70	25 ↑	13.73	11.27	0.8204 *	15.44	0.0064 *	0.255 *	0.4746
Rutaceae	28	17 ↑	6.34	10.66	1.6808 *	6.18	0 *	0.4226 *	0.7648
Phyllanthaceae	51	21 ↑	10.39	10.61	1.0213 *	11.25	0.0017 *	0.2873 *	0.549
Solanaceae	51	21 ↑	10.39	10.61	1.0213 *	11.25	0.0017 *	0.2873 *	0.549
Capparaceae	48	20 ↑	9.86	10.14	1.0281 *	10.59	0.0018 *	0.2882 *	0.5579
Amaranthaceae	64	22 ↑	12.68	9.32	0.7354 *	14.12	0.0162 *	0.2392 *	0.4665
Annonaceae	30	15 ↑	6.69	8.31	1.241 *	6.62	0.0007 *	0.3306 *	0.6694
Sapindaceae	36	15 ↑	7.75	7.25	0.9357 *	7.94	0.0066 *	0.271 *	0.579
Anacardiaceae	25	13 ↑	5.81	7.19	1.2363 *	5.52	0.001 *	0.3337 *	0.7007
Moraceae	55	18 ↑	11.09	6.91	0.6226 *	12.13	0.045 *	0.2181	0.4596
Oleaceae	17	11 ↑	4.41	6.59	1.497 *	3.75	0.0002 *	0.4099 *	0.827
Meliaceae	23	12 ↑	5.46	6.54	1.1973 *	5.07	0.0015 *	0.3282 *	0.7088
Vitaceae	55	17 ↑	11.09	5.91	0.5325 *	12.13	0.0815	0.2029	0.441
Rhamnaceae	18	10 ↑	4.58	5.42	1.1828 *	3.97	0.002 *	0.335 *	0.7555
Salicaceae	18	10 ↑	4.58	5.42	1.1828 *	3.97	0.002 *	0.335 *	0.7555
Celastraceae	37	13 ↑	7.93	5.07	0.6403 *	8.16	0.048 *	0.2181	0.5135
Araliaceae	12	8 ↑	3.53	4.47	1.2693 *	2.65	0.0012 *	0.3857 *	0.8614
Melastomataceae	15	8 ↑	4.05	3.95	0.9737 *	3.31	0.008 *	0.2988 *	0.7535
Bignoniaceae	6	6 ↑	2.47	3.53	1.4298 *	1.32	0.0001 *	0.5904 *	0.9964
Apiaceae	41	12 ↑	8.63	3.37	0.3906	9.05	0.1761	0.1762	0.4458
Burseraceae	42	12 ↑	8.81	3.19	0.3628	9.27	0.1998	0.1718	0.4367
Ebenaceae	20	8 ↑	4.93	3.07	0.6216 *	4.41	0.0545	0.2182	0.6156
Menispermaceae	16	7 ↑	4.23	2.77	0.6551 *	3.53	0.0438 *	0.2298	0.6708
Verbenaceae	22	8 ↑	5.29	2.71	0.5136 *	4.85	0.0914	0.1971	0.5727
Sapotaceae	28	9 ↑	6.34	2.66	0.4193	6.18	0.1453	0.1794	0.5083
Crassulaceae	34	10 ↑	7.4	2.6	0.3518	7.5	0.2007	0.1685	0.463
Cannabaceae	6	5 ↑	2.47	2.53	1.0249 *	1.32	0.0026 *	0.4213 *	0.9633
Urticaceae	29	9 ↑	6.52	2.48	0.3809	6.4	0.1716	0.1729	0.494
Hypericaceae	12	6 ↑	3.53	2.47	0.702 *	2.65	0.0309 *	0.2513 *	0.7487
Apocynaceae	235	45 ↑	42.77	2.23	0.0521	51.85	0.8773	0.1464	0.2467
Thymelaeaceae	14	6 ↑	3.88	2.12	0.5475 *	3.09	0.067	0.2127	0.6771
Polygonaceae	20	7 ↑	4.93	2.07	0.4189	4.41	0.1317	0.1811	0.5697
Rosaceae	26	8 ↑	5.99	2.01	0.3357	5.74	0.1981	0.1652	0.5018
Ranunculaceae	16	6 ↑	4.23	1.77	0.4187	3.53	0.1202	0.1844	0.6167
Zygophyllaceae	16	6 ↑	4.23	1.77	0.4187	3.53	0.1202	0.1844	0.6167
Icacinaceae	3	3 ↑	1.94	1.06	0.5454 *	0.66	0.0107 *	0.3976 *	0.9937
Olacaceae	3	3 ↑	1.94	1.06	0.5454 *	0.66	0.0107 *	0.3976 *	0.9937
Salvadoraceae	4	3 ↑	2.12	0.88	0.4169	0.88	0.0359 *	0.2836 *	0.9473
Boraginaceae	61	13 ↑	12.15	0.85	0.07	13.46	0.606	0.1293	0.3318
Loranthaceae	50	11 ↑	10.21	0.79	0.077	11.03	0.5595	0.1279	0.3532
Primulaceae	17	5 ↑	4.41	0.59	0.135	3.75	0.3151	0.1334	0.5348
Moringaceae	6	3 ↑	2.47	0.53	0.2149	1.32	0.1258	0.1841	0.8159
Oxalidaceae	6	3 ↑	2.47	0.53	0.2149	1.32	0.1258	0.1841	0.8159
Asparagaceae	103	20 ↑	19.54	0.46	0.0235	22.73	0.7755	0.1296	0.2813
Ochnaceae	18	5 ↑	4.58	0.42	0.0914	3.97	0.3638	0.1258	0.512
Loganiaceae	13	4 ↑	3.7	0.3	0.0807	2.87	0.3182	0.1276	0.581
Canellaceae	2	2 ↑	1.77	0.23	0.133	0.44	0.0487 *	0.2924 *	0.9916
Peraceae	2	2 ↑	1.77	0.23	0.133	0.44	0.0487 *	0.2924 *	0.9916
Phytolaccaceae	2	2 ↑	1.77	0.23	0.133	0.44	0.0487 *	0.2924 *	0.9916
Pittosporaceae	2	2 ↑	1.77	0.23	0.133	0.44	0.0487 *	0.2924 *	0.9916
Typhaceae	2	2 ↑	1.77	0.23	0.133	0.44	0.0487 *	0.2924 *	0.9916
Zingiberaceae	8	3 ↑	2.82	0.18	0.0633	1.77	0.2501	0.137	0.7007
Clusiaceae	3	2 ↑	1.94	0.06	0.0302	0.66	0.1246	0.1941	0.9324
Opiliaceae	3	2 ↑	1.94	0.06	0.0302	0.66	0.1246	0.1941	0.9324
Myrtaceae	9	3	3	0	0.0009	1.99	0.3175	0.1216	0.6525
Rhizophoraceae	9	3	3	0	0.0009	1.99	0.3175	0.1216	0.6525
Geraniaceae	15	4 ↓	4.05	−0.05	−0.0131	3.31	0.4298	0.1102	0.5238
Talinaceae	4	2 ↓	2.12	−0.12	−0.0554	0.88	0.2133	0.1466	0.8534
Aristolochiaceae	5	2 ↓	2.29	−0.29	−0.1279	1.1	0.3054	0.1181	0.7772
Santalaceae	23	5 ↓	5.46	−0.46	−0.0845	5.07	0.596	0.0977	0.4215
Plumbaginaceae	6	2 ↓	2.47	−0.47	−0.1901	1.32	0.3952	0.099	0.7096
Convolvulaceae	114	21 ↓	21.48	−0.48	−0.0222	25.15	0.854	0.1239	0.2655
Nyctaginaceae	12	3 ↓	3.53	−0.53	−0.149	2.65	0.5135	0.0909	0.5381
Piperaceae	12	3 ↓	3.53	−0.53	−0.149	2.65	0.5135	0.0909	0.5381
Adoxaceae	1	1 ↓	1.59	−0.59	−0.3708	0.22	0.2206	0.1581	0.9874
Aquifoliaceae	1	1 ↓	1.59	−0.59	−0.3708	0.22	0.2206	0.1581	0.9874
Berberidaceae	1	1 ↓	1.59	−0.59	−0.3708	0.22	0.2206	0.1581	0.9874
Buxaceae	1	1 ↓	1.59	−0.59	−0.3708	0.22	0.2206	0.1581	0.9874
Cactaceae	1	1 ↓	1.59	−0.59	−0.3708	0.22	0.2206	0.1581	0.9874
Flagellariaceae	1	1 ↓	1.59	−0.59	−0.3708	0.22	0.2206	0.1581	0.9874
Francoaceae	1	1 ↓	1.59	−0.59	−0.3708	0.22	0.2206	0.1581	0.9874
Hamamelidaceae	1	1 ↓	1.59	−0.59	−0.3708	0.22	0.2206	0.1581	0.9874
Lecythidaceae	1	1 ↓	1.59	−0.59	−0.3708	0.22	0.2206	0.1581	0.9874
Liliaceae	1	1 ↓	1.59	−0.59	−0.3708	0.22	0.2206	0.1581	0.9874
Monimiaceae	1	1 ↓	1.59	−0.59	−0.3708	0.22	0.2206	0.1581	0.9874
Musaceae	1	1 ↓	1.59	−0.59	−0.3708	0.22	0.2206	0.1581	0.9874
Myrothamnaceae	1	1 ↓	1.59	−0.59	−0.3708	0.22	0.2206	0.1581	0.9874
Penaeaceae	1	1 ↓	1.59	−0.59	−0.3708	0.22	0.2206	0.1581	0.9874
Arecaceae	7	2 ↓	2.65	−0.65	−0.2439	1.54	0.4792	0.0852	0.6509
Proteaceae	7	2 ↓	2.65	−0.65	−0.2439	1.54	0.4792	0.0852	0.6509
Passifloraceae	30	6 ↓	6.69	−0.69	−0.1036	6.62	0.6769	0.0959	0.3747
Pedaliaceae	13	3 ↓	3.7	−0.7	−0.1895	2.87	0.5721	0.0839	0.508
Basellaceae	2	1 ↓	1.77	−0.77	−0.4335	0.44	0.3926	0.0943	0.9057
Chrysobalanaceae	2	1 ↓	1.77	−0.77	−0.4335	0.44	0.3926	0.0943	0.9057
Dilleniaceae	2	1 ↓	1.77	−0.77	−0.4335	0.44	0.3926	0.0943	0.9057
Nymphaeaceae	2	1 ↓	1.77	−0.77	−0.4335	0.44	0.3926	0.0943	0.9057
Picrodendraceae	2	1 ↓	1.77	−0.77	−0.4335	0.44	0.3926	0.0943	0.9057
Smilacaceae	2	1 ↓	1.77	−0.77	−0.4335	0.44	0.3926	0.0943	0.9057
Velloziaceae	2	1 ↓	1.77	−0.77	−0.4335	0.44	0.3926	0.0943	0.9057
Cornaceae	3	1 ↓	1.94	−0.94	−0.4849	0.66	0.5266	0.0676	0.8059
Lauraceae	3	1 ↓	1.94	−0.94	−0.4849	0.66	0.5266	0.0676	0.8059
Myricaceae	3	1 ↓	1.94	−0.94	−0.4849	0.66	0.5266	0.0676	0.8059
Simaroubaceae	3	1 ↓	1.94	−0.94	−0.4849	0.66	0.5266	0.0676	0.8059
Araceae	26	5 ↓	5.99	−0.99	−0.1652	5.74	0.7098	0.0862	0.3808
Dichapetalaceae	9	2 ↓	3	−1	−0.3327	1.99	0.6236	0.0667	0.5561
Dioscoreaceae	9	2 ↓	3	−1	−0.3327	1.99	0.6236	0.0667	0.5561
Cleomaceae	15	3 ↓	4.05	−1.05	−0.2599	3.31	0.6752	0.0727	0.4565
Balsaminaceae	21	4 ↓	5.11	−1.11	−0.2171	4.63	0.7126	0.0782	0.4028
Hernandiaceae	4	1 ↓	2.12	−1.12	−0.5277	0.88	0.6311	0.0527	0.7164
Resedaceae	4	1 ↓	2.12	−1.12	−0.5277	0.88	0.6311	0.0527	0.7164
Malpighiaceae	10	2 ↓	3.17	−1.17	−0.3697	2.21	0.6833	0.0602	0.5178
Achariaceae	5	1 ↓	2.29	−1.29	−0.5639	1.1	0.7125	0.0433	0.6412
Colchicaceae	5	1 ↓	2.29	−1.29	−0.5639	1.1	0.7125	0.0433	0.6412
Putranjivaceae	5	1 ↓	2.29	−1.29	−0.5639	1.1	0.7125	0.0433	0.6412
Stilbaceae	5	1 ↓	2.29	−1.29	−0.5639	1.1	0.7125	0.0433	0.6412
Asphodelaceae	57	10 ↓	11.45	−1.45	−0.1263	12.58	0.8371	0.0987	0.2943
Connaraceae	6	1 ↓	2.47	−1.47	−0.595	1.32	0.7759	0.0367	0.5787
Molluginaceae	6	1 ↓	2.47	−1.47	−0.595	1.32	0.7759	0.0367	0.5787
Alismataceae	1	0 ↓	1.59	−1.59	−1	0.22	1	0.0126	0.8419
Alismataceae	1	0 ↓	1.59	−1.59	−1	0.22	1	0.0126	0.8419
Ancistrocladaceae	1	0 ↓	1.59	−1.59	−1	0.22	1	0.0126	0.8419
Aphloiaceae	1	0 ↓	1.59	−1.59	−1	0.22	1	0.0126	0.8419
Bixaceae	1	0 ↓	1.59	−1.59	−1	0.22	1	0.0126	0.8419
Burmanniaceae	1	0 ↓	1.59	−1.59	−1	0.22	1	0.0126	0.8419
Cabombaceae	1	0 ↓	1.59	−1.59	−1	0.22	1	0.0126	0.8419
Caricaceae	1	0 ↓	1.59	−1.59	−1	0.22	1	0.0126	0.8419
Casuarinaceae	1	0 ↓	1.59	−1.59	−1	0.22	1	0.0126	0.8419
Gunneraceae	1	0 ↓	1.59	−1.59	−1	0.22	1	0.0126	0.8419
Kirkiaceae	1	0 ↓	1.59	−1.59	−1	0.22	1	0.0126	0.8419
Lophiocarpaceae	1	0 ↓	1.59	−1.59	−1	0.22	1	0.0126	0.8419
Metteniusaceae	1	0 ↓	1.59	−1.59	−1	0.22	1	0.0126	0.8419
Montiaceae	1	0 ↓	1.59	−1.59	−1	0.22	1	0.0126	0.8419
Montiniaceae	1	0 ↓	1.59	−1.59	−1	0.22	1	0.0126	0.8419
Myristicaceae	1	0 ↓	1.59	−1.59	−1	0.22	1	0.0126	0.8419
Papaveraceae	1	0 ↓	1.59	−1.59	−1	0.22	1	0.0126	0.8419
Petiveriaceae	1	0 ↓	1.59	−1.59	−1	0.22	1	0.0126	0.8419
Zosteraceae	1	0 ↓	1.59	−1.59	−1	0.22	1	0.0126	0.8419
Ericaceae	7	1 ↓	2.65	−1.65	−0.622	1.54	0.8253	0.0319	0.5265
Juncaceae	7	1 ↓	2.65	−1.65	−0.622	1.54	0.8253	0.0319	0.5265
Portulacaceae	19	3 ↓	4.76	−1.76	−0.3694	4.19	0.8238	0.0573	0.3789
Balanophoraceae	2	0 ↓	1.77	−1.77	−1	0.44	1	0.0084	0.7076
Ceratophyllaceae	2	0 ↓	1.77	−1.77	−1	0.44	1	0.0084	0.7076
Costaceae	2	0 ↓	1.77	−1.77	−1	0.44	1	0.0084	0.7076
Droseraceae	2	0 ↓	1.77	−1.77	−1	0.44	1	0.0084	0.7076
Gisekiaceae	2	0 ↓	1.77	−1.77	−1	0.44	1	0.0084	0.7076
Goodeniaceae	2	0 ↓	1.77	−1.77	−1	0.44	1	0.0084	0.7076
Haloragaceae	2	0 ↓	1.77	−1.77	−1	0.44	1	0.0084	0.7076
Limeaceae	2	0 ↓	1.77	−1.77	−1	0.44	1	0.0084	0.7076
Menyanthaceae	2	0 ↓	1.77	−1.77	−1	0.44	1	0.0084	0.7076
Phrymaceae	2	0 ↓	1.77	−1.77	−1	0.44	1	0.0084	0.7076
Pontederiaceae	2	0 ↓	1.77	−1.77	−1	0.44	1	0.0084	0.7076
Tamaricaceae	2	0 ↓	1.77	−1.77	−1	0.44	1	0.0084	0.7076
Amaryllidaceae	14	2 ↓	3.88	−1.88	−0.4842	3.09	0.8486	0.0433	0.4046
Didiereaceae	3	0 ↓	1.94	−1.94	−1	0.66	1	0.0063	0.6024
Gelsemiaceae	3	0 ↓	1.94	−1.94	−1	0.66	1	0.0063	0.6024
Pandanaceae	3	0 ↓	1.94	−1.94	−1	0.66	1	0.0063	0.6024
Podostemaceae	3	0 ↓	1.94	−1.94	−1	0.66	1	0.0063	0.6024
Vahliaceae	3	0 ↓	1.94	−1.94	−1	0.66	1	0.0063	0.6024
Onagraceae	9	1 ↓	3	−2	−0.6664	1.99	0.8939	0.0252	0.445
Caryophyllaceae	21	3 ↓	5.11	−2.11	−0.4128	4.63	0.8733	0.0519	0.3491
Erythroxylaceae	4	0 ↓	2.12	−2.12	−1	0.88	1	0.0051	0.5218
Hypoxidaceae	10	1 ↓	3.17	−2.17	−0.6849	2.21	0.9173	0.0228	0.4128
Polygalaceae	33	5 ↓	7.22	−2.22	−0.3076	7.28	0.8825	0.0676	0.3106
Linaceae	5	0 ↓	2.29	−2.29	−1	1.1	1	0.0042	0.4593
Aponogetonaceae	6	0 ↓	2.47	−2.47	−1	1.32	1	0.0036	0.4096
Begoniaceae	6	0 ↓	2.47	−2.47	−1	1.32	1	0.0036	0.4096
Potamogetonaceae	6	0 ↓	2.47	−2.47	−1	1.32	1	0.0036	0.4096
Xyridaceae	6	0 ↓	2.47	−2.47	−1	1.32	1	0.0036	0.4096
Eriocaulaceae	7	0 ↓	2.65	−2.65	−1	1.54	1	0.0032	0.3694
Violaceae	13	1 ↓	3.7	−2.7	−0.7298	2.87	0.9609	0.0178	0.3387
Gesneriaceae	8	0 ↓	2.82	−2.82	−1	1.77	1	0.0028	0.3363
Commelinaceae	71	11 ↓	13.91	−2.91	−0.2092	15.67	0.9355	0.0892	0.2569
Caprifoliaceae	9	0 ↓	3	−3	−1	1.99	1	0.0025	0.3085
Aizoaceae	10	0 ↓	3.17	−3.17	−1	2.21	1	0.0023	0.2849
Lentibulariaceae	11	0 ↓	3.35	−3.35	−1	2.43	1	0.0021	0.2646
Scrophulariaceae	17	1 ↓	4.41	−3.41	−0.773	3.75	0.9856	0.0138	0.2729
Lythraceae	28	2 ↓	6.34	−4.34	−0.6846	6.18	0.9917	0.0219	0.2277
Hydrocharitaceae	17	0 ↓	4.41	−4.41	−1	3.75	1	0.0014	0.1853
Iridaceae	23	1 ↓	5.46	−4.46	−0.8169	5.07	0.9968	0.0103	0.2112
Plantaginaceae	23	1 ↓	5.46	−4.46	−0.8169	5.07	0.9968	0.0103	0.2112
Campanulaceae	38	3 ↓	8.1	−5.1	−0.6297	8.38	0.9948	0.0287	0.2087
Linderniaceae	28	1 ↓	6.34	−5.34	−0.8423	6.18	0.9991	0.0085	0.1776
Brassicaceae	38	2 ↓	8.1	−6.1	−0.7531	8.38	0.9991	0.0162	0.1732
Gentianaceae	28	0 ↓	6.34	−6.34	−1	6.18	1	0.0009	0.1194
Orobanchaceae	55	3 ↓	11.09	−8.09	−0.7296	12.13	0.9998	0.0198	0.1487
Acanthaceae	271	34 ↓	49.11	−15.11	−0.3077	59.79	1	0.0913	0.1703
Orchidaceae	240	10 ↓	43.65	−33.65	−0.7709	52.95	1	0.023	0.075
Cyperaceae	269	12 ↓	48.76	−36.76	−0.7539	59.35	1	0.0259	0.0763
Poaceae	546	13 ↓	97.51	−84.51	−0.8667	120.47	1	0.0141	0.0403

**Table 3:** Quantitative analysis of food orders. T—total number of species in the flora; F—food species; ↑/↓—positive/negative outliers based on residuals; P_a_—predicted food species under regression model; Res.—regression residuals; P_b_—predicted food species under binomial model; inf.—Bayesian inferior 95% probability credible intervals; sup.—Bayesian superior 95% probability credible intervals; *—positive outliers.

			Regression Analysis			Binomial Analysis		Bayesian Analysis	
Orders	T	F	P_a_	Res.	R-Value	P_b_	*p*-Value	inf. (0.1050)	sup. (0.1212)
Gentianales	533	87 ↑	41.73	45.27	1.0847 *	60.16	0.0003 *	0.1343 *	0.197
Sapindales	158	54 ↑	16.05	37.95	2.3654 *	17.83	0 *	0.2723 *	0.4189
Fabales	592	72 ↑	45.77	26.23	0.5729 *	66.82	0.2681	0.0977	0.1504
Rosales	134	36 ↑	14.4	21.6	1.4997 *	15.12	0 *	0.2009 *	0.3496
Ericales	94	29 ↑	11.66	17.34	1.4868 *	10.61	0 *	0.2242 *	0.4082
Brassicales	116	30 ↑	13.17	16.83	1.2781 *	13.09	0 *	0.1877 *	0.3454
Caryophyllales	182	34 ↑	17.69	16.31	0.922 *	20.54	0.0022 *	0.137 *	0.2498
Malvales	236	37 ↑	21.39	15.61	0.7299 *	26.64	0.025 *	0.116	0.2087
Cucurbitales	76	26 ↑	10.43	15.57	1.4931 *	8.58	0 *	0.2453 *	0.4545
Solanales	166	25 ↑	16.59	8.41	0.5066 *	18.74	0.0826	0.1043	0.213
Malpighiales	418	40 ↑	33.86	6.14	0.1815	47.18	0.8842	0.0711	0.1277
Celastrales	37	13 ↑	7.76	5.24	0.6759 *	4.18	0.0001 *	0.2181 *	0.5135
Boraginales	61	11 ↑	9.4	1.6	0.1701	6.88	0.078	0.1042	0.2953
Magnoliales	32	8 ↑	7.41	0.59	0.0789	3.61	0.023 *	0.133 *	0.4226
Myrtales	100	12 ↓	12.07	−0.07	−0.006	11.29	0.4569	0.0704	0.1983
Zygophyllales	16	6 ↓	6.32	−0.32	−0.0504	1.81	0.0061 *	0.1844 *	0.6167
Arecales	7	5 ↓	5.7	−0.7	−0.1231	0.79	0.0003 *	0.3491 *	0.9148
Vitales	55	8 ↓	8.99	−0.99	−0.1101	6.21	0.278	0.0762	0.2622
Santalales	81	9 ↓	10.77	−1.77	−0.1644	9.14	0.5719	0.0601	0.1982
Nymphaeales	3	2 ↓	5.43	−3.43	−0.6316	0.34	0.0353 *	0.1941 *	0.9324
Ranunculales	34	4 ↓	7.55	−3.55	−0.4703	3.84	0.5444	0.0481	0.2674
Zingiberales	9	2 ↓	5.84	−3.84	−0.6575	1.02	0.27	0.0667	0.5561
Dioscoreales	10	2 ↓	5.91	−3.91	−0.6615	1.13	0.314	0.0602	0.5178
Oxalidales	12	2 ↓	6.04	−4.04	−0.6691	1.35	0.3996	0.0504	0.4545
Aquifoliales	1	1 ↓	5.29	−4.29	−0.811	0.11	0.1129	0.1581	0.9874
Canellales	2	1 ↓	5.36	−4.36	−0.8134	0.23	0.213	0.0943	0.9057
Dilleniales	2	1 ↓	5.36	−4.36	−0.8134	0.23	0.213	0.0943	0.9057
Piperales	17	2 ↓	6.39	−4.39	−0.6869	1.92	0.5871	0.0358	0.3471
Icacinales	3	1 ↓	5.43	−4.43	−0.8158	0.34	0.3018	0.0676	0.8059
Pandanales	5	1 ↓	5.57	−4.57	−0.8203	0.56	0.4505	0.0433	0.6412
Alismatales	58	4 ↓	9.2	−5.2	−0.565	6.55	0.905	0.0281	0.1646
Buxales	1	0 ↓	5.29	−5.29	−1	0.11	1	0.0126	0.8419
Metteniusales	1	0 ↓	5.29	−5.29	−1	0.11	1	0.0126	0.8419
Geraniales	16	1 ↓	6.32	−5.32	−0.8417	1.81	0.8528	0.0146	0.2869
Ceratophyllales	2	0 ↓	5.36	−5.36	−1	0.23	1	0.0084	0.7076
Gunnerales	2	0 ↓	5.36	−5.36	−1	0.23	1	0.0084	0.7076
Vahliales	3	0 ↓	5.43	−5.43	−1	0.34	1	0.0063	0.6024
Fagales	4	0 ↓	5.5	−5.5	−1	0.45	1	0.0051	0.5218
Lamiales	676	46 ↓	51.53	−5.53	−0.1073	76.3	1	0.0515	0.0896
Cornales	5	0 ↓	5.57	−5.57	−1	0.56	1	0.0042	0.4593
Proteales	7	0 ↓	5.7	−5.7	−1	0.79	1	0.0032	0.3694
Laurales	8	0 ↓	5.77	−5.77	−1	0.9	1	0.0028	0.3363
Liliales	8	0 ↓	5.77	−5.77	−1	0.9	1	0.0028	0.3363
Dipsacales	10	0 ↓	5.91	−5.91	−1	1.13	1	0.0023	0.2849
Commelinales	73	4 ↓	10.22	−6.22	−0.6087	8.24	0.9711	0.0223	0.1327
Saxifragales	37	1 ↓	7.76	−6.76	−0.8711	4.18	0.9881	0.0064	0.1381
Apiales	55	2 ↓	8.99	−6.99	−0.7775	6.21	0.989	0.0112	0.1231
Asterales	440	15 ↓	35.36	−20.36	−0.5758	49.66	1	0.0209	0.0555
Asparagales	447	15 ↓	35.84	−20.84	−0.5815	50.45	1	0.0205	0.0546
Poales	838	15 ↓	62.63	−47.63	−0.7605	94.58	1	0.0109	0.0293

**Table 4:** Quantitative analysis of food families. T—total number of species in the flora; F—food species; ↑/↓—positive/negative outliers based on residuals; P_a_—predicted food species under regression model; P_b_—predicted food species under binomial model; inf.—Bayesian inferior 95% probability credible intervals; sup.—Bayesian superior 95% probability credible intervals; *—positive outlier.

			Regression Analysis			Binomial Analysis		Bayesian Analysis	
Family	T	F	P_a_	Res	R-Value	P_b_	*p*-Value	inf. (0.1050)	sup. (0.1212)
Fabaceae	559	71 ↑	42.28	28.72	0.6793 *	63.09	0.1609	0.102	0.1572
Apocynaceae	235	41 ↑	18.5	22.5	1.2166 *	26.52	0.0032 *	0.1314 *	0.2282
Rubiaceae	254	40 ↑	19.89	20.11	1.0109 *	28.67	0.0192 *	0.1179	0.2074
Cucurbitaceae	70	26 ↑	6.39	19.61	3.0715 *	7.9	0 *	0.2676 *	0.489
Malvaceae	221	37 ↑	17.47	19.53	1.118 *	24.94	0.0096 *	0.1241 *	0.2223
Anacardiaceae	25	19 ↑	3.08	15.92	5.163 *	2.82	0 *	0.5635 *	0.8843
Sapotaceae	28	19 ↑	3.3	15.7	4.7522 *	3.16	0 *	0.4917 *	0.8206
Amaranthaceae	64	18 ↑	5.95	12.05	2.0275 *	7.22	0.0002 *	0.186 *	0.4019
Capparaceae	48	16 ↑	4.77	11.23	2.3535 *	5.42	0 *	0.2167 *	0.4754
Burseraceae	42	14 ↑	4.33	9.67	2.2327 *	4.74	0.0001 *	0.2101 *	0.4854
Celastraceae	37	13 ↑	3.96	9.04	2.2798 *	4.18	0.0001 *	0.2181 *	0.5135
Moraceae	55	14 ↑	5.28	8.72	1.6491 *	6.21	0.0026 *	0.1583 *	0.3837
Sapindaceae	36	11 ↑	3.89	7.11	1.8275 *	4.06	0.0015 *	0.1801 *	0.4698
Rhamnaceae	18	8 ↑	2.57	5.43	2.1139 *	2.03	0.0004 *	0.2445 *	0.665
Convolvulaceae	114	15 ↑	9.62	5.38	0.56 *	12.87	0.3042	0.0817	0.206
Ebenaceae	20	8 ↑	2.72	5.28	1.9456 *	2.26	0.0009 *	0.2182 *	0.6156
Boraginaceae	61	11 ↑	5.73	5.27	0.9213 *	6.88	0.078	0.1042	0.2953
Solanaceae	51	10 ↑	4.99	5.01	1.0035 *	5.76	0.0566	0.1106	0.3253
Rutaceae	28	8 ↑	3.3	4.7	1.422 *	3.16	0.0102 *	0.1528 *	0.4724
Cleomaceae	15	7 ↑	2.35	4.65	1.9801 *	1.69	0.0007 *	0.2465 *	0.7012
Annonaceae	30	8 ↑	3.45	4.55	1.3189 *	3.39	0.0157 *	0.1422 *	0.4461
Rosaceae	26	7 ↑	3.16	3.84	1.2178 *	2.93	0.0221 *	0.1375 *	0.4628
Loganiaceae	13	6 ↑	2.2	3.8	1.7247 *	1.47	0.0017 *	0.2304 *	0.7114
Zygophyllaceae	16	6 ↑	2.42	3.58	1.477 *	1.81	0.0061 *	0.1844 *	0.6167
Polygonaceae	20	6 ↑	2.72	3.28	1.2092 *	2.26	0.0197 *	0.1459 *	0.5218
Arecaceae	7	5 ↑	1.76	3.24	1.8382 *	0.79	0.0003 *	0.3491 *	0.9148
Verbenaceae	22	6 ↑	2.86	3.14	1.0959 *	2.48	0.0312 *	0.1321 *	0.4841
Vitaceae	55	8 ↑	5.28	2.72	0.5137 *	6.21	0.278	0.0762	0.2622
Salicaceae	18	5 ↑	2.57	2.43	0.9462 *	2.03	0.0446 *	0.1258 *	0.512
Lamiaceae	198	18 ↑	15.78	2.22	0.1406	22.35	0.8635	0.0585	0.1392
Combretaceae	38	6 ↑	4.04	1.96	0.4862	4.29	0.254	0.0754	0.3053
Urticaceae	29	5 ↑	3.38	1.62	0.4808	3.27	0.2242	0.0771	0.3472
Menispermaceae	16	4 ↑	2.42	1.58	0.6513 *	1.81	0.0977	0.1031	0.499
Olacaceae	3	3 ↑	1.47	1.53	1.0435 *	0.34	0.0014 *	0.3976 *	0.9937
Opiliaceae	3	3 ↑	1.47	1.53	1.0435 *	0.34	0.0014 *	0.3976 *	0.9937
Oleaceae	17	4 ↑	2.5	1.5	0.6028 *	1.92	0.1168	0.0969	0.4764
Salvadoraceae	4	3 ↑	1.54	1.46	0.9462 *	0.45	0.0053 *	0.2836 *	0.9473
Myrtaceae	9	3 ↑	1.91	1.09	0.5719 *	1.02	0.0717	0.1216 *	0.6525
Phyllanthaceae	51	6 ↑	4.99	1.01	0.2021	5.76	0.522	0.0559	0.2344
Pedaliaceae	13	3 ↑	2.2	0.8	0.3623	1.47	0.1744	0.0839	0.508
Chrysobalanaceae	2	2 ↑	1.39	0.61	0.434	0.23	0.0127 *	0.2924 *	0.9916
Nymphaeaceae	2	2 ↑	1.39	0.61	0.434	0.23	0.0127 *	0.2924 *	0.9916
Passifloraceae	30	4 ↑	3.45	0.55	0.1595	3.39	0.4436	0.0545	0.2983
Clusiaceae	3	2 ↑	1.47	0.53	0.3623	0.34	0.0353 *	0.1941 *	0.9324
Talinaceae	4	2 ↑	1.54	0.46	0.2974	0.45	0.0654	0.1466 *	0.8534
Cannabaceae	6	2 ↑	1.69	0.31	0.1846	0.68	0.1404	0.099	0.7096
Moringaceae	6	2 ↑	1.69	0.31	0.1846	0.68	0.1404	0.099	0.7096
Zingiberaceae	8	2 ↑	1.84	0.16	0.0899	0.9	0.2259	0.0749	0.6001
Dioscoreaceae	9	2 ↑	1.91	0.09	0.0479	1.02	0.27	0.0667	0.5561
Hypoxidaceae	10	2 ↑	1.98	0.02	0.0091	1.13	0.314	0.0602	0.5178
Alismataceae	1	1 ↓	1.32	−0.32	−0.2432	0.11	0.1129	0.1581 *	0.9874
Aquifoliaceae	1	1 ↓	1.32	−0.32	−0.2432	0.11	0.1129	0.1581 *	0.9874
Kirkiaceae	1	1 ↓	1.32	−0.32	−0.2432	0.11	0.1129	0.1581 *	0.9874
Basellaceae	2	1 ↓	1.39	−0.39	−0.283	0.23	0.213	0.0943	0.9057
Canellaceae	2	1 ↓	1.39	−0.39	−0.283	0.23	0.213	0.0943	0.9057
Dilleniaceae	2	1 ↓	1.39	−0.39	−0.283	0.23	0.213	0.0943	0.9057
Limeaceae	2	1 ↓	1.39	−0.39	−0.283	0.23	0.213	0.0943	0.9057
Peraceae	2	1 ↓	1.39	−0.39	−0.283	0.23	0.213	0.0943	0.9057
Pittosporaceae	2	1 ↓	1.39	−0.39	−0.283	0.23	0.213	0.0943	0.9057
Asphodelaceae	57	5 ↓	5.43	−0.43	−0.0795	6.43	0.7854	0.0389	0.1898
Didiereaceae	3	1 ↓	1.47	−0.47	−0.3188	0.34	0.3018	0.0676	0.8059
Icacinaceae	3	1 ↓	1.47	−0.47	−0.3188	0.34	0.3018	0.0676	0.8059
Pandanaceae	3	1 ↓	1.47	−0.47	−0.3188	0.34	0.3018	0.0676	0.8059
Primulaceae	17	2 ↓	2.5	−0.5	−0.1986	1.92	0.5871	0.0358	0.3471
Ochnaceae	18	2 ↓	2.57	−0.57	−0.2215	2.03	0.6189	0.0338	0.3314
Achariaceae	5	1 ↓	1.61	−0.61	−0.3808	0.56	0.4505	0.0433	0.6412
Aristolochiaceae	5	1 ↓	1.61	−0.61	−0.3808	0.56	0.4505	0.0433	0.6412
Putranjivaceae	5	1 ↓	1.61	−0.61	−0.3808	0.56	0.4505	0.0433	0.6412
Portulacaceae	19	2 ↓	2.64	−0.64	−0.2431	2.14	0.6489	0.0321	0.317
Bignoniaceae	6	1 ↓	1.69	−0.69	−0.4077	0.68	0.5125	0.0367	0.5787
Connaraceae	6	1 ↓	1.69	−0.69	−0.4077	0.68	0.5125	0.0367	0.5787
Oxalidaceae	6	1 ↓	1.69	−0.69	−0.4077	0.68	0.5125	0.0367	0.5787
Dichapetalaceae	9	1 ↓	1.91	−0.91	−0.476	1.02	0.6597	0.0252	0.445
Onagraceae	9	1 ↓	1.91	−0.91	−0.476	1.02	0.6597	0.0252	0.445
Aizoaceae	10	1 ↓	1.98	−0.98	−0.4954	1.13	0.6981	0.0228	0.4128
Malpighiaceae	10	1 ↓	1.98	−0.98	−0.4954	1.13	0.6981	0.0228	0.4128
Hypericaceae	12	1 ↓	2.13	−1.13	−0.5302	1.35	0.7624	0.0192	0.3603
Nyctaginaceae	12	1 ↓	2.13	−1.13	−0.5302	1.35	0.7624	0.0192	0.3603
Piperaceae	12	1 ↓	2.13	−1.13	−0.5302	1.35	0.7624	0.0192	0.3603
Araceae	26	2 ↓	3.16	−1.16	−0.3663	2.93	0.8086	0.0235	0.2429
Amaryllidaceae	14	1 ↓	2.28	−1.28	−0.5605	1.58	0.813	0.0166	0.3195
Lythraceae	28	2 ↓	3.3	−1.3	−0.3945	3.16	0.8405	0.0219	0.2277
Adoxaceae	1	0 ↓	1.32	−1.32	−1	0.11	1	0.0126	0.8419
Alismataceae	1	0 ↓	1.32	−1.32	−1	0.11	1	0.0126	0.8419
Ancistrocladaceae	1	0 ↓	1.32	−1.32	−1	0.11	1	0.0126	0.8419
Aphloiaceae	1	0 ↓	1.32	−1.32	−1	0.11	1	0.0126	0.8419
Berberidaceae	1	0 ↓	1.32	−1.32	−1	0.11	1	0.0126	0.8419
Bixaceae	1	0 ↓	1.32	−1.32	−1	0.11	1	0.0126	0.8419
Burmanniaceae	1	0 ↓	1.32	−1.32	−1	0.11	1	0.0126	0.8419
Buxaceae	1	0 ↓	1.32	−1.32	−1	0.11	1	0.0126	0.8419
Cabombaceae	1	0 ↓	1.32	−1.32	−1	0.11	1	0.0126	0.8419
Cactaceae	1	0 ↓	1.32	−1.32	−1	0.11	1	0.0126	0.8419
Caricaceae	1	0 ↓	1.32	−1.32	−1	0.11	1	0.0126	0.8419
Casuarinaceae	1	0 ↓	1.32	−1.32	−1	0.11	1	0.0126	0.8419
Flagellariaceae	1	0 ↓	1.32	−1.32	−1	0.11	1	0.0126	0.8419
Francoaceae	1	0 ↓	1.32	−1.32	−1	0.11	1	0.0126	0.8419
Gunneraceae	1	0 ↓	1.32	−1.32	−1	0.11	1	0.0126	0.8419
Hamamelidaceae	1	0 ↓	1.32	−1.32	−1	0.11	1	0.0126	0.8419
Lecythidaceae	1	0 ↓	1.32	−1.32	−1	0.11	1	0.0126	0.8419
Liliaceae	1	0 ↓	1.32	−1.32	−1	0.11	1	0.0126	0.8419
Lophiocarpaceae	1	0 ↓	1.32	−1.32	−1	0.11	1	0.0126	0.8419
Metteniusaceae	1	0 ↓	1.32	−1.32	−1	0.11	1	0.0126	0.8419
Monimiaceae	1	0 ↓	1.32	−1.32	−1	0.11	1	0.0126	0.8419
Montiaceae	1	0 ↓	1.32	−1.32	−1	0.11	1	0.0126	0.8419
Montiniaceae	1	0 ↓	1.32	−1.32	−1	0.11	1	0.0126	0.8419
Musaceae	1	0 ↓	1.32	−1.32	−1	0.11	1	0.0126	0.8419
Myristicaceae	1	0 ↓	1.32	−1.32	−1	0.11	1	0.0126	0.8419
Myrothamnaceae	1	0 ↓	1.32	−1.32	−1	0.11	1	0.0126	0.8419
Papaveraceae	1	0 ↓	1.32	−1.32	−1	0.11	1	0.0126	0.8419
Penaeaceae	1	0 ↓	1.32	−1.32	−1	0.11	1	0.0126	0.8419
Petiveriaceae	1	0 ↓	1.32	−1.32	−1	0.11	1	0.0126	0.8419
Zosteraceae	1	0 ↓	1.32	−1.32	−1	0.11	1	0.0126	0.8419
Geraniaceae	15	1 ↓	2.35	−1.35	−0.5743	1.69	0.8341	0.0155	0.3023
Balanophoraceae	2	0 ↓	1.39	−1.39	−1	0.23	1	0.0084	0.7076
Ceratophyllaceae	2	0 ↓	1.39	−1.39	−1	0.23	1	0.0084	0.7076
Costaceae	2	0 ↓	1.39	−1.39	−1	0.23	1	0.0084	0.7076
Droseraceae	2	0 ↓	1.39	−1.39	−1	0.23	1	0.0084	0.7076
Gisekiaceae	2	0 ↓	1.39	−1.39	−1	0.23	1	0.0084	0.7076
Goodeniaceae	2	0 ↓	1.39	−1.39	−1	0.23	1	0.0084	0.7076
Haloragaceae	2	0 ↓	1.39	−1.39	−1	0.23	1	0.0084	0.7076
Menyanthaceae	2	0 ↓	1.39	−1.39	−1	0.23	1	0.0084	0.7076
Phrymaceae	2	0 ↓	1.39	−1.39	−1	0.23	1	0.0084	0.7076
Phytolaccaceae	2	0 ↓	1.39	−1.39	−1	0.23	1	0.0084	0.7076
Picrodendraceae	2	0 ↓	1.39	−1.39	−1	0.23	1	0.0084	0.7076
Pontederiaceae	2	0 ↓	1.39	−1.39	−1	0.23	1	0.0084	0.7076
Smilacaceae	2	0 ↓	1.39	−1.39	−1	0.23	1	0.0084	0.7076
Tamaricaceae	2	0 ↓	1.39	−1.39	−1	0.23	1	0.0084	0.7076
Typhaceae	2	0 ↓	1.39	−1.39	−1	0.23	1	0.0084	0.7076
Velloziaceae	2	0 ↓	1.39	−1.39	−1	0.23	1	0.0084	0.7076
Cornaceae	3	0 ↓	1.47	−1.47	−1	0.34	1	0.0063	0.6024
Gelsemiaceae	3	0 ↓	1.47	−1.47	−1	0.34	1	0.0063	0.6024
Lauraceae	3	0 ↓	1.47	−1.47	−1	0.34	1	0.0063	0.6024
Myricaceae	3	0 ↓	1.47	−1.47	−1	0.34	1	0.0063	0.6024
Podostemaceae	3	0 ↓	1.47	−1.47	−1	0.34	1	0.0063	0.6024
Simaroubaceae	3	0 ↓	1.47	−1.47	−1	0.34	1	0.0063	0.6024
Vahliaceae	3	0 ↓	1.47	−1.47	−1	0.34	1	0.0063	0.6024
Hydrocharitaceae	17	1 ↓	2.5	−1.5	−0.5993	1.92	0.8694	0.0138	0.2729
Erythroxylaceae	4	0 ↓	1.54	−1.54	−1	0.45	1	0.0051	0.5218
Hernandiaceae	4	0 ↓	1.54	−1.54	−1	0.45	1	0.0051	0.5218
Resedaceae	4	0 ↓	1.54	−1.54	−1	0.45	1	0.0051	0.5218
Colchicaceae	5	0 ↓	1.61	−1.61	−1	0.56	1	0.0042	0.4593
Linaceae	5	0 ↓	1.61	−1.61	−1	0.56	1	0.0042	0.4593
Stilbaceae	5	0 ↓	1.61	−1.61	−1	0.56	1	0.0042	0.4593
Aponogetonaceae	6	0 ↓	1.69	−1.69	−1	0.68	1	0.0036	0.4096
Begoniaceae	6	0 ↓	1.69	−1.69	−1	0.68	1	0.0036	0.4096
Molluginaceae	6	0 ↓	1.69	−1.69	−1	0.68	1	0.0036	0.4096
Plumbaginaceae	6	0 ↓	1.69	−1.69	−1	0.68	1	0.0036	0.4096
Potamogetonaceae	6	0 ↓	1.69	−1.69	−1	0.68	1	0.0036	0.4096
Xyridaceae	6	0 ↓	1.69	−1.69	−1	0.68	1	0.0036	0.4096
Ericaceae	7	0 ↓	1.76	−1.76	−1	0.79	1	0.0032	0.3694
Eriocaulaceae	7	0 ↓	1.76	−1.76	−1	0.79	1	0.0032	0.3694
Juncaceae	7	0 ↓	1.76	−1.76	−1	0.79	1	0.0032	0.3694
Proteaceae	7	0 ↓	1.76	−1.76	−1	0.79	1	0.0032	0.3694
Caryophyllaceae	21	1 ↓	2.79	−1.79	−0.6415	2.37	0.9191	0.0112	0.2284
Asparagaceae	103	7 ↓	8.81	−1.81	−0.2053	11.63	0.9534	0.0338	0.1338
Gesneriaceae	8	0 ↓	1.84	−1.84	−1	0.9	1	0.0028	0.3363
Caprifoliaceae	9	0 ↓	1.91	−1.91	−1	1.02	1	0.0025	0.3085
Rhizophoraceae	9	0 ↓	1.91	−1.91	−1	1.02	1	0.0025	0.3085
Meliaceae	23	1 ↓	2.94	−1.94	−0.6594	2.6	0.9364	0.0103	0.2112
Santalaceae	23	1 ↓	2.94	−1.94	−0.6594	2.6	0.9364	0.0103	0.2112
Brassicaceae	38	2 ↓	4.04	−2.04	−0.5046	4.29	0.9384	0.0162	0.1732
Campanulaceae	38	2 ↓	4.04	−2.04	−0.5046	4.29	0.9384	0.0162	0.1732
Lentibulariaceae	11	0 ↓	2.06	−2.06	−1	1.24	1	0.0021	0.2646
Araliaceae	12	0 ↓	2.13	−2.13	−1	1.35	1	0.0019	0.2471
Violaceae	13	0 ↓	2.2	−2.2	−1	1.47	1	0.0018	0.2316
Thymelaeaceae	14	0 ↓	2.28	−2.28	−1	1.58	1	0.0017	0.218
Melastomataceae	15	0 ↓	2.35	−2.35	−1	1.69	1	0.0016	0.2059
Ranunculaceae	16	0 ↓	2.42	−2.42	−1	1.81	1	0.0015	0.1951
Commelinaceae	71	4 ↓	6.46	−2.46	−0.3807	8.01	0.9659	0.0229	0.1362
Scrophulariaceae	17	0 ↓	2.5	−2.5	−1	1.92	1	0.0014	0.1853
Polygalaceae	33	1 ↓	3.67	−2.67	−0.7275	3.72	0.9808	0.0072	0.1533
Crassulaceae	34	1 ↓	3.74	−2.74	−0.7329	3.84	0.983	0.007	0.1492
Balsaminaceae	21	0 ↓	2.79	−2.79	−1	2.37	1	0.0012	0.1544
Loranthaceae	50	2 ↓	4.92	−2.92	−0.5933	5.64	0.9815	0.0123	0.1346
Iridaceae	23	0 ↓	2.94	−2.94	−1	2.6	1	0.0011	0.1425
Plantaginaceae	23	0 ↓	2.94	−2.94	−1	2.6	1	0.0011	0.1425
Apiaceae	41	1 ↓	4.26	−3.26	−0.7651	4.63	0.9926	0.0058	0.1257
Orobanchaceae	55	2 ↓	5.28	−3.28	−0.6216	6.21	0.989	0.0112	0.1231
Gentianaceae	28	0 ↓	3.3	−3.3	−1	3.16	1	0.0009	0.1194
Linderniaceae	28	0 ↓	3.3	−3.3	−1	3.16	1	0.0009	0.1194
Euphorbiaceae	217	13 ↓	17.18	−4.18	−0.2431	24.49	0.9973	0.0356	0.0998
Acanthaceae	271	12 ↓	21.14	−9.14	−0.4323	30.59	1	0.0257	0.0758
Cyperaceae	269	5 ↓	20.99	−15.99	−0.7618	30.36	1	0.0082	0.0427
Asteraceae	398	13 ↓	30.46	−17.46	−0.5732	44.92	1	0.0193	0.0551
Orchidaceae	240	0 ↓	18.86	−18.86	−1	27.09	1	0.0001	0.0152
Poaceae	546	10 ↓	41.32	−31.32	−0.758	61.63	1	0.0101	0.0334

## Data Availability

The data used to support the findings of the manuscript is contained in Appendix A and Appendix B.

## Appendix A

Checklist of medicinal plants of Kenya, introduced species are indicated in brackets as ‘exotic.’

**Species/Family**	**Habit**	**Parts**	**Diseases Treated**	**References**
Acanthaceae				
*Acanthus eminens* C.B.Clarke	Shrub	Leaf, whole plant, roots	Chest/joint pains, fever, abdominal cramps/pains, spleen/liver problems, alimentary canal problems, hypertension, stomachache, typhoid, amoebiasis, headache, heart-problem, skin diseases, wounds, eye infections	[78,88,109,119,120,132,141,143,152]
*Acanthus polystachyus* Delile	Shrub	Leaf, root, stem	Mental disturbance, acaricide, cough, spleen/liver problems, fever	[98,120,133,144]
*Anisotes ukambensis* Lindau	Shrub	Bark	Bleeding during pregnancy	[74]
*Asystasia mysorensis* (Roth) T.Anderson	Herb	Leaf	*Chira*, false teeth, colds, headache, malaria, ringworms, cough, skin diseases	[76,78,99,143]
*Barleria acanthoides* Vahl	Shrub	Roots, leaves	Tonic, eye lotion	[77]
*Barleria delamerei* S.Moore	Herb	Whole plant, roots, leaf	Polio, fever, tonic, eye lotion	[72,77]
*Barleria eranthemoides* R.Br. ex C.B.Clarke	Shrub	Leaf, root, whole plant	Dyspepsia, vomiting, postpartum hemorrhage, diaphragm, foreign objects pierced into the skin, boils	[51,81,102,148]
*Barleria grandicalyx* Lindau	Herb	Leaf	Snakebites	[78]
*Barleria quadrispina* Lindau	Shrub	Roots	Wasting sickness in goats, menstrual disorders	[127,142]
*Barleria ventricosa* Hochst. ex Nees	Herb	Whole plant	Rheumatism	[106]
*Blepharis edulis* (Forssk.) Pers.	Herb	Whole plant	Lung problems, malaria, hepatitis, TB, chest pain	[72,77,92]
*Blepharis maderaspatensis* (L.) B.Heyne ex Roth	Herb	Whole plant	Sores	[140]
*Crabbea velutina* S.Moore	Herb	Root, leaf, whole plant	Scabies, prophylaxis for baby ailments, enlarged glands	[76,140,141]
*Crossandra massaica* Mildbr.	Herb	Leaf	Snakebites (humans/cattle), syphilis	[67,76]
*Dicliptera laxata* C.B.Clarke	Herb	Roots, leaf, aerial parts	Colorectal cancer, diarrhea, skin rashes, chest pain, difficult breathing, wounds, mouth ulcers in children, cough, eye/ear infection, boils, snakebites	[117,119,122,144]
*Duosperma kilimandscharicum* (Lindau) Dayton	Shrub	Leaf	Cuts, sores, wounds	[80]
*Dyschoriste radicans* Nees	Herb	leaf	Itchy skin, HIV/AIDs, fever, STIs, orchitis, skin diseases, wounds, eye infections	[78,119,143,144]
*Dyschoriste thunbergiiflora* (S.Moore) Lindau	Herb/shrub	Leaf	Skin diseases, wounds, eye infections	[78]
*Hygrophila auriculata* (Schumach.) Heine	Herb	Whole plant	Corneal ulcers	[143]
*Hypoestes aristata* (Vahl) Sol. ex Roem. & Schult.	Herb	Leaves, roots, stems	Wounds, stomachache, colds, malaria, liver problems, stomach ulcers, nausea, joint/chest pain, TB, chest complaints, pneumonia, sore throat, cough, eye diseases, abdominal pains	[67,87,109,115,131,141,152]
*Hypoestes forskaolii* (Vahl) R.Br.	Herb	Root, bark, leaves, aerial parts	Stomachache, colds, malaria, liver problems, stomach ulcers, nausea, pesticide	[91,104,132,152,168]
*Justicia anagalloides* (Nees) T.Anderson	Herb	Leaves	Edema	[144]
*Justicia calyculata* Deflers	Herb	Aerial parts	Snakebites	[126]
*Justicia exigua* S.Moore	Herb	Whole plant	Gastrointestinal problems	[79]
*Justicia flava* (Forssk.) Vahl	Herb	Leaves, roots	Postnatal pains, eyes problems, cough, boils, diarrhea, stomachache, vomiting, ulcers, pneumonia	[67,78,122,125,132]
*Justicia odora* (Forssk.) Lam.	Shrub	Aerial parts, bark	Aphrodisiac	[80]
*Justicia striata* (Klotzsch) Bullock	Herb	Leaves	Skin sores/ulcers	[141]
*Lepidagathis scariosa* Nees	Herb	Leaves	Diarrhea, wounds, edema, foot and mouth in livestock, pneumonia	[78]
*Monechma subsessile* (Oliv.) C.B.Clarke	Herb	Roots	Labor pains	[144]
*Nicoteba betonica* (L.) Lindau	Herb	Leaves, flowers, roots, whole plant, aerial parts	Malaria, cough, breast/colorectal/skin cancer, toothaches, diarrhea, stomachache, STIs, edema, orchitis	[67,78,79,101,117,119,122,143,144]
*Ruellia patula* Jacq.	Herb	Root	Joint diseases	[149]
*Thunbergia alata* Bojer ex Sims	Herb	Leaves, buds	Joint dislocation (humans, livestock), boils, toothache, body swellings, watery painful eyes, wounds, internal injury, bone fractures, stomachache, mouth ulcers, cellulitis, cough, suppurating ears, diarrhea, skin diseases, goat/sheep diseases, backache, hydrocele, joint pains, fungal diseases, tongue thrush, fetus placement in the womb, hardening of soft parts of the central part of the head in babies, gall sickness in cattle	[78,86,120,122,125,141,143,144,168]
*Thunbergia fischeri* Engl.	Herb	Whole plant	Purgative	[67]
*Thunbergia holstii* Lindau	Shrub	Leaves	Malaria	[138]
**Achariaceae**				
*Grandidiera boivinii* Jaub.	Shrub	Roots, leaves	Common cold, tonsils, septic swelling, poison antidote	[127]
**Amaranthaceae**				
*Achyranthes aspera* L.	Herb/shrub	Roots, whole plant, leaves	Skin diseases, wounds/cuts, teeth problems, back/knee aches, conjunctivitis, malaria, venereal diseases, septic swelling, urinary tract problems, constipation, STIs, back/joint/bone problems, arthritis, general body, stomachache, anthrax, toothache, cough, excess menstrual discharge, headache, gonorrhea, kidney problems, diabetes, kidney pains	[41,51,76,78,84,89,90,92,96,115,120,127,132,136,138,143,144,147,149,152,168]
*Achyropsis fruticulosa* C.B.Clarke	Shrub	Root	Malaria	[77]
*Aerva javanica* (Burm.f.) Juss. ex Schult.	Hern/shrub	Roots, flowers	ECF in cattle, retained placenta	[67,89,139]
*Alternanthera pungens* Kunth (exotic)	Herb	Leaves, roots	Toothache, mouth sores	[106,122]
*Alternanthera sessilis* (L.) R.Br. ex DC.	Herb	Leaves	Eye infections, wounds, skin diseases	[119]
*Amaranthus graecizans* L.	Herb	Leaves, whole plant	AIDS, amoebiasis, cough, colds/flu, diabetes, energy boost, high blood pressure, immunity booster, increase blood, poor digestion, boils, rheumatism, anthelmintic, wounds, prevent abortion, cancer	[41,78,81,147]
*Amaranthus hybridus* L.	Herb	Leaves, roots, whole plant	Colic pain in infants, mineral/vitamin deficiency in pregnancy, urinary tract infection, kidney and stomach ailments, bilharzia, stomach acid, anthelmintic in human/animals, measles, malaria	[84,88,96,111,147,153,168]
*Amaranthus spinosus* L.	Herb	Roots, whole plant	Mouth infection, venom antidote, wounds, fever	[76,132,144]
*Amaranthus tricolor* L. (exotic)	Herb	Leaves	Ear diseases, skin disease	[141,144]
*Beta vulgaris* L. (exotic)	Herb		Cancer	[41]
*Celosia anthelminthica* Asch.	Herb	Whole plant	Anthelmintic	[143]
*Celosia argentea* L.	Herb	Leaves, flowers, stems	Convalescents	[67]
*Celosia schweinfurthiana* Schinz (exotic)	Herb	Whole plant	Malaria, anthelmintic	[124,143]
*Celosia trigyna* L.	Herb	Whole plant	Anthelmintic	[143]
*Chenopodium album* L. (exotic)	Herb	Whole plant	Abdominal upsets in infants, blood detoxifier in pregnancy	[88]
*Chenopodium opulifolium* Schrad. ex W.D.J.Koch & Ziz	Herb	Leaves, roots	Diarrhea, fever in babies, cough, anaplasmosis in cattle, roundworms in children, suppurating ears, urinary disorders	[119,132,168]
*Cyathula cylindrica* Moq.	Herb	Roots, bark, leaves	Malaria, stomach problems, wounds, joint pains, emetic, purgative, diarrhea, fungal infections	[67,78,104,109]
*Cyathula polycephala* Baker	Herb	Roots, leaves, bark, whole plant	Dental problems, malaria, emetic, ulcers, breast cancer, boils, urinary problems, diabetes, vomiting, diarrhea, heart problems, stomachache, anthelmintic	[41,67,84,104,115,138,144,147,168]
*Cyathula uncinulata* (Schrad.) Schinz	Herb/shrub	Roots, leaves	Malaria, wounds, induce pregnancy, skin rashes, abdominal pains, emetic	[99,141,144,152]
*Digera muricata* (L.) Mart.	Herb	Leaves	Generally medicinal	[91]
*Dysphania ambrosioides* (L.) Mosyakin & Clemants	Herb	Leaves, whole plant	Neck swelling in calves, wounds, anthelmintic, boils	[67,115,132,140]
*Gomphrena celosioides* Mart. (exotic)	Herb	Roots	Wound, burns	[77]
*Ouret lanata* (L.) Kuntze	Herb/shrub	Leaves	Sores eyes	[67]
*Psilotrichum elliotii* Baker	Shrub	Whole plant	Boils	[147]
*Psilotrichum scleranthum* Thwaites	Herb/shrub	Roots, leaves	Convulsions, aphrodisiac, smoothening skin	[127,150]
*Pupalia lappacea* (L.) Juss.	Herb	Whole plant, roots	Venereal diseases, induce salivation in babies	[127]
*Sericocomopsis hildebrandtii* Schinz	Shrub	Root	Malaria, stomachache, weight during pregnancy, induce bile release from the gallbladder, hepatitis, purgative	[77,92,97,105,136]
*Sericocomopsis pallida* (S.Moore) Schinz	Shrub	Root	Malaria	[72]
**Amaryllidaceae**				
*Allium ampeloprasum* L. (exotic)	Herb	Leaves	Colds, flu, cleaning mouth, earache, cough	[84,108,147]
*Allium cepa* L. (exotic)	Herb	Roots	Cough, flu, headache, pneumonia, bloat (cows), chest problem, digestive disorders, rheumatism, wounds, colds (human/cattle)	[14,41,73,74,168]
*Allium sativum* L. (exotic)	Herb	Roots, fruits	Malaria, HIV/AIDs, toothache, diabetes mellitus, hypertension, respiratory diseases, stomachache, mosquito repellant, allergies, colds, runny nose	[14,83,85,98,108,129,137,146]
*Ammocharis tinneana* (Kotschy & Peyr.) Milne-Redh. & Schweick.	Herb	Root, exudate	Snakebites, emetic	[126,143]
*Crinum macowanii* Baker	Herb	Root, exudate	Urinary tract infections, chest pains, boils, snakebites, livestock diseases	[80,143]
**Anacardiaceae**				
*Anacardium occidentale* L. (exotic)	Shrub/tree	Leaves, roots	Toothache, child colic	[150]
*Lannea alata* (Engl.) Engl.	Shrub/tree	Roots, leaves	Burns, wounds, stomach problems	[72,80]
*Lannea fulva* (Engl.) Engl.	Shrub/tree	Roots, bark	Gut disorders, diarrhea, chest pains, cough, stomachache, antivenom, headache, astringent, expectorant, colds	[91,99,116,132,141,142]
*Lannea rivae* (Chiov.) Sacleux	Shrub/tree	Bark	Ear problems, nose and throat problems, diarrhea	[41,77]
*Lannea schimperi* (Hochst. ex A.Rich.) Engl.	Tree	Bark, roots, leaves	Aphrodisiac, gut problems, diarrhea, dysentery, acaricide, prophylaxis for baby/children’s ailments, stomachache, chest problems	[41,78,116,133,140]
*Lannea schweinfurthii* (Engl.) Engl.	Shrub/tree	Bark, roots, leaves	Malaria, venereal diseases, induce labor pains, amoeba, stomachache, boils, skin rashes, cough, colds/flu, STIs, urinary system infections, pneumonia, gastrointestinal problems, pregnancy anemia, vomiting, headache, waterborne diseases, arthritis, increase blood, barrenness, boils, skin diseases, toothache, heart problem, dermatological problems, jaundice, anthrax, gynecological problems, dysentery, infertility, teeth removal, anti-acid, laxative, backache, gonorrhea	[14,41,51,75,76,79,81,100,102,104,106,108,127,132,138,142,143,145,148,150,151]
*Lannea triphylla* (Hochst. ex A.Rich.) Engl.	Shrub/tree	Roots, bark	Diarrhea, liver diseases, dysentery, rheumatism, body swelling, malaria, colds, stomachache, chest problems, nausea in pregnancy	[14,77,80,99,139]
*Lannea welwitschii* (Hiern) Engl.	Tree		Diarrhea, hemorrhoids, infertility in women, menstrual troubles, gonorrhea, epilepsy, skin infections, ulcers	[110]
*Mangifera indica* L. (exotic)	Tree	Roots, leaves, bark, fruits	TB, malaise, allergies, amoebiasis, back/joint/bone problems, burns, cough, colds/flu, diabetes, goiter, hypertension, injuries/cuts, wounds, kwashiorkor, malaria, pneumonia, rheumatism, typhoid, toothache, ringworms, diarrhea, gonorrhea, stomachache, ear infections, chest pains, toothache, skin/throat/breast cancer, constipation, STIs, diabetes, yellow fever, diarrhea, abdominal pains	[14,41,80,83,84,106,116,117,118,119,129,139,143,146,150,168]
*Ozoroa insignis* Delile	Shrub/tree	Bark, root, stems, leaves	Mumps, toothache, snakebites, complications in menstruation, venereal diseases, diarrhea, clean womb, liver problems, rib pain, tonic, stomachache, kidney diseases, acaricide, retained placenta in human/animals, constipation in animals, gastrointestinal problems, enlarged spleen, headache, cancer, vomiting, heart problem, pregnancy pains, astringent, dysmenorrhea, malaria	[67,77,79,86,87,91,99,105,107,111,112,127,132,133,140,141,142,143,145]
*Ozoroa obovata* (Oliv.) R.Fern. & A.Fern.	Shrub/tree	Roots	Menstrual disorders, venereal diseases, stomachache, malaria, dizziness	[127,142,150]
*Pistacia aethiopica* Kokwaro	Shrub/tree	Roots, bark	Allergies, cough, colds/flu, dental problems, diabetes, malaria, spleen pain and enlargement, dizziness, liver problems, fever, pneumonia, rheumatism	[41,84,140,168]
*Schinus molle* L. (exotic)	Tree	Roots, leaves, stems	Cough, colds/flu, malaria, rheumatism, chicken pox	[41,71,107,168]
*Sclerocarya birrea* (A.Rich.) Hochst.	Tree	Bark, roots, fruits	Colds, flu, edema of limbs, respiratory disorders, joint pains, toothache, joint aches, backache, body aches, malaria, stomachache, constipation, dysentery, diarrhea, fever, ulcers, enlarged spleen, heartburn, dysentery, liver problems, rheumatism, diabetes, tick control, abdominal pain, spleen diseases, STI’s, cough	[14,41,64,72,90,91,99,100,104,112,127,141,142,143,147]
*Searsia longipes* (Engl.) Moffett	Shrub/tree	Fruits, roots	Amoeba, anthelmintic, backache, stomachache, headache, influenza, diarrhea	[14,99,147]
*Searsia natalensis* (Bernh. ex C.Krauss) F.A.Barkley	Shrub/tree	Leaves, bark, seeds, roots, stems,	Abdominal pains, gonorrhea, anthelmintic in human/livestock, malaria, tonic, respiratory disorders, stomachic, endometritis, foot/mouth disease, postpartum pain, stomach problems, diarrhea, teeth cleaning, stomachache, bone pains, premenstrual pains, madness, weak bone, joint pains, abdominal pains, urinary tract infection, convulsions, constipation, spinal cord problem, liver and spleen diseases, peptic ulcers, invigorant, helps in child delivery, back/bone joint problems, cough, colds, flu, ear-nose-throat problems, lack of appetite, low libido, back/joint/bone problems, arthritis, wounds, dental diseases, livestock weakness, giardia, headache, neck pain, fever, TB, vomiting, eye lotion, stomach/skin/breast cancer, influenza, back pain, anthrax, postpartum pains, toothache, tonsillitis, boils, acaricide, abdominal pain, heartburn, rashes, liver enlargement, asthma, wounds, prophylaxis for diseases various diseases, general body weakness, colic pain, pain in pregnancy, strengthen bones, vitamin supplements, bilharzia, afterbirth pains, indigestion, chest problems, pneumonia, respiratory disorders, abdominal pains, venereal diseases, sore feet, purgative	[14,41,66,68,71,72,75,76,77,78,79,81,82,84,86,88,90,98,99,100,101,104,105,106,107,108,109,112,113,115,117,119,120,123,125,127,132,133,135,136,138,140,141,142,143,144,148,152,154,168]
**Annonaceae**				
*Annona senegalensis* Pers.	Shrub/tree	Leaves, bark, roots	Eye problems, snakebites, stomach upsets, acaricide stomachache, poison antidote, intestinal pains, vomiting, diarrhea, constipation, sores, ulcers, boils, liver/spleen problems, headache, dizziness, colds	[95,112,126,133,134,142,143,144,150]
*Annona squamosa* L. (exotic)	Shrub/tree	Roots, leaves, fruits	Nutrient supplement, ulcers, blood purifier, hypertension, reproductive disorders, dizziness	[110,150]
*Asteranthe asterias* (S.Moore) Engl. & Diels	Shrub/tree	Roots, leaves	Sore throats, coughs, venereal diseases	[127]
*Huberantha stuhlmannii* (Engl.) Chaowasku	Shrub	Roots, bark	Hypertension, typhoid, obesity	[150]
*Monanthotaxis fornicata* (Baill.) Verdc.	Shrub	Roots, leaves	General body pains, prenatal care, menstruation complications	[127]
*Monanthotaxis schweinfurthii* (Engl. & Diels) Verdc.	Liana	Roots	Malaria, rheumatism	[41,129]
*Monanthotaxis trichocarpa* (Engl. & Diels) Verdc.	Liana	Leaves	Headache	[142]
*Monodora grandidieri* Baill.	Shrub/tree	Roots, leaves	Rectal problems, anthelmintic, aphrodisiac, gastrointestinal gas, reproductive disorders	[127,134,150]
*Uvaria acuminata* Oliv.	Shrub/tree	Roots, leaves	Convulsions, snakebites, dysentery, cough, stomach upsets, spleen problems, laxative, gastrointestinal gas, aphrodisiac, rectal prolapse, menstrual cramps, malaria, female reproductive disorders	[75,100,127,142,145,150]
*Uvaria faulknerae* Verdc.	Liana	Roots, leaves	Convulsions	[127]
*Uvaria leptocladon* Oliv.	Shrub/tree/liana	Root	Diuretic, abortifacient	[142]
*Uvaria lucida* Bojer ex Benth.	Shrub/liana	Roots, leaves	Convulsions, reproductive disorders, aphrodisiac	[127,150]
*Uvaria scheffleri* Diels	Tree/liana	Bark, root	Malaria, joint/chest pains, cough/colds/flu, lack of appetite, painkiller, stomach disorders, debility, pneumonia, swollen legs, stomach pain in pregnancy, retained placenta	[41,77,84,91,96,99,129,139,141,142]
*Uvariodendron anisatum* Verdc.	Shrub/tree	Roots, leaves	Allergies, amoebiasis, arthritis, cough, colds, flu, diabetes, HIV/AIDs, infertility, injuries, cuts, wounds, retained placenta in cows/human, low libido, rheumatism, STIs, ulcers, scorpion/snake bites, breast/prostate cancer, calf rejection, erectile dysfunction/impotence, malaria, labor pains, aphrodisiac	[14,41,84,146,168]
*Uvariodendron kirkii* Verdc.	Shrub/tree	Roots	Convulsions, bloody diarrhea	[127]
*Xylopia parviflora* Spruce	Tree	Roots	Aphrodisiac	[150]
**Apiaceae**				
*Afroligusticum aculeolatum* (Engl.) P.J.D.Winter	Herb	Roots, leaves	Cancer, purgative, colds	[129,141]
*Alepidea peduncularis* Steud. ex A.Rich.	Herb	Bark	Chest problems, bronchitis	[144]
*Anthriscus sylvestris* (L.) Hoffm.	Herb	Seeds	Colds	[67,89]
*Centella asiatica* (L.) Urb.	Herb	Leaves, roots, stems, whole plant	Loss of memory, toothache, ear infections, boils, mouth ulcers, infertility, measles, stomachache, HIV/AIDs	[119,122,143,144,154]
*Coriandrum sativum* L. (exotic)	Herb	Leaves	Colds, flu, digestive disorders, nausea, dysentery	[84,168]
*Daucus carota* L.	Herb	Roots	Heartburns, stomach disorders	[41]
*Daucus incognitus* (C.Norman) Spalik, Reduron & Banasiak	Herb	Roots	Pneumonia	[120]
*Diplolophium africanum* Turcz.	Herb	Root	Ailments in children/babies	[140]
*Ferula communis* L.	Herb	Roots	General sickness in babies/children	[140]
*Foeniculum vulgare* Mill. (exotic)	Herb		Colic, Vitamin A supplement	[41]
*Heteromorpha trifoliata* (H.L.Wendl.) Eckl. & Zeyh.	Shrub/tree	Roots, levees	Stomachache, skin infections, measles, ulcers, tonic, syphilis, poison, fever in children/babies, cough, aphrodisiac	[67,99,109,120,140,142,152,168]
*Oenanthe palustris* (Chiov.) C.Norman	Herb	Leaves	Stomach complications, poisonous to livestock	[109,168]
*Oenanthe procumbens* (H.Wolff) C.Norman	Herb	Leaves, seeds	Mouth infection in babies, cough	[67,109]
*Steganotaenia araliacea* Hochst.	Tree	Leaves, bark, aerial parts, roots, stems	Skin, partial blindness, edema, dysentery, diabetes mellitus, boils, protracted labor, retained afterbirth, malaria, acaricide, emetic, fever, pneumonia, spleen pain and enlargement, swollen legs and body pains during pregnancy, itchy eyes, sensitive eyes	[51,67,81,85,98,106,120,129,133,139,140,142,147]
*Torilis arvensis* (Huds.) Link	Herb	Leaves	Preventing miscarriage	[77]
**Apocynaceae**				
*Acokanthera oppositifolia* (Lam.) Codd	Shrub/tree	Bar, roots, fruits, leaves	Cancer, liver disease, poison	[72,88,100,142,168]
*Acokanthera schimperi* (A.DC.) Schweinf.	Shrub/tree	Roots, stems, leaves, bark	Blood pressure, HIV/AIDs, diarrhea, antivenom, syphilis, poisonous, ectoparasites (ticks, fleas, mites), malaria	[68,78,88,89,91,105,107,142,145,168]
*Adenium obesum* (Forssk.) Roem. & Schult.	shrub	Bark, root	Stomach ailments, snakebites, poisonous, ectoparasites	[68,72,77,127,134,142]
*Buckollia volubilis* (Schltr.) Venter & R.L.Verh.	Liana	Bark	Aid in child delivery, malaria	[78]
*Calotropis procera* (Aiton) W.T.Aiton	Shrub/tree	Roots, bark, exudates, fruits, stems	Determine unborn baby’s sex, toothache, yellow fever, anemia, malaria, camel cough, poisonous, emetic, vomiting, diarrhea, stomachache, foreign objects pierced into the body	[14,51,88,91,104,132,134,141,142,147]
*Carissa spinarum* L.	Shrub	Roots, fruits, bark, leaves, seeds	Abdominal pains, infertility in women, malaria, gonorrhea, syphilis, nose bleeding, diarrhea, typhoid fever, lactation, postpartum pain, pelvic pains, backache, skin allergies, seizures, fever, herpes zoster (shingles), amoebas, STIs, measles, joint pains, afterbirth pains, cancer, chest problems, cleans stomach, cough, colds/flu, energy boost, high blood pressure, painkiller, pneumonia, medicine antidote, rheumatism, TB, anthelmintic, chest pains, polio, arthritis, chest pains, stomachache, asthma, allergy, peptic ulcers, colic pain in infants, wounds, hypertension, diabetes, menstrual pains, ingestion and abdominal pains during pregnancy, bone pain, toothache, dizziness, headache, theileriosis, helminthiasis, salmonellosis, heart water, milk letdown, prevent first and second trimester, abortion, vomiting, infertility (sheep, goat), milk let down, mastitis, miscarriage (cow), tonic, constipation, eye infections, reduces body fat, dysentery, aphrodisiac, abdominal pains, breast cancer, acaricide, general malaise, appetizer, swelling and bruises, HIV/AIDs, strengthen bones, bloat, heartburn, cathartic, gynecological diseases, softens stool, digestion and cough (cattle), muscle pain, epilepsy	[14,41,64,68,71,72,73,74,76,77,78,79,81,82,83,86,87,88,89,90,91,93,97,98,99,101,102,103,105,106,107,108,112,113,118,119,120,123,124,129,130,132,133,135,136,137,138,140,141,142,143,146,147,150,151,154,168]
*Carissa tetramera* (Sacleux) Stapf	Shrub/tree	Roots, leaves	Convulsions	[127]
*Cascabela thevetia* (L.) Lippold (exotic)	Tree	Fruit, leaf, flower, bark, roots, fruits	Wounds, skin rashes, cuts/burns, lung problems in livestock, mosquito repellant, congested nose in children, skin disease, ringworms, induce sneezing	[74,76,87,106,108,143,150]
*Catharanthus roseus* (L.) G.Don (exotic)	Herb/shrub	Leaves, whole plant, roots	Throat/stomach/esophageal cancer, pneumonia, chest pain, gastrointestinal problems, malaria	[79,104,117,122,124,143]
*Cryptolepis oblongifolia* (Meisn.) Schltr.	Shrub	Roots	General body weakness	[140]
*Cynanchum altiscandens* K.Schum.	Shrub	Tuber, aerial parts	Colds, stomachache, circumcision wounds, increase milk letdown	[144,152]
*Cynanchum ethiopicum* Liede & Khanum	Herb	Roots	Pneumonia	[109]
*Cynanchum insipidum* (E.Mey.) Liede & Khanum	Herb	Leaves, roots	Stomachache, abnormal vaginal secretion, anaplasmosis, *Chira*, malaria, eye problem, reproductive disorders, increase milk letdown in animals	[68,76,127,144,150]
*Cynanchum viminale* (L.) L.	Herb	Roots, bark	Syphilis, bone pain, body pains, cough	[14,67,140]
*Desmidorchis retrospiciens* Ehrenb.	Herb	Whole plant	Infertility, chest problems, congestion/wheezing	[88,91]
*Desmidorchis speciosa* (N.E.Br.) Plowes	Herb	Exudate	Emetic	[67]
*Edithcolea grandis* N.E.Br.	Herb	Exudates	Ringworms	[51]
*Funtumia africana* (Benth.) Stapf	Tree	Leaves, bark	Boils, dysentery, diarrhea	[116]
*Gomphocarpus fruticosus* (L.) W.T.Aiton	Shrub	Seeds, roots, whole plant, exudate	Diarrhea, diabetes, fevers, abdominal pains, wounds, toothache (never to swallowed), liver diseases, kidney problems, chest pains, headache	[83,99,136,140,141,168]
*Gomphocarpus integer* (N.E.Br.) Bullock	Herb	Roots	Amoeba	[147]
*Gomphocarpus physocarpus* E.Mey.	Shrub	Stems, fruits, roots, exudate, leaves	Stomach problems in women, toothache, aids calf delivery in cows, flu, congested nose	[67,76,109,152]
*Gomphocarpus semilunatus* A.Rich.	Shrub	Leaves, whole plant	Gum lesions, toothache, syphilis, gonorrhea, gastroenteritis, malaria,nose infection, stomach problem, headache, infertility, purgative	[104,115,122,131,143]
*Kanahia laniflora* (Forssk.) R.Br.	Shrub		Epilepsy	[142]
*Landolphia buchananii* (Hallier f.) Stapf	Liana	Leaves, roots	Back/joint ache, venereal diseases, malaria, wounds, gonorrhea, molluscoid	[78,84,111,168]
*Landolphia kirkii* Dyer ex Hook.f.	Shrub	Roots	Stomach ailments	[127]
*Leptadenia lanceolata* (Poir.) Goyder	Liana	Leaves	General medicine	[91]
*Marsdenia abyssinica* (Hochst.) Schltr.	Liana	Bark, roots	Diarrhea, heartburn, oral thrush	[67,86,142]
*Marsdenia angolensis* N.E.Br.	Liana	Root	Anthelmintic, runny nose, poison	[14]
*Marsdenia rubicunda* (K.Schum.) N.E.Br.	Liana	Roots	Postnatal care, threatened miscarriage	[127,141]
*Marsdenia schimperi* Decne.	Liana	Root	Abdominal pain	[141]
*Mondia whitei* (Hook.f.) Skeels	Shrub	Roots, fruits, flowers, stems,	Appetizer, fatigue, mineral deficiency, aphrodisiac, respiratory problems, allergies, cough, colds/flu, dental disorders, malaria, rheumatism, stomach disorders, indigestion, heartburn, kidney ailments, asthma, gonorrhea, acaricide, anorexia, STIs, stimulant after giving birth, aphrodisiac, infertility, stomachache	[14,41,66,99,105,107,110,125,133,142,143,144,146,168]
*Nerium oleander* L. (exotic)	Shrub/tree	Leaves	Upper respiratory tract infections, gastrointestinal complications, blocked nose, chest pain	[68,122]
*Orbea dummeri* (N.E.Br.) Bruyns	Herb	Stems	Gastrointestinal ulcers, sores on lips/tongue, baby stomach problems	[139,140]
*Pachycarpus concolor* E. Mey.	Herb	Roots	Cattle sores caused by skin maggots	[67]
*Pergularia daemia* (Forssk.) Chiov.	Herb/shrub	Exudate, roots	Stomachache, rashes, dermatitis	[67,132,141]
*Periploca linearifolia* Quart.-Dill. & A.Rich.	Shrub	Roots, leaves, stems, exudate	Infertility, abdominal pains in pregnancy, ulcers, malaria, stomachache, headache, backache, diarrhea, colds, flu, cough, chest problems, bad smell of flatulence, mouth inflammation in children, anthelmintic, retained placenta, fever, joint pains, gonorrhea, teeth problems, spinal cord problem, aphrodisiac, herpes zoster, oral thrush, pneumonia, cancer, pre/postmenopausal symptoms, lumbago in women, diabetes, syphilis, pesticide, eye problems, amoeba, wounds, skin sores/ulcers, typhoid, body strength, warts	[41,78,83,84,87,88,91,99,103,104,107,109,120,128,131,132,141,152,168]
*Plumeria alba* L. (exotic)	Shrub/tree	Bark, root, leaves, exudate	Removal of thorn, wounds (cow, goat, sheep, human), STI’s	[14,73,146]
*Plumeria rubra* L. (exotic)	Shrub/tree	Exudate	Ringworms	[122]
*Rauvolfia caffra* Sond.	Shrub/tree	Bark, fruit, root	Allergies, amoebiasis, cough, colds/flu, pneumonia, rheumatism, stomach disorders, respiratory disorders, body weakness, chest diseases, bloat, ECF, anthelmintic, trachoma in cattle, arthritis, skin care, toothache	[14,41,80,100,143,146,147]
*Rauvolfia mannii* Stapf	Shrub/tree	Root, bark	Itching and pimples, arrow poison, malaria, headache, toothache, general body weakness, cough, arthralgia, anthelmintic in chicken	[14,145]
*Rauvolfia mombasiana* Stapf	Shrub/tree	Bark, roots	Malaria, poison, venereal diseases, stomach pains, skin conditions, swellings	[134,142]
*Saba comorensis* (Bojer ex A.DC.) Pichon	Liana	Roots, fruits	Prenatal care, diabetes, enlarged prostate	[41,91,127]
*Schizozygia coffeoides* Baill.	Shrub	Roots, bark	Skin diseases	[134,142]
*Secamone punctulata* Decne.	Liana	Roots	Fever, headache, chest pain, malaria, fever, stomach problems, gonorrhea, prevent abortion	[77,136,139]
*Strophanthus kombe* Oliv.	Liana		Poison	[134]
*Tabernaemontana pachysiphon* Stapf	Tree	Root, bark, stem, leaves	Malaria, anthelmintic, typhoid, amoeba, pneumonia, livestock diseases	[74,145,147]
*Tabernaemontana stapfiana* Britten	Tree	Roots, bark, fruits	Respiratory disorders, cancer, breast cancer, headache, stomachache, cough, toothache, wounds/cuts, cattle diseases (ECF, constipation), pneumonia, chest problems, aids in delivery	[14,78,88,99,117]
*Tabernaemontana ventricosa* Hochst. ex A.DC.	Shrub/tree	Latex	Amoeba, asthma, malaria, measles, wounds	[41,142]
*Tacazzea conferta* N.E.Br.	Liana	Root	Stimulate milk letdown (human/cattle)	[142]
*Vincetoxicum fruticulosum* (Decne.) Decne.	Shrub/liana	Root	Tonic	[99]
**Aquifoliaceae**				
*Ilex mitis* (L.) Radlk.	Shrub/tree	Bark	Infertility, abdominal pains	[141]
**Araceae**				
*Anchomanes abbreviatus* Engl.	Herb	Roots	Wounds, septic swellings	[127]
*Colocasia esculenta* (L.) Schott (exotic)	Herb	Roots, leaves	Colds (human, cattle, sheep), sore throat, anxiety, fast heart beat	[14,127,144]
*Culcasia falcifolia* Engl.	Herb	Leaves	Dry cough, ECF, edema, epilepsy	[78]
*Gonatopus boivinii* (Decne.) Engl.	Herb	Roots	Mumps	[127]
*Stylochaeton salaamicus* N.E.Br.	Herb	Whole plant	Induce salivation in babies	[127]
*Zamioculcas zamiifolia* (G.Lodd.) Engl.	Herb	Whole plant	Eardrops	[127]
**Araliaceae**				
*Astropanax abyssinicus* (Hochst. ex A.Rich.) Seem.	Tree	Leaves, exudate	Bronchitis, cough, pneumonia, aphrodisiac, cleaning the womb	[131,147]
*Astropanax volkensii* (Harms) Lowry, G.M.Plunkett, Gostel & Frodin	Shrub/tree	Leaves, bark, stem, exudate	Chest pains, malaria, pneumonia, livestock diseases, fever, kidney problems, colds, sore throat, whooping cough, swollen bodies, cough, inhaled to clear blocked nose, typhoid, TB, goiter, rashes, allergies, headache, expectorant, liver problems, skin diseases (goats/sheep)	[67,91,109,120,132,141,142,152,168]
*Cussonia arborea* Hochst. ex A.Rich.	Tree	Leaves	Stomachache, childbirth, veterinary uses, constipation	[116,144]
*Cussonia holstii* Harms ex Engl.	Tree	Bark, roots	Abdominal pains, bones/joints problems, cough, colds/flu, kwashiorkor, malaria, rheumatism, clear uterus after parturition, fever, alcoholic tremors, stimulant, retained placenta, tonic, ear-nose-mouth infection, irregular menstruation, wounds (cows), backache, stomachache	[14,41,67,71,77,105,136,138,142,147,168]
*Cussonia spicata* Thunb.	Tree	Leaves, bark, roots	STI’s, fever, spinal cord problem, teething, abdominal upsets, stimulant, liver enlargement, abdominal pains, diarrhea, splenomegaly, painkiller, emetic, malaria	[77,88,109,132,141,145,152]
*Cussonia zimmermannii* Harms	Tree	Roots	Kwashiorkor	[127]
*Polyscias fulva* (Hiern) Harms	Tree	Bark	Obesity	[78]
*Polyscias kikuyuensis* Summerh.	Tree	Leaves, bark	Joint pains, arthritis, rheumatism, wounds, skin diseases	[41,88,109,168]
**Arecaceae**				
*Cocos nucifera* L. (exotic)	Tree	Fruits, leaves	Skin rash, asthma, kidney problems, wounds	[108,150]
*Hyphaene compressa* H.Wendl.	Tree	Bark	Anthelmintic	[14]
*Phoenix reclinata* Jacq.	Tree	Root, leaves, exudate	Stomachache, chest problems, stimulant	[14,41,80,152]
**Aristolochiaceae**				
*Asarum canadense* L. (exotic)	Herb		Cough, colds/flu, mental ailments, pneumonia, STI’s	[41]
*Aristolochia albida* Duch.	Herb		Snake antidote, poison	[134]
**Asparagaceae**			Root, leaves, exudate	
*Agave sisalana* Perrine (exotic)	Tree	Roots, leaves, exudates	Earache, burns, eye problems, injuries, cuts/wounds, acaricide, gonorrhea, appetizer, anthelmintic, retained placenta, mosquito repellant, fed to livestock to increase milk letdown	[14,41,64,84,106,120,133,139,144,152]
*Albuca abyssinica* Jacq.	Herb	Roots	Arthritis, sores	[67,154]
*Albuca bracteata* (Thunb.) J.C.Manning & Goldblatt	Herb	Roots	Bone injuries, wounds, cancer, colds, constipation/bloat (cattle)	[14,67,91,152]
*Albuca virens* (Lindl.) J.C.Manning & Goldblatt	Herb	Roots	Diabetes, back pains, tonsils, arthritis, poisonous to livestock	[83,91,115,168]
*Asparagus africanus* Lam.	Liana	Roots, leaves	Boils, mental illness, wounds, venereal diseases, cough, sore throat, skin infections, urinary tract infections, arthritis, gonorrhea, parturition	[67,89,94,123,135,154]
*Asparagus buchananii* Baker	Shrub/liana	Leaves, roots	Prevent second trimester abortion, pregnancy edema	[81]
*Asparagus falcatus* L.	Herb	Stems, roots, leaves	Stomach ailments, constipation, convulsions, teeth problem, rashes, rheumatoid arthritis	[127,132,138]
*Asparagus flagellaris* (Kunth) Baker	Shrub	Leaves, roots, fruits	Boils, abscesses, stomachache, syphilis, conjunctivitis	[94,99]
*Asparagus racemosus* Willd.	Liana	Leaves, roots	Arthritis, infertility in women, cancer, boils, stomachache, gonorrhea, immuno-booster, syphilis, malaria, labor, scrotum problems, removing thorn, rheumatism, improve sexual desire, indigestion, kidney diseases, abdominal pains, bone setting, jaundice, venereal diseases, HIV/AIDs, anthelmintic (cattle), retained placenta, lower back pain	[14,84,87,99,104,109,120,131,141,143,146,147,152,168]
*Asparagus setaceus* (Kunth) Jessop	Herb	Roots, leaves	Back pain in women, postpartum pain, STI’s, boils, toothache, abscess, diarrhea, sores, wounds, medicine for cattle, rheumatoid arthritis	[14,113,115,119,138,147]
*Chlorophytum subpetiolatum* (Baker) Kativu	Herb	Roots	Reduce madness	[99]
*Dracaena afromontana* Mildbr.	Shrub/tree	Leaves, roots, stems	Stomach problems, swollen spleen, chest pains	[99,131]
*Dracaena arborescens* (Cornu ex Gérôme & Labroy) Byng & Christenh.	Herb	Roots, leaves	Venereal diseases, snakebites	[127]
*Dracaena fragrans* (L.) Ker Gawl.	Shrub/tree	Leaves, roots	labor pains, chase mosquitoes	[144]
*Dracaena frequens* (Chahin.) Byng & Christenh.	Herb	Roots	Immune booster	[14]
*Dracaena parva* (N.E.Br.) Byng & Christenh.	Herb	Leaves, exudate	Snakebites, wounds	[126,143]
*Dracaena perrotii* (O.Warburg) Byng & Christenh.	Herb	Exudates, roots	Gonorrhea, rheumatism, arthritis, snakebites, earache	[51,72,143]
*Dracaena steudneri* Engl.	Tree	Bark, leaves, roots	Cancer, toothaches, rheumatism, anthelmintic, skin cancer, diabetes, stomachache, measles, diarrhea, veterinary medicine (goat), reduce pains birth and prevent dizziness at childbirth birth	[41,83,116,144,145,154]
*Dracaena suffruticosa* (N.E.Br.) Byng & Christenh.	Herb	Bark, branches, roots, stems	Gonorrhea	[135]
*Dracaena volkensii* (Gürke) Byng & Christenh.	Herb	Exudates, stems	Eye problems, STIs, snake repellant	[91,92,95]
*Drimia indica* (Roxb.) Jessop	Herb	Root	Ulcers, cancer	[91]
**Balsaminaceae**				
*Impatiens hochstetteri* Warb.	Herb	Roots	Mouth infection in babies	[109]
*Impatiens sodenii* Engl. & Warb.	Herb	Roots	Difficult births in women, joint pain, purgative	[77,141,152]
*Impatiens stuhlmannii* Warb.	Herb	Fruits	Fever, dizziness, vitamin C	[144]
*Impatiens tinctoria* A.Rich.	Herb	Roots	Retained placenta, infertility, purgative, abdominal pains, thrush in mouth	[120,131,141]
**Basellaceae**				
*Basella alba* L.	Herb	Leaves, roots	Abdominal/colic pains, anemia, stomachache, joint pains, burns, child delivery, retained placenta, lumbago, blood cleanser, breathing difficulty, diarrhea, amoebiasis, irregular menstruation, constipation, anthrax, bloat in cattle, antidote, increases milk production, stomach pain/constipation after birth	[14,78,84,87,88,109,115,120,131,132,143,144,152,168]
**Berberidaceae**				
*Berberis holstii* Engl.	Shrub	Stem, roots, leaves, bark	Malaria, wounds, anthelmintic, stomachache, pain relief	[99,104,107,132]
**Bignoniaceae**				
*Fernandoa magnifica* Seem.	Tree	Roots	Chest medicine, female reproductive disorders	[142,150]
*Jacaranda mimosifolia* D.Don (exotic)	Tree	Leaves	STIs, acaricide	[41,133]
*Kigelia africana* (Lam.) Benth.	Tree	Bark, roots, fruits, leaves, seeds	Measles, liver diseases, edema, stomachache, anemia, arthritis, asthma, cleans digestive system, depression, aphrodisiac, rheumatism, STIs, typhoid, abortifacient, headache, malaria, purify blood, amoeba, anthelmintic (cow, poultry, goat, sheep, pig, human), dystocia (cow), breast/uterine/skin cancer, gut problems, gonorrhea, syphilis, kidney diseases, piles, snakebite antidote, leprosy, rashes, pneumonia, skin sores and ulcers, colds/flu, sores/wounds, promote conception and pregnancy, cholesterol control, abdominal pains, infertility in women, emaciation, skin diseases, ulcers, diabetes, purgative, diarrhea	[14,41,72,73,76,78,79,84,88,89,98,103,104,105,108,110,116,117,121,132,139,141,143,144,146,147,168]
*Markhamia lutea* (Benth.) K.Schum.	Tree	Leaves, bark, root	Eye infection (cattle/human), toothache, snakebites, arthritis, skin inflammation, myalgia, earache, malaria, cataract, amoeba, back/joint/bone/chest problems, diarrhea, family planning, injuries/cuts/wounds, measles, pancreas problems, rheumatism, STIs, stomach disorders, typhoid, arthritis, colorectal cancer, stomachache, sore throat, flu, stomachache, measles, cardiac conditions, kidney ailments, acaricide, ringworms, menstrual pains, pneumonia	[14,41,66,76,87,101,116,117,119,120,122,124,125,133,143,147,154]
*Markhamia zanzibarica* (Bojer ex DC.) K.Schum.	Shrub/tree	Leaves, roots	Abdominal pain, bleeding and edema in pregnancy, snakebites, bleeding during childbirth	[87,127]
*Spathodea campanulata* P.Beauv.	Tree	Bark, leaves, stems, exudate	Liver disorders, headache, sore throat, stomachache, malaria, chest, backache, STI’s, arthritis, tongue infections, cervical/bone/breast/colorectal/skin cancer, abortifacients, infertility, veterinary uses, rashes in children that turn into wounds, appetizer, cut/wounds/burns, chest pain, liver complaints, livestock diseases (increase milk production, bloat, cough, fever), sore throat, headache, colds	[66,78,101,116,117,119,120,122,142,144,154]
*Stereospermum kunthianum* Cham.	Shrub/tree	Roots, leaves, bark, flowers, fruits	Cough, malaria, ulcers, venereal diseases, wounds, acaricide	[66,133,144]
**Boraginaceae**				
*Bourreria nemoralis* (Gürke) Thulin	Shrub/tree	Roots	Stomach problems, kidney ailments, asthma	[41,127]
*Bourreria petiolaris* (Lam.) Thulin	Shrub/tree	Roots	Female reproductive disorders	[150]
*Cordia africana* Lam.	Tree	Bark, leaf, fruit, aerial parts	Vitamins, pneumonia, broken limbs, amoeba, back/joint/bone problems, cancer, eye problems, family planning, heartburns, injuries/cuts, wounds, livestock diseases, labor pains, stomach disorders, typhoid, prevents abortion, malaria, eye diseases pain (cow), general weakness (cow, goat, sheep, pig), bone injuries, toothache, cough, venereal diseases	[14,41,73,84,88,104,105,119,120,144,146,147]
*Cordia monoica* Roxb.	Shrub/tree	Roots, bark, leaves, fruits	Backache, diarrhea, leprosy, mental illness, ocular disorders, retained placenta, livestock diseases, fever, joint/chest pains, stomach disorder, malaria, vomiting, eye ache/infection, fractures, gonorrhea	[67,71,77,80,89,99,105,135,136,145,147]
*Cordia sinensis* Lam.	Shrub/tree	Roots, flowers, bark, exudate	Eye problems, diarrhea, malaria, chest problems, pneumonia, fractures, woman’s stomach, fevers, eye infection, stomachache	[67,68,72,77,95]
*Cynoglossum coeruleum* Hochst. ex A.DC.	Herb	Leaves	Back/joint/bone problems, skin rashes, ulcers, chest pain, wounds, parturition (man, livestock), sore throat	[41,115,122,144,168]
*Echiochilon lithospermoides* (S.Moore) I.M.Johnst.	Herb	Whole plant	Livestock medicine	[77]
*Ehretia bakeri* Britten	Shrub/tree	Bark	Gastrointestinal complications, upper respiratory tract infections, malaria, muscle ache, toothache	[68,145]
*Ehretia cymosa* Thonn.	Shrub/tree	Leaves, roots, bark, aerial parts	Abdominal ulcers, livestock diseases, malaria, rheumatism, anaplasmosis (cow, ruminant), diarrhea/dysentery (human, cow, poultry, goat, sheep, pig), boils, stomachache, chest pain, boils, aphrodisiac, typhoid, muscle ache, toothache, impotency, wounds, pain killer, cough, colds, vomiting, venereal diseases, pneumonia, epilepsy, ECF, tonsils, mental problems, asthma	[14,41,73,78,84,88,91,116,122,141,144,145,147,168]
*Euploca rariflora* (Stocks) Diane & Hilger (exotic)	Herb	Root	Joint diseases	[149]
*Heliotropium steudneri* Vatke	Herb	Leaves	Fleas, arrow poison wounds	[72,147]
*Heliotropium zeylanicum* (Burm.f.) Lam.	Herb	Roots	Boils, abscesses,	[94]
*Symphytum officinale* L. (exotic)	Herb	Roots	Wounds, joints/bones, problems, inflamed muscles, ulcers, boils, dry cough	[168]
*Trichodesma zeylanicum* (Burm.f.) R.Br.	Herb	Roots, leaves, whole plant	Snakebites, TB, ulcers, stomachache, colds/flu, wounds	[84,99,148]
**Brassicaceae**				
*Brassica oleracea* L. (exotic)	Herb		Heartburns	[41]
*Cardamine obliqua* Hochst. ex A.Rich.	Herb	Scurvy	Scurvy	[147]
*Lepidium bonariense* L. (exotic)	Herb	Stems, leaves	Cleaning stomach after childbirth	[77]
*Lepidium sativum* L. (exotic)	Herb	Seeds	Bone/joint pains, fever, respiratory diseases, stomachache	[137]
*Nasturtium officinale* W.T.Aiton (exotic)	Herb	Leaves	Peptic ulcers, anemia, allergies, blood cleanser, respiratory diseases, cough	[88,168]
*Spinacia oleracea* L. (exotic)	Herb		Pneumonia	[41]
**Burseraceae**				
*Boswellia neglecta* S.Moore	Shrub/tree	Bark, resin	Chest, headache, ribs, diarrhea, stomach disorders, swollen legs and stomach pain in pregnant women	[72,77,100,139]
*Commiphora africana* (A.Rich.) Engl.	Shrub/tree	Fruits, bark, roots, exudates	Abdominal pains, renal diseases, wounds, skin moisturizer, jiggers, eye problem, convulsions, venereal diseases, boils, skin rashes, edema, ulcers, swollen testicles, snakebite, typhoid, diarrhea, stomach problems, malaria, pneumonia, brucellosis, back/pain/joint pain, dysmenorrhea, burns, gum diseases, cough, gynecological problems	[71,72,77,79,84,87,89,92,96,98,104,105,107,108,127,138,141,143,148,150]
*Commiphora baluensis* Engl.	Tree	Bark, roots	Stomach disorders, body swellings, typhoid, malaria, diarrhea (human, poultry), swollen diaphragm	[51,77,100,139,145]
*Commiphora edulis* (Klotzsch) Engl.	Shrub	Bark, leaves	Convulsions, diarrhea, sore throat, pregnancy stomach pains, retained placenta, tapeworms, cough, chest pains	[51,100,127,139]
*Commiphora eminii* Engl.	Tree	Roots, leaves, branches, bark	After-birth pains, constipation, hard stool, rheumatism, STIs, stomach disorders, malaria, stomachache, skin rushes, colds, pneumonia, anthrax, constipation, acidity, muscle pains, stomach crumps, measles, cough (cow, goat, sheep, poultry, pig, ruminants, human), anaplasmosis (cow, ruminants), helminthiasis (cow, poultry, goat, sheep, pig), diarrhea/dysentery (cow, poultry, goat, sheep, pig), pleuritis, bloat, cuts/wounds	[14,41,133,146,147]
*Commiphora kataf* (Forssk.) Engl.	Tree	Fruits	Toothache	[64,77]
*Commiphora kua* (R.Br. ex Royle) Vollesen	Shrub/tree	Roots, bark, leaves, exudate	Body swelling, prenatal care, wounds, ringworms, toothache, acaricide (goats, cattle)	[51,94,100,127]
*Commiphora oddurensis* Chiov.	Shrub	Bark	Abdominal pains	[139]
*Commiphora ovalifolia* J.B.Gillett	Shrub/tree	Bark, exudate	Cellulitis, scabies	[80,94]
*Commiphora rostrata* Engl.	Shrub/tree	Leaves, twigs	Mild chest pains, cough	[80]
*Commiphora schimperi* (O.Berg) Engl.	Shrub/tree	Root, bark, leaves, exudate	Malaria, constipation, wounds	[97,100,111]
*Commiphora swynnertonii* Burtt	Shrub/tree	Stems, leaves	Gonorrhea	[105,107]
**Buxaceae**				
*Buxus obtusifolia* (Mildbr.) Hutch.	Shrub/tree	Root	Chest complaints	[142]
**Cactaceae**				
*Opuntia ficus-indica* (L.) Mill. (exotic)	Shrub	Fruits	Boils	[147]
*Rhipsalis baccifera* (J.S.Muell.) Stearn	shrub	Roots, branches	Gonorrhea	[99]
**Campanulaceae**				
*Lobelia fervens* Thunb.	Herb	Whole plant	Body swellings	[134]
*Lobelia giberroa* Hemsl.	Shrub	Stems, roots, leaves	Urinary tract infections, joint aches, malaria, ECF	[77,145,147]
*Lobelia holstii* Engl.	Herb	Roots	Cough, stomachache	[99]
**Canellaceae**				
*Warburgia stuhlmannii* Engl.	Tree	Bark, leaves	Toothache, rheumatism, ease pains, joint pains, cough, malaria, chest pain, heart pain, antioxidant, veterinary medicine	[134,142,150]
*Warburgia ugandensis* Sprague	Tree	Bark, stems, leaves, roots, seeds, fruits	Eye problems, stomachache, internal wounds, bloat, malaria, cough, appetizer, chest and lungs problems, diarrhea, respiratory problems, back pain in women, cleansing uterus, colds/flu, healing after delivery, chicken pox, stinking feet, gonorrhea, pneumonia, skin problems, livestock diseases, anthelmintic, headache, allergies, toothache, amoebiasis, asthma, back/bone/joints problems, diabetes, energy boost, general body pains, high blood pressure, meat appetizer, prostate cancer, rheumatism, STIs, swellings, TB, typhoid, ulcers, abdominal disorders, arthritis, constipation, general body weakness, fever, muscular pains, internal wounds, bacterial infections, heart water, ectoparasites, black quarter, emetic, trypanosomiasis, ECF, helminthiasis (cow, poultry, goat, sheep, pig), pneumonia (cow, goat, sheep, poultry), general weakness/dullness (cow, goat, sheep, poultry, pig), sore throat, skin diseases, oral thrush, gastrointestinal problems, blood/mucus in stool, afterbirth stomach pains, prophylaxis for children diseases, cough (cattle), anodyne, expectorant, erectile, dysfunction/impotence, abdominal disorders, heart problems, gout, purgative, tonsils, uvala problems	[14,41,64,67,68,71,72,73,74,77,78,79,82,84,86,88,89,90,91,92,95,98,99,101,104,105,107,109,110,116,119,120,123,129,132,135,136,138,140,141,142,143,144,146,147,152,153,168]
**Cannabaceae**				
*Cannabis sativa* L. (exotic)	Herb	Leaves, flowers, seeds	Wounds, diarrhea/dysentery (cow, poultry, goat, sheep, pig), pneumonia (poultry), Newcastle disease (poultry), stimulant	[73,144,146]
*Celtis africana* Burm.f.	Tree	Roots, stems	Bone problems, livestock diseases, antipruritic, stomachache	[14,152]
*Celtis gomphophylla* Baker	Tree	Roots, leaves, roots	Backache, general body illness, infertility, anthelmintic, STIs	[66,116]
*Celtis philippensis* Blanco	Shrub/tree	Roots	Diarrhea	[142]
*Chaetachme aristata* Planch.	Shrub/tree	Leaves, roots, bark	Malaria, strength, skin, boils, infertility	[77,116,120]
*Trema orientale* (L.) Blume	Shrub/tree	Roots, bark	Afterbirth pains, bacterial infections, chest problems, cough, colds/flu, stomach disorders, anthelmintic, yellow fever, insect repellant, constipation	[41,144,148,168]
**Capparaceae**				
*Boscia coriacea* Graells	Shrub/tree	Leaves, roots, fruits, seeds	Dental problems, detoxifier, eye problems, meat appetite, pneumonia, prostate cancer, tonsillitis, stomachache, fleas/mites, gonorrhea, malaria, burns, hepatitis, livestock diseases, obesity, food poisoning, abdominal pains, partial blindness/eye injuries in cattle, anthelmintic and diarrhea (human, goats, cattle)	[14,41,51,64,67,72,77,80,91,100,132,139]
*Boscia integrifolia* J.St.-Hil.	Tree	Stems, leaves, bark	Diarrhea, gonorrhea, cattle fever, gynecological disorders, malaria, retained afterbirth, prevent first trimester abortion, vomiting, hepatitis, diarrhea, joint pains, swelling/bruises due to thorns	[71,72,77,81,89,97,103,104]
*Boscia salicifolia* Oliv.	Tree	Bark, leaves	Backache, chronic joint pains	[130,142]
*Cadaba farinosa* Forssk.	Shrub/tree	Fruits, roots, stems, leaves	Emetic, pregnancy anemia, STIs, colds, fever, snakebites	[72,100,134,135,142]
*Cadaba glandulosa* Forssk.	Shrub	Root	Purgative	[142]
*Capparis cartilaginea* Decne.	Shrub	Root, bark, leaves	Eye lotion, heartburn, peptic ulcers, skin sores/ulcers, abdominal pains,swollen legs and body pains during pregnancy, retained placenta	[67,91,139,141]
*Capparis fascicularis* DC.	Liana	Roots, leaves, fruits	Venereal diseases, diarrhea, stomachache, colds, poisonous, wounds, burns, anthelmintic, false teeth, gastrointestinal problems, stomach pains	[67,72,76,79,108,127,142,143]
*Capparis sepiaria* L.	Liana	Roots, leaves	Poisonous, colds, emetic, purgative, chest pains, wounds, malaria, menstrual cramps	[74,142,150]
*Capparis tomentosa* Lam.	Liana	Roots, leaves, fruits	Respiratory problems, determine unborn baby’s sex, poisonous to camels, emetic/purgative, cough, colds/flu, back/bone joint problems, prostate cancer, wounds, burns, muscle ailment, painkiller, female infertility, menstrual problems	[41,77,88,105,136,142,145,150]
*Capparis viminea* Oliv.	Liana	Roots	Venereal diseases, infertility, septic swelling, poisonous	[127,142,168]
*Crateva adansonii* DC.	Shrub/tree		Amoebiasis	[132]
*Crateva kirkii* (Oliv.) Christenh. & Byng	Shrub/tree	Leaves	Wounds	[142]
*Maerua angolensis* DC.	Shrub/tree	Leaves, fruits	Toothache, colds, poison, wounds	[134,136]
*Maerua crassifolia* Forssk.	Shrub/tree	Leaves, roots, bark	Wound, stomachache	[132,142]
*Maerua denhardtiorum* Gilg	Shrub/tree	Leaves	Eye infection	[77]
*Maerua edulis* (Gilg & Gilg-Ben.) DeWolf	Shrub	Roots, bark	Sore joints, stimulant, amoeba, diabetes, stomach problems, eye problems, swollen legs and body pains during pregnancy	[67,86,100,139,142,143]
*Maerua endlichii* Gilg & Gilg-Ben.	Shrub	Stems, roots, leaves	Swollen/bruised skin, purgative, wounds, snakebites/spits, swollen diaphragm	[14,51,77,80]
*Maerua kirkii* (Oliv.) F.White	Shrub/tree	Leaves, roots	Constipation and stomach disorders, wounds, sore throat	[80,100]
*Maerua subcordata* (Gilg) DeWolf	Tree	Roots, leaves	Colic pain, anorexia, blood cleanser, pneumonia, mites, bone/joint pains, gland complications, stomachache, toothache, women’s health, malaria	[14,80,88,137]
*Maerua triphylla* A. Rich.	Shrub/tree	Leaves, roots, shoots,	Wounds, aphrodisiac, headache, tonic, STIs, gonorrhea, backache, toothache, chest congestion, skin disorders, burns, boils, eye lotion, diarrhea, aphrodisiac, bone pain, pain in pregnancy, vaginal bleeding during pregnancy, eye problems, rheumatism, venereal diseases, cuts, sores, rheumatoid, arthritis, mouth blisters	[72,76,77,88,90,99,113,136,138,140,143,152,168]
**Caricaceae**				
*Carica papaya* L. (exotic)	Tree	Roots, leaves, stems, bark, exudate	STIs, malaria, gonorrhea, pneumonia, afterbirth pains, allergies, asthma, back/joint/bone problems, body weakness, chest problems, constipation, cough, colds/flu, dental problems, diarrhea, heartburn, injuries/cuts, wounds, chicken diseases, retained placenta, prostrate problems, rheumatism, skin diseases, anthelmintic, stomach disorders, typhoid, toothache, ulcers, syphilis, lung ailment, amoeba, respiratory, pneumonia, miscarriage,cervical/colorectal/breast, skin problems, ringworms, bronchitis, venereal diseases, gastrointestinal problems, diuretic, cattle cough, induce labor	[14,41,74,84,96,98,106,107,108,113,116,117,119,139,143,146,150]
**Caryophyllaceae**				
*Drymaria cordata* (L.) Willd. ex Schult.	Herb	Aerial parts	Fever	[144]
*Pollichia campestris* Aiton	Herb	Roots	Malaria, emetic	[67]
*Stellaria media* (L.) Vill.	Herb	Leaves	Heartburn, cough, sore throat, skin diseases	[168]
**Celastraceae**				
*Catha edulis* (Vahl) Endl.	Shrub/tree	Bark, roots, leaves, aerial parts	Skin diseases, TB, cough, colds/flu, dental problems, diarrhea, livestock flu, rheumatism, fatigue, toothache, gonorrhea, stimulant, gum, acidity, pneumonia, malaria, STI’s	[14,41,67,72,74,77,104,106,120,146,147,168]
*Elaeodendron buchananii* (Loes.) Loes.	Tree	Bark, roots, leaves	Cuts, wounds, syphilis, respiratory disorders, diarrhea, urinary tract problems, malaria, stomach problems, sores, earache, poisonous to livestock and human, kidney problems	[89,104,105,123,138,142,168]
*Gymnosporia arbutifolia* (Hochst. ex A.Rich.) Loes.	Shrub/tree	Fruit, leaf, stems, bark, root	Malaria, wound, acaricide, cough, tuberculosis, general body itching	[103,119,133,140]
*Gymnosporia buchananii* Loes.	Shrub/tree	Bark, roots	Stomachache, cancer	[99,145]
*Gymnosporia heterophylla* (Eckl. & Zeyh.) Loes.	Shrub/tree	Bark, leaves, roots, fruits, stems	Headache, diabetes, gonorrhea, malaria, wounds, infertility in women, eye inflammation, prenatal care, venereal diseases, liver problems, breathing problems, STIs, gut problems, syphilis, boils, acaricide, chest pain	[71,77,99,103,104,116,120,127,133,135,136,143,144,152]
*Gymnosporia mossambicensis* (Klotzsch) Loes.	Shrub/tree	Roots	Prenatal care	[127]
*Gymnosporia obscura* (A.Rich.) Loes.	Shrub/tree	Roots	Arthritis, cancer of breast and prostate glands	[84,154]
*Gymnosporia putterlickioides* Loes.	Shrub/tree	Roots, bark	Joint pains, diarrhea, toothache, ear infections, malaria, tonic, stomachache, abdominal pains	[77,80,96,104,129,138,139]
*Gymnosporia senegalensis* (Lam.) Loes.	Shrub/tree	Bark, roots, aerial parts, leaves, stems, fruits	Malaria, gynecological conditions, lumbago, blood cleanser, amoebiasis, back/joint/bone problems, constipation, cough, colds/flu, fever, indigestion, malaria, stomach disorders, mild stimulant, TB, stomachache, toothache, boils, arthritis, body aches, stomachache, acaricide, poison, pregnancy pain, good health of fetus, anthrax, wounds, sores, respiratory system, chest complaints, diarrhea, dysentery, gastrointestinal diseases, menstrual pain, eye infections, nausea, severe headache, aphrodisiac, kidney problems	[14,41,64,68,72,76,79,80,84,88,105,107,124,128,129,133,140,142,144,146,147,168]
*Loeseneriella africana* (Willd.) R.Wilczek	Liana	Roots, leaves	Gastrointestinal pains, diarrhea, colorectal/skin/breast cancer, aphrodisiac, male impotency	[117,127,168]
*Maytenus acuminata* (L.f.) Loes.	Shrub/tree		Tonic, stronger bones	[142,168]
*Maytenus undata* (Thunb.) Blakelock	Shrub/tree	Roots, bark, stems, leaves	Diarrhea, antiseptic, wounds, teething, diarrhea, false teeth, tonic, malaria, stomach disorders, laxative, general body wellness	[14,67,77,100,103,109,127,142,152]
*Mystroxylon aethiopicu*m (Thunb.) Loes.	Shrub/tree	Bark, leaves, roots, seeds	Pneumonia, colic pain in children, stomach disorder, antiseptic, colic pain	[67,77,89,107,135]
**Chrysobalanaceae**				
*Parinari curatellifolia* Planch. ex Benth.	Tree	Bark	Bleedings, diarrhea, injuries, cuts, wounds, malaria	[41,104]
**Cleomaceae**				
*Cleome gynandra* L.	Herb	Roots, leaves, stems, whole plant	Stomachache, colic pain in infants, ear infection, blood cleanser, diarrhea, leprosy, acaricide, typhoid, stomach problems, conjunctivitis, thread worms, stomach congestion, facilitate birth, epileptic fits, malaria, retained afterbirth	[66,76,78,79,88,91,108,120,132,133,144,147]
*Cleome strigosa* (Bojer) Oliv.	Herb	Leaves	Earache	[134]
*Cleome usambarica* Pax	Herb	Roots	Eye problems, tonic	[136]
**Clusiaceae**				
*Garcinia buchananii* Baker	Tree	Twig, bark, root, fruit	Stomach problems, diabetes mellitus, chest pain, asthma, diarrhea, abdominal discomfort, respiratory symptoms in livestock, acaricide	[82,85,131,133]
*Garcinia livingstonei* T.Anderson	Tree	Bark, roots	Childbirth pains, stomach disorder, tonic, typhoid, stomachache, teeth problems, liver pain and enlargement	[67,77,132,140]
**Colchicaceae**				
*Gloriosa superba* L.	Herb	Whole plant, roots, exudate	Abortifacient, poison, abdominal pains, wounds	[89,134,141,143,144,168]
**Combretaceae**				
*Combretum aculeatum* Vent.	Shrub	Roots, leaves	Stomachache, constipation, wounds, sores, malaria, gonorrhea, back pains, hepatitis, dysentery, hiccup, retained placenta, abdominal pains	[14,72,77,80,139]
*Combretum apiculatum* Sond.	Shrub/tree	Roots, bark, leaves	Stomach pains, eye infections, hypertension, female infertility, gonorrhea, skin diseases, bloody urine, scorpion bite, diarrhea, snakebites, cancer, ear problems	[14,80,86,91,99,119]
*Combretum collinum* Fresen.	Shrub/tree	Root, bark, leaves, exudate	Infertility in women, snakebites, amoebiasis, back/bone joints problems, cough, colds/flu, diarrhea, malaria, pneumonia, stomach disorders, TB, typhoid, acaricide, gastrointestinal problems, dysentery	[41,79,87,126,133,143]
*Combretum exalatum* Engl.	Shrub/tree	Roots	Swollen legs and body pains during pregnancy	[139]
*Combretum hereroense* Schinz	Shrub/tree	Leaves, roots	Chest pains, tuberculosis, cough	[51,127]
*Combretum illairii* Engl.	Shrub/liana	Leaves, bark, roots	Venereal diseases, cough, colds, malaria, female infertility	[75,127,150]
*Combretum molle* R.Br. ex G.Don	Shrub/tree	Leaves, bark, roots, stems, branches	STI’s, backache, respiratory problems, kidney problems, back/joint problems, cough, colds/flu, diarrhea, general body pains, malaria, medicine antidote, typhoid, warts, liver disease, sore throat, rib pain, stomachache, boils, gonorrhea, snakebites, acaricide, gastrointestinal problems, colic pain, typhoid, abortion, leprosy, fever, pain, hookworm, bone fractures, abdominal pain, constipation, anthelmintic, dysentery, headache, HIV infections	[76,77,79,80,84,90,105,107,116,118,119,133,135,138,140,145,168]
*Combretum mossambicense* (Klotzsch) Engl.	Shrub	Exudate	Earache	[80]
*Combretum padoides* Engl. & Diels	Shrub/tree	Roots, leaves	Bloody diarrhea, wounds, conjunctivitis, malaria	[75,111]
*Combretum paniculatum* Vent.	Shrub/liana	Roots, seeds	Poisonous, STIs, joint ailments, stomachache, birth pains, fever	[142,144]
*Combretum pentagonum* M.A.Lawson	Liana	Leaves	Headache	[142]
*Combretum schumannii* Engl.	Shrub/liana	Bark	Reproductive disorder, body swellings	[150]
*Combretum zeyheri* Sond.	Tree		Stomach disorders	[41]
*Terminalia brownii* Fresen.	Shrub/tree	Roots, bark, leaves	Skin disorders emetic, fevers, colds, cholera, flu, cough, eye problems, malaria, jaundice, amoebiasis, back/bone problems, diabetes, family planning, inflammations, injuries, cuts/wounds, livestock diseases, pneumonia, rheumatism, stomach disorders, STIs, yellow fever, ulcers, clotting agent (human, cattle), joint stiffness/pains, excessive bleeding after birth, sterilize dogs, induce abortion, syphilis, gonorrhea, heartburn, jaundice, arthritis, trypanosomiasis, prevent second trimester abortion, pregnancy edema, pregnancy anemia, allergy, kidney, family planning, abdominal/colic pains, cancer, blindness, typhoid, stomachache, teeth problems, waterborne diseases, asthma, urinary tract infection, dysmenorrhea, debility, abdominal pains, allergy, abortion, ringworm, anthelmintic in (human, animals), toothache, poison for hunting, infertility, edema, chest pains, reddening of child’s anal pore, retained placenta, appetizer, swollen legs and body pains during pregnancy, diarrhea	[14,41,51,64,68,74,80,81,84,85,86,91,96,98,100,104,105,106,129,130,132,137,139,140,141,142,143,144,145,146,148,151]
*Terminalia kilimandscharica* Engl.	Tree	Bark	Cough, STI’s	[153]
*Terminalia mollis* M.A.Lawson	Shrub/tree	Stems, roots, bark	Gut problems, diarrhea, stomachache, acaricide	[116,133]
*Terminalia orbicularis* Engl. & Diels	Shrub/tree	Bark	Bone/joint pain	[137]
*Terminalia prunioides* M.A.Lawson	Shrub/tree	Bark, leaves	Postnatal pains, mild madness, ringworms, peptic ulcers, kidney pains, aphrodisiac	[51,80,142]
*Terminalia spinosa* Engl.	Shrub/tree	Bark, roots	Stomach ailments, jaundice, malaria, sore throat	[91,100,127,142]
*Terminalia tetraptera* (Wickens) Gere & Boatwr.	Tree	Bark	Venereal diseases, wounds, men aphrodisiac	[150]
**Commelinaceae**				
*Aneilema aequinoctiale* (P.Beauv.) G.Don	Herb	Whole plant, leaves, flowers	Malnutrition, colds, ocular disorders, children fever	[77,89,134]
*Aneilema leiocaule* K.Schum.	Herb		Cough in children	[136]
*Aneilema spekei* C.B.Clarke	Herb	Whole plant, leaves	Fever, swollen organs, stomach ailments and emaciation in animals	[92,144]
*Commelina africana* L.	Herb	Leaves, exudate, whole plant	Stillbirth, fever, wounds, eyes, earache, colds, cough, sores, diseases of the ear, hemostatic when umbilical cord cut, immune booster	[14,99,105,115,136,140,141,144,145]
*Commelina albescens* Hassk.	Herb		Children’s fever	[77]
*Commelina benghalensis* L.	Herb	Leaves, exudate	Eye problems, children’s diseases, dislocations, injuries, cuts/wounds, colds, earache, eye infections, cough	[41,67,74,95,115,127,136,168]
*Commelina erecta* L.	Herb	Spathe	Sores	[140]
*Commelina foliacea* Chiov.	Herb		Children’s fever, cough in children	[77,136]
*Commelina imberbis* Ehrenb. ex Hassk.	Herb	Whole, flower	Children’s cough, fever	[72,99]
*Commelina ramulosa* (C.B.Clarke) H.Perrier (exotic)	Herb		Malaria	[124]
*Cyanotis foecunda* DC. ex Hassk.	Herb		Cough in children	[136]
*Cyanotis lanata* Benth.	Herb	Shoots, leaves	Ulcers in animals, knee problems, thinning in humans, fever, heart ailments	[144]
**Compositae**				
*Acanthospermum glabratum* (DC.) Wild (exotic)	Herb	Leaves	Broken limbs, boils	[66,144]
*Acmella caulirhiza* Delile	Herb	Leaves, flowers, aerial parts, whole plant, fruits, bark	Malaria, oral thrush, ulcers, nasal congestion, diarrhea, spinal cord problem, halitosis, toothache, afterbirth, oral cavity infections, diabetes, fever, cough, mouth ulcers, ringworms, wounds, ear discharge, craniotomy (surgery), gingivitis, bad smell, gum diseases, stomachache, sore throat, insect repellant, broken limps	[77,83,86,88,89,91,101,102,109,115,119,120,122,123,132,138,141,143,144,152,168]
*Ageratum conyzoides* L. (exotic)	Herb	Leaves, roots	Eye problems, mouth sores, tetanus, wounds, chest pains, rheumatism, malaria, stomachache, injury/cuts, bloody diarrhea, bronchitis, poisonous to livestock, hemostatic, stop epistaxis, sore eyes, bowel problems	[76,84,95,104,118,119,143,144,146,147,168]
*Artemisia abyssinica* Sch.Bip. ex A.Rich. (exotic)	Shrub	Leaves	Heavy menstruation (menorrhagia), livestock diseases, common colds	[86,87]
*Artemisia afra* Jacq. ex Willd.	Shrub	Whole plant, roots, leaves	ECF, malaria, anthelmintic, colds, influenza, fever, ear drop, abdominal pain, expectorant, cough, headache, loss of appetite	[99,104,105,120,132,141,147,168]
*Artemisia annua* L. (exotic)	Herb	Leaves	Asthma, malaria, cough	[14,41,98,104]
*Aspilia mossambicensis* (Oliv.) Wild	Herb/shrub	Roots, leaves, whole plant	Stomachache, cough, pneumonia, yellow fever, wounds, diarrhea, poison antidote, malaria, postpartum hemorrhage, prevent first/second trimester abortion, vomiting, pregnancy edema, sore eyes/gums, ulcers (animals), bums, eye diseases, anthrax, cuts, contraceptive, retained placenta	[14,74,80,81,104,140,143,145,147,151,168]
*Aspilia pluriseta* Schweinf. ex Engl.	Herb/shrub	Leaves, roots	Wounds, drippy nose in poultry, postpartum pain, headache, cuts, snakebites, diabetes mellitus, malaria, skin diseases, liver damage, anthelmintic, coagulant, anthrax, pimples, postpartum pains, gastrointestinal problems, joint diseases, eye diseases, contraceptive, stomachache, fever, edema	[51,79,85,96,104,106,113,115,119,125,143,144,149,168]
*Baccharoides adoensis* (Sch.Bip. ex Walp.) H.Rob.	Shrub	Roots, leaves, whole plant, bark	Eye infections, malaria, TB, gastrointestinal disorders, heart/kidney diseases, stomachache, acaricide, livestock diseases, anthelmintic, gonorrhea	[79,99,119,133,142,144]
*Baccharoides hymenolepis* (A.Rich.) Isawumi, El-Ghazaly & B.Nord.	Shrub	Roots, leaves	Stomachache, abdominal pains, purgative, infantile jaundice	[99,119,141,143]
*Baccharoides lasiopus* (O.Hoffm.) H.Rob.	Shrub	Leaves, roots, whole plant, bark, stems	Malaria, stomachache, shoulder pains, backache, pneumonia, scurvy, chest pains, burns, colds, spinal cord problem, typhoid, allergies, amoeba, asthma, cancer, dental problems, diabetes, heartburn, hypertension, inflammation, tonsils in cows, loose bladder, reduces addiction, reduces menstrual flow, rheumatism, skin diseases, STIs, stomach disorders, lungs, dog poison, acidity, nausea, sexual weakness, rashes, cough, diabetes, crop pests, prevent first/second trimester abortion, vomiting, colorectal cancer, postpartum pains, skin rashes, diarrhea, sore throat, acaricide, scabies, venereal diseases, maggot in sores, human/sheep anthelmintic, suppurating ear, skin diseases, appetizer, aphrodisiac, menstrual pains, fever	[41,67,74,81,83,84,99,103,104,106,109,115,117,118,129,131,133,138,142,143,144,145,146,147,152,168]
*Berkheya spekeana* Oliv.	Herb	Roots, leaves, whole plant	Arthritis, indigestion, amoeba, sores on lips/tongue, abdominal pains	[99,132,140,141,154]
*Bidens hildebrandtii* O.Hoffm.	Herb/shrub	Roots	Stomach disorders, malaria	[77]
*Bidens pilosa* L.	Herb	Leaves, roots, shoots, whole plant, flowers, aerial parts, stems	Eye problems, snakebites, aphrodisiac, infertility, skin infections, hemorrhage, stomachache, amoeba, back/joint/bone problems, cough, colds/flu, dizziness, ear-nose-throat problems, eye problems, heartburns, injuries/cuts/wounds, malaria, pneumonia, rheumatism, STIs, stomach disorders, ear complications, toothache, eye inflammation, malaria, ringworms, gonorrhea, sore eyes, postpartum hemorrhage, milk letdown, first/second trimester abortion, skin/throat cancer, anthelmintic, coagulant, diarrhea, stomach upsets, acaricide, rashes, diabetes, urinary disorders, liver/kidney problems, diabetes	[14,41,74,81,84,86,88,95,104,106,108,109,113,115,117,122,126,132,133,138,143,144,145,146,147,168]
*Blumea axillaris* (Lam.) DC.	Herb	Roots	Gonorrhea	[99]
*Bothriocline longipes* (Oliv. & Hiern) N.E.Br.	Shrub	Roots, leaves	Sores, sore throat, fever, malaria, strength and hygiene on babies	[67,136]
*Brachylaena huillensis* O.Hoffm.	Shrub/tree	Leaves, roots	Gastrointestinal complaints	[150]
*Carduus keniensis* R.E.Fr.	Herb	Roots	Spinal cord problems	[109]
*Carduus schimperi* Sch.Bip.	Herb	Whole plant	Arthritis	[154]
*Chrysanthemum indicum* L. (exotic)	Herb	Whole plant	Cough	[78]
*Cirsium vulgare* (Savi) Ten.	Herb	Leaves, twigs, whole plant	Arthritis, heartburn	[91,154]
*Conyza newii* Oliv. & Hiern	Shrub	Leaves, roots	Backache, pimples, expectorant	[77,109,141]
*Cotula anthemoides* L.	Herb	Leaves	HIV/AIDs, cough	[143]
*Crassocephalum montuosum* (S.Moore) Milne-Redh.	Herb	Roots, shoots	Infertility in women	[144]
*Crassocephalum picridifolium* (DC.) S.Moore	Herb	Roots	Stomachache	[144]
*Crassocephalum vitellinum* (Benth.) S.Moore	Herb	Flowers, leaves	Stomach complications, malaria, mouth infection in babies, remove particles from eyes, wounds/burns	[109,168]
*Crepis carbonaria* Sch.Bip.	Herb	Flowers	Assist in parturition	[67]
*Dichrocephala chrysanthemifolia* (Blume) DC.	Herb		Poisonous to cattle	[168]
*Dichrocephala integrifolia* (L.f.) Kuntze	Herb	Fruits, leaves, seeds	Oral cavity infections, memory loss, measles, skin growths, stomach ailments, diarrhea, head rashes	[119,123,144,147,154]
*Echinops amplexicaulis* Oliv.	Herb	Roots	Arthritis, chronic cough	[140,154]
*Echinops angustilobus* S.Moore	Herb	Roots	STIs	[120]
*Echinops hispidus* Fresen.	Herb	Roots	Chest congestion	[86]
*Emilia coccinea* (Sims) G.Don	Herb		Stomach problems	[115]
*Emilia discifolia* (Oliv.) C.Jeffrey	Herb	Roots, leaves, whole plant	Stomach ailments, constipation, nausea, amoeba, body aches, fatigue, malaria, clean womb, skin rashes, allergy, ringworm, peptic ulcers, pregnancy stomachache, aching bones, infantile diseases that causes disability, appetizer, anthelmintic, eye infection, chronic asthma, eye infection	[14,78,84,140,143,144,147]
*Erigeron bonariensis* L. (exotic)	Herb	Roots, leaves, stems	Toothache, snakebites, stomachache, back pain in women, postpartum pain, ringworms, wounds, headache, malaria, meningitis, arthritis, heartburn, oral thrush, gonorrhea, diarrhea, measles, stomach ailments, rashes, boils, itching, liver diseases, dysentery, anaplasmosis (cattle), heartburn, STIs, colds, throat/breast/squamous cell carcinoma of the gums, appetizer, tonsils, skin pimples, throat infections, backache, pimples, sore throat, postpartum pains, fever	[74,86,99,113,115,117,118,119,120,122,125,126,146,147,152,154,168]
*Erigeron canadensis* L. (exotic)	Herb		Toothache	[41]
*Erigeron karvinskianus* DC. (exotic)	Herb	Leaves	Heartburn, oral thrush, sore throat, cough	[86,144]
*Erythrocephalum marginatum* (O.Hoffm.) S.Ortiz & A.P.Cout.	Herb	Roots, leaves, whole plant	Restore penile erection and sexual vigor, liver pains, malaria	[96,106,127,129]
*Eschenbachia subscaposa* (O.Hoffm.) G.L.Nesom	Herb	Roots, leaves	Obesity, breast cancer, tonsils	[78]
*Ethulia scheffleri* S.Moore	Herb/shrub	Leaves	Malaria	[67]
*Ethulia vernonioides* (Schweinf.) M.G.Gilbert	Herb	Roots	Retained placenta	[67]
*Euryops jacksonii* S.Moore	Herb	Flowers	Assist childbirth	[67]
*Galinsoga parviflora* Cav. (exotic)	Herb	Leaves, roots	Wounds, tetanus, cough, colds, diabetes, colorectal, skin care cancer, skin sores/ulcers	[14,83,117,141,147]
*Gutenbergia cordifolia* Benth. ex Oliv.	Herb	Leaves, roots	Skin problems, oral cavity infections, stomachache, malaria, livestock diseases and hard dung, ticks, giardiasis, emetic, measles, eye ailments	[68,79,82,97,107,123,138,143,144,168]
*Gymnanthemum amygdalinum* (Delile) Sch.Bip.	Shrub/tree	Whole plant, roots, leaves	Abdominal pains, arthritis, meningitis, malaria, typhoid, epilepsy, diarrhea, stomachache, stiches, acaricide, gastrointestinal problems, typhoid, amoebiasis, teeth-problems, constipation, flatulence, menstrual problems, cuts, trematode/termite control	[64,76,79,87,101,116,132,133,144]
*Gymnanthemum auriculiferum* (Hiern) Isawumi	Shrub	Bark, roots, leaves	Stomachache, earache, chest pains, anthelmintic, menstrual pains, livestock diseases, heartburn, peptic ulcers, labor, malaria, pain in male private parts, after birth pains, amoebiasis, bilharzia, gastritis, measles, typhoid, cough, colds, mouth wounds, snakebites, eye problem, headache, influenza, kidney stones, wounds, venereal diseases, diarrhea in cattle	[41,88,103,104,119,120,132,144,147,152,168]
*Gymnanthemum myrianthum* (Hook.f.) H.Rob.	Shrub	Root, bark	Heartburn, oral thrush, impotence, anthrax, abdominal pains	[86,141]
*Gynura scandens* O.Hoffm.	Herb	Leaves	Fever	[144]
*Gynura valeriana* Oliv.	Herb	Whole plant	Fever, mouth rashes, wounds	[144]
*Helichrysum formosissimum* Sch.Bip.	Shrubs	Leaves	Sores on cattle	[67]
*Helichrysum forskahlii* (J.F.Gmel.) Hilliard & B.L.Burtt	Herb/shrub	Whole plant, roots	Nausea, vomiting, toothache, headache, stomachache	[71,99]
*Helichrysum globosum* Sch.Bip.	Herb	Roots	Expel placenta, chronic cough	[99,140]
*Helichrysum glumaceum* DC.	Herb	Roots	Pregnancy edema	[81]
*Helichrysum luteoalbum* (L.) Rchb. (exotic)	Herb	Stems, leaves	Tonic	[67]
*Helichrysum nudifolium* (L.) Less.	Herb	Leaves	Tonic, keep illness away	[67]
*Helichrysum odoratissimum* (L.) Sweet	Herb		Anthelminthic, cough, headache, wounds, repel fleas and lice	[115,168]
*Helichrysum schimperi* (Sch.Bip. ex A.Rich.) Moeser	Shrub	Leaves, roots, stems	Stomachache, wounds, colds, malaria, stomach disorders, fever, nausea, cough, inflammation	[99,109,131,152,168]
*Hirpicium diffusum* (O.Hoffm.) Roessler	Herb	Whole plant	Eye lotion	[77]
*Hoffmannanthus abbotianus* (O.Hoffm.) H.Rob., S.C.Keeley & Skvarla	Liana	Roots, stems, bark, leaves	Pneumonia, wounds, stomachache, cough, malaria, eye infections, stomach acidity, constipation, poisoning, first/second trimester abortion, vomiting, skin rashes, emetic, typhoid, joint pain/brucellosis/ back pain, fecal impaction in babies/children, anaplasmosis	[14,67,71,72,77,81,84,91,103,104,107,131,140,141,142,144,146,147,152,168]
*Jeffreycia hildebrandtii* (Vatke) H.Rob., S.C.Keeley & Skvarla	Herb/shrub	Roots	Convulsions, stomach trouble, snakebite antidote	[127,142]
*Jeffreycia usambarensis* (O.Hoffm.) H.Rob., S.C.Keeley & Skvarla	Shrub	Flowers	Cause eyes sores	[142]
*Kleinia petraea* (R.E.Fr.) C.Jeffrey	Herb	Whole plant, leaves	Abdominal pains, sore eyes	[141,168]
*Kleinia squarrosa* Cufod.	Shrub	Stems, aerial parts, bark, leaves	Asthma, amenorrhea, syphilis, jaundice, malaria, edema, cholera, thrombosis, second trimester abortion, pregnancy edema, measles, smallpox, diarrhea in poultry	[51,81,96,102,106]
*Lactuca inermis* Forssk.	Herb	Roots, whole plant, leaves	Joint pains, cough, stomachache, rheumatism, diabetes, heartburn, gonorrhea, syphilis	[74,83,99,130,147]
*Laggera brevipes* Oliv. & Hiern	Herb	Roots, leaves	Snakebite, measles, afterbirth pains, toothache, suppurating ears, baby skin care	[14,99,126,168]
*Laggera crispata* (Vahl) Hepper & J.R.I.Wood	Herb	Leaves, roots	Hiccups, colds, measles	[99,144]
*Laggera elatior* R.E.Fr.	Herb	Roots	Liver problems, gonorrhea, afterbirth pains, toothache, suppurating ears	[152,168]
*Launaea cornuta* (Hochst. ex Oliv. & Hiern) C.Jeffrey	Herb	Leaves, exudate, whole plant, leaves, shoots	*Chira*, chronic joint pains, malaria, amoebiasis, diabetes, appetizer, aphrodisiac, stomach disorders, typhoid, anthelmintic, chest pain, throat cancer, ulcers, dysentery, breast/prostate cancer, stomachache, warts, toothache, liver problems, coccidiosis in poultry, measles, stop sneezing, swollen testicles, smallpox, edema	[14,41,51,83,84,86,96,106,108,111,112,129,130,139,144,168]
*Linzia glabra* Steetz	Herb	Roots, leaves, whole plant	Snakebites, boils, stomach ailments, post/partum hemorrhage, first/second trimester abortion, diarrhea, gastrointestinal problems, stomach problems, menstrual problems, oral thrush, fever	[79,81,122,126,143,144,147]
*Lipotriche scandens* (Schumach. & Thonn.) Orchard	Shrub	Leaves	Teeth infections	[144]
*Microglossa densiflora* Hook.f.	Shrub	Leaves, roots, stems	Pneumonia, wounds, stomachache, menstrual pains, backache	[99,152]
*Microglossa pyrifolia* (Lam.) Kuntze	Shrub	Leaves, roots, whole plant	Malaria, boils, abscesses, arthritis, emetic, gum aches, eye lotion, skin rashes, measles, cough, nose bleeding, colorectal/skin/breast cancer, expel placenta, joints, wounds, toothache, acaricide, snakebites, gastrointestinal problems, promote fetal alignment or labor, colds, stomachache, anthrax, mental illness, skin infections, promote appetitepremature birth	[67,77,79,94,98,101,104,115,117,119,126,133,142,143,144,147,154,168]
*Microglossa pyrrhopappa* (Sch.Bip. ex A.Rich.) Agnew	Herb/shrub	Leaves, roots	Malaria, abdominal pains	[67,141]
*Mikania cordata* (Burm.f.) B.L.Rob. (exotic)	Herb	Leaves	Measles	[144]
*Orbivestus karaguensis* (Oliv. & Hiern) H.Rob.	Herb		Anthrax	[143]
*Gerbera piloselloides* (L.) Cass. (exotic)	Herb	Leaves	Tonic	[67]
*Pluchea dioscoridis* (L.) DC.	Shrub	Leaves	Infantile ailments, pregnancy pains	[142]
*Psiadia punctulata* Vatke	Shrub	Leaves, roots, bark, flowers	Abdominal pains, STIs, ectoparasites (ticks, fleas), insecticide, wounds, burns, malaria, scabies, skin infection, diarrhea, urinary problems, cough, sore throat, colds, afterbirth pains, inflammation, brucellosis, gonorrhea, edema, aphrodisiac, infertility, vertigo, abdominal pains, sexual stimulant, female conditions, headache, afterbirth pains	[68,71,72,76,77,91,97,99,105,107,113,115,140,141,142,143,144,168]
*Schkuhria pinnata* (Lam.) Kuntze ex Thell.	Herb	Leaves, whole plant	Malaria, joint pains, diabetes, fever, meningitis, stomach disorders, stomachache, colds/flu, wounds, gastrointestinal problems, plague, gingivitis, HIV/AIDs, abdominal pains during menstruation, vomiting	[14,41,76,79,84,103,104,115,118,129,130,132,143,147,153]
*Senecio hadiensis* Forssk.	Liana	Roots, leaves, stems	Malaria, diarrhea, myalgia, kidney diseases, blood cleanser, baby tonic, colds	[72,88,109,141,152]
*Senecio lyratus* Forssk.	Herb	Roots	Fever	[77]
*Senecio moore*i R.E.Fr.	Herb/shrub		Cough	[125]
*Senecio syringifolius* O.Hoffm.	Liana	Leaves, roots	Wounds, eye inflammation, stomachache, malaria	[111,152]
*Solanecio angulatus* (Vahl) C.Jeffrey	Herb	Roots, stems, leaves, bark	Mouth wash/teeth cleaning, livestock diseases, aphrodisiac, pneumonia, prostate cancer, STIs, whooping cough, backache, colds, gonorrhea	[41,77,107,135,147]
*Solanecio cydoniifolius* (O.Hoffm.) C.Jeffrey	Herb	Leaves	Skin rashes, joint pains	[77,119]
*Solanecio mannii* (Hook.f.) C.Jeffrey	Tree	Roots, leaves, fruits, whole plant	Snakebites, STIs, malaria, livestock, spinal cord problem, colds, chest infections, heartburn, dental problems, poison antidote, rheumatism, arthritis, sprains, boils, scurvy, wounds, stomach ailments, purgative, anaplasmosis (cow), trypanosomiasis (cow, goat), skin/breast/colorectal cancer, skin diseases, acaricide, sterility, itchy rash, abdominal pains, livestock fever, stomachache, cancer, pneumonia, cough, epilepsy, typhoid	[41,73,74,77,78,91,109,113,117,119,120,126,131,133,141,143,145,147,152,154,168]
*Solanecio nandensis* (S.Moore) C.Jeffrey	Liana	Stems, leaves	Malaria, stomachache, breast/colorectal cancer	[117,131]
*Sonchus asper* (L.) Hill	Herb	Roots, whole plant, leaves	Amoeba, diabetes, erectile dysfunction (impotence), tonsils, cough	[41,78,83,84]
*Sonchus luxurians* (R.E.Fr.) C.Jeffrey	Herb	Leaves, whole plant	Diabetes	[83]
*Sonchus oleraceus* L.	Herb	Roots, leaves	Gonorrhea, STIs, diabetes, typhoid, liver problems	[83,113,115,168]
*Sonchus schweinfurthii* Oliv. & Hiern	Herb	Leaves, roots	Gastrointestinal problems, sore throat, neck pains	[79,143,144]
*Sphaeranthus bullatus* Mattf.	Herb	Leaves, roots	Tonic, first/second trimester abortion, vomiting, pregnancy edema	[67,81]
*Sphaeranthus confertifolius* Robyns	Shrub	Fruits, leaves, roots, stems	Smoothen skin, skin conditions	[135]
*Sphaeranthus kirkii* Oliv. & Hiern	Herb	Leaves, aerial parts	Swollen legs during pregnancy, malaria, edema	[51,139]
*Sphaeranthus steetzii* Oliv. & Hiern	Herb	Leaves, whole plant	Medicine for pregnancy, diarrhea, cough, inflammation, difficulty in breathing, skin ulcers/rashes, madness	[77,122,140,144]
*Sphaeranthus suaveolens* DC.	Herb	Leaves, flowers, whole plant, shoots, fruits	Postpartum pain, nasal congestion, malaria, retained afterbirth, stomachache, rheumatism, cough, sore throat, flu, eye ailments	[67,103,104,113,144,152,168]
*Sphaeranthus ukambensis* Vatke & O.Hoffm.	Herb	Stems, roots	Food poisoning, stomachache, baby fever	[91,139]
*Tagetes minuta* L. (exotic)	Herb	Bark, leaves, whole plant, roots, flowers, shoots	Fever, infertility, polio, muscle ache, nose bleeding, malaria, meningitis, amoeba, back/joint/bone problems, cough, colds/flu, dental problems, diabetes, headache, herbicide, injuries/cuts, wounds, skin diseases, TB, arthritis, asthma, toothache, control cockroaches/fleas, mosquito/ant repellant, ectoparasites (cow, poultry), skin diseases, stomachache, toothache, abdominal pains, ulcers	[41,66,73,76,77,78,109,113,115,118,119,120,122,129,133,141,143,144,146,147,152,154,168]
*Tanacetum cinerariifolium* (Trevis.) Sch.Bip. (exotic)	Herb	Flower, leaves, stems, aerial parts	General ectoparasites i.e., mites, lice, flea infestation (cow, poultry), tick infestation (cow, poultry, goat, sheep, pig)	[73,133]
*Taraxacum* sect. Taraxacum F.H.Wigg. (exotic)	Herb	Roots, shoots	Barrenness, afterbirth pains	[144]
*Tarchonanthus camphoratus* L.	Shrub/tree	Leaves, stems, roots, bark	General body fatigue, wounds, skin infections, asthma, cough, bronchitis, fever, constipation, gastrointestinal diseases, vitamin, nausea, headache, chest diseases	[71,99,107,109,140,152,168]
*Tithonia diversifolia* (Hemsl.) A.Gray (exotic)	Shrub	Leaves, roots, bark, whole plant, flowers, stems	Indigestion, sprains, livestock diseases, malaria, snakebites, stomachache, headache, joint pains, typhoid, amoebiasis, contagious diseases, cough, colds/flu, insecticides, chicken diseases, pesticide, pneumonia, stomach disorders, anthelmintic, herbicide, diabetes mellitus, sore throat, liver problems, livestock weakness, ectoparasites (cow), helminthiasis (cow, poultry, goat, sheep, pig), tonsils, jiggers, acaricide, anaplasmosis, pimples, wounds, gastrointestinal problems, asthma, skin diseases, venereal diseases	[41,66,73,79,82,84,87,98,101,104,106,108,115,118,119,122,126,129,130,133,143,144,146,168]
*Tridax procumbens* L. (exotic)	Herb	Leaves, stems	Dental problems, injuries/cuts, wounds, malaria, stomachache, toothache	[14,41,51,67,99,147]
*Vernonia colorata* (Willd.) Drake	Shrub	Roots	Anthelmintic, cough, pneumonia, stomachache	[74]
*Vernonia galamensis* (Cass.) Less.	Herb	Leaves	Eye lotion, eye problems	[77,136]
*Vernonia holstii* O.Hoffm.	Herb/shrub	Roots	Abdominal pains, tonsils	[99,100]
**Connaraceae**				
*Agelaea pentagyna* (Lam.) Baill.	Shrub/liana	Roots	Impotence	[144]
**Convolvulaceae**				
*Astripomoea grantii* (Rendle) Verdc.	Herb	Leaves	Chronic headache	[144]
*Astripomoea hyoscyamoides* (Vatke) Verdc.	Herb	Leaves	Typhoid	[144]
*Astripomoea malvacea* (Klotzsch) A.Meeuse	Herb	Roots	Malaria, stomach dysentery, constipation	[74]
*Cuscuta australis* R.Br.	Herb	Whole plant	Typhoid, diarrhea	[119]
*Cuscuta campestris* Yunck.	Herb	Exudate	Scabies, pimples, warts, boils	[168]
*Cuscuta kilimanjari* Oliv.	Herb	Bark, whole plant	Pregnancy edema, stomach pains	[81,84]
*Dichondra repens* J.R.Forst. & G.Forst. (exotic)	Herb	Leaves	Snakebites, skin wounds, ringworms	[118,126]
*Evolvulus alsinoides* (L.) L.	Herb	Leaves, roots	Diarrhea, wounds, oral thrush	[76,143]
*Hewittia malabarica* (L.) Suresh	Herb	Roots	Stomach swellings, constipation, pain	[144]
*Hildebrandtia obcordata* S.Moore	Shrub	Roots	Constipation	[142]
*Hildebrandtia sepalosa* Rendle	Shrub	Roots	Stomach problems, upper respiratory tract infections, gastrointestinal complications	[72,116]
*Ipomoea batatas* (L.) Lam. (exotic)	Herb	Leaves, stems, exudate	Wounds from poisoned arrows, acaricide	[133,147]
*Ipomoea cairica* (L.) Sweet	Herb	Leaves, roots	Breast/cervical/skin cancer, colds, measles	[117,119]
*Ipomoea hildebrandtii* Vatke	Shrub	Exudate	Fungal infections	[143]
*Ipomoea jaegeri* Pilg.	Shrub	Roots	Emetic, gonorrhea	[67,77]
*Ipomoea kituiensis* Vatke	Shrub	Leaves, roots, stems	Stomachache, typhoid, constipation, digestive disorders, cough, remove thorn from flesh, snake poison	[41,76,80,98,100,108,142]
*Ipomoea lapidosa* Vatke	Liana	Twigs, roots, leaves, stems	Ocular disorders, toothache, paresthesia, blood cleanser, STIs	[88,91]
*Ipomoea longituba* Hallier f.	Shrub	Roots	Kwashiorkor	[138]
*Ipomoea obscura* (L.) Ker Gawl.	Herb	Leaves	Anthrax	[147]
*Ipomoea spathulata* Hallier f.	Shrub	Roots, exudates, leaves	Eyes problems, eye lotion, ringworms, diarrhea, tonic, infertility	[72,76,77,99,136,141]
*Ipomoea tenuirostris* Choisy	Herb	Leaves	Rheumatism	[99,141]
*Ipomoea wightii* (Wall.) Choisy	Herb	Exudate	Cuts/wounds	[152]
**Cornaceae**				
*Cornus volkensii* Harms	Tree		Typhoid, amoeba, stomachache, vomiting	[132]
**Crassulaceae**				
*Cotyledon barbeyi* Schweinf. ex Baker	Herb	Leaves	Teeth problem, skin infections, stomach complications, spinal cord problem, painful parts of the body, fatigue pains, rheumatism	[67,109,142]
*Crassula granvikii* Mildbr.	Herb	Shoots, leaves	Rheumatism and thinning, black water, dermatitis, increase blood, swelling of stomach/legs/whole body	[144]
*Crassula schimperi* Fisch. & C.A.Mey.	Herb		Wounds	[136]
*Kalanchoe crenata* (Andrews) Haw.	Herb	Leaves, exudate	Navel infections in children, swollen areas for muscle sprain and myalgia, stomach problems, bruises, wounds, umbilical cord problem, rashes, disinfectant	[87,89,109,132]
*Kalanchoe densiflora* Rolfe	Herb	Leaves, roots, stems	Eye problems, joint pains, stomachache, umbilical cord, wounds, muscle pains, fast heartbeat, heartburn, heartache, skin problems, rheumatism, muscular/bone dislocations, dermatitis, arthritis, dislocated joints, stiff/swollen muscles, liver problems, boils, whitlow, edema, poisonous, swollen body, abortifacient	[14,67,72,94,95,119,131,136,143,144,152,154,168]
*Kalanchoe glaucescens* Britten	Herb	Leaves	Rheumatism	[67]
*Kalanchoe lanceolata* (Forssk.) Pers.	Herb	Leaves, flowers	Splenomegaly, hepatomegaly, blood cleanser, rheumatism	[67,88]
*Kalanchoe marmorata* Baker	Herb	Leaves	Stiff muscles, liver problems, skin diseases, diarrhea and foot rot in sheep	[168]
*Kalanchoe obtusa* Engl.	Herb	Whole plant	Children’s diseases, pesticide	[127]
*Kalanchoe prittwitzii* Engl.	Herb	Leaves	Stiff joints, rheumatism	[67]
**Cucurbitaceae**				
*Citrullus lanatus* (Thunb.) Matsum. & Nakai	Herb		Cleanse blood	[41]
*Coccinia grandis* (L.) Voigt	Herb	Leaves, whole plant	Diarrhea, snoring, abdominal pains	[76,91,143]
*Cucumis aculeatus* Cogn.	Herb	Leaves, fruits, exudate	Malaria, livestock diseases, anaplasmosis (cow, ruminant), helminthiasis (cow, poultry, goat, sheep, pig), loss of feathers (poultry), cough, ECF, emetic, nausea, appetite	[14,67,73,101,114,115,140]
*Cucumis dipsaceus* Ehrenb. ex Spach	Herb	Fruits, roots, leaves	Abdominal pains, acidity, emetic, gastrointestinal problems, emetic, hasten delivery, cathartic, hemorrhoids	[79,86,91,143,147]
*Cucumis engleri* (Gilg) Ghebret. & Thulin	Herb	Roots	Malaria	[67]
*Cucumis ficifolius* A.Rich.	Herb	Leaves, roots, bark, whole plant	Malaria, stomach complications, *Chira*, constipation, emetic, abdominal pains, bloody stool	[14,108,109,114,119,141,168]
*Cucumis prophetarum* L.	Herb	Fruits, roots	Emetic, remove phlegm, malaria, infertility, infected ears, syphilis	[86,87,114]
*Cucurbita ficifolia* Bouché (exotic)	Herb	Leaves	Mouth ulcers	[122]
*Cucurbita maxima* Duchesne (exotic)	Herb	Fruits	Cough, AIDs, amoeba, cancer, hard stool, immunity booster, operated mother, prostrate problems, skin diseases, snakebites, stomach disorders, anthelmintic, urinary disorders, aphrodisiac	[41,96,168]
*Cucurbita pepo* L. (exotic)	Herb	Roots, seeds	Afterbirth, anthelmintic	[120]
*Gerrardanthus lobatus* (Cogn.) C.Jeffrey	Herb	Roots	Malaria	[111]
*Kedrostis foetidissima* (Jacq.) Cogn.	Herb	Roots	Smallpox, bloat in livestock, measles, boils, whitlow	[114,121,143]
*Kedrostis gijef* (Forssk. ex J.F.Gmel.) C.Jeffrey	Liana	Roots	Eye medicine, snake poison, malaria, cough, stomachache	[72,114,142]
*Kedrostis heterophylla* Zimm.	Liana	Leaves	Children’s diseases	[127]
*Kedrostis pseudogijef* (Gilg) C.Jeffrey	Liana	Leaves, bark, exudate, stems	Diarrhea, yellow fever, malaria, hepatitis, measles, cuts	[51,77]
*Lagenaria abyssinica* (Hook.f.) C.Jeffrey	Herb	Leaves, exudate	Wounds, toothache	[114,152]
*Lagenaria siceraria* (Molina) Standl.	Herb	Leaves	Cough	[87]
*Lagenaria sphaerica* (Sond.) Naudin	Herb	Flowers, roots	Scabies, candidiasis, ringworms, leprosy, retained placenta, abdominal pains	[114,131]
*Momordica anigosantha* Hook.f.	Herb	Fruits	Vomiting, emetic	[132,141]
*Momordica boivinii* Baill.	Herb	Roots	Induce menstruation	[114]
*Momordica foetida* Schumach.	Herb	Roots, whole plant, aerial parts, fruits, leaves, exudate	Trigeminal neuralgia, sinusitis, wounds, teething problems, abdominal pains, amoeba, fever, arthritis, heartburn, oral thrush, colic pain in infants, emetic, diabetes mellitus, skin rashes, fever, stomachache, malaria, anthelmintic, cough (livestock, human), breast/cervical cancer, poisonous, chest problems, headache, typhoid	[82,85,86,87,88,109,114,117,118,120,132,154]
*Momordica friesiorum* (Harms) C.Jeffrey	Herb	Stem, bark, leaves, roots, seeds	Malaria, livestock diseases, emetic, spleen, liver diseases, vomiting	[67,88,89,114,152]
*Momordica pterocarpa* Hochst. ex A.Rich.	Herb		Ear infection, malaria	[114]
*Momordica rostrata* Zimm.	Herb	Roots, leaves, fruits, shoots	Malaria, brain stimulant, emetic, laxative, swollen legs and body pains during pregnancy, retained placenta, abdominal pains, fever, burns, stomachache, coccidiosis (poultry), measles, trematodes, cuts, boils of livestock	[114,131,139,144]
*Momordica spinosa* (Gilg) Chiov.	Shrub/liana	Roots, bark	Headache, hepatitis, respiratory diseases, fever, malaria	[72,137]
*Peponium vogelii* (Hook.f.) Engl.	Herb	Leaves, fruits, whole plant	Fever, dysmenorrhea, venereal diseases, headache, rash	[77,114]
*Zehneria pallidinervia* (Harms) C.Jeffrey	Herb	Leaves	Snakebites	[127]
*Zehneria scabra* (L.f.) Sond.	Herb	Stems, leaves, seeds, roots, shoots, branches	Malaria, asthma, boils, wounds, constipation, livestock diseases, skin disorders, ringworms, gonorrhea, measles, colds, flu, hypertension, eye infections, diarrhea, vomiting, abdominal pains, skin rashes, cough, cancer, recuperate patients, eye/ear problems, warts, cuts	[41,67,76,86,91,99,105,107,109,114,115,131,132,138,147,152,168]
**Cupressaceae**				
*Cupressus torulosa* D.Don ex Lamb. (exotic)	Tree	Leaves, bark	Colds, urinary bladder inflammation	[152]
*Hesperocyparis lusitanica* (Mill.) Bartel (exotic)	Tree	Leaves, exudate, fruit, bark, stems	Cancer, epilepsy, rheumatism, skin diseases, typhoid, toothache, anthelmintic, measles, dysentery, acaricide	[41,116,133]
*Juniperus procera* Hochst. ex Endl. (exotic)	Tree	Leaves, bark, branches, exudates, roots	Eye problems, whopping cough, stomachache, livestock diseases, cancer, wounds, breathing difficulty, amoeba, cough, colds/flu, diarrhea, gall bladder, kidney problems, knock swells, malaria, placenta removal, rheumatism, typhoid, anthelmintic, broken bones, sore throat, breast/throat/squamous carcinoma of the gum, acaricide, aphrodisiac, expectorant	[41,64,72,86,88,95,117,120,132,133,136,140,141,145,152,168]
**Cyperaceae**				
*Cyperus alternifolius* L.	Herb	Roots	Boils, rashes	[80]
*Cyperus articulatus* L.	Herb	Roots	Malaria, body weakness, fever, pneumonia, hiccups	[103,104,115,128,144,147]
*Cyperus brevifolius* (Rottb.) Hassk.	Herb		Fever	[136]
*Cyperus dichrostachyus* Hochst. ex A.Rich.	Herb		Fever	[136]
*Cyperus elegantulus* Steud.	Herb		Fever	[136]
*Cyperus esculentus* L.	Herb	Leaves, roots	Abdominal upsets, bad breadth, stomachache, abdominal/colic pains, eye problems, typhoid, cough, blindness, backache, headache, chest problem, gingivitis, appetizer	[88,91,132]
*Cyperus impubes* Steud.	Herb		Fever	[136]
*Cyperus laevigatus* L.	Herb		Chickenpox, malaria, stomach disorders	[41]
*Cyperus niveus* Retz.	Herb		Fever	[136]
*Cyperus papyrus* L.	Herb	Leaves, roots	Pneumonia, headache, malaria, hypertension, nutritional edema, HIV/AIDs	[74,143,168]
*Cyperus rigidifolius* Steud.	Herb		Fever	[136]
*Cyperus rotundus* L.	Herb	Leaves	Heart problems	[144]
**Dichapetalaceae**				
*Dichapetalum ruhlandii* Engl.	Shrub/liana		Poisonous	[142]
*Dichapetalum zenkeri* Engl.	Shrub/tree	Roots	Venereal diseases, menstrual disorders, prenatal care	[127]
**Dilleniaceae**				
*Tetracera boiviniana* Baill.	Shrub	Roots	Stomach ailments, venereal diseases	[127]
**Dioscoreaceae**				
*Dioscorea minutiflora* Engl. (exotic)	Herb	Tubers, exudate	Blood cleanser, viral diseases, rheumatism, heartburn, gonorrhea, warts	[41,84,147,168]
*Dioscorea praehensilis* Benth.	Herb	Fruits	Measles	[144]
*Dioscorea quartiniana* A.Rich.	Herb	Roots	Poisonous, fever	[77,99]
**Ebenaceae**				
*Diospyros abyssinica* (Hiern) F.White	Shrub/tree	Seeds, bark, roots, twigs	Diarrhea, sores, laxative, malaria, ringworms, skin rashes, internal injuries, ocular disorders in livestock, STIs, venereal diseases	[66,89,116,120,125,144]
*Diospyros consolatae* Chiov.	Shrub/tree	Roots, leaves, back	Toothache, stomach ailments, skin diseases, respiratory problems, kidney problem, gastrointestinal gas, snakebites, arthritis, umbilical cord, hernia, veterinary medicine	[127,150]
*Diospyros loureiroana* G.Don	Shrub/tree		Snakebites	[134]
*Diospyros scabra* (Chiov.) Cufod.	Shrub/tree	Bark	Women’s stomach troubles, fever in children	[91,142]
*Diospyros squarrosa* Klotzsch	Shrub/tree	Roots	Prenatal care, septic swelling, convulsions	[127]
*Euclea divinorum* Hiern	Shrub/tree	Stems, bark, leaves, roots, seeds, fruits	Sore throat, cathartic, malaria, constipation, excessive menstrual flow, anthelmintic, diarrhea, infertility, spinal cord problem, abdominal upsets, skin disorders, blood cleansers, invigorant, prophylaxis of cancer and respiratory disorders, stomachache, amoeba, boils, skin rashes, snakebites, dental problems, dislocation, indigestions, livestock diseases, heartburn, toothache, cough, tonic, diabetes mellitus, anthrax, rheumatism, stomach rumblings, cleans the womb, headache, appetizer, joint pains in livestock, fever, venereal diseases, protracted labor, postpartum hemorrhage, retained afterbirth, anaplasmosis (cow, ruminant), constipation (cow, goat, sheep), sore throat, pneumonia, chest pains, acaricide, problems during pregnancy, arthritis, leprosy, asthma, joint diseases, bleeding gums, loose teeth, dysentery, menstrual pains, bloat, laxative, ulcers, wounds, gonorrhea, purgative, tonic for anemia	[14,41,66,67,68,71,72,73,74,79,80,81,82,85,86,87,88,91,98,99,100,104,105,107,108,109,112,113,115,116,120,124,132,133,135,138,141,142,143,146,147,149,154,168]
*Euclea natalensis* A.DC.	Shrub/tree	Roots	Diarrhea, poisoning, snakebites, diabetes mellitus, anthelmintic, mouth infections, stomach ulcers, gonorrhea, constipation, vaginal discharge, toothache, earache	[85,99,127,150]
*Euclea racemosa* L.	Shrub/tree	Root, bark, leaves	Poisoning, diabetes mellitus, liver, anthelmintic, stomach disorders, *Chira*, bloody diarrhea, stomachache, constipation, purgative, colic pain, general body wellness	[14,76,77,85,127,140,142]
**Ericaceae**				
*Agarista salicifolia* (Lam.) G.Don	Shrub/tree	Leaves, bark	Digestion, indigestion, poisonous	[99,142]
**Euphorbiaceae**				
*Acalypha fruticosa* Forssk.	Shrub	Roots, leaves	Snakebites, induce labor pains, liver, wounds, scorpion/bee stings, fever in children, cerebral malaria, headache, gastrointestinal gas, gonorrhea, gastrointestinal problems	[64,77,91,100,127,142,143,150]
*Acalypha paniculata* Miq.	Herb/shrub	Roots, stems, bark	Acaricide	[133]
*Acalypha psilostachya* Hochst. ex A.Rich.	Herb	Roots	Retained placenta	[144]
*Acalypha volkensii* Pax	Shrub	Leaves, roots	Gonorrhea, sores, fatigue, dizziness	[67,144,168]
*Croton alienus* Pax	Shrub/tree	Roots	General health, rheumatism	[168]
*Croton dichogamus* Pax	Shrub/tree	Roots, bark, leaves, seeds, stems	Gonorrhea, arthritis, polio symptoms, chest pain, tonic, malaria, bone/joint pain, respiratory diseases, fever, abdominal pain, oral thrush, chest congestion, asthma, cough, colds, rheumatism, joint diseases, miscarriage, swollen legs and body pains during pregnancy, back pains, stomachache, edema, infertility	[14,51,67,71,72,77,84,89,91,98,105,107,129,136,137,139,142,143,149,168]
*Croton macrostachyus* Hochst. ex Delile	Tree	Roots, bark, leaves, seeds, stems, exudate	Eye problems, sore throat, flu, TB, stomachache, measles, dysentery, malaria, respiratory disorders, ear infection, allergies, ringworms, dysmenorrhea, diarrhea, fever in cows, typhoid, measles, acaricide, asthma, bites, cough, colds/flu, dental problems, general body pains, heart problems, increase body temperatures, injuries/cuts, wounds, livestock (pregnancy test in cows), measles, mumps, pneumonia, rheumatism, skin diseases, STIs, straighten muscles, thorn pricks, anthelmintic, oral cavity infections, cleansing digestive and blood circulation system, diabetes mellitus, amoeba, venereal diseases, backache, fatigue, postpartum hemorrhage, colorectal/skin/breast, sore throat, flu, dysentery, asthma, headache, rashes, purgative, aphrodisiac, cathartic, food poisoning, asthenia, warts, fever in livestock/man	[14,41,66,67,81,84,85,87,88,95,99,101,104,116,117,119,120,123,132,133,141,142,143,144,145,146,147,153,168]
*Croton megalocarpus* Hutch.	Tree	Roots, bark, leaves	Stomachache, malaria, respiratory conditions, back pain, amoeba, joint/bone problems, body weakness, chest congestions, chest problems, cough, colds/flu, dental problems, general body pains, headache, injuries/cuts, wounds, livestock diseases, pneumonia, rheumatism, STIs, stomach acid, body swellings, typhoid, ulcers, anthelmintic, kidney failure, lung problems, cancer, toothache, diabetes mellitus, fever, diarrhea, anaplasmosis, diarrhea/dysentery (cow, poultry, goat, sheep, pig), paralysis/stroke, abortifacients, purgative, acaricide, boils, pneumonia, migraine, diaphragm, throat infection, swollen legs and body pains during pregnancy, retained placenta, abdominal pains, constipation, liver disease in poultry, aphrodisiac	[14,41,51,64,68,72,73,74,77,85,91,92,98,99,105,106,107,113,116,121,122,133,136,139,140,145,146,151,168]
*Croton menyharthii* Pax	Shrub	Leaves, roots	Influenza, malaria, pregnancy pains, menstrual pains, muscle spasms, female reproductive disorders, vaginal discharge, ulcers, respiratory problem	[67,100,142,150]
*Croton pseudopulchellus* Pax	Shrub/tree	Roots, leaves	Convulsions, gastric lesions, inflammation, convulsions, snakebite, rheumatism	[127,150]
*Croton scheffleri* Pax	Shrub	Roots	Fever, malaria	[77]
*Croton somalensis* Pax	Tree	Stem, bark, leaves, roots	Influenza, malaria, stomachache	[107,142]
*Croton sylvaticus* Hochst.	Shrub	Roots, bark, buds, seeds	Acaricide, malaria, stomach disorders, respiratory infections, wounds, tonsillitis, colds, stomachache	[14,133,134,142]
*Croton talaeporos* Radcl.-Sm.	Shrub/tree	Roots, leaves	General body pains, inflammation, colds, stomach complaints, typhoid, hypertension, rheumatism	[127,142,150]
*Erythrococca atrovirens* (Pax) Prain	Shrub		Septic wounds	[125]
*Erythrococca bongensis* Pax	Shrub/tree	Leaves, roots, stems	Arthritis, livestock diseases, stomachache, *Chira*, acaricide, livestock diseases, dizziness, insect bites, ECF in cattle, heartburn, backache, gynecological problems, HIV/AIDs, anthelmintic	[66,67,76,77,133,140,142,143,168]
*Erythrococca fischeri* Pax	Shrub/tree	Roots	Infertility, gonorrhea, chest pains	[99]
*Euphorbia abyssinica* J.F.Gmel.	Tree	Branches	Stomach pains in childbirth	[67]
*Euphorbia bicompacta* Bruyns	Shrub/tree	Leaves, exudate	Eye problems, livestock diseases, tonsils, ear complication, wounds, retained placenta in cattle, ECF in cattle, poison, muscle pull, prevent termites, bloat, edema in cattle, swollen glands in calves, asthma	[14,41,73,100,106,142,146,168]
*Euphorbia bussei* Pax	Tree	Branches	Sore throat, flu, asthma	[100]
*Euphorbia candelabrum* Trémaux ex Kotschy	Tree	Bark, roots, latex, leaves, stems	Venereal diseases, infertility, gonorrhea, flu, joint pains, STIs, eye problems, malaria, retained placenta, chest problems, bronchitis, headache, barrenness, upper respiratory tract infections, gastrointestinal complications, wounds, coenuruses, ECF in cows, stomach diseases, acaricide, poison, backache, mumps, blood pressure, menstrual cramps, rash, asthma, bronchial conditions, hernia, leprosy, lumpy skin disease in cows	[41,67,68,71,72,73,77,100,105,107,120,132,133,135,138,142,145,150,168]
*Euphorbia crotonoides* Boiss.	Herb	Exudate	Skin ulcers, warts	[51,140]
*Euphorbia cryptospinosa* P.R.O.Bally	Shrub	Exudate	Poisonous	[142]
*Euphorbia cuneata* Vahl	Shrub/tree	Roots, aerial parts, exudate	Gonorrhea, constipation, increase fertility, dust in eyes, hepatitis in calves	[77,80,105]
*Euphorbia engleri* Pax	Shrub/tree		Poisonous	[142]
*Euphorbia friesiorum* (A.Hässl.) S.Carter	Shrub/tree	Exudate, bark	Poisonous, elephantiasis, blindness	[84,142,168]
*Euphorbia gossypina* Pax	Shrub	Stems, roots, exudate	Warts (verrucae), ringworms, poisonous, swollen legs and body pains during pregnancy, fever	[80,94,139,142]
*Euphorbia heterochroma* Pax	Shrub	Roots, stems, exudate, whole plant	Malaria, gonorrhea, fever, gland complications, respiratory diseases, ticks, ectoparasites, pneumonia, tuberculosis, acaricide, gonorrhea, chest problems, virus diseases in chicken, HIV/AIDs	[68,72,73,77,100,136,137,145]
*Euphorbia heterophylla* L.	Herb	Leaves	*Chira*, increase letdown in livestock	[108,144]
*Euphorbia hirta* L.	Herb	Leaves, roots, whole plant, exudate	Skin infections, stomach problems, chest problems, asthma, respiratory and urinary disorders, diarrhea, skin diseases, male impotency, lactating mothers/cows, rashes	[109,119,144,168]
*Euphorbia inaequilatera* Sond.	Herb	Leaves, roots, exudate, whole plant, stems	Gonorrhea, stimulant, to allay hunger, prevent sleep, wounds, burns, skin ulcers, glaucoma, snakebite, pregnancy problems	[67,77,143,144]
*Euphorbia joyae* P.R.O.Bally & S.Carter	Shrub	Roots, stems	Postpartum hemorrhage, cough	[113,142]
*Euphorbia meridionalis* P.R.O.Bally & S.Carter	Herb	Stems	Malaria, STIs, induce diarrhea as a means of cleansing the body	[92]
*Euphorbia milii* Des Moul. (exotic)	Shrub	Fruits, leaves, exudate	Skin infections, warts	[74,146]
*Euphorbia nyikae* Pax ex Engl.	Shrub/tree	Latex	Septic swelling, malaria, wounds/burns	[127,136]
*Euphorbia pereskiifolia* Houllet ex Baill.	Shrub/tree	Latex	Fish poison	[142]
*Euphorbia quinquecostata* Volkens	Tree		Insecticide in grain stores	[100]
*Euphorbia scheffleri* Pax	Shrub	Stems, exudate, roots, fruits	Vomiting, toothache, goat medicine, poisonous, stomachache, cuts	[51,77,81,100,138]
*Euphorbia schimperiana* Scheele	Herb	Shoots, exudate, leaves, branches	Coughs, colds, poisonous,increase lactation in animals	[67,99,144,168]
*Euphorbia systyloides* Pax	Herb		Wounds	[72]
*Euphorbia tenuispinosa* Gilli	Shrub	Stems	Poison, diarrhea in chicken/goats	[100,142]
*Euphorbia tirucalli* L.	Shrub/tree	Stems, bark, exudate, branches	Eye infection, warts (verrucae), cancer, eye problems, injuries/cuts/wounds, livestock diseases, malaria, STIs, stomach disorders, typhoid, epilepsy, toothaches, pesticide in stored grains, women’s health, acaricide, poisonous, infertility, relieves pain and prevents infection to sores, eye infection, swollen glands, poisonous to eyes, sore throat, measles, removing specks from eyes, pesticidal (aphids)	[14,41,66,86,94,100,116,127,133,137,141,142,143,145,147,168]
*Euphorbia transvaalensis* Schltr.	Herb	Latex	Warts	[77]
*Euphorbia ugandensis* Pax & K.Hoffm.	Herb/shrub		Colds, cough	[142]
*Euphorbia uhligiana* Pax	Herb	Branches, fruits, roots, whole plant	Wounds, colds, stomach disorders, hypertension	[51,72,135]
*Euphorbia umbellata* (Pax) Bruyns	Shrub/tree	Leaves, latex	Poisonous, backache, joint swelling, sore throat, respiratory problems, fungal skin disease, boils (cattle)	[142,143]
*Euphorbia wakefieldii* N.E.Br.	Tree	Stems	Postnatal abdominal pains	[127]
*Excoecaria madagascariensis* (Baill.) Müll.Arg.	Shrub/tree	Bark, latex	Poisonous	[134,142]
*Jatropha curcas* L. (exotic)	Shrub/tree	Leaves, roots, fruits, leaves	Burns, injuries/cuts, wounds, snakebites, stomach disorders, mouth blisters, constipation, lice/flea control, miscarriage, diarrhea, kwashiorkor	[41,74,106,138,143,146,147,150]
*Jatropha dichtar* J.F.Macbr.	Shrub	Root, bark, exudates	Stomach, chest problems, eyedrops, emetic	[72,142]
*Jatropha ellenbeckii* Pax	Shrub	Exudates	Wounds	[142]
*Jatropha podagrica* Hook. (exotic)	Shrub	Leaves	Chest problems, fever	
*Jatropha spicata* Pax	Herb/shrub	Leaves, stems	Laxative, diarrhea	[100,142]
*Jatropha velutina* Pax & K.Hoffm.	Herb	Leaves, exudates	Wounds	[100,120]
*Macaranga capensis* (Baill.) Sim	Tree	Bark	Diarrhea	[122]
*Macaranga kilimandscharica* Pax	Tree	Roots, leaves, stems	Bilharzia, cough, stomachache, cancer, acaricide	[41,99,131,133]
*Mallotus oppositifolius* (Geiseler) Müll.Arg.	Shrub/tree	Roots	Chest pains	[142]
*Manihot esculenta* Crantz (exotic)	Herb/shrub	Root, bark, stems, leaves	Foot and mouth disease, acaricide, aphrodisiac, abdominal pains	[14,96,133,139]
*Neoboutonia macrocalyx* Pax	Tree	Bark, exudate	Warts, malaria	[93,103,104,168]
*Neoboutonia melleri* (Müll.Arg.) Prain	Shrub/tree	Stems, roots	Acaricide, poisonous	[133,142]
*Ricinus communis* L.	Herb	Leaves, roots, seeds, bark	Ruminal impaction, constipation, infertility, regulate menstrual cycle, chest pains, stomachache (after-birth), constipation, livestock diseases, snakebites, septic swelling, laxative, joint pains, burns, diarrhea, vomiting, stomachache, arthritis, bites, blood cleanser, burns, cough, colds/flu, dental problems, detoxifier, family planning, hard stool, indigestion, injuries/cuts, wounds, labor pains, aphrodisiac, pimples, pneumonia, rheumatism, stomach disorders, laxative, umbilical cord infection, dry skin, ringworms, asthma, whooping, malaria, fevers, retained afterbirth, protracted labor, postpartum hemorrhage, ECF, bloat (cow), skin rushes, urinary problems, enhance appetite, abdominal pains, syphilis, ulcers, acaricide, eye ailments (cloudy eyes), STIs, emetic, poisonous, smooth skin, ease parturition, scorpion bites, earache, venereal diseases, gonorrhea, bilharzia, earache, pinworms	[14,41,51,64,67,68,73,74,77,81,86,91,94,96,99,105,106,111,113,115,118,119,120,122,127,130,132,133,134,139,141,142,143,144,145,147,151,152,168]
*Shirakiopsis elliptica* (Hochst.) Esser	Tree	Bark, leaves, seeds	Eye injury/infection in livestock, dental problems, malaria, colorectal/esophageal cancer, burns, asthma, acaricide, man/animal fattening	[41,66,116,117,119,125,133,144]
*Spirostachys africana* Sond.	Tree	Roots, bark, stems	Antiseptic, congestion, breast swelling	[100,127]
*Spirostachys venenifera* (Pax) Pax	Tree	Stems, leaves, branches	Poisonous, livestock boils	[14,142]
*Suregada zanzibariensis* Baill.	Shrub/tree	Roots	Body swelling	[127]
*Tragia brevipes* Pax	Herb	Roots, leaves	Hair loss, indigestion, ulcers, anemia, hypertension, peptic ulcers, diabetes, topical anesthetic agent, rheumatism, purgative, snakebites, delayed/protracted labor, tonic, breast cancer, leukemia, urinary problems, joint problems, rheumatism, labor pains, diarrhea, antivenom, purgative, impotency	[66,67,77,81,86,88,91,99,115,117,141,168]
*Tragia furialis* Bojer	Herb	Roots, leaves	Skin diseases, diarrhea, tonic	[77,127]
*Tragia impedita* Prain	Herb	Roots	Retained placenta	[139]
**Flagellariaceae**				
*Flagellaria guineensis* Schumach.	Liana	Leaves, fruits	Venereal diseases	[127,134]
**Geraniaceae**				
*Monsonia angustifolia* E.Mey. ex A.Rich.	Herb	Leaves, whole plant	Black quarter in cattle, liver disease	[67,77]
*Monsonia glauca* R.Knuth	Herb	Roots	Fleas on calves	[67]
*Pelargonium alchemilloides* (L.) L’Hér.	Herb	Leaves	Sore eyes, eye diseases	[99,141]
*Pelargonium quinquelobatum* Hochst. ex A.Rich.	Herb	Roots	Diarrhea	[67]
**Hamamelidaceae**				
*Trichocladus ellipticus* Eckl. & Zeyh.	Shrub/tree	Bark, roots, leaves	Diarrhea, teeth problems, oral candidiasis, digestion, stomach disorders, strength	[67,77,88,109,152]
**Hernandiaceae**				
*Gyrocarpus americanus* Jacq.	Tree	Leaves	Antiseptic	[127]
**Hydnoraceae**				
*Hydnora abyssinica* A.Br.	Herb	Roots, stems, whole plant	Diarrhea, amoebic dysentery, diabetes, first/second trimester abortion, stomachache, retained placenta, female conditions	[77,81,83,102,143,168]
**Hypericaceae**				
*Harungana madagascariensis* Lam. ex Poir.	Tree	Leaves, fruits, bark, roots	Eye infection, scabies, flu, stomachache, heavy menstruation, malaria, bloody diarrhea, arthritis, breast development in young, colorectal/skin/breast cancer, general body pains, dysentery, itchy genitals, acaricide, infertility, wounds, chest pain, jigger/thorn, headache, cough, venereal diseases	[66,74,98,101,112,116,117,119,122,133,142,143,144,154,168]
*Hypericum peplidifolium* A.Rich.	Herb	Whole plant, leaves, roots	Injured/infected eyes, dermatitis, diarrhea	[144]
*Hypericum quartinianum* A.Rich.	Shrub	Leaves, roots, branches	Rheumatism, diarrhea	[99]
*Hypericum revolutum* Vahl	Shrub/tree	Roots, leaves	Joint pains, diarrhea, rheumatism, wounds	[99,109]
*Psorospermum febrifugum* Spach	Shrub/tree	Fruit, bark/stem/leaf	Acaricide, fattening (goats, rams)	[133,144]
*Psorospermum orientale* (Engl.) Byng & Christenh.	Shrub/tree	Roots	Tonic	[142]
**Hypoxidaceae**				
*Hypoxis obtusa* Burch. ex Ker Gawl.	Herb	Roots	Baby/children skin rashes, stomach medicine	[140,168]
**Icacinaceae**				
*Apodytes dimidiata* E.Mey. ex Arn.	Shrub/tree	Seeds, fruits, roots, bark	General body weakness, joints pains/weakness, sore throat, malaria, chest pains, tonic, stomachache, anthelmintic, diarrhea, internal injuries, blood clot in muscles, livestock diseases, spleen pain and enlargement	[67,77,140,152]
*Pyrenacantha kaurabassana* Baill.	Herb	Tubers	Jiggers, boils, rashes, aphrodisiac, fetus development, labor pains, veterinary medicine	[142,150]
*Pyrenacantha malvifolia* Engl.	Herb		Induce vomiting in pregnant women or those suspected to have malaria	[92]
**Iridaceae**				
*Gladiolus dalenii* Van Geel	Herb	Roots	Meningitis, malaria, diarrhea, asthma, allergy, joint pains, rheumatism	[98,118,140]
**Juncaceae**				
*Juncus oxycarpus* E.Mey. ex Kunth	Herb	Flowers	Colds, chest pains	[67]
**Lamiaceae**				
*Aeollanthus repens* Oliv.	Herb	Leaves, whole plant	Eye diseases, mouth thrush	[141]
*Ajuga integrifolia* Buch.-Ham. ex D.Don	Herb	Leaves, aerial parts, whole plant, stems, roots	Malaria, menstrual pains, indigestion, abdominal pains, boils, chronic joint pains, amoeba, back/bone problems, bites, cough, colds/flu, headache, livestock diseases, rheumatism, typhoid, arthritis, fever, gonorrhea, toothache, epilepsy, diabetes mellitus, acidity, nausea, pneumonia, body pains, dysentery, stomachache, retained placenta, anaplasmosis, mastitis, ECF (cows), Newcastle disease (poultry), helminthiasis (cows, poultry, goats, sheep, pigs), lung diseases (cows, goats, sheep, poultry), anthelmintic, tonsils (cattle), indigestion, menstrual problems, convulsions, dermatitis, liver problems, hypertension, afterbirth pains, measles	[7,14,41,64,66,67,68,73,74,77,84,85,86,87,101,103,104,107,110,115,122,129,130,138,141,143,144,147,153,154,168]
*Clerodendrum hildebrandtii* Vatke	Shrub/tree	Roots	Chest medicine, general medicine	[134,142]
*Clerodendrum johnstonii* Oliv.	Shrub	Leaves, roots, shoots	Malaria, hypertension, heart pains, joint pains, toothache, body pains, abdominal pains, aphrodisiac, dysmenorrhea, general weakness, stomach upsets/pains	[14,88,99,101,104,141,145,147,152]
*Clerodendrum rotundifolium* Oliv.	Shrub	Leaves, roots, bark	Malaria, stomachache, fever	[104,143]
*Clinopodium abyssinicum* (Benth.) Kuntze	Herb	Leaves, whole plant	Cough, headache, indigestion, anodyne, eye diseases, abdominal pains	[7,132,141]
*Clinopodium uhligii* (Gürke) Ryding	Herb/shrub		Constipation, headache, chest congestion	[7,86]
*Coleus aegyptiacus* (Forssk.) A.J.Paton	Herb/shrub		Stomachache, prenatal care, stomach after giving birth	[77,127]
*Coleus barbatus* (Andrews) Benth. ex G.Don	Shrub	Leaves, roots	Abdominal pains, diarrhea, vomiting, malaria, stomachache, joint pains, constipation, headache, backache, protrusion of bowels, livestock diseases, body swellings/legs, joint pain, liver diseases, skin rashes, stomach upsets, dysentery, amoeba, arthritis, bilharzia, brucellosis, cough, colds/flu, dental problems, eczema, eye problems, hypertension HIV/AIDs, injuries/cuts, wounds, neutralizes poison, rheumatism, typhoid, anthelmintic, retained placenta, constipation, tonic, sprains, stiff neck, muscle cramp, colic pain in infants, eyes, oral thrush, measles, snakebites, skin growths, tooth/gum aches, pneumonia, biopesticide, first abortion, vomiting, pneumonia (cow), fractures, dysentery, chest problems, skin diseases, abscess, warts, acaricide, indigestion, painful teeth, mouth ulcers, tetanus, disinfectant, asthma, allergy, strength, purgative, gonorrhea, eye ailments, induce blood production, dermatitis, earache, skin damaged (cow)	[7,41,73,74,79,81,84,86,87,88,96,98,100,101,106,109,111,113,115,116,119,120,121,122,129,132,133,136,141,144,146,147,152,168]
*Coleus caninus* (B.Heyne ex Roth) Vatke	Herb	Leaves, roots	Cough, liver diseases, livestock diseases, toothache	[7,77,99]
*Coleus cylindraceus* (Hochst. ex Benth.) A.J.Paton	Herb	Whole plant, stems	Morning sickness, general body weakness, peptic ulcers, livestock diseases, prevent first abortion	[7,81,139]
*Coleus hadiensis* (Forssk.) A.J.Paton	Herb	Leaves, stems	Childbirth	[72]
*Coleus igniarius* Schweinf.	Shrub	Bark	Malaria, livestock diseases, strength	[77,104,136]
*Coleus lactiflorus* Vatke	Shrub	Aerial parts	Acaricide, stomach trouble	[133,142]
*Coleus lasianthus* Gürke	Herb	Whole plant	Kwashiorkor, edema in children	[51]
*Coleus maculosus* (Lam.) A.J.Paton	Herb	Roots	Stomachache, strength	[7,136]
*Coleus otostegioides* (Schweinf. ex Gürke) A.J.Paton	Herb/shrub	Whole plant	Kwashiorkor, edema in children	[51]
*Coleus prostratus* (Gürke) A.J.Paton	Herb	Whole plant	Skin problems	[118]
*Coleus succulentus* Pax	Herb	Leaves, stems	Abscesses	[94]
*Coleus sylvestris* (Gürke) A.J.Paton & Phillipson	Herb/shrub	Leaves	Wounds, livestock diseases, allergies, amoebiasis, diarrhea, painkiller, toothache, typhoid, constipation, strength	[7,41,136,152]
*Coleus umbrosus* Vatke	Herb	Roots, leaves, whole plant	Smallpox, tonic, livestock diseases, emetic, diarrhea, stomachache	[7,51,142,168]
*Endostemon tereticaulis* (Poir.) M.R.Ashby	Herb	Roots, shoots	Malaria, edema, diarrhea, backpains	[51]
*Equilabium kamerunense* (Gürke) Mwany. & A.J.Paton	Shrub	Whole plant	Stomachache	[7]
*Equilabium laxiflorum* (Benth.) Mwany. & A.J.Paton	Herb	Roots, leaves	Anthelmintic, sore throat, purgative, infertility, eye diseases, abortifacient	[99,141]
*Fuerstia africana* T.C.E.Fr.	Herb/shrub	Leaves, roots, whole plant, shoots	Malaria, snakebites, pneumonia, ulcers, infertility, mouth inflammation in children, respiratory disorders, ringworm, abscesses, ophthalmia, problems, skin complaints, colds, heartburn, oral thrush, livestock diseases, diabetes mellitus, stomachache, urinary infections, ulcers, gonorrhea, venereal diseases, postpartum hemorrhage, prevent first/second trimester abortion, pregnancy edema, pregnancy anemia, eyes infection, colorectal cancer, oral infections, anthelmintic, respiratory disorders, internal injury, swollen legs, wounds, bloody diarrhea, mouth ulcers, boils, STIs, cuts, toothache, tonic, infant postpartum care, sore/red eyes, ear diseasesfemale conditions, dandruffs, sore throat, chest complaints, kidney	[7,67,71,74,81,82,85,86,88,91,94,97,99,101,102,103,104,115,117,119,122,126,140,141,152,168]
*Hoslundia opposita* Vahl	Shrub	Roots, leaves, whole plant	Prenatal care, convulsions, tonic, asthma, cough, colds/flu, typhoid, snakebite, aphrodisiac, constipation, sore throat, postpartum hemorrhage, prevent first trimester abortion, vomiting, acaricide, colic pain in children, ameba, oral thrush/wounds, cuts, pimples/scabies, itches/rashes, anthelmintic, stomach pains, sores, ulcers, boils, hiccups, hemorrhoids, diabetes, blood purifier, child delivery, snakebites, edema, malaria	[7,41,51,64,75,81,84,91,111,112,127,131,133,144,145,148,150]
*Hyssopus officinalis* L. (exotic)	Herb/shrub		Cough, colds, sore throat, nose blocking, indigestion	[168]
*Leonotis nepetifolia* (L.) R.Br.	Herb	Leaves, buds, whole plant, roots, aerial parts	Malaria, wounds, heart problems, stomachache, lungs/kidneys/liver problems, cough, toothache, boils, heartburn, oral thrush, acidity, anthelmintic, vomiting, diarrhea, gonorrhea, acaricide, gastrointestinal problems, abortion, anaplasmosis in cattle, insect bites, skin diseases, colds, bleeding during pregnancy, conjunctivitis, headache and body weakness, eye treatment, fever, problems in passing urine	[7,14,67,76,79,81,86,123,124,133,136,138,142,143,144,147,152,168]
*Leonotis ocymifolia* (Burm.f.) Iwarsson	Shrub	Bark, roots, leaves, whole plant	Wounds, teething, amoeba, back/joint/bone problems, cough, colds/flu, dental problems, diarrhea, indigestion, appetizer, malaria, rheumatism, ulcers, anthelmintic (human/animals), antiseptic, skin rashes, blood purifier, dysentery, chest congestion, diabetes, gall sickness, stomach pains, diarrhea, urinary problems, sore lips and tongue, anaplasmosis (cattle), insect bites, skin diseases, venereal diseases, fever, edema, problems in passing urine	[7,41,67,84,86,89,99,109,115,129,138,140,142,144,152,168]
*Leucas calostachys* Oliv.	Shrub	Roots, bark, leaves	Colds, cough, diarrhea, malaria, respiratory problems, headache, tonic, pneumonia, sprains, heartburn, peptic ulcers, abdominal distension, colic pain in infants, amoeba, hypertension, renal disorders, skin disorders, cancer, arthritis, acaricide, stomachache, gastrointestinal problems, expectorant, dysmenorrhea, abdominal pains, hernia, cuts, wounds, livestock (diarrhea, chest problems)	[7,79,86,87,101,105,120,125,133,141,142,143,144]
*Leucas deflexa* Hook.f.	Herb		Eye infection in livestock	[125]
*Leucas glabrata* (Vahl) Sm.	Herb	Root	Child’s tonic	[142]
*Leucas grandis* Vatke	Herb/shrub	Leaves, roots, whole plant	Wounds, tonic, colds, persistent skin disease, anthelmintic, ear infection, abdominal pains, ringworms, stomachache, diarrhea, anaplasmosis in cattle, anti-acid, earache, thrush (in children) and sores	[7,14,74,84,120,141,144,168]
*Leucas jamesii* Baker	Shrub	Leaves	Cholera, stomachache	[92]
*Leucas martinicensis* (Jacq.) R.Br.	Herb	Leaves	Eye problems, wounds, liver problems, gastrointestinal troubles, constipation, stomachache, black tongue, eye ailments, edema, fever, snakebite	[7,95,109,144]
*Leucas tomentosa* Gürke	Shrub	Roots	Dysentery, stomachache, purgative	[142]
*Mesosphaerum pectinatum* (L.) Kuntze	Herb/shrub	Leaves, roots	Molluscoid, stomachache, diarrhea, skin problems, gastrointestinal problems, oral thrush, fever in animals	[7,119,122,143,144]
*Mesosphaerum suaveolens* (L.) Kuntze	Herb	Leaves	Skin wounds, fungal infections	[118]
*Micromeria imbricata* (Forssk.) C.Chr.	Herb/shrub	Roots, stems, leaves, whole plant	Chest pains, pimples, mouth inflammation, livestock diseases, teeth problem, pneumonia, stomachache, gastroenteritis, cough, colds	[7,109,131,152,168]
*Nepeta azurea* R.Br. ex Benth.	Herb	Leaves	Backache, livestock diseases	[7]
*Ocimum americanum* L.	Herb	Leaves, whole plant	Malaria, delayed/protracted labor, postpartum hemorrhage, prevent first/second trimester abortion, vomiting, pregnancy edema, stomach complaints, mosquito repellant	[81,100,104,134]
*Ocimum basilicum* L.	Herb	Leaves, roots, whole plant	Headache, morning sickness, allergies, bites, cough, colds/flu, anthelmintic, nasal/bronchial catarrh, stomachache, constipation, vermifuge, diabetes mellitus, malaria, eye ailments, gastrointestinal problems, headaches, itching skin/rashes, repel insects (termites, fleas, mites, weevils, mosquitoes), abdominal pains, pain relief, rectal problems, pregnancy and child birth problems	[7,14,41,64,77,79,84,85,91,102,111,112,115,129,141,143,147]
*Ocimum gratissimum* L.	Herb/shrub	Leaves, roots, bark	Headache, diarrhea, toothache, swelling of the body, chronic joint pains, chest pains, cough, colds/flu, fever, ear/nose/throat problems, general body weakness, malaria, rheumatism, anthelmintic, skin infection, stomachache, insecticide, body rash, pesticide of stored grains, pneumonia, mosquito repellent, colorectal cancer, bronchitis, stomach ulcers, throat infections, constipation, abdominal pain, sore eyes, hiccups, hemorrhoids, control termites, runny nose, ulcers	[7,14,41,64,74,76,84,90,99,111,115,117,119,122,127,129,130,141,142,145,146,147,168]
*Ocimum kilimandscharicum* Gürke	Herb	Leaves, roots, stems, shoots	Nasal congestion, colds, flu, insect bites, general pains, malaria, diarrhea, dysentery, constipation, cough, eye problems, congested chest, measles, stomachache, repel insects, diabetes, sore eyes, acaricide, wounds, respiratory problems, fevers, abdominal pains	[7,86,96,101,106,122,125,133,139,142,147,168]
*Ocimum lamiifolium* Hochst. ex Benth.	Herb	Leaves	Respiratory disorders and common ailments, stomachache	[14,168]
*Ocimum spectabile* (Gürke) A.J.Paton	Shrub	Leaves	Asthma, cough, colds/flu, eye lotion	[41,77]
*Orthosiphon thymiflorus* (Roth) Sleesen	Herb	Leaves	Stomach and oral cavity infections	[123]
*Platostoma africanum* P.Beauv.	Herb	Shoots	Mouth thrush, sore throat	[144]
*Platostoma hildebrandtii* (Vatke) A.J.Paton & Hedge	Herb/shrub	Leaves	Colds, wounds	[7]
*Premna chrysoclada* (Bojer) Gürke	Shrub/tree	Roots, leaves	Diarrhea, prenatal care, convulsions, induce conception, dysentery, kidney trouble, purgative, fevers, female reproductive disorder, malaria	[75,127,134,142,150]
*Premna hildebrandtii* Gürke	Shrub/liana	Leaves, roots	Convulsions, diarrhea, stomachache	[127,142]
*Premna oligotricha* Baker	Shrub	Roots	Joint/bone pain	[77]
*Premna resinosa* (Hochst.) Schauer	Shrub	Roots, leaves	Fever, venereal diseases, fever, malaria, mosquito repellant, joint pains	[77,80,127,129]
*Premna serratifolia* L.	Shrub/tree	Leaves	Headache	[142]
*Rotheca microphylla* (Blume) Callm. & Phillipson	Shrub	Rots, leaves	Convulsions, headache, vomiting, gastrointestinal gas, cerebral malaria, postnatal problems	[127,142,150]
*Rotheca myricoides* (Hochst.) Steane & Mabb.	Shrub	Roots, bark, leaves, shoots	Sore throat, ear infections, malaria, gonorrhea, syphilis, aphrodisiac, STIs, venereal diseases, diarrhea, polio, joint pains, abscesses, back/bone pains, burns, cough, colds/flu, tonsilitis, arthritis, typhoid, rheumatism, emetic, gonorrhea, heartburn, oral thrush, cancer, throat cancer, diabetes mellitus, polio, abortions, headache, clean the womb, diabetes, gland complications, respiratory diseases, stomachache, gastrointestinal disorders, lumbago, venereal diseases, pneumonia, sore throat, ear problems, flu, dysentery, diarrhea, headache, eye infections, chest pains, bronchitis, indigestion, infertility, amoebic dysentery, anthelmintic, rheumatism, acaricide, venereal diseases, typhoid, asthma, strength, splenic pain and enlargement, enema against extreme body weakness due to chronic diseases, abdominal pains, threatened miscarriage, joint diseases, stimulate spasms during childbirth, menstrual pains, tonic, mental illness, abdominal pains during menses, HIV/AIDs, retained placenta, rabies, measles, TB, colic, eye disease, swellings, wounds, asthma, aphrodisiac, labor pains, poultry fever, ECF in livestock, toothache, purgative	[14,41,66,67,68,72,77,79,83,84,85,86,91,94,97,98,99,101,103,105,107,113,116,119,120,123,124,129,130,132,133,135,136,137,138,139,140,141,142,143,144,146,147,149,154,168]
*Salvia coccinea* Buc’hoz ex Etl. (exotic)	Herb	Leaves	Menstrual overflow, ulcers, bad eye, breast/esophageal/colorectal cancer	[117,147]
*Salvia leucantha* Cav. (exotic)	Shrub	Shoots, leaves	Burns, edema	[144]
*Salvia rosmarinus* Spenn. (exotic)	Shrub	Leaves, stems	Chest problems, cough, colds/flu, pneumonia, retarded memory, rheumatism, stomachache, stress, inappetence, smelly body/feet	[41,74,119,147]
*Tetradenia urticifolia* (Baker) Phillipson	Shrub	Stems, leaves, roots,	Malaria, inappetence, amoeba, catalyzes vomiting, dental problems, general body pains, heartburns, livestock diseases, malaria, pneumonia, rheumatism, stomach disorders, cough, colds, flu, bronchitis, stomach upsets, flatulence, mouth ulcers, diarrhea, fevers, boils, mumps, stomach ailments, constipation, body pains, burns/scalds, anaplasmosis (cow, ruminants), trypanosomiasis (cow, goat), miscarriage (cow), cataract, abdominal pains, cough in cattle and goats, vomiting, asthma, edema, anthelmintic	[7,14,41,73,105,121,141,142,144,146,147]
*Tinnea aethiopica* Kotschy ex Hook.f.	Shrub	Whole plant, roots, leaves, fruits	Abdominal pains, constipation, eye lotion, *Chira*, acaricide, stomach upsets, gastrointestinal complaints in children, measles	[76,77,127,133,143,145,150]
*Vitex doniana* Sweet	Tree	Leaves, fruits, bark	Diarrhea, vomiting, whooping cough, acaricide, allergies, colds, cancer of breast and prostate glands, vitamin supplements, wounds	[84,98,120,133,144,147]
*Vitex ferruginea* Schumach. & Thonn.	Shrub/tree	Fruits, roots	Appetite, gastrointestinal complaints	[150]
*Vitex fischeri* Gürke	Tree	Leaves, bark	Livestock diseases, acaricide, anti-acid, fever, infectionstomachache	[14,41,133,144]
*Vitex payos* (Lour.) Merr.	Shrub/tree	Roots	Venereal diseases, amoeba, back/joint/bone problems, chest problems, dental problems, diarrhea, increase blood, typhoid	[41,127]
*Volkameria eriophylla* (Gürke) Mabb. & Y.W.Yuan	Shrub/tree	Leaves, roots	Swollen testicles, otitis, earache, anthelmintic, amoeba, stomachache, snake bites, malaria, edema	[51,74,103,104,142]
**Lauraceae**				
*Kuloa usambarensis* (Engl.) Trofimov & Rohwer	Tree	Bark, leaf, roots	Back pain, asthma, cancer, cardiac disorders, cough, colds/flu, dizziness, hypertension, joints, malaria, pneumonia, rheumatism, typhoid, anthelmintic, chest pains, appetizer, whooping cough, body ache, swelling, tumors, measles, muscular contractions, disturbed breathing, stomachache, antidote, aphrodisiac, clearing throat, relaxation	[14,41,103,104,113,145,146,147,168]
*Persea americana* Mill. (exotic)	Tree	Bark, seeds, roots, leaves, fruits	Toothache, anemia, invigorant, amoeba, blood purifier, cancer, cough, colds/flu, dental problems, ear-nose-throat problems, energy boost, headache, immune booster, increase blood, malaria, prostate cancer, rheumatism, skin diseases, stomach disorders, diabetes, colorectal/skin/breast cancer, skin, gut problems, STIs, toothache, cholesterol control	[14,41,83,88,116,117,119,152]
**Lecythidaceae**				
*Barringtonia racemosa* (L.) Spreng.	Tree	Bark	Fish poison	[142]
**Fabaceae**				
*Abrus fruticulosus* Wall. ex Wight & Arn.	Shrub	Roots	Pneumonia	[84]
*Abrus precatorius* L.	Shrub	Leaves, roots, seeds, whole plant	Eye problems, asthma, septic swelling, gynecological pains, venereal diseases, colds, cough, stomachache, diabetes mellitus, febrifuge, fever, throat, skin cancer, gonorrhea, emetic, inflamed breasts during breast-feeding, family planning, emetic, poison, asthma, epilepsy, heart pain, snakebites, venereal diseases, malaise	[75,76,85,95,100,108,117,119,127,134,142,143,150]
*Acacia mearnsii* De Wild. (exotic)	Tree	Leaves, stems, bark, roots	Typhoid, chronic joint pains, chickenpox, dental problems, measles, skin diseases, smallpox, toothaches, wounds from poisoned arrows, cough (cows, goats, sheep, poultry, pig, ruminants)	[41,73,130,146,147,152]
*Aeschynomene abyssinica* (A.Rich.) Vatke	Herb/shrub	Leaves, bark, roots	Heartburn, chest pain, cough, uterine/skin/squamous cell carcinoma of the gums	[117,120,140]
*Afzelia quanzensis* Welw.	Tree	Roots, bark	Chest pains, stomachache, postnatal care, chest pain, snakebites, excessive menstruation	[127,150]
*Albizia amara* (Roxb.) B.Boivin	Tree	Bark, branches, fruits, roots, stem	Muscle and bodybuilding, indigestion, smooth baby skin and softens baby faces, back/bone problems, cough, colds/flu, chest problems, joints, malaria, peptic ulcers, hypertension, female infertility, stomachache, emetic, wounds, joint diseases, typhoid, piles, diarrhea, gonorrhea, ulcers, dandruff, cancer	[14,41,64,84,86,103,104,107,128,135,142,149]
*Albizia anthelmintica* (A.Rich.) Brongn.	Tree	Bark, roots, leaves, aerial parts	Malaria, stomachache, backache, emetic, induce bile release from the gall bladder, purgative, tonic, helminthiasis, diarrhea, convulsions, allergies, amoeba, joint/bone pain, general body pains, gonorrhea, headache, women’s health, wounds, syphilis, arthritis, flu/cold, pneumonia, brucellosis, anthelmintic (human, animals), runny nose, cough, skin diseases, asthma, snakebite, aphrodisiac, venereal diseases, edema, eye problems, retained placenta, abdominal pains, tonic, fever, liver diseases (poultry), cattle diseases (eye injuries, cough, bloat)	[14,41,51,64,67,68,72,80,84,90,92,96,100,102,105,107,110,121,127,128,132,134,135,137,138,139,141,142,150,168]
*Albizia coriaria* Welw. ex Oliv.	Tree	Roots, bark, stems, leaves	TB, genital thrush, gonorrhea, malaria, STIs, venereal diseases, pneumonia, menorrhagia, postpartum hemorrhage, sore eyes, livestock respiratory diseases, breast/uterine/skin cancer, diarrhea, stomachache, eye problems, boils, flu, colic pains, abdominal discomfort, constipation, infertility, anthelmintic, joints/backbone, cardiac conditions, kidney ailments, acaricide, gastrointestinal conditions, threatened abortion, whooping cough, anthrax, jaundice	[66,76,79,82,104,116,117,118,124,133,143,144]
*Albizia glaberrima* (Schumach. & Thonn.) Benth.	Shrub/tree	Leaves	Headache, stomachache	[120]
*Albizia grandibracteata* Taub.	Tree	Roots, bark, leaves	Gonorrhea, headache, stomachache, acaricide	[120,125,133]
*Albizia gummifera* (J.F.Gmel.) C.A.Sm.	Tree	Bark, roots, fruits, leaves, gum	Oral thrush, postpartum bleeding, STIs, stomachache, malaria, liver diseases, abdominal ulcers, amoeba, back/joint/bone problems, chest problems, pneumonia, rheumatism, TB, typhoid, skin disorders, throat/skin cancer, gut problems, cough, flu, anthelmintic, ringworms, HIV/AIDs, dental care, acaricide, immune booster	[14,41,67,74,84,87,88,89,101,103,104,116,117,119,125,129,141,142,145,168]
*Albizia versicolor* Welw. ex Oliv.	Tree	Roots, leaves	Convulsions, chest pains, venereal diseases	[127]
*Albizia zygia* (DC.) J.F.Macbr.	Tree	Bark	Pneumonia	[98]
*Astragalus atropilosulus* (Hochst.) Bunge	Herb		Uterine pains after childbirth	[67]
*Bauhinia taitensis* Taub.	Shrub	Roots	Joint problems, malaria, pneumonia, rheumatism, sores, wounds	[41,80]
*Bauhinia tomentosa* L.	Shrub/tree	Roots, bark	Retained placenta, cough, malaria	[14,80,146]
*Bauhinia variegata* L. (exotic)	Shrub/tree	Leaves	Meat allergy, allergy rash	[144]
*Biancaea decapetala* (Roth) O.Deg.	Shrub	Roots	Skin infections, urinary tract infections, gum aches, poisonous, toothache, stomachache	[14,123,142,144,147]
*Cadia purpurea* (G.Piccioli) Aiton	Shrub	Bark	Poison, flees and parasites	[77,136]
*Cajanus cajan* (L.) Huth	Shrub	Leaves, roots, branches, seeds	Allergies, joint pains, amoeba, bites, malaria, snakebites, stomach disorders, flu, constipation, weight loss, stomachache, tonsils, eye/ear problems, infertility, nausea in pregnant women	[41,84,105,130,139,146,150,151]
*Calpurnia aurea* (Aiton) Benth.	Shrub/tree		Poisonous	[142]
*Canavalia ensiformis* (L.) DC.	Liana	Root	Urinary problem, passing blood in urine	[144]
*Cassia abbreviata* Oliv.	Tree	Aerial parts, bark, roots, leaves, pods	Malaria, diabetes mellitus, chest pain, muscular pain, fever, ulcers, goat medicine, hypertension, hemostatic, yellow fever, typhoid fever. fatigue, body aches, inflammatory skin diseases, painful joints, menstrual cramps, aphrodisiac, venereal disease, stomachache, swollen legs and body pains during pregnancy, retained placenta, toothache, cough, gonorrhea, kidney pains, colds, pain in joints	[51,85,100,128,139,142,145,148,150,151]
*Chamaecrista hildebrandtii* (Vatke) Lock	Herb	Roots, leaves	Gastrointestinal problems, body pain including bones and joints, stomachache	[79,140,143]
*Chamaecrista kirkii* (Oliv.) Standl.	Herb	Leaves, shoots	Wounds, meat allergy, skin rashes, men infertility, livestock black water	[144]
*Chamaecrista nigricans* (Vahl) Greene	Herb	Roots, leaves	Stomachache, anthelmintic	[76]
*Craibia brevicaudata* (Vatke) Dunn	Shrub/tree	Roots	Hypertension	[127]
*Craibia brownii* Dunn	Tree	Roots	Cough	[80]
*Craibia zimmermannii* (Harms) Dunn	Tree	Bark, leaves, stems	high blood pressure, fever, anthelmintic, animals (constipation)	[145]
*Crotalaria agatiflora* Schweinf.	Shrub/tree	Leaves, roots	Wounds, backache, colds, dirty body system, skin infections, STIs, gonorrhea, urinary infections, gastrointestinal problems	[109,115,142,143,152,168]
*Crotalaria axillaris* Aiton	Herb/shrub	Leaves	Sore eyes, fever, stomach upsets	[100,142,145]
*Crotalaria brevidens* Benth.	Herb		Stomachache, body swellings, gastrointestinal conditions, conjunctivitis in children	[79,95,99,143]
*Crotalaria incana* L.	Herb	Leaves, roots, whole plant	Stomach upsets (constipation, dyspepsia), cough, anemia, strength, chest pains, purgative, proper placement of baby in womb, sore throat, mouth rushes, wounds	[72,88,91,136,141,144]
*Crotalaria keniensis* Baker f.	Shrub	Roots	Fever	[109]
*Crotalaria laburnifolia* L.	Herb	Roots	Helminthiasis (cow, poultry, goat, sheep, pig)	[73]
*Crotalaria lachnocarpoides* Engl.	Herb/shrub	Root	Sore throat	[140]
*Crotalaria mauensis* Baker f.	Herb/shrub	Roots, leaves	Mouth infection in babies, asthma, urinary infections	[109,147,168]
*Crotalaria natalitia* Meisn.	Herb/shrub		Strength, cough, chest pain	[136]
*Crotalaria pallida* Aiton	Herb	Roots	Oral infections	[119]
*Crotalaria polysperma* Kotschy	Herb		Vomiting, diarrhea	[132]
*Cynometra webberi* Baker f.	Tree	Leaves	Children’s diseases	[127]
*Dalbergia boehmii* Taub.	Shrub/tree	Roots	General body pains, diarrhea, induce labor pains	[127]
*Dalbergia lactea* Vatke	Shrub/liana	Roots, leaves	STIs, diabetes, managing pain and inflammation, back pains, liver pains, gonorrhea	[113,115,154,168]
*Dalbergia melanoxylon* Guill. & Perr.	Tree	Bark, roots, leaves	Gastrointestinal pains, prenatal care, tonsils, venereal diseases, artery problems, back/joint/bone pains, chest problems, cough/colds/flu, dental problems, malaria, pneumonia, rheumatism, typhoid, tonic, liver problems	[41,84,100,127,129,134]
*Dalbergia microphylla* Chiov.	Shrub	Leaves	Mouth ulcers	[142]
*Delonix elata* (L.) Gamble (exotic)	Tree	Bark	Livestock diseases, wounds	[14,51,80]
*Delonix regia* (Bojer ex Hook.) Raf.	Tree	Bark	STIs, stomachache	[144]
*Dichrostachys cinerea* (L.) Wight & Arn.	Shrub/tree	Roots, bark, leaves, branches	Diarrhea, prenatal care, cough, convulsions, anesthesia, ulcers, gonorrhea, malaria, poisonous, scorpion stings, snakebites, bones, injuries to reduce swelling, colds, asthma, toothache, gastrointestinal problems, constipation, amoebic dysentery, body pains	[51,89,104,111,112,127,134,142,143,150]
*Dolichos compressus* R.Wilczek	Herb	Roots	Infertility	[120]
*Dolichos kilimandscharicus* Taub.	Herb	Roots, stems, leaves	Acaricide	[133]
*Dolichos oliveri* Schweinf.	Herb/shrub	Roots	Poisonous, dementia, epilepsy; fever in livestock	[77,142,143]
*Entada abyssinica* Steud. ex A.Rich.	Tree	Bark, leaves, roots	Scabies, flu, infertility, cough, anthelmintic, body aches, dysentery, stomachache, edema, diarrhea, STIs, TB, childbirth, joint/backbones, cardiac conditions, kidney ailments, cancer, veterinary uses, acaricide, sprained muscles/limbs, body swelling, heart ailments	[66,87,88,116,118,120,125,133,143,144]
*Entada leptostachya* Harms	Liana	Roots, bark, leaves, exudate	Tuberculosis, cough, boils, abscesses, cuts/wounds, amoeba, eye problems, knee problems, STIs, stomachache, snakebites/spits, arrow poison, polio, back pain, anthelmintic (human, animals), malaria, anemia, hemorrhage, high fever, madness, gastroenteritis, epilepsy, helps increase milk letdown, edema, snake repellent, swollen legs, body pains and hemorrhage in pregnant women, retained placenta, abdominal pains, eye injury, food poisoning, internal injury	[14,41,51,72,80,84,94,96,100,139,142,151,153]
*Eriosema glomeratum* (Guill. & Perr.) Hook.f.	Herb	Roots	Fever	[144]
*Eriosema robustum* Baker	Shrub	Roots	Cough	[144]
*Erythrina abyssinica* Lam.	Tree	Stems, roots, bark	Swollen lymph glands, abdominal/colic pain in children, chickenpox, infertility, malaria, urinary tract infections, cancerous wounds, stomach pains, postpartum pain, mouth, laxative, internal injuries, pneumonia, STIs, abscesses, allergies, amoeba, antidote, back/joint/bone problems, bilharzia, cancer, cholera, cough, colds/flu, dental problems, diarrhea, ear-nose-throat problems, general body pains, increase blood, injuries/cuts/wounds, livestock diseases, male paralysis, measles, medicine antidote, prostrate problems, rheumatism, RVF, stomach disorders, typhoid, ulcers, uterus problems, vomiting, whooping cough, anthelmintic, arthritis, toothache, chest pains, peptic ulcers, hypertension, mumps, gonorrhea, snakebites, amoeba, diarrhea, pregnancy anemia, pregnancy edema, anaplasmosis (cow, ruminant), helminthiasis (cow, poultry, goat, sheep, pig), skin diseases, stomachache, mumps, body swellings, chest problems, blood cleanser, evacuation of bowels, anemia, fever, liver inflammation, acaricide, eye infection, syphilis, abdominal pains, tonic, skin sores/ulcers and swellings, allergy, gonorrhea, diabetes, gout, liver disorder, epilepsy, swelling of legs, anthrax, cattle cough and bloat, elephantiasis, kidney problems, menstrual problems	[14,41,66,67,73,74,81,84,86,87,91,94,99,101,102,105,110,113,116,119,120,123,129,133,135,138,140,141,142,143,144,145,146,147,151,153,154,168]
*Erythrina excelsa* Baker	Tree	Bark, roots, exudate	Snakebites, STIs, loose stool	[118,126,143]
*Erythrina melanacantha* Taub. ex Harms	Tree	Bark	Stomachache, venereal diseases, eradicate mites from chicken/cattle, chest pain, colds, cough, snakebites	[14,80,146]
*Erythrophleum suaveolens* (Guill. & Perr.) Brenan	Tree	Bark, seeds	Poisonous	[142]
*Faidherbia albida* (Delile) A.Chev.	Shrub/tree	Fruits	Cancer, liver enlargement	[91,132]
*Gelrebia trothaei* (Harms) Gagnon & G.P.Lewis	Shrub	Fruits	Poisonous	[142]
*Grona adscendens* (Sw.) H.Ohashi & K.Ohashi	Herb/shrub		Stomachache	[125]
*Grona barbata* (L.) H.Ohashi & K.Ohashi	Herb	Leaves	Edema	[144]
*Grona setigera* (E.Mey.) H.Ohashi & K.Ohashi	Herb	Leaves, roots	Strengthen sunken part of baby head and scalp and bring head to normal shape	[144]
*Guilandina bonduc* L.	Liana	Seeds, leaves, roots	Asthma, complications during menstruation, miscarriage, internal blood clots in eyes	[127]
*Guilandina volkensii* (Harms) G.P.Lewis	Liana	Leaves, roots, bark, seeds	Eye problems, back pain in women, back/joint/bone problems, cough, colds/flu, malaria, pneumonia, rheumatism, STIs, typhoid, anthelmintic, acidity, headache, general body pains, fevers, venereal diseases, stomach ulcers, sore throat, chest pain, stomachache	[14,41,84,95,103,104,107,113,119,129,138,142,143,146,147,168]
*Hylodesmum repandum* (Vahl) H.Ohashi & R.R.Mill	Herb		Stomach upsets	[125]
*Indigofera ambelacensis* Schweinf.	Herb	Roots	Heart disease	[99]
*Indigofera arrecta* Hochst. ex A.Rich.	Herb	Leaves, roots	Toothache, abdominal cramps/pains, malaria, abortion, pain in urination, arthritis, chest pains, cough, colds, asthma, diarrhea, skin rashes, stomachache, anthelmintic, cancer, skin sores and ulcers, anthrax, congested chest, sore throat, aphrodisiac, retained placenta, hear problem, kidney ailment, fever, edema	[14,86,88,91,99,105,115,119,120,132,138,139,141,142,143,144,146,147,152,154,168]
*Indigofera atriceps* Hook.f.	Herb		Teeth problems	[132]
*Indigofera bogdanii* J.B.Gillett	Shrub	Roots	Joint pain, bone pain	[77]
*Indigofera circinella* Baker f.	Herb	Leaves, roots	Snakebites, cough, stomach problems, sore throat, stomachache	[67,126,143,144]
*Indigofera colutea* (Burm.f.) Merr.	Herb	Shoots	Kill mites, snakebites wounds	[144]
*Indigofera conjugata* Baker	Herb	Leaves	Chest problems	[144]
*Indigofera dendroides* Jacq.	Herb	Leaves	Fungal diseases	[118]
*Indigofera emarginella* Steud. ex A.Rich.	Herb/shrub	Roots, stems	Acaricide	[133]
*Indigofera garckeana* Vatke	Shrub	Roots	Gonorrhea, joint pain	[77]
*Indigofera lupatana* Baker f.	Herb/shrub	Roots	Cough, colds/flu, epiglottis hair, anthelmintic, malaria, pneumonia, backache, diarrhea, constipation	[14,41,51,74,80,96]
*Indigofera spicata* Forssk.	Herb	Roots, whole plant, stems, leaves	Cough, wounds, burns, diarrhea, sore throat	[67,77,122,143]
*Indigofera spinosa* Forssk.	Herb/shrub	Roots	Strength	[77]
*Indigofera swaziensis* Bolus	Shrub	Roots, whole plant	Malaria, anthelmintic, body aches, weaknesses, rheumatism, bone pain	[77,147]
*Indigofera tanganyikensis* Baker f.	Herb	Roots	Baby diseases	[140]
*Indigofera trita* L.f.	Herb/shrub	Leaves	Headache, stomachache	[99,142]
*Kotschya africana* Endl.	Shrub	Various parts	Acaricide	[133]
*Lablab purpureus* (L.) Sweet	Herb	Seeds, roots	Lung disease in sheep, poisonous	[168]
*Millettia dura* Dunn	Shrub/tree	Bark, roots	Allergies, cough, colds/flu, malaria, typhoid, anthelmintic, ECF in cattle, pneumonia, diarrhea, amoeba, madness	[14,41,74,146]
*Millettia usaramensis* Taub.	Shrub/tree	Roots	Coughs, chest pains	[127]
*Mucuna gigantea* (Willd.) DC.	Liana	Leaves	Cough	[120]
*Mucuna poggei* Taub.	Liana	Bark, root, aerial parts	Acaricide	[133]
*Mundulea sericea* (Willd.) A.Chev.	Shrub/tree	Bark, leaves	Fish poison, jiggers	[142,150]
*Neonotonia wightii* (Wight & Arn.) J.A.Lackey	Herb	Leaves	Breast cancer, edema, excess bleeding after miscarriage	[117,144]
*Neorautanenia mitis* (A.Rich.) Verdc.	Herb	Roots	Abortifacients	[141]
*Newtonia buchananii* (Baker) G.C.C.Gilbert & Boutique	Tree	Bark, root	Malaria, anthelmintic, chest pains, gonorrhea, blood pressure, anemia, immune booster, fatigue, runny nose (cattle), colds, cough	[14,41,146]
*Newtonia hildebrandtii* (Vatke) Torre	Tree	Bark	Chest problems, dysentery, asthma and other respiratory disorders, malaria, stomach disorders	[41,72,77,80,129]
*Ormocarpum keniense* J.B.Gillett	Shrub	Leaves	Anthelmintic	[80]
*Ormocarpum kirkii* S.Moore	Shrub/tree	Bark, roots, leaf	Postpartum bleeding, threatened abortion, cuts, wounds, septic swelling, diabetes mellitus, headache, swollen breasts, stomachache, livestock stomach cleansing, diarrhea, dislocations, joint swellings, malaria, colds	[14,85,90,100,105,127,142,143,145]
*Ormocarpum sennoides* (Willd.) DC.	Shrub/tree	Roots, leaves	Convulsions, prenatal care, gynecological pains, burns, children’s diseases	[127]
*Ormocarpum trachycarpum* (Taub.) Harms	Shrub/tree	Leaves, roots, stems, bark	Diarrhea, whooping cough, miscarriage, sunken fontanelle, swellings, cough, tuberculosis	[67,76,107,140]
*Ormocarpum trichocarpum* (Taub.) Engl.	Shrub/tree	Roots, leaves	Ringworm, stomachache, ulcers, maintain pregnancy, pregnancy cramps, gastrointestinal disorders, bleeding after miscarriage, diarrhea, stomach problems, dislocations, joint swellings	[67,71,77,79,94,96,106,142,143]
*Parkinsonia aculeata* L. (exotic)	Shrub/tree	Roots	Prevent miscarriage	[144]
*Philenoptera bussei* (Harms) Schrire	Tree	Leaves	Convulsions, venereal diseases, prenatal care	[127]
*Philenoptera eriocalyx* (Harms) Schrire	Tree	Leaves, bark, roots	Back/joint/bone problems, chest problems, cough, colds/flu, aphrodisiac, malaria, pneumonia, eye infections, wounds, ulcers, diabetes, blood pressure, stomachache, waterborne diseases, gonorrhea, rheumatism, digestion, chest pains	[14,41,80,84,100,104,129,132,142,148]
*Piliostigma thonningii* (Schumach.) Milne-Redh.	Tree	Bark, leaves, roots	Livestock medicine, asthma, TB, chronic joint pains, amoeba, chest problems, cough, colds/flu, malaria, typhoid, dysentery, fever, respiratory ailments, snakebites, hookworm, skin infections, livestock respiratory symptoms, prolonged menstruation, gonorrhea and other venereal diseases, ear, stomachache, childbirth, fractures, colic pains, acaricide, venereal diseases, chest pains, bronchitis, diarrhea, blood pressure, cattle medicine, wounds/ulcers, diuretic	[14,41,66,74,80,82,84,107,112,116,120,130,133,142,143]
*Platycelyphium voense* (Engl.) Wild	Herb	Bark	Bone/joint pain, yellow fever, snake repellant	[100,137]
*Polhillides velutina* (Willd.) H.Ohashi & K.Ohashi	Herb/shrub	Leaves	Sore eyes	[112]
*Pseudarthria hookeri* Wight & Arn.	Herb/shrub	Roots	Abdominal pains, diarrhea	[87]
*Pterolobium stellatum* (Forssk.) Brenan	Liana	Roots, leaves	Colds, cough (asthma), splenomegaly, stomachache, TB, chest pains, joint pains, backache	[67,87,99,107,109,113,138,142,168]
*Rhynchosia elegans* A.Rich.	Herb	Roots	Cough, mental illness	[98,143]
*Rhynchosia hirta* (Andrews) Meikle & Verdc.	Herb/shrub		Ease child delivery, retained placenta	[143]
*Rhynchosia resinosa* (Hochst. ex A.Rich.) Baker	Shrub	Leaf, stems, roots, bark	Acaricide	[133]
*Senegalia adenocalyx* (Brenan & Exell) Kyal. & Boatwr.	Shrub/tree	Roots	Septic swelling	[127]
*Senegalia ataxacantha* (DC.) Kyal. & Boatwr.	Shrub	Roots, bark	Cough, pneumonia, malaria, eyes, boils, back/joint ache, gonorrhea	[14,77,80,84,129]
*Senegalia brevispica* (Harms) Seigler & Ebinger	Shrub/tree	Roots, leaves, aerial parts	Wounds, ringworm, headache/fontanelle, back/bone joints problems, giddiness, cough, livestock diseases, malaria, stomachache, diarrhea, itchy skin, rashes, removal of ganglion, colic pain after meals, infertility, swollen reproductive organs in men, anthelmintic, jaundice in infants, swollen legs and body pains during pregnancy, edema	[41,51,67,76,77,80,91,94,96,100,106,137,138,139,140,141,143,168]
*Senegalia hamulosa* (Benth.) Boatwr.	Shrub	Bark	Diarrhea	[77]
*Senegalia mellifera* (Benth.) Seigler & Ebinger	Shrub/tree	Bark, roots, leaves	Appetizer, tonic, indigestion, postpartum tonic, sore throat, ECF, convulsions, snakebites, joint pains, cholera, asthma, gonorrhea, back/bone problems, chest problems, cough, colds/flu, general body pains, malaria, wounds, STIs, stomachache, infertility, pneumonia, syphilis, rheumatism, back/joint ache, swollen legs, diarrhea, purgative	[41,51,80,84,90,91,92,96,97,100,104,106,107,110,127,129,130,138,139,141,142,147,168]
*Senegalia montigena* (Brenan & Exell) Kyal. & Boatwr.	Shrub	Roots	Anthelmintic	[142]
*Senegalia persiciflora* (Pax) Kyal. & Boatwr.	Tree	Bark	Colic pain	[140]
*Senegalia polyacantha* (Willd.) Seigler & Ebinger	Tree	Leaves	Sores	[168]
*Senegalia senegal* (L.) Britton	Shrub/tree	Roots, bark	Indigestion, stomachache, purgative, constipation, gonorrhea, diarrhea, abdominal disorders, abortion, malaria, skin rashes, eye medicine	[67,72,77,89,92,104,107,115,136,142,148]
*Senna bicapsularis* (L.) Roxb.	Shrub	Leaves	Diarrhea, relax infant’s body	[76]
*Senna didymobotrya* (Fresen.) H.S.Irwin & Barneby	Shrub/tree	Leaves, leaves, bark, stems, aerial parts	Cathartic, malaria, back pain in women, STIs, joint pain, eye infection, constipation, *Chira*, fever, typhoid, allergies, amoeba, bone problems, chest congestion, chickenpox, diarrhea, gall bladder, general body pains, hard stool, heartburn, measles, meat allergy, rheumatism, skin diseases, stomach disorders, swellings, urinary system infections, oral cavity infections, cancer, breast pain, skin swellings, emetic, purgative, diabetes mellitus, anthrax, boils, scurvy, mosquito/bee repellant, anaplasmosis (cow), helminthiasis (cow, poultry, goat, sheep, pig), hepatitis, colorectal cancer, back pains, pimples, pneumonia (cattle, human), skin rashes, tonsils, headache, acaricide, ringworms, antihemorrhagic, hypertension, gastrointestinal disorders, pain/swelling of liver, anthelmintic (human, animals), poisonous, stomachache, enhance childbirth, constipation in cattle, skin care, abdominal and menstruation pains, mental problems, insecticide, asthma, gonorrhea, excess bile	[14,41,67,73,76,77,78,79,84,85,86,87,91,99,101,103,104,108,109,113,115,117,119,120,123,129,131,133,134,135,138,140,141,142,143,144,145,146,147,152,153,154,168]
*Senna longiracemosa* (Vatke) Lock	Shrub/tree	Leaves, roots, stems	Stomachache, cough, malaria, headache, fever, painkiller, abortifacient, vomiting, swollen legs and body pains during pregnancy, abdominal pains	[14,72,77,80,139,142,145]
*Senna obtusifolia* (L.) H.S.Irwin & Barneby	Herb	Leaves, seeds, roots	Children’s medicine, abortifacient	[91,142]
*Senna occidentalis* (L.) Link	Shrub	Leaves, roots	Malaria, stomachache, diarrhea, typhoid, fever, backache, cough, chest pain, gastrointestinal problems, gastric complaints, cathartic, abdominal menstruation pains, fever and dizziness (people, livestock), snakebites, kidney problems, ECF, swollen testicles	[14,51,76,79,80,101,108,111,118,119,122,124,143,144,147,150]
*Senna septemtrionalis* (Viv.) H.S.Irwin & Barneby (exotic)	Shrub	Leaves, roots, seeds	Inhalant, anaplasmosis (cow), helminthiasis (cow, poultry, goat, sheep, pig), retained placenta, purgative, constipation, afterbirth pains, stomachache	[66,73,99,168]
*Senna siamea* (Lam.) H.S.Irwin & Barneby (exotic)	Tree	Roots, stems, leaves	Snakebites, allergies, back/joint/bone problems, cough, colds/flu, headache, injuries/cuts, wounds, malaria, TB, ulcers, gonorrhea, acaricide, stomachache	[41,106,126,133,143,147]
*Senna singueana* (Delile) Lock	Shrub/tree	Leaves, bark, roots	Prenatal care, general body pains, venereal diseases, anthrax, chickenpox, cough, colds/flu, dental problems, indigestions, diabetes mellitus, malaria, fevers, diarrhea, stomach disorders, sore throat, anthelmintic, acaricide, anthrax, elephantiasis	[14,41,68,77,84,85,127,129,133,142,145,147]
*Senna spectabilis* (DC.) H.S.Irwin & Barneby (exotic)	Tree	Bark, roots, leaves	Chronic joint pains, cramping, backbone, STIs, venereal diseases, joint ailment	[106,116,130,144]
*Sesbania macrantha* Welw. ex E.Phillips & Hutch.	Shrub	Leaves, stems	Acaricide	[133]
*Sesbania sesban* (L.) Merr.	Shrub/tree	Leaves, stems, roots, whole plant, bark, seeds	Stomach ailments, ulcers, tonic, throat/uterine/skin cancer, *Chira*, flu, childbirth, poultry, genital infections, constipation, acaricide, hunchback, jaundice, livestock blindness, afterbirth pains, retained placenta	[66,76,77,116,117,119,133,143,144]
*Sophora tomentosa* L.	Shrub		Fish poison	[142]
*Stylosanthes fruticosa* (Retz.) Alston	Shrub/tree	Roots, leaves, whole plant	Malaria, first trimester abortion, pregnancy edema	[81,96]
*Tamarindus indica* L.	Tree	Roots, fruits, bark, leaves	Nausea, vomiting, diarrhea, malaria, stomachache, headache, dizziness, snake spits/bites, allergies, pimples, joint pains, amoeba, back/bone problems, chickenpox, cough, colds/flu, eye problems, appetizer, measles, ringworms, arthritis, gonorrhea, cancer, diabetes mellitus, rheumatism, earache, dysentery, fevers, asthma, leprosy, ulcers, liver disease, acaricide, sores, ulcers, boils, rashes, amenorrhea, throat infections, anthelmintic, livestock diseases, laxative, tuberculosis, typhoid, general body malaise, leech, pneumonia, anemia, retained placenta, abdominal pain, tonsillitis, smallpox, edema	[14,41,51,66,76,77,80,85,88,96,98,99,106,110,111,112,121,124,127,132,133,134,137,139,141,142,143,144,145,146,147,150,151]
*Tephrosia emeroides* A.Rich.	Herb	Roots	Cough	[140]
*Tephrosia interrupta* Hochst. & Steud. ex Engl.	Shrub	Leaves	Poultry for fever, induce lactation (cows), sore throat	[144]
*Tephrosia noctiflora* Bojer ex Baker	Herb/shrub	Roots	Fever	[77]
*Tephrosia polyphylla* (Chiov.) J.B.Gillett	Herb	Roots	Fever	[77]
*Tephrosia purpurea* (L.) Pers.	Herb	Roots	Malaria	[74]
*Tephrosia uniflora* Pers.	Herb/shrub	Roots	Fever	[77]
*Tephrosia villosa* (L.) Pers.	Herb	Roots	Prenatal care, cough, fever, malaria	[51,127]
*Tephrosia vogelii* Hook.f.	Herb	Roots, leaves, fruits, bark, seeds	Diseases, constipation, control of rodents ectoparasites, crop pests, general ectoparasites (cow, poultry), acaricide, fish poison	[73,74,133,142]
*Teramnus labialis* (L.f.) Spreng.	Herb	Roots	*Chira*	[76]
*Trifolium baccarinii* Chiov.	Herb	Leaves	Infected gums	[144]
*Trifolium rueppellianum* Fresen.	Herb	Shoots	Fever in animals, measles, anthelmintic	[144]
*Tylosema fassoglense* (Kotschy ex Schweinf.) Torre & Hillc.	Herb/shrub	Roots, leaves, whole plant	Epilepsy, infertility in women, renal disease, cancer, epilepsy, constipation (huma, livestock), gastrointestinal complications e.g., stomachache, anemia, fever, jaundice, pneumonia, hypertension, fever/joint pains in livestock, acaricide, flu, asthma, diabetes, obesity, venereal diseases	[79,82,86,87,98,133,143,144]
*Vachellia abyssinica* (Hochst. ex Benth.) Kyal. & Boatwr.	Tree	Bark, roots, leaves	Spinal cord problem, body weakness, arthritis, gastrointestinal distress, lumbago, wounds, colds/flu, skin problems	[68,84,109,116,143,154]
*Vachellia amythethophylla* (Steud. ex A.Rich.) Kyal. & Boatwr.	Tree	Leaves, roots, bark	Acaricide	[133]
*Vachellia ancistroclada* (Brenan) Kyal. & Boatwr.	Shrub/tree	Roots, bark	Indigestion, appetizer, tonic, malaria	[92,138]
*Vachellia drepanolobium* (Harms ex Y.Sjöstedt) P.J.H.Hurter	Shrub/tree	Roots, bark, branches	Cleaning women after giving birth, postpartum pain, infertility, tonic, uterine cleaning, TB, back/joint/bone problems, cough, colds/flu, diarrhea, rheumatism, sore throat, diuretic, retained placenta in (human, cows), babesiosis, gastrointestinal problems, malaria, livestock diseases, sore throat, stomachache	[41,67,68,73,77,90,92,105,121,129,135,138,142,143,168]
*Vachellia elatior* (Brenan) Kyal. & Boatwr.	Tree	Bark	Diarrhea, stomach disorder, cough	[67,77,142]
*Vachellia etbaica* (Schweinf.) Kyal. & Boatwr.	Tree	Bark, stems, roots	Stomachache, colds, venereal diseases, respiratory disorders, chest pains	[77,88,100,127,136]
*Vachellia gerrardii* (Benth.) P.J.H.Hurter	Shrub/tree	Stems, bark, roots, seeds	Retained placenta, prostrate problems, arthritis, wounds from poisoned arrows, bone pain, joint pain, backache, livestock diseases, digestion, stomachache, after-delivery tonic	[41,71,77,121,123,135,147,154]
*Vachellia hockii* (De Wild.) Seigler & Ebinger	Shrub/tree	Bark, leaves, roots	Cancer, STIs, childbirth, diarrhea, malaria, labor cramps, stomachache, boils, swelling, gynecological problems, abdominal/colic pains, fecal impaction, astringent, colds/flu, gastrointestinal problems, cough, abscess, whitlow, venereal diseases, joint ailments	[72,76,77,84,88,91,97,99,118,136,140,141,143,144]
*Vachellia horrida* (L.) Kyal. & Boatwr.	Shrub	Bark	Stomach disorders	[77]
*Vachellia kirkii* (Oliv.) Kyal. & Boatwr.	Tree	Bark	Abdominal pains	[139]
*Vachellia lahai* (Steud. & Hochst. ex Benth.) Kyal. & Boatwr.	Tree	Bark, roots, leaves, gum	Skin eruptions, astringent, stomachache, toxemias of pregnancy and bowels	[88,91,120,132,141,142,143]
*Vachellia nilotica* (L.) P.J.H.Hurter & Mabb.	Tree	Bark, roots, stems, seeds	Indigestion, tonic, painful joints/arthritis, stomachache, wounds, body aches, stomachic, stamina, stimulant, gonorrhea, chest pain, upper respiratory diseases, stomachache, joint pains, colds/flu, cough, amoeba, arthritis, back/bone problems, heartburn, burns, malaria, medicine antidote, anthelmintic (human, cattle), eye infection, diabetes, gastrointestinal complications, babesiosis, pregnancy edema, women after giving a birth, aphrodisiac, acaricide, diarrhea, dysentery, leprosy, ulcers, cancers/tumors, abdominal/colic pains, headache, tuberculosis, constipation, venereal diseases, sores, rashes, sore eyes, eyelids, liver enlargement, chest pains, firms stool, typhoid, stomach upsets/ulcers, spleen problems, kidney problems, fever, asthma, fibroids, infertility, throat cleaning and bloat in cattle, retained placenta, bleeding gums, sore throat, tonsils, kwashiorkor	[14,41,51,67,68,71,72,77,80,81,83,89,90,91,92,93,96,99,100,102,105,106,107,110,112,123,127,129,130,132,133,135,138,139,141,142,147,148,149,150,151,168]
*Vachellia oerfota* (Forssk.) Kyal. & Boatwr.	Shrub	Stems, bark, roots, leaves, seeds	Retained placenta, STIs, postpartum tonic, facilitate lactation, rejuvenation, diarrhea, women’s stomach pain, hepatitis, fever, gonorrhea, bone disease, gonorrhea, malaria, rheumatism, typhoid, amoeba, joint diseases, facilitate lactation, purgative, mouth blisters	[72,77,90,92,107,132,138,142,149]
*Vachellia paolii* (Chiov.) Kyal. & Boatwr.	Shrub	Bark	Skin diseases	[142]
*Vachellia reficiens* (Wawra & Peyr.) Kyal. & Boatwr.	Shrub/tree	Bark, roots	Strengthener, appetizer, tonic/adaptogen, laxative, stomach disorders, stomachache, aphrodisiac	[77,90,99]
*Vachellia robusta* (Burch.) Kyal. & Boatwr.	Tree	Roots, bark	Retained placenta, livestock diseases, chest pains, colds, cough, bronchial obstruction, malaria	[90,92,98,100,127,135]
*Vachellia seyal* (Delile) P.J.H.Hurter	Tree	Roots, bark, leaves	Diarrhea, cough, colds, blood cleanser, chronic joint pains, amoeba, stomachache, livestock diseases, headache, abdominal pains, induce perspiration, appetizer, gastrointestinal problems, malaria	[41,64,88,111,127,130,132,135,141,142,143,168]
*Vachellia sieberiana* (DC.) Kyal. & Boatwr.	Tree	Bark, branches, fruits, roots, stems	Given to women after delivery to help in healing, acaricide	[133,135]
*Vachellia stuhlmannii* (Taub.) Kyal. & Boatwr.	Shrub/tree	Roots, leaves, bark	Cholera, pneumonia, cough, sores, ulcers, boils	[112,127]
*Vachellia tortilis* (Forssk.) Galasso & Banfi	Tree	Bark, roots, fruits	Gonorrhea, malaria, stomachache, back/joint/bone problems, cough, colds/flu, pancreatic diseases, pains, pneumonia, wounds, fever, polio, protracted labor, bone disease/strength, indigestion, clean kidneys, cancer, diarrhea, chest pains, swollen legs, retained placenta, abdominal pains/swelling, mouth diseases/ulcers	[41,51,72,77,80,81,91,92,97,100,104,106,129,138,139,142,148]
*Vachellia xanthophloea* (Benth.) Banfi & Galasso	Tree	Bark, roots	Pneumonia, appetizer, skin disorders, fatigue, typhoid, diarrhea, stomach disorders, peptic ulcers, amoeba, hypertension, renal disorders, female infertility, mumps, cancer, arthritis, sore throat (human), cough (livestock, human), malaria, foot and mouth (ruminants, poultry, cows), indigestion, constipation, colds, receding gums, sore throat	[41,73,82,86,92,104,105,138,142,152,168]
*Vachellia zanzibarica* (S.Moore) Kyal. & Boatwr.	Shrub/tree	Roots, leaves	Stomachache, diarrhea, cough, asthma, septic swelling, snakebites	[127]
*Vigna schimperi* Baker	Herb	Leaves	Tonsils	[144]
*Vigna subterranea* (L.) Verdc.	Herb	Leaves, seeds	Nutritional supplement	[66]
*Vigna unguiculata* (L.) Walp.	Herb	Roots, leaves, seeds	Boils, conjunctivitis, poisonous	[94,143,168]
**Linaceae**				
*Hugonia castaneifolia* Engl.	Shrub/liana	Roots	Snakebites, convulsions, colds	[127,142]
**Linderniaceae**				
*Craterostigma plantagineu*m Hochst.	Herb		Toothache	[143]
**Loganiaceae**				
*Strychnos henningsii* Gilg	Shrub/tree	Roots, leaves, branches, bark	Arthritis, stomachache, malaria, body/joint/bone pain, epilepsy, diabetes, back pain in women, postpartum pain, amoeba, cough, colds/flu, headache, stomach disorders, swellings, typhoid, anthelmintic, mosquito repellant, delayed labor, prevent first/second trimester abortion, vomiting, pregnancy edema, pregnancy anemia, postpartum hemorrhage, fever, induce abortion, make arrow poison, rheumatism, chest pain, strengthen bones, dysmenorrhea, impotence, ulcers, muscular pains/swellings, diarrhea, general body weakness, pneumonia, alcohol hangover, swollen legs and body pains in pregnancy, wounds, gastrointestinal complaints, snakebites, constipation, kidney pains	[14,41,51,64,77,81,84,85,93,96,99,100,102,103,104,105,107,113,129,130,135,138,139,140,142,146,147,149,151,168]
*Strychnos innocua* Delile	Shrub/tree	Leaves, seeds	Acaricide	[133]
*Strychnos madagascariensis* Poir.	Shrub/tree	Roots, fruits	Enhances conception and pregnancy, jiggers	[127,150]
*Strychnos spinosa* Lam.	Shrub/tree	Roots, seeds, leaves, bark	Stomach ailments, pneumonia, asthma, induce labor pains, colds, flu, acaricide, jiggers, abortifacient, poisonous	[106,112,127,133,142]
**Loranthaceae**				
*Agelanthus oehleri* (Engl.) Polhill & Wiens	Shrub	Shoots, whole plant	Prevent miscarriage, enhance conception in sterile women	[77,144]
*Agelanthus pennatulus* (Sprague) Polhill & Wiens	Shrub	Leaves, roots	Chest problems, malaria, fever, pneumonia, colds, joint pain, stomach complications	[109]
*Agelanthus sansibarensis* (Engl.) Polhill & Wiens	Shrub	Leaves, branches	Headache	[99]
*Emelianthe panganensis* (Engl.) Danser		Whole plant	Prevent miscarriage	[77]
*Englerina woodfordioides* (Schweinf.) Balle ex M.G. Gilbert	Shrub	Whole plant, bark, twigs	Pneumonia, liver/spleen diseases, pneumonia, liver enlargement, heartburn	[67,99,132,141]
*Erianthemum dregei* (Eckl. & Zeyh.) Tiegh.		Bark	Snake bite wounds	[99]
*Oliverella hildebrandtii* (Engl.) Tiegh.	Shrub	Whole plant	Prevent miscarriage	[77]
*Oncocalyx fischeri* (Engl.) M.G.Gilbert	Shrub	Whole plant, bark, twigs	Pain, retained afterbirth, wounds, prevent miscarriage	[67,68,77]
*Phragmanthera usuiensis* (Oliv.) M.G.Gilbert	Shrub	Whole branch, branches, bark, leaves, roots	Clean umbilical cord wound, paralysis, stroke, gout, stomach cancer, arthritis, pneumonia, pain, colds, stroke, gout, liver diseases	[67,99,120,135,141,154]
*Plicosepalus curviflorus* (Benth. ex Oliv.) Tiegh.	Shrub	Twigs	Pneumonia	[67]
*Tapinanthus buvumae* (Rendle) Danser	Shrub		Heartburn	[132]
**Lythraceae**				
*Lawsonia inermis* L.	Shrub/tree	Roots	Emetic, stomach problems, dysentery/severe diarrhea, women’s stomach disorder after birth	[72,77,142]
*Punica granatum* L. (exotic)	Shrub/tree	Fruits, seeds, roots	Amoeba, infertility, malaria, meat appetite, STIs, stomachache, tonsilitis, pneumonia, wounds, erectile dysfunction/impotence, gonorrhea, diarrhea, anthelmintic, aphrodisiac	[14,41,84,146,168]
*Rotala tenella* (Guill. & Perr.) Hiern	Herb	Whole plant	Blood cleanser, lumbago, peripheral neuropathy, muscle cramps, joint pains, pre/post-menopausal syndromes, obesity, cardiovascular disorders, cerebrovascular disorders, hyperlipidemia	[88]
**Malpighiaceae**				
*Caucanthus auriculatus* (Radlk.) Nied.	Liana	Roots	Bone pain, rheumatism, backache, malaria	[77]
*Triaspis mozambica* A. Juss.	Liana	Leaves	Stomach medicine	[142]
**Malvaceae**				
*Abutilon hirtum* (Lam.) Sweet	Herb/shrub		Retained placenta	[136]
*Abutilon longicuspe* Hochst. ex A.Rich.	Shrub		Retained placenta	[136]
*Abutilon mauritianum* (Jacq.) Medik.	Herb/shrub	Seeds, roots, leaves	Dysentery, retained placenta, expectorant, aphrodisiac	[136,141,142,168]
*Adansonia digitata* L.	Tree	Leaves, bark, fruits, roots	Dizziness, nausea, headache, hypertension, children’s diseases, skin infections, malaria, fly repellant, fumigant, arrow poison antidote, erectile dysfunction/impotence,epilepsy, body swellings, kidney stones, measles, appetizer, backache, stomachache, anthelmintic, abdominal pains, tie umbilical cord	[14,80,84,100,111,127,129,134,139,145,150]
*Ceiba pentandra* (L.) Gaertn. (exotic)	Tree	Bark	General body pains	[116]
*Ceiba speciosa* (A.St.-Hil., A.Juss. & Cambess.) Ravenna (exotic)	Tree	Bark	Wounds	[116]
*Corchorus olitorius* L.	Herb	Leaves	Wounds, infant’s mouth, growth, teething in babies	[108,120]
*Corchorus trilocularis* L.	Herb	Leaves	Snakebites, applied to gums to treat plastic teeth in infants, sore throat, vomiting	[126,143,144]
*Dombeya burgessiae* Gerrard ex Harv.	Shrub/tree	Leaves, roots	Cough, stomach ulcers, retained placenta, pneumonia, hiccups, wounds, stomach upsets	[84,142,144]
*Dombeya kirkii* Mast.	Shrub/tree	Roots	Strength, yaws	[77,143]
*Dombeya rotundifolia* (Hochst.) Planch.	Tree	Bark, roots, stems, leaves	Dysentery, back/joint/bone problems, cough, colds/flu, malaria, wounds, rheumatism, diarrhea, meningitis, typhoid, joint pains, stomachache	[41,77,84,99,105,129,141,168]
*Dombeya taylorii* Baker f.	Shrub/tree	Roots, leaves	Stomach ailments	[127]
*Dombeya torrida* (J.F.Gmel.) Bamps	Tree	Roots, leaves, bark	Diarrhea, stomachache, backache, typhoid fever, livestock diseases, malaria, anthelmintic, chest problems, pre/post-menopausal syndrome, allergies, joint pains, STI’s, sustain pregnancy, joints, rheumatism, indigestion, allergies, vomiting, goiter, abdominal pains, liver diseases, vertigo,chest pains, colds, cough, retained placenta	[14,41,67,88,99,109,120,127,131,132,141,168]
*Grewia arborea* (Forssk.) Lam.	Shrub/tree	Leaves, bark	Chronic joint pains	[130]
*Grewia forbesii* Harv. ex Mast.	Shrub/tree	Roots	Convulsions	[127]
*Grewia holstii* Burret	Shrub	Roots	Stomach ailments, asthma	[127]
*Grewia kakothamnos* K.Schum.	Shrub	Roots, bark	Skull fissures, cough, injuries, cuts, wounds, malaria, gonorrhea, stomach problems	[41,84,92,139,142]
*Grewia lilacina* K.Schum.	Herb	Roots	Strength	[77]
*Grewia micrantha* Bojer	Shrub/tree	Roots, leaves	Dysentery, pneumonia	[142]
*Grewia microcarpa* K.Schum.	Shrub/tree	Roots	Diarrhea	[108]
*Grewia plagiophylla* K.Schum.	Shrub/tree	Leaves, bark, roots	Diarrhea, pre/post-natal care, kidney trouble, fever, blood clotting, excess menstrual bleeding, paralysis, yellow fever, malaria, obesity, skin diseases, epilepsy	[75,100,127,142,150]
*Grewia similis* K.Schum.	Shrub/tree	Roots, bark, stems	Stomachache, constipation, wounds, bleeding gums, diabetes, impotency, sores, malaria, menstrual problems	[83,106,138,147,168]
*Grewia tembensis* Fresen.	Shrub	Bark	Reduced appetite, swollen diaphragm	[51]
*Grewia tephrodermis* K.Schum.	Shrub/tree	Stem, bark, leaves, roots, seeds	Eye infections, malaria, chest pains, snakebites, cough, colds/flu, diarrhea, respiratory disorders, boils, sores, gonorrhea, infertility, retained placenta, livestock disease, soreness after birth, acaricide, stomach upsets, wounds, backache, cuts, respiratory disorders, aphrodisiac, edema, skin rashes	[14,41,51,72,80,89,92,94,100,107,124,133,135,139,143]
*Grewia trichocarpa* Hochst. ex A.Rich.	Shrub/tree	Roots	Skin, malaria, diarrhea, acaricide	[76,111,120,133]
*Grewia truncata* Mast.	Shrub	Roots, bark	Chest pains, convulsions, diarrhea	[100,127]
*Grewia villosa* Willd.	Shrub	Bark, twigs, leaves, roots, stems, fruits	Appetizer, galactagogue, tonic, stomachache, diarrhea, rheumatism, malaria, facial swellings, sore/swollen eyes, spleen diseases, cancer of breast and prostate glands,body swellings, excessive menstrual flow, remove thorns pierced into the skin, retained placenta	[14,41,51,77,84,90,92,100,134,139,141,142,145]
*Heritiera littoralis* Aiton	Tree	Roots	Anthelmintic, stomachache, mouth infection, toothache	[76]
*Hibiscus aethiopicus* L.	Herb	Roots	Spleen pain	[140]
*Hibiscus aponeurus* Sprague & Hutch.	Herb	Roots, leaves	Burns, rashes, boil, abdominal pains, fever, anemia, HIV/AIDs, dysentery, diarrhea, dermatitis, stomachache, fever	[67,77,141,143,147]
*Hibiscus calyphyllus* Cav.	Shrub	Roots, leaves, aerial parts, stems	Acaricide, abdominal pains	[133,139]
*Hibiscus diversifolius* Jacq.	Shrub	Bark	Malaria, appetizer, vomiting, diarrhea, colds, pneumonia	[100,109,168]
*Hibiscus faulknerae* Vollesen	Shrub	Roots, leaves	Convulsions, toothache	[127]
*Hibiscus flavifolius* Ulbr.	Herb	Leaves, roots	Burns	[67]
*Hibiscus fuscus* Garcke	Herb	Roots, bark, seeds, leaves	Cough, colds/flu, swollen feet, rheumatism, stroke, chest problems, wounds, aphrodisiac, chest pain, induce spasms during childbirth, sprained muscles/limbs	[41,99,115,119,143,144,147,168]
*Hibiscus greenwayi* Baker f.	Shrub	Bark	Colds	[72]
*Hibiscus kabuyeanus* Mwachala	Herb	Leaves	Boils	[100]
*Hibiscus ludwigii* Eckl. & Zeyh.	Herb/shrub		Wounds	[136]
*Hibiscus micranthus* L.f.	Herb	Roots, leaves	Eye infections, boils, *Chira*, stomach ulcers, mental disorders, cuts, fever, anemia, HIV/AIDs, chicken diarrhea	[14,76,77,80,84,100,143]
*Hibiscus vitifolius* L.	Herb	Roots	Inflammations, wounds, eyes problems	[14,80,136]
*Kosteletzkya adoensis* (Hochst. ex A.Rich.) Mast.	Herb/shrub	Shoots	Menstrual fever	[144]
*Malva parviflora* L.	Herb	Roots	Boils	[152]
*Malva verticillata* L.	Herb	Exudate, roots	Wounds, boils, toothache	[168]
*Melhania muricata* Balf.f.	Shrub	Roots	Arthritis, pneumonia, lung problems	[92]
*Melhania ovata* (Cav.) Spreng.	Herb/shrub	Leaves	Wounds, burns	[72]
*Melochia corchorifolia* L.	Herb/shrub	Roots, leaves	Chest blockage, sore throat, reduce fetal movements, typhoid, flu, waterborne diseases, headache, mumps, retained placenta	[89,109,120,132,144]
*Pavonia arabica* Hochst. & Steud. ex Boiss.	Herb	Whole plant	Fever	[77]
*Pavonia burchellii* (DC.) R.A.Dyer	Herb	Roots	Eye problems (chalazion, conjunctivitis, proptosis tumors, retina blastoma), diarrhea	[95,147]
*Pavonia urens* Cav.	Herb/shrub	Roots	Rheumatism, barrenness	[41,138]
*Rhodognaphalon mossambicense* (A.Robyns) A.Robyns	Tree	Roots	Asthma, coughs, diarrhea	[127]
*Sida cordifolia* L.	Herb	Roots, leaves	Skin cancer, family planning	[117,119]
*Sida javensis* Cav.	Herb	Leaves, whole plant	Internal injury, bleeding, back pain	[122]
*Sida massaica* Vollesen	Shrub	Leaves, roots	Excess gas in stomach, sprains	[146,152]
*Sida ovata* Forssk.	Herb/shrub		Remove retained placenta (cows), wounds, kwashiorkor	[80,138]
*Sida rhombifolia* L.	Herb	Leaves	Uterine/skin/squamous cell carcinoma of the gums, diarrhea, stomachache, sunken fontanelle	[76,117]
*Sida schimperiana* Hochst. ex A.Rich.	Shrub	Roots, whole plant	Colic pain, anthelmintic	[140,144]
*Sida tenuicarpa* Vollesen	Shrub	Roots, leaves, whole plants, aerial parts	Respiratory disorders, peptic ulcers, rheumatism, diarrhea, protracted labor, pregnancy edema, boils, wounds, pregnancy complications, chest complication, appetizer, chest pains, kidney problemssore throat, kwashiorkor	[41,81,88,119,122,138,143,147,168]
*Sterculia africana* (Lour.) Fiori	Tree	Bark, aerial parts, seeds, roots	Malaria, erectile dysfunction, stomach upsets, diarrhea, heart problem, dysentery, rectal prolapse, asthma	[51,72,91,100,129,150]
*Sterculia appendiculata* K.Schum.	Tree	Roots	Stomach ailments, hypertension	[127]
*Sterculia rhynchocarpa* K.Schum.	Shrub/tree	Leaves, roots	Induce labor pains, venereal diseases	[127]
*Sterculia stenocarpa* H.J.P.Winkl.	Herb/shrub	Roots, bark	Back/joint/bone problems, indigestion, malaria, anthelmintic, stomachache	[14,41,77]
*Thespesia danis* Oliv.	Shrub/tree	Roots, leaves	Infertility in women, convulsions, septic swelling, gonorrhea, fibroids, female sexual problems	[127,142,150]
*Thespesia garckeana* F.Hoffm.	Shrub/tree	Fruits, bark	Amoeba, back/joint/bone problems, cough, colds/flu, malaria, typhoid, stomach disorders, acidity, epigastric distress, heartburn, abdominal pain	[41,140,147]
*Triumfetta brachyceras* K.Schum.	Shrub	Boils, leaves	Boils, retained placenta, boils, malignant cells	[99,143,144]
*Triumfetta cordifolia* A.Rich.	Shrub	Leaves	Hepatic diseases	[99]
*Triumfetta rhomboidea* Jacq.	Herb/shrub	Leaves, roots	Colorectal/uterine/squamous cell carcinoma of the gums, labor pain, reduce blood flow after birth, whooping cough	[76,117,119,144]
*Waltheria indica* L.	Shrub	Leaves	Skin/uterine/breast cancer	[117]
**Marantaceae**				
*Maranta arundinacea* L. (exotic)	Herb		Tonsilitis	[41]
**Melastomataceae**				
*Antherotoma naudinii* Hook.f.	Herb		Increase milk letdown in livestock	[144]
*Antherotoma senegambiensis* (Guill. & Perr.) Jacq.-Fél.	Herb	Leaves	Falling person diseases	[143]
*Dissotis speciosa* Taub.	Herb/shrub	Leaves	Diarrhea, cough	[99,125]
*Dupineta brazzae* (Cogn.) Ver.-Lib. & G.Kadereit	Herb	Leaves	Cough	[144]
*Heterotis rotundifolia* (Sm.) Jacq.-Fél.	Herb	Leaves	Cough	[134]
*Memecylon teitense* Wickens	Shrub/tree	Leaves	Malaria, fever	[145]
*Tristemma mauritianum* J.F.Gmel.	Herb	Leaves	Allergies, heart ailment, labor pains, proper fetus positioning	[119,144]
*Warneckea amaniensis* Gilg	Shrub/tree	Roots	Bronchial trouble	[142]
**Meliaceae**				
*Azadirachta indica* A.Juss. (exotic)	Tree	Bark, leaves, roots, fruits	Eye/ear infections, typhoid, ringworms, genital thrush, herpes, malaria, fever, body pains, insect bites, pest control, skin rashes, HIV/AIDs, *Chira*, cholera, amoeba, back/joint/bone pains, blood purifier, brucellosis, chest problems, cough, colds/flu, dental problems, diabetes, hypertension, injuries/cuts, wounds, appetizer, numbness, pimples, pneumonia, rheumatism, skin diseases, STIs, stomach disorders, anthelmintic, helminthiasis (cow, poultry, goat, sheep, pig), weakness/dullness (cow, goat, sheep, poultry, pig), acaricide, boils, pimples, hepatitis, leprosy, ulcers, abdominal pains, infertility, edema, continuous menstruation, contraceptive, gonorrhea, diarrhea, gout, yellow fever, arthritis, fatigue, antiseptic, insect repellant (moth/mosquito) skin care, arthralgia, asthenia, measles	[14,41,64,66,68,73,75,84,85,93,96,98,105,106,107,108,110,111,125,129,133,138,144,145,146,147,150,151,168]
*Ekebergia capensis* Sparrm.	Tree	Bark, roots, flowers, fruits, stems, leaves	Diarrhea, stomach problems, difficult delivery in women, chest problems, pneumonia, earache, infertility in women, menstrual disorders, joint pains, kidney problem, spinal cord problem, fever, typhoid, dysentery, skin allergies, backache, chest problems, diarrhea (cow), fever, body swellings, arthritis, malaria, anthelmintic, heartburn, emetic, cough, colds, headache, gastritis, skin rashes, fungal infections, acne, abscesses, livestock diseases (respiratory symptoms, anthelmintic), tonic, skin/throat/breast cancer, skin cancer, TB, expectorant, acaricide, purgative, abdominal pains	[14,41,82,88,91,97,99,104,109,117,120,131,133,141,145,147,152,154,168]
*Lepidotrichilia volkensii* (Gürke) J.-F.Leroy	Tree	Leaves	Scalp in babies	[144]
*Melia azedarach* L.		Leaves, bark, roots	Abdominal pains, STIs, malaria, measles, immunity booster, typhoid, colorectal/esophageal cancer, stomachache, diarrhea, gut diseases, dysentery, kidney ailments, blood cleanser, skin diseases, headache, acaricide, colds/flu, fever	[41,76,79,87,101,104,108,116,117,119,120,124,133,143,150]
*Melia volkensii* Gürke	Tree	Roots, leaves, fruits, bark	Cholera, constipation, malaria, boils, amoeba, chest problems, cough, colds/flu, pneumonia, stomach disorders, insecticide, gum/tooth ache, ear problems, swollen legs, abdominal pains, edema	[14,41,51,77,80,96,139,147]
*Trichilia dregeana* Sond.	Tree	Bark	Stomach ailments, poisoning	[144]
*Trichilia emetica* Vahl	Tree	Bark, leaves, twigs, root	Flu, general body weakness, fever, stomachache, STIs, malaria, amoeba, back/joint/bone problems, cough, colds/flu, epilepsy, pancreas diseases, rheumatism, anthelmintic, moisturizer, poisoned arrow wounds, body aches, pneumonia, inappetence, breast/skin/colorectal cancer, scabies, boils, chest problems, whooping cough, TB, gut, constipation, prenatal care, abortifacients, infertility, measles, dysentery, diarrhea, cardiac conditions, veterinary, emetic, poisonous, cuts/bruises, purgative, abdominal pains, kidney problems, skin diseases, immune booster, jiggers, body swellings, venereal diseases, induce labor pains, stomach ailments in livestock, insecticide (cockroaches, bedbugs)	[14,41,66,74,80,84,101,104,112,116,117,125,134,141,142,144,145,147,168]
*Turraea abyssinica* Hochst.	Shrub/tree	Stem, bark, leaves, roots	Tonic, anthelmintic	[107,135]
*Turraea floribunda* Hochst.	Shrub/tree	Roots	Cough	[142]
*Turraea holstii* Gürke	Shrub/tree	Roots, leaves, bark	Acaricide, painkiller, nerval disorder, laxative, menstrual cycle, anthelmintic in cattle, measles, diarrhea, stomach congestion	[133,144,145,168]
*Turraea mombassana* Hiern ex C.DC.	Shrub	Fruits, roots, stems, bark	Emetic, malaria, dizziness, body pains, joint diseases, excess bile, fever	[67,104,105,107,135,140,149]
*Turraea robusta* Gürke	Tree	Roots, bark, whole plant	STIs, anthelmintic, malaria, acaricide	[104,118,133,147]
*Turraea wakefieldii* Oliv.	Shrub/tree	Bark, roots, leaves	Measles, chest pain, chickenpox, cough	[150]
**Melianthaceae**				
*Bersama abyssinica* Fresen.	Tree	Roots, bark, leaves	Urinary tract infections, STIs, enlarged prostate gland, eye inflammation, colds, stomach complications, fever, livestock diseases, birth difficulty, bleeding, stomachache, pneumonia, TB, malaria, amoebiasis, diabetes mellitus, joints/backbone, aphrodisiac, poisonous to livestock, cancer, chest problems, infertility, syphilis	[66,85,86,108,109,113,116,120,131,138,141,142,144,152,168]
**Menispermaceae**				
*Chasmanthera dependens* Hochst.	Liana	Stems	Stomachache, diarrhea	[51,139]
*Cissampelos mucronata* A.Rich.	Liana	Roots, leaves, stems	Malaria, hypertension, abortifacient, stomach ailments, constipation, abdominal pain, whooping cough, burns, snakebite antidote	[101,120,143,144,147]
*Cissampelos pareira* L.	Liana	Roots, bark, leaves	Wounds, snakebites, amoeba, asthma, diarrhea, typhoid, vomiting, anthelmintic, malaria, abdominal severe pains in women, stomachache, hypertension, cough, sore throat, colds, gastric complaints, urinary disorders, venereal diseases	[14,41,51,86,99,103,104,127,128,129,150,168]
*Cocculus hirsutus* (L.) W.Theob.	Liana	Leaves	Stomachache	[142]
*Cocculus pendulus* (J.R.Forst. & G.Forst.) Diels	Liana	Wood	Emetic	[142]
*Jateorhiza palmata* (Lam.) Miers	Liana	Roots	Stomach medicine	[142]
*Stephania abyssinica* (Quart.-Dill. & A.Rich.) Walp.	Liana	Roots, leaves, bark	Liver problems, colic in infants, stomachache, malaria, diarrhea/dysentery (cow, poultry, goat, sheep, pig), heart problems, gonorrhea, impotency	[73,74,104,119,152,168]
**Molluginaceae**				
*Paramollugo nudicaulis* (Lam.) Thulin	Herb	Leaves, whole plant	Flu, madness, cough, mental illness/madness	[76,98,143]
**Monimiaceae**				
*Xymalos monospora* (Harv.) Baill.	Tree	Bark, roots	General body relief, anthelmintic, stomachache	[14]
**Moraceae**				
*Antiaris toxicaria* (J.F.Gmel.) Lesch.	Tree	Leaves, bark, roots	Fontanel gap, abdominal pains, STIs, kidney ailments, ringworms	[116,122]
*Ficus amadiensis* De Wild.	Tree	Fruits, bark, roots, latex	Acaricide	[133]
*Ficus asperifolia* Miq.	Shrub	Latex	Skin swellings in human/livestock	[142]
*Ficus benjamina* L. (exotic)	Tree		Chest problems, diarrhea, headache, livestock diseases, stomach disorders	[41]
*Ficus bussei* Warb. ex Mildbr. & Burret	Tree	Roots, leaves	Malaria	[111]
*Ficus cordata* Thunb.	Tree	Bark, roots, stems	Boost immunity, improve general health	[135]
*Ficus glumosa* Delile	Shrub/tree	Root, bark, fruit, latex	Stomachache, diarrhea, acaricide	[77,99,133]
*Ficus ingens* (Miq.) Miq.	Shrub/tree	Roots	Diarrhea	[142]
*Ficus lutea* Vahl	Tree	Leaves, roots, bark	Sore throat, asthma, heavy menstruation, general illness, veterinary uses	[116]
*Ficus natalensis* Hochst.	Tree	Fruits, bark, roots, latex, stems, leaves	Toothache, skin infections, teeth problems, diabetes mellitus, arthritis	[66,85,109,116,132]
*Ficus populifolia* Vahl	Shrub/tree	Latex	Sore eyes	[142]
*Ficus scassellatii* Pamp.	Tree		Stomach problems, malaria	[136]
*Ficus sur* Forssk.	Tree	Bark, roots, fruits, latex	Diarrhea, vomiting, back/joint/bone problems, cough, colds/flu, dental problems, pneumonia, malaria, pregnancy edema, toothache, acaricide, stomachache	[41,67,77,81,104,116,120,133,142]
*Ficus sycomorus* L.	Tree	Leaves, bark, latex, fruits	Diarrhea, nausea, vomiting, uterine cleaning, excess menstrual flow/inconsistency, clean uterus after birth, afterbirth bleeding, toothache, stomachache, joint pains, allergies, amoeba, dental problems, clean kidney, malaria, meat allergy, pneumonia, body swellings, typhoid, abortifacient, constipation, colic pains, gut problems, abdominal discomfort, acaricide, scrofula, cough, chest diseases, dysentery, snakebites, laxative, anthelmintic, liver troubles, stomachache and hemorrhage in pregnancy	[14,41,51,66,77,89,100,105,108,110,113,116,127,130,133,135,139,143,145,146,168]
*Ficus thonningii* Blume	Tree	Bark, latex, roots	Malaria, respiratory problems, excess menstrual flow/pains, joint pains, toothache, livestock diseases, arthritis, liver diseases, edema, dental problems, diarrhea, STIs, typhoid, uterine problems, cancer, toxicity, prevent abortion, diarrhea/dysentery (cow, poultry, goat, sheep, pig), chest problems, stomachache, acaricide, sore throat, wounds, constipation, nose bleeding, mouth sores, venereal diseases, fever	[41,73,81,86,88,100,101,105,113,116,132,133,143,144,145,146,147,152,168]
*Ficus vasta* Forssk.	Tree	Bark, roots, fruits, buds, latex	Liver disease, edema, acaricide, astringent	[88,133,141]
*Milicia excelsa* (Welw.) C.C.Berg	Tree		Allergies, bites, cancer, cough, colds/flu, dental problems, family planning, hypertension, malaria, stomach disorders, STIs, typhoid	[41]
*Morus alba* L. (exotic)	Tree	Leaves, fruits	Cough, colds/flu, dental problems, rheumatism, stomach disorders, blindness, anthelmintic, blood recharge	[41,74,106]
*Morus mesozygia* Stapf	Tree	Stems, bark	Urinary tract infections, veterinary	[116]
*Morus nigra* L. (exotic)	Tree	Leaves	Arthritis	[154]
*Trilepisium madagascariense* DC.	Tree	Bark	Joint/backbone problems	[116]
**Moringaceae**				
*Moringa borziana* Mattei	Herb/shrub	Roots	Swollen legs/pains in pregnancy, retained placenta, abdominal pains, increase milk letdown, edema, malaria, gonorrhea	[51,139]
*Moringa oleifera* Lam.	Shrub/tree	Leaves, roots, seeds, flowers	Back pain, HIV/AIDs, amoeba, asthma, clean blood, cancer, chest problems, cough, colds/flu, detoxifier, fibroids, immune booster, malaria, protein source, rheumatism, stomach disorders/acid, typhoid, hypertension, toothache, ulcers, whiten teeth, diabetes, fungal infection, herpes zoster, genital thrush, herpes simplex, syphilis, anemia, arthritis, malaise, vitamin/iron source, hysteria, conjunctivitis, reproductive disorder, diabetes	[14,41,83,96,98,106,110,145,150]
*Moringa stenopetala* (Baker f.) Cufod.	Tree	Roots	Fever	[77]
**Musaceae**				
*Ensete ventricosum* (Welw.) Cheesman	Tree	Roots, exudate, fruits, stems	Infertility, snakebite, measles, runny eyes, rumbling stomach, tapeworms, livestock diseases, rheumatism, dental problems, dislocations, injuries/cuts/wounds, STIs, arthritis, heartburn, liver diseases/enlargement, medicine for cows, eye infection, earache/infection, stomachache	[41,87,91,100,116,120,126,132,141,144,145,152,154,168]
*Musa × paradisiaca* L. (exotic)	Tree	Roots, bark, exudate	Ear infections, asthma, cough, gonorrhea, family planning, wounds, female infertility, ant repellent	[14,66,113,116,146,150]
*Musa acuminata* Colla (exotic)	Tree	Bark	Blood pressure	[105]
**Myricaceae**				
*Myrica salicifolia* Hochst. ex A.Rich.	Shrub/tree	Bark, stems, leaves, roots	Urinary tract problems, tonic, tonsillitis, cough, chest congestion, goiter, malaria, livestock diseases, anodyne, abdominal pains, asthma, stomach upsets, analgesic, respiratory diseases, colds, anti-acid, intoxicating property, throat infections	[14,67,72,86,93,103,104,107,120,121,141,145,168]
**Myrothamnaceae**				
*Myrothamnus flabellifolius* Welw.	Shrub	Leaves	Chest pain	[77]
**Myrtaceae**				
*Corymbia citriodora* (Hook.) K.D.Hill & L.A.S.Johnson (exotic)	Tree	Bark, leaves	Fever, flu, cough	[143]
*Eucalyptus camaldulensis* Dehnh. (exotic)	Tree	Leaves	Colds	[98]
*Eucalyptus globulus* Labill. (exotic)	Tree	Leaves	Allergies, back/joint/bone problems, chest problems, chickenpox, cough, colds/flu, fever, headache, injuries, cuts/wounds, livestock diseases, malaria, measles, pneumonia, rheumatism, skin diseases, slow urine passing, smallpox, stomach disorders, measles, pimples	[41,84,115,116]
*Eucalyptus grandis* W.Hill ex Maiden (exotic)	Tree	Bark, leaves, roots	Fever, colds, headache	[150]
*Eucalyptus regnans* F.Muell. (exotic)	Tree	Leaves	Chickenpox, livestock diseases	[152]
*Eucalyptus robusta* Sm. (exotic)	Tree	Leaves	Skin infections, chickenpox, measles, asthma	[109]
*Eucalyptus saligna* Sm. (exotic)	Tree		Back pain in women, cough, colds/flu, injuries/cuts, wounds, malaria, skin diseases, chicken diseases, fever, toothache, measles, stomach ailments, allergy, poison emetic, chickenpox, bloat in cattle	[14,41,74,84,136,145,146,147]
*Eucalyptus tereticornis* Sm. (exotic)	Tree	Leaves	Smallpox	[106]
*Psidium guajava* L. (exotic)	Tree	Leaves, bark, fruits, roots	Constipation, malaria, allergies, amoeba, jaundice, back/joint/bone problems, cough, colds/flu, chest problems, goiter, painkiller, rheumatism, skin diseases, typhoid, stomachache, cancer, STIs, nausea, diarrhea, colic pains, anthelmintic (human, animals), typhoid, dysentery, inflammatory, ringworms, ulcers, diabetes fever, hypertension, toothache, reproductive disorders, wounds, boils, runny nose	[14,41,76,84,88,96,99,106,108,116,119,143,145,146,150,168]
*Syzygium cordatum* Hochst. ex Krauss	Tree	Leaves, bark, fruits, stems, roots	Colds, diarrhea, afterbirth stomachache, stomachache, malaria, typhoid, strength/tonic, gastrointestinal complications, acaricide, abdominal pains, cleaning blood	[14,41,67,68,72,87,120,133,136,141,142,143,152,168]
*Syzygium cumini* (L.) Skeels	Tree	Fruits, leaves, bark, roots	Dysentery, dysentery, gut problems, acaricide, boils, stomach complications, emetic, cough, hypertension	[66,98,116,122,133,145]
*Syzygium guineense* (Willd.) DC.	Tree	Bark, roots, fruits	Infertility, typhoid, wounds, paresthesia, cancer, invigorant, back/joint/bone problems, cough, colds/flu, diarrhea, HIV/AIDs, malaria, tonic, rheumatism, stomachache, skin cancer, kidney ailments, veterinary uses, menstrual pains, acaricide, eye problem, heartburn, fever, indigestion, aphrodisiac	[41,66,67,77,88,109,116,117,119,132,133,140,144,145,168]
**Nyctaginaceae**				
*Boerhavia erecta* L.	Herb	Leaves	Diarrhea, ringworm	[76]
*Commicarpus helenae* (Roem. & Schult.) Meikle	Herb		Tonic, malaria, earache, wounds	[136]
*Commicarpus plumbagineus* (Cav.) Standl.	Herb	Leaves, roots	Malaria, earache, headache, cuts, removing ganglion	[72,91,99]
**Nymphaeaceae**				
*Nymphaea nouchali* Burm.f.	Herb	Stems, leaves	Stomach ailments, chest conditions	[134]
**Ochnaceae**				
*Ochna holstii* Engl.	Tree		Stomachache, emetic when poison ingested	[145]
*Ochna insculpta* Sleumer	Shrub/tree	Roots, seeds, leaves	Strength, chest pain, diarrhea in children, boils, children fever	[14,77,140]
*Ochna mossambicensis* Klotzsch	Shrub/tree	Roots	Stomachache	[142]
*Ochna ovata* F. Hoffm.	Shrub/tree	Leaves	Sore/inflamed skin, stomachache, poison emetic	[80,145]
*Ochna thomasiana* Engl. & Gilg	Shrub/tree	Roots, leaves	Menstrual disorders, leprosy, miscarriage	[127]
**Olacaceae**				
*Strombosia scheffleri* Engl.	Tree	Leaves, bark	Stomachache, tonic, insecticide	[77,84]
*Ximenia americana* L.	Shrub/tree	Stem, bark, leaves, roots, fruits	Malaria, stomachache, constipation, uterine bleeding, tonic, venereal diseases, toothache, boils, skin rashes, abscesses, back/bone problems, dental problems, rheumatism, skin diseases, stomach acid, thorn pricks, joint pains, pneumonia, gum pains, diarrhea, acaricide, wounds, tonsillitis, mouth sores, fever, cough, anemia, menstrual pains (dysmenorrhea), gonorrhea, respiratory problems, vomiting, constipation, gynecological problems, pleuritis, HIV, rheumatoid arthritis	[14,41,71,77,79,84,90,91,94,98,100,104,105,106,107,108,112,124,127,133,135,138,140,143,146,147,150]
*Ximenia caffra* Sond.	Shrub/tree	Roots, fruits, bark, leaves	Ulcers, wounds, asthma, livestock diseases, anthelmintic, diarrhea, toothache, painkiller, malaria, fevers, acute upper respiratory tract infections, dermatitis, ulcers, toothache, mouth infection, stomachache, abdominal pains	[14,68,72,74,76,77,80,118,138,141]
**Oleaceae**				
*Jasminum abyssinicum* Hochst. ex DC.	Liana	Roots, leaves, bark	Diarrhea, oral thrush, chest pains, weak joints, malaria, spinal cord aches, cough, aphrodisiac, wounds	[132,152]
*Jasminum dichotomum* Vahl	Liana	Leaves	Menstrual pains, epilepsy	[143]
*Jasminum fluminense* Vell.	Shrub/liana	Roots, leaves	Heartburn, headache, *Wuoyo*, snakebites, cough, chest/joint pains, insecticide, wounds, menstrual pains, leprosy	[71,76,77,86,99,142,143,168]
*Jasminum grandiflorum* L.	Liana	Leaves, roots, whole plant	Malaria, laxative, stomachache, diarrhea, anthelmintic, cough, chest pains, liver diseases, HIV/AIDs	[67,109,120,140,141,142,143]
*Jasminum streptopus* E.Mey.	Shrub/liana	Roots	Headache, snakebites	[77]
*Noronhia battiscombei* (Hutch.) Hong-Wa & Besnard	Shrub/tree	Bark	Malaria, back pains, anthelmintic	[77]
*Noronhia nilotica* (Oliv.) Hong-Wa & Besnard	Shrub/tree	Bark	Stomach disorders	[77]
*Olea capensis* L.	Tree	Bark, leaves, fruits, roots	Scabies, constipation, urinary infections, stomachache, peptic ulcers, tonic, colds, fever, livestock diseases, backache, diabetes, chest pains, mouth inflammation in children, malaria, pneumonia, general body weakness/fatigue/pains, amoeba, joint pains, anthelmintic, blood cleanser, cough, TB, difficulty in breathing, amoeba, arthritis, injuries/cuts/wounds, aphrodisiac, prostrate problems, rheumatism, skin cancer, acaricide, wounds	[14,41,66,72,88,90,99,103,104,117,120,125,131,133,136,138,146,147,152,168]
*Olea europaea* L.	Shrub/tree	Stems, bark, leaves, roots	Malaria, pneumonia, anthelmintic, retained afterbirth, respiratory problems, anaplasmosis, eye inflammation, stomachache, joint pains, chest pains, umbilical cord, livestock diseases, diarrhea, mouth infection in babies, fever, eye infections, seizures, ringworms, stomach rumbles, amoeba, back/bone problems, cough, colds/flu, dislocation, body pains, headache, boost immunity, injuries/cuts, wounds, internal injuries, appetizer, muscle fatigue, rheumatism, body swellings, typhoid, urinary diseases, liver disease, polio, gonorrhea, enlarged prostate, arthritis, skin disorders e.g., allergy, cancer, mellitus, itchy rashes, Contagious Bovine Pleuropneumonia in livestock, asthma, lumbago, helminthiasis (cow, poultry, goat, sheep, pig), bone fractures, acaricide, eye ailments, heartburn, sore throat, kidney problems, hypertension	[41,68,71,72,73,77,82,83,84,85,86,88,89,91,92,97,99,103,104,105,107,109,110,120,129,130,131,132,133,135,136,138,140,141,142,145,147,149,152,168]
*Olea welwitschii* (Knobl.) Gilg & G.Schellenb.	Tree	Bark, root, twigs	Scabies, chest problems, flu, gut problems, constipation, stomachache, childbirth, prenatal care, infertility, joint/backbone problems, STIs, dysentery, diarrhea, cancer, urinary tract infections	[116]
*Schrebera alata* (Hochst.) Welw.	Tree	Bark, twigs, leaves, roots	Malaria, chest problems, sore throat, toothache, diabetes mellitus, candidiasis, anesthesia, stomach pains, bloody stool, diarrhea, pregnancy pains, meat allergy, painkiller, poison antidote, headache, cuts	[41,67,68,71,77,85,136,140,142,145,152,168]
**Onagraceae**				
*Epilobium hirsutum* L.	Herb	Roots	Stomach pains	[84]
**Opiliaceae**				
*Opilia amentacea* Roxb.	Liana	Roots, bark, branches	Malaria, diarrhea, fever	[41,92,138,142]
*Pentarhopalopilia umbellulata* (Baill.) Hiepko	Shrub/liana	Roots	Venereal diseases, children’s diseases	[127]
**Orchidaceae**				
*Aerangis kirkii* (Rchb.f.) Schltr.	Herb	Leaves	Convulsions	[127]
*Angraecum sacciferum* Lindl.	Herb	Leaves	Pneumonia	[109]
*Ansellia africana* Lindl.	Herb	Branches	Eardrops	[99,127]
*Bonatea steudneri* (Rchb.f.) T.Durand & Schinz	Herb	Roots	Sores	[67]
*Eulophia petersii* (Rchb.f.) Rchb.f.	Herb	Roots	Prevent first trimester abortion	[81]
*Habenaria cirrhata* (Lindl.) Rchb.f.	Herb	Roots	Abdominal pains	[141]
*Microcoelia exilis* Lindl.	Herb	Leaves	Convulsions	[127]
*Solenangis wakefieldii* (Rolfe) P.J.Cribb & J.Stewart	Herb	Leaves	Convulsions	[127]
*Vanilla roscheri* Rchb.f.	Herb		Stomach pains, prenatal care	[127]
*Ypsilopus amaniensis* (Kraenzl.) D’haijère & Stévart	Herb	Root	Chest pain	[77]
**Orobanchaceae**				
*Asepalum eriantherum* (Vatke) Marais	Shrub	Whole plant	Backache	[142]
*Cistanche tubulosa* (Schenk) Wight ex Hook.f.	Herb		Childbirth problems	[72]
*Striga hermonthica* (Delile) Benth.	Herb	Leaves	Thrush	[143]
**Oxalidaceae**				
*Averrhoa bilimbi* L. (exotic)	Tree	Fruits	Aphrodisiac, appetizer	[150]
*Biophytum abyssinicum* Steud. ex A.Rich.	Herb		Rashes, smallpox	[132]
*Biophytum umbraculum* Welw.	Herb	Whole plant, leaves	Arthritis, dizziness, stomachache	[144,154]
*Oxalis corniculata* L.	Herb	Leaves	Skin infections, memory loss, mouth ulcers/sores, diseases of the eye	[122,123,141,154]
**Passifloraceae**				
*Adenia cissampeloides* (Planch. ex Hook.) Harms	Liana	Leaves, branches	Wounds	[99]
*Adenia ellenbeckii* Harms	Herb/shrub	Roots	Swollen legs and body pains during pregnancy	[139]
*Adenia gummifera* (Harv.) Harms	Liana	Stems, roots	Cough, gonorrhea, headache, miscarriage, aphrodisiac, STIs, female reproductive disorder, boost immunity, diarrhea, colds, dysmenorrhea	[14,77,100,127,142,150]
*Adenia kirkii* (Mast.) Engl.	Herb	Stems, roots	Complications in menstruation	[127]
*Adenia venenata* Forssk.	Liana	Roots	Cattle diarrhea	[14]
*Adenia volkensii* Harms	Herb/shrub	Fruits, roots	Poisonous	[77]
*Passiflora edulis* Sims (exotic)	Liana	Stems, leaves	Amoeba, cough, colds/flu, epilepsy, heart problems, insomnia, nervous system problems, poisoning, stomachache, rheumatism	[41,144,147,150]
*Passiflora quadrangularis* L. (exotic)	Liana		Amoeba	[41]
*Passiflora subpeltata* Ortega (exotic)	Herb	Leaves	Diabetes mellitus	[85]
**Pedaliaceae**				
*Sesamothamnus busseanus* Engl.	Shrub/tree	Whole plant	Bleeding	[142]
*Sesamum angolense* Welw.	Herb	Leaves	Measles	[99]
*Sesamum calycinum* Welw.	Herb	Leaves, roots, seeds	Sore eyes, hair relaxer, prevent first/second trimester abortion, vomiting, pregnancy edema, pregnancy anemia, stomachache, ringworm, constipation	[81,99,115,143,147]
**Oliniaceae**				
*Olinia rochetiana* A.Juss.	Shrub/tree	Bark, leaves, stems, roots	Respiratory, STIs, pneumonia, colds, cough, oral candidiasis, urinary tract infections, chest problems, TB, rheumatism, malaria, fever, bronchitis, indigestion, tapeworms, anodyne, expectorant, dysmenorrhea, abdominal pains	[67,77,88,99,105,113,120,131,135,140,141,142,147]
**Peraceae**				
*Clutia abyssinica* Jaub. & Spach	Shrub	Bark, leaves, roots	Menstrual cramps, spinal cord problem, joint pains, appetizer, arthritis, colds, rheumatism, liver pains, colic pain in infants, malaria, eye lotion, headache, stomachache, influenza, indigestion, liver pains, acaricide, erectile dysfunction, teeth problems, prophylaxis, pain in pregnancy, aphrodisiac, infertility, purgative, abdominal pains, diarrhea, kwashiorkor	[67,77,86,89,91,99,103,104,109,132,133,138,140,141,142,154,168]
*Clutia kilimandscharica* Engl.	Shrub	Roots, leaves, aerial parts, bark	Joints pains, diarrhea, typhoid, menstrual problems, colds, appetizer, general body weakness, anthelmintic, gonorrhea, constipation, stomachache, malaria, aphrodisiac, tonic, indigestion, rheumatism, acaricide, wounds	[67,104,120,131,133,142,152]
**Phyllanthaceae**				
*Antidesma venosum* E.Mey. ex Tul.	Shrub/tree	Roots, leaves, twigs, sap	Amoeba, back/joint/bone problems, blood purifier, cough, colds/flu, diabetes, immune booster, low libido, malaria, pneumonia, rheumatism, stomach disorders, tonsilitis, liver complaints, livestock diseases, abdominal pain, poisonous	[41,64,112,142,143,168]
*Bischofia javanica* Blume (exotic)	Tree	Roots	Stomach infections	[123]
*Bridelia cathartica* Bertol.	Shrub/tree	Leaves, roots	Convulsions, prenatal care	[127]
*Bridelia micrantha* (Hochst.) Baill.	Shrub/tree	Bark, roots, leaves	Boils, stomachache, STIs, dysentery, diarrhea, HIV/AIDs, back/joint/bone problems, blood purifier, brucellosis, cancer, chest problems, cough, colds/flu, cleans uterus, dizziness, hypertension, increase blood, malaria, pneumonia, rheumatism, skin diseases, typhoid, clean digestive system, tonic, livestock diseases, acidity, earache, cervical/breast/skin/colorectal cancer, dysentery, allergy, acaricide, epigastric distress, immune booster	[14,41,66,67,74,111,113,116,117,119,120,133,140,142,144,146,147,168]
*Bridelia scleroneura* Müll.Arg.	Shrub/tree	Stems, roots, bark	Acaricide	[133]
*Bridelia taitensis* Vatke & Pax	Shrub/tree	Leaves, stems, whole plant, bark	Cough, colds/flu, diarrhea, livestock diseases, earache, stomachache, laxativeabdominal pains, wounds	[14,51,80,139]
*Flueggea virosa* (Roxb. ex Willd.) Royle	Shrub	Leaves, roots, fruits, aerial parts	Malaria, venereal diseases, convulsions, stomachache, kidney pains, childbirth problems, amoeba, bilharzia, dental problems, meat allergy, prostate cancer, typhoid, irritated/itchy skin, cough, joint aches, chest pains, vomiting, headache, acaricide, boils, diarrhea, back pain, insect repellant, breast cancer, kidney problems, female reproductive disorders, aphrodisiac, HIV/AIDs, anthelmintic, fatigue, chickenpox, retained placenta	[14,41,75,77,80,81,84,101,112,119,122,127,133,139,142,143,146,147,150,168]
*Hymenocardia acida* Tul.	Shrub/tree	Bark, leaves	Acaricide	[133]
*Margaritaria discoidea* (Baill.) G.L.Webster	Shrub/tree	Bark	Acaricide, pregnancy pain	[133,140]
*Phyllanthus amarus* Schumach. & Thonn.	Herb	Roots	Arthritis	[154]
*Phyllanthus fischeri* Pax	Shrub	Leaves, fruits, stems, bark	Breast/skin/colorectal, skin diseases, roundworms, HIV/AIDs, gynecological diseases	[117,119,142,143]
*Phyllanthus kaessneri* Hutch.	Shrub/tree		Cough	[142]
*Phyllanthus muellerianus* (Kuntze) Exell	Shrub/tree	Leaves	Acaricide	[133]
*Phyllanthus nummulariifolius* Poir.	Shrub	Fruits	Roundworms, anthelmintic	[67,144]
*Phyllanthus odontadenius* Müll.Arg.	Herb	Roots	Stomach pains	[144]o
*Phyllanthus ovalifolius* Forssk.	Shrub/tree	Aerial parts	Acaricide, corneal ulcers	[133,143]
*Phyllanthus physocarpus* Müll.Arg.	Shrub/tree	Roots	STIs	[142]
*Phyllanthus reticulatus* Poir.	Shrub/tree	Roots, leaves	Convulsions, snakebites	[127]
*Phyllanthus sepialis* Müll.Arg.	Shrub	Leaves, roots	Malaria, constipation, general illness, toothache	[74,84,101]
*Phyllanthus somalensis* Hutch.	Shrub	Roots	Poisonous to livestock	[142]
*Phyllanthus welwitschianus* Müll.Arg.	Herb/shrub	Roots	Fertility potions, aphrodisiac	[134]
*Thecacoris spathulifolia* (Pax) Leandri	Shrub	Roots	Stomachache, diarrhea, induce salivation in children	[127]
**Phytolaccaceae**				
*Hilleria latifolia* (Lam.) H.Walter	Herb	Whole plant	Dermatitis	[144]
*Phytolacca dodecandra* L’Hér. (exotic)	Shrub	Leaves, roots, fruits	Fracture, antivenom, poisonous (human, livestock), breast cancer, body pains, stomach problems, childbirth, emetic, purgative, roundworms, tapeworms, syphilis, skin rashes, enlarged glands, abortifacient, barrenness	[72,77,86,99,120,138,141,142,143,154,168]
*Phytolacca octandra* L.	Shrub	Roots	Pneumonia	[152]
**Picrodendraceae**				
*Oldfieldia somalensis* (Chiov.) Milne-Redh.	Tree	Roots, bark	Pneumonia, arthritis, fatigue, cough, tuberculosis	[142,150]
**Pinaceae**				
*Pinus patula* Schiede ex Schltdl. & Cham. (exotic)	Tree	Leaves	Flu, respiratory	[116]
**Piperaceae**				
*Piper capense* L.f.	Herb/shrub	Leaves, roots, stems	Cough, colds, kidney problems, malaria, depression, anthelmintic, diarrhea, sore throat, enlarged uvula, increase milk letdown, arthritis, laxative, anthrax (cattle)	[14,97,99,125,147,168]
*Piper guineense* Schumach. & Thonn.	Shrub	Roots	Sore throat, fever, toothache	[142,144]
*Piper umbellatum* L.	Shrub	Roots, roots	Headache, fever, bronchitis, inflammation, rashes, eye inflammation, anthelmintic	[122,125,131,144]
**Pittosporaceae**				
*Pittosporum abyssinicum* Delile	Tree	Bark	Emetic, malaria, nausea, purgative, stomach problems	[131,142,168]
*Pittosporum viridiflorum* Sims	Shrub/tree	Roots, bark, leaves	Boils, STIs, malaria, diarrhea, stomachache, urinary tract, emetic, fever, purgative, tonic, measles, oral infections, fever, chest pains, amoeba, appetizer, constipation, acaricide, headache, vomiting, bronchitis, asthma, liver diseases, nose bleeding, rectum infection, infertility in goats, mosquito repellant	[66,67,77,99,101,103,104,109,116,119,120,132,133,140,141,142,144,152,168]
**Plantaginaceae**				
*Plantago palmata* Hook.f.	Herb	Roots	Tonsils, pneumonia, eye problems, venereal diseases, typhoid, diarrhea	[78]
**Plumbaginaceae**				
*Plumbago dawei* Rolfe	Herb/shrub	Bark	Diarrhea, malaria, stomach problems	[136]
*Plumbago zeylanica* L.	Herb/shrub	Root, aerial parts, roots, leaves	Boils, abscesses, burns, malaria, infertility, aphrodisiac, anthelmintic, remove pseudo teeth in children, skin blisters, swollen legs, stomachache, liver, anthrax, measles, sprained limps, skin diseases, fever, eye infection, gonorrhea, eye injury (cattle), lung/liver diseases (poultry)	[51,72,74,77,94,107,138,143,144,147]
**Poaceae**				
*Cenchrus americanus* (L.) Morrone	Herb	Bark, seeds	Chronic joint pains, malaria, vomiting, asthenia, swelling on the body	[14,130]
*Cenchrus clandestinus* (Hochst. ex Chiov.) Morrone	Herb	Leaves, roots	Skin infections, ringworms, heartburns, diarrhea, anthelmintic, jaundice, tonsils, urinary problems, venereal diseases, liver troubles	[41,88,109,115,168]
*Cenchrus hohenackeri* (Hochst. ex Steud.) Morrone	Herb	Bark, branches, roots	Malaria	[135]
*Cenchrus purpureus* (Schumach.) Morrone	Herb	Leaves	Allergies, dermatological infections	[41,143]
*Cymbopogon citratus* (DC.) Stapf (exotic)	Herb	Leaves	Headache, purify blood, clean pores, flu/colds, diarrhea	[108,119,143]
*Cymbopogon nardus* (L.) Rendle	Herb		Cough, colds/flu, fever, malaria, stomach disorders	[41]
*Cynodon dactylon* (L.) Pers.	Herb	Whole plant, leaves, stems	Kidney stones, stomachache, prevent second trimester abortion, vomiting	[81,96,106]
*Dactyloctenium aegyptium* (L.) Willd.	Herb	Leaves	Heartburn	[91]
*Digitaria abyssinica* (Hochst. ex A.Rich.) Stapf	Herb	Leaves	Ringworms, pain inside umbilical cord of adults, backache, urinary disorders	[147,152,168]
*Eleusine coracana* (L.) Gaertn. (exotic)	Herb	Seeds	Ringworm, cough, colds/flu, measles, chickenpox, itchy rash, kidney problems, diabetes	[41,94,138,143,146]
*Eleusine jaegeri* Pilg.	Herb	Leaves	Wounds, skin infections	[109]
*Elymus repens* (L.) Gould (exotic)	Herb		STIs, back/bone joints problems, chest problems, cough, colds/flu, gouts, malaria	[41]
*Enteropogon macrostachyus* (Hochst. ex A.Rich.) Munro ex Benth.	Herb	Whole plant	Trypanosomiasis	[68]
*Hyparrhenia diplandra* (Hack.) Stapf	Herb	Leaves	Insect repellant for safari ants	[144]
*Oldeania alpina* (K.Schum.) Stapleton	Tree	Fruits, shoots, stems	Balding, edema, blood cleanser, cough, colds/flu, mental ailments, pneumonia, rheumatism, STIs, malaria, diarrhea, aphrodisiac, impotency, retained placenta (cattle, human), menstruation problems	[14,41,88,132,147,152,168]
*Oryza sativa* L. (exotic)	Herb	Roots	Colds	[14]
*Saccharum officinarum* L. (exotic)	Herb	Leaves, roots, exudate	Acaricide, malaria, baby stomach problems	[14,133,139]
*Sorghum bicolor* (L.) Moench (exotic)	Herb	Seeds	Measles, energy boost, acaricide, gastrointestinal problems, diarrhea, colds	[14,41,88,133,143]
*Sporobolus pyramidalis* P.Beauv.	Herb	Roots	Gonorrhea, stomach ailments	[113,147]
*Triticum aestivum* L. (exotic)	Herb		Cancer	[41]
*Zea mays* L. (exotic)	Herb	Cobs, flowers	Cough, heartburn, abdominal ulcers, retained placenta (cows, human), acaricide, urinary disorders in men	[73,88,133,139,152,168]
**Podocarpaceae**				
*Afrocarpus falcatus* (Thunb.) C.N.Page (exotic)	Tree	Bark, roots, leaves, twigs	General body health, fever, malaria, liver and spleen diseases, peptic ulcers, stomachache, measles, acaricide, hypertension, arthritis, anodyne, itchy skin rash, chest troubles	[14,67,68,88,91,109,118,132,133,135,141,168]
*Podocarpus latifolius* (Thunb.) R.Br. ex Mirb. (exotic)	Tree	Bark, leaves, roots	Malaria, stomachache, dysmenorrhea, cleanse womb, afterbirth, asthma, gall bladder, indigestion, acaricide, chest troubles	[41,67,88,97,120,133,142,152,168]
**Polygalaceae**				
*Carpolobia goetzei* Gürke	Shrub/tree	Roots, leaves	Aphrodisiac	[127]
*Polygala erioptera* DC.	Herb		Cough, whitlow, skin rashes	[141]
*Polygala persicariifolia* DC.	Herb	Leaves	Eye and ear diseases	[141]
*Polygala sphenoptera* Fresen.	Herb	Leaves, stem, roots	Infertility in women, eye inflammation, stomachache, cough, aphrodisiac	[99,152,168]
*Securidaca longepedunculata* Fresen.	Shrub/tree	Roots, bark, leaves	Body swellings, chest pains, amoeba, asthma, back/bone problems, cough (human, cattle), colds/flu, general body pains, malaria, mental disorders, tonsilitis, anthelmintic, acidity, stomach ailments, delayed delivery, purgative, dizziness, headache, acaricide, vaginal discharge, menstrual cramps, infertility, aphrodisiac, joint pains, sores, toothache, retained placenta, abdominal pains	[14,41,74,111,118,129,133,139,142,146,147,150,168]
**Polygonaceae**				
*Oxygonum sinuatum* (Hochst. & Steud. ex Meisn.) Dammer	Herb	Roots, leaves, whole plant	Tonsillitis, conjunctivitis, boils, cellulitis, toothache, diabetes mellitus, rashes, dandruffs, burns, skin/throat cancer, body swellings, wounds, skin infection, stomachache, urinary problems, gonorrhea, typhoid, venereal diseases, joint pains, eye diseases, enlarged uvula	[76,84,85,90,94,106,115,117,118,143,147,168]
*Persicaria setosula* (A.Rich.) K.L.Wilson	Herb	Leaves	Revive the dying	[67]
*Rumex abyssinicus* Jacq.	Herb	Leaves, roots	Malaria, menstrual problems, stomachache, conjunctivitis (cow, poultry, goat, sheep, pig), eye infections, general wellbeing, scabies, anthelmintic	[41,67,73,101,119,168]
*Rumex acetosella* L.	Herb	Roots	Abdominal pains, hypertension, diabetes	[86,91]
*Rumex hypogaeus* T.M.Schust. & Reveal	Herb	Roots	Venereal diseases	[168]
*Rumex nepalensis* Spreng.	Herb	Leaves, roots	Malaria, stomachache, fever, snakebites paralysis, sore throat, livestock diseases (infection/stomach)	[101,144,152]
*Rumex usambarensis* (Engl.) Dammer	Shrub	Leaves, roots	Anthelmintic, constipation, joint pains, vomiting, urinary tract infections, cough, chest pain, difficulty in breathing	[81,105,119,122,130]
**Portulacaceae**				
*Portulaca foliosa* Ker Gawl.	Herb	Whole plant	Wounds, burns, swollen’ belly in children	[77,140]
*Portulaca oleracea* L.	Herb	Whole plant	Wounds, burns, typhoid, cancer, diarrhea, urinary disorders, liver/kidney problems	[77,91,115,168]
*Portulaca quadrifida* L.	Herb	Whole plant	Wound, burns	[77,91]
**Primulaceae**				
*Embelia schimperi* Vatke	Shrub/liana	Stem, bark, leaves, roots, seeds	Joint pains, chest pain, malaria, colds, anthelmintic, wounds, stomach problems, fever, rheumatic fever, tuberculosis, pneumonia, brucellosis, diarrhea	[107,109,131,142,168]
*Lysimachia volkensii* Engl.	Herb		Wounds	[136]
*Maesa lanceolata* Forssk.	Shrub/tree	Roots, fruits, bark, seeds, leaves	Flu, anthelminthic, appetizer, stomachache, STIs, malaria, backache, arthritis, snakebites, skin allergies, heartburn, chest pain, boils, colic pains, headache, abdominal pains, pregnancy, kidney diseases, acaricide, cough, fontanel inflammation or wounds, veterinary use as poison antidote, astringent, purgative, dysmenorrhea, itchy skin rashes, enlarged glands, abortifacients, fever (humans, animals), sore throat, heart problems, gonorrhea, healing umbilical scar, dysentery	[77,79,88,91,92,99,101,116,120,122,126,133,141,142,144,145,146,168]
*Myrsine africana* L.	Shrub/tree	Bark, fruits, leaves, seeds	Gonorrhea, constipation, headache, malaria, respiratory disorders, anthelmintic (human, livestock), heartburn, tuberculosis, diabetes, bone/joint pain, kidney diseases, helminthiasis, wounds, tonic, tranquillizer, fever, typhoid, tuberculosis, stomach problems, dysentery, chest pain, abdominal disorders, backache, dysmenorrhea, internal boils due to injuries or inflammatory diseases, purgative, copious perspiration, relaxation, appetite, measles, stomachache	[67,68,72,77,83,88,89,105,132,135,136,137,138,140,141,147,149,168]
*Myrsine melanophloeos* (L.) R.Br. ex Sweet	Tree	Fruits, seeds, bark	Anthelmintic, gonorrhea, heartburn, weak bones, chest pains, joint pains, diarrhea, malaria, general body weakness, pneumonia, stomachache, colds, rumbling stomach, hypertension, livestock diseases, purgative, dysmenorrhea, afterbirth pains, amoeba, back/bone pains, blood purifier, constipation, injuries/cuts, wounds, kidney disorders, appetizer, prostate cancer, rheumatism, skin diseases, typhoid, headache, purgative, arthralgia, body pain, fever	[14,41,67,68,88,99,105,107,109,120,131,132,141,142,145,146,147,152]
**Proteaceae**				
*Faurea rochetiana* (A.Rich.) Chiov. ex Pic.Serm.	Tree	Bark	Chronic cough	[140]
*Faurea saligna* Harv.	Shrub/tree	Roots, bark	Stomach upsets/ulcers, urinary tract infections, indigestion, tonic, asthma, back/joint/bone problems, chest problems, cough, colds/flu, general body pains, stomach disorders, diarrhea, menstrual pains, stomachache, headache, itchy skin rash, abdominal pain	[41,67,77,91,99,105,131,132,135,141,142,168]
*Grevillea robusta* A.Cunn. ex R.Br. (exotic)	Tree	Bark, roots	Back/joint/bone problems, cough, colds/flu, anthelmintic, sore throat, ear, chest problems, toothache, immune booster, antidote	[14,41,116]
*Macadamia tetraphylla* L.A.S.Johnson (exotic)	Tree	Bark	Allergies, skin cancer	[41,117]
**Putranjivaceae**				
*Drypetes gerrardii* Hutch.	Tree	Leaves, roots	Wounds, acaricide	[133,147]
**Ranunculaceae**				
*Clematis brachiata* Thunb.	Liana	Roots, leaves, flowers, stems	Mouth inflammation, stomachache, chest pains, colds, headache, backache, syphilis, gonorrhea, fever, arthritis, cough, malaria, amoeba, stomach ailments, constipation, depression, flu, bleeding gums, toothache, body swellings, nasal congestion, snuff	[77,97,103,104,122,131,140,142,143,146,147,152,154,168]
*Clematis hirsuta* Guill. & Perr.	Liana	Roots, leaves, bark	Headache, colds, malaria, fever, ear problem, chest problem, diabetes, anthelmintic, antidote	[14,67,83,98,104,109,146]
*Clematis simensis* Fresen.	Liana	Roots, stems, leaves, exudate, flowers	Sinusitis, allergy, colds, cough, wheezing, chest pain, blocked nose, headache, wounds, mouth inflammation, stomachache, chest pains, malaria, purgative, rashes, meningitis, anodyne, abdominal pains, oral thrush, bleeding gums	[67,86,87,132,141,142,152,168]
*Clematis villosa* DC.	Herb		Stuffy nose and associated respiratory problems	[125]
*Hydrastis canadensis* L. (exotic)	Herb		Painkiller	[41]
*Nigella sativa* L. (exotic)	Herb	Seeds	Headache, hypertension, stomachache	[137]
*Ranunculus multifidus* Forssk.	Herb	Roots, seeds, leaves	Pneumonia, malaria, oral thrush, purgative, expectorant	[67,141]
*Thalictrum rhynchocarpum* Quart.-Dill. & A.Rich.	Herb	Roots	Purgative, expectorant	[141]
**Resedaceae**				
*Caylusea abyssinica* (Fresen.) Fisch. & C.A.Mey.	Herb	Roots, whole plant	Abdominal pains, anthelmintic, cough, abdominal pains	[99,140,141]
**Rhamnaceae**				
*Berchemia discolor* (Klotzsch) Hemsl.	Shrub/tree	Roots, bark	Diarrhea, men infertility, stomach disorder, liver ailments, erectile dysfunction, typhoid, fever	[14,77,80,86,91,142]
*Helinus integrifolius* (Lam.) Kuntze	Liana	Bark	Arthritis, paralysis	[68]
*Helinus mystacinus* (Aiton) E.Mey. ex Steud.	Liana	Leaves, bark, roots, shoots	Respiratory disorders, snakebites, stomachache, animal fever, anthrax, rinderpest, headache	[77,88,139,144]
*Maesopsis eminii* Engl.	Tree	Bark	Cough	[122]
*Rhamnus prinoides* L’Hér.	Shrub/tree	Roots, seeds, bark, leaves	Splenomegaly, infertility, cancer, syphilis, gonorrhea, arthritis, stomachache, pneumonia, brucellosis, indigestion, tonic, backache, arthritis, prostate, malaria, postpartum weakness, dizziness, dirty circulatory system, headache, waist pains, constipation, weak joints, body weakness, fever, diarrhea, appetizer, amoeba dysentery, abdominal cramps, joint pains, allergies, amoeba, bone problems, blood cleanser, cough, colds/flu, dental problems, rheumatism, typhoid, anthelmintic, colic pain in infants, renal disorders, skin disorders, snakebites, neck stiffness, diabetes, abortifacient, chest problems, pleuritis, purgative, epilepsy, mouth diseases	[14,41,67,72,77,83,86,87,88,89,90,92,97,99,103,104,105,107,109,110,113,131,132,135,136,138,141,142,146,147,149,152,154,168]
*Rhamnus staddo* A.Rich.	Shrub/tree	Roots, bark, leaves, stems	Tonic, STIs, flu/colds, diabetes, endometritis, diarrhea, chest pains, heartburn, stomachache, toothache, cough, malaria, gonorrhea, syphilis, diabetes, chest pain, backache, fever, pneumonia, bone problems, dental problems, headache, rheumatism, arthritis, venereal diseases, polio, body aches, fever, hepatitis, menstrual problems, livestock diseases, anaplasmosis, typhoid, male’s libido, vomiting in babies, epilepsy/tetanus, infertility, hypertension, joint diseases, rheumatic pains, kidney problems	[41,67,68,71,72,77,92,97,99,103,104,105,107,109,110,118,121,135,136,138,140,141,142,147,149,152,154,168]
*Scutia myrtina* (Burm.f.) Kurz	Shrub/tree	Roots, bark, leaves, fruits	Boosting immunity, stomachache, anthelmintic, joint pains, prenatal care, tonic, pneumonia, respiratory disorders, joint ache, retained afterbirth, allergies, anemia, pregnancy pains, measles, infertility, epilepsy, dizziness, fever, ringworm, menstrual problems	[68,71,77,88,127,130,131,132,135,138,140,141,143,147,151,152]
*Ziziphus abyssinica* Hochst. ex A.Rich.	Shrub/tree	Roots, bark, leaves, aerial parts	Burns, kidney, asthma, pneumonia, Contagious Bovine Pleuropneumonia in livestock, malaria, stomach disorders, acaricide	[77,82,104,120,133,153]
*Ziziphus jujuba* Mill.	Shrub/tree	Roots, leaves, bark	Tuberculosis, indigestion, diarrhea, indigestion, cough, abdominal pains, colic pains, stomachache, dysentery, catarrhs, amoeba, rheumatism, retained placenta	[86,99,112,132,139,141,142]
*Ziziphus mucronata* Willd.	Shrub/tree		Abdominal pains, prenatal care, body inflammation, snakebite, arthritis, stomachache, diarrhea, acaricide, wounds, bleeding in miscarriage, fecal impaction in babies/children, heartburn, pain in pregnancy, rheumatism, joint diseases	[67,71,77,80,87,89,99,127,133,140,141,148,149]
**Rhizophoraceae**				
*Cassipourea celastroides* Alston	Tree	Leaves	Body wellness	[139]
*Cassipourea euryoides* Alston	Shrub/tree	Bark	Aphrodisiac, rheumatism	[150]
*Cassipourea malosana* (Baker) Alston	Shrub/tree	Roots	Malaria, pneumonia, cancer, fibroids, wounds	[41,152]
**Rosaceae**				
*Alchemilla abyssinica* Fresen.	Herb	Leaves	Backache, spinal cord problems, eye infection, bruises, malaria	[109]
*Hagenia abyssinica* (Bruce) J.F.Gmel.	Tree	Flowers, bark, aerial parts	Infertility in women, livestock diseases, amoeba, cough, colds, flu, headache, rheumatism, typhoid, stomachache, diarrhea, anthelmintic, allergy, gout, abdominal pains	[41,67,109,132,141,142,152,168]
*Prunus africana* (Hook.f.) Kalkman	Tree	Bark, roots, fruits, leaves, stems	Eye problems, joint pains, prostate cancer, stomachache, urinary tract infections, back pain in women, HIV/AIDs, washing newborns to shed off dead skin, appetizer, fever, malaria, vomiting, diarrhea, postmenopausal syndrome, allergies, arthritis, amoeba, back/bone problems, blood cancer, blood cleanser, brucellosis, cancer, cough, colds/flu, dental problems, diabetes, epilepsy, family planning, fibroids, injuries, cuts/wounds, heart problems, increase blood, indigestion, meat allergy, pneumonia, rheumatism, tonsilitis, tumors, typhoid, ulcers, anthelmintic, chest pains, general body weakness, peptic ulcers, hypertension, rheumatism, brain fatigue, congested chest, anthrax, body ache, joint ache, cough (cow, goats, sheep, poultry, pig, ruminants), tonic, colorectal/breast/skin/prostate cancer, STIs, acaricide, purgative, aphrodisiac, abdominal pains, astringent, gonorrhea, breast cancer, liver diseases, indigestion, meat allergy, anaplasmosis in cattle, toothache, arthritis	[14,41,66,67,73,74,77,83,84,86,88,89,91,95,103,104,105,107,109,113,116,117,120,125,131,132,133,135,136,141,142,145,146,147,152,168]
*Rhaphiolepis bibas* (Lour.) Galasso & Banfi (exotic)	Shrub	Roots, fruits, bark	Kwashiorkor, marasmus, diabetes, stomachache, toothache, earache	[41,119,144]
*Rubus apetalus* Poir.	Shrub	Roots, leaves, fruits	Blood cleanser, malnutrition, prophylaxis of cancer, cough, colds, STIs, joint ailment, stomachache	[88,144,147]
*Rubus keniensis* Standl.	Shrub	Roots	Stomachache, STIs, afterbirth problems	[120,131]
*Rubus pinnatus* Willd.	Tree	Roots, leaves	Malaria, diarrhea, stomachache, amoeba, gonorrhea	[41,101,146,152]
*Rubus steudneri* Schweinf.	Shrub	Roots	Cough, abdominal pains, stomachache	[99,132]
*Rubus volkensii* Engl.	Shrub	Fruits	Cough, colds, acidity, general body health	[147]
**Rubiaceae**				
*Afrocanthium keniense* (Bullock) Lantz	Shrub/tree	Bark	Liver problems	[168]
*Afrocanthium lactescens* (Hiern) Lantz	Tree	Roots, bark	Backache, purgative	[140,141]
*Agathisanthemum bojeri* Klotzsch	Herb/shrub	Roots, leaves	Prenatal care, convulsions, skin infections, colds, stomachache, sore eyes, ears, anthelmintic	[75,112,127]
*Agathisanthemum globosum* (Hochst. ex A.Rich.) Klotzsch	Herb	Roots	Malaria	[111]
*Breonadia salicina* (Vahl) Hepper & J.R.I.Wood	Tree	Roots, bark	Malaria, headache	[74]
*Canthium glaucum* Hiern	Shrub	Fruits	Malaria	[111]
*Catunaregam nilotica* (Stapf) Tirveng.	Shrub/tree	Roots	Constipation, stomach ailments	[112,127]
*Coffea arabica* L.	Shrub/tree	Roots	Back pain in women, allergies, appetizer, low libido, pneumonia, pregnancy problems, rheumatism, sleeping sickness, flu	[41,113,146]
*Coffea eugenioides* S.Moore	Tree	Leaves	Eye problems in livestock, heavy menstruation	[116,125]
*Conostomium quadrangulare* (Rendle) Cufod.	Herb	Leaves, roots	Purgative, lice infestation	[141]
*Coptosperma graveolens* (S.Moore) Degreef	Shrub/tree	Bark, roots, stems, leaves	Malaria, aphrodisiac, erectile dysfunction, eyedrops, ulcers, sores, smallpox lesions, swollen legs and body pains during pregnancy	[104,119,134,139,142]
*Craterispermum schweinfurthii* Hiern	Shrub/liana	Bark	Aphrodisiac, diarrhea, malaria, veterinary	[116]
*Dirichletia glaucescens* Hiern	Shrub	Leaves	Insecticide e.g., termites	[84]
*Dolichopentas longiflora* (Oliv.) Kårehed & B.Bremer	Herb	Bark, roots, leaves	Laxative, skin, malaria, purgative, back pains, lice infestation, warts	[67,91,115,120,141,168]
*Galium aparinoides* Forssk.	Herb	Roots, whole plant	Stomach ulcers, urinary infections, throat cancer in cattle, anaplasmosis in cattle, nervous and urinary disorders	[89,105,168]
*Galium simense* Fresen.	Herb	Leaves	Cancer, general body rashes	[131]
*Galium spurium* L.	Herb	Roots, leaves	Stomach disorders, false teeth	[109]
*Gardenia posoquerioides* S.Moore	Shrub/tree		Syphilis	[142]
*Gardenia ternifolia* Schumach. & Thonn.	Shrub/tree	Roots, fruits, bark	Malaria, purgative, acaricide, emetic, eyes, amoeba, vomiting, cough, pneumonia, epigastric pain, appetizer, heartburn, chest pain, mental illness	[67,71,72,98,132,133,140,142,143,148]
*Gardenia transvenulosa* Verdc.	Shrub/tee	Roots	Stomach ailments	[142]
*Gardenia volkensii* K.Schum.	Shrub/tree	Roots, fruits, bark	Emetic, back/joint/bone problems, burns, malaria, STIs, snakebite, breast/uterine/skin cancer, purgative, colds, sores, cough, stomachache, anthelmintic	[14,41,86,87,117,142]
*Hymenodictyon parvifolium* Oliv.	Shrub/tree	Roots, leaves	Constipation, diarrhea, snakebites, livestock diseases, ears sores, sooth red eyes, ulcer pains, aid conception, edema	[51,64,96,100,105]
*Keetia gueinzii* (Sond.) Bridson	Shrub/liana	Bark, roots	Asthma, pneumonia, cough, allergy, joint pains, appetizer, fatigue, diarrhea	[98,144]
*Lamprothamnus zanguebaricus* Hiern	Shrub/tree	Roots	Diarrhea	[127]
*Lasianthus kilimandscharicus* K.Schum.	Shrub/tree	Roots	Malaria, colds	[14]
*Meyna tetraphylla* (Schweinf. ex Hiern) Robyns	Shrub/tree	Roots	Alleviate pain in pregnant women	[142]
*Mussaenda arcuata* Poir.	Shrub	Leaves, bark	Joint pains, venereal diseases	[144]
*Oxyanthus speciosus* DC.	Shrub/tree	Bark	Sore throat	[72]
*Pavetta abyssinica* Fresen.	Shrub/tree	Roots, leaves, bark	Colorectal/skin/breast cancer, purgative, abdominal pains	[91,117,141]
*Pavetta crassipes* K.Schum.	Shrub/tree	Roots, leaves	Chronic joint pains, asthma, acaricide	[118,130,133]
*Pavetta oliveriana* Hiern	Shrub/tree	Leaves, roots	Acaricide, aphrodisiac, abdominal pains, meningitis	[133,141]
*Pavetta subcana* Hiern	Shrub	Roots, whole plant	Gonorrhea, cough (cows), fleas (cattle)	[67,89,142]
*Pavetta teitana* K.Schum.	Shrub/tree	Leaves, roots	Pneumonia	[74]
*Pentanisia ouranogyne* S.Moore	Herb	Roots	Tonic, appetizer in babies/children, malaria	[67,111,140]
*Pentas lanceolata* (Forssk.) Deflers	Herb/shrub	Roots, bark	Joint aches, malaria, kidney problems, scurvy, depression, arthritis, venereal disease	[97,104,147]
*Pentas pubiflora* S.Moore	Herb/shrub	Roots	Purgative	[99,141]
*Pentas zanzibarica* (Klotzsch) Vatke	Herb/shrub	Roots, leaves	Malaria, tonic	[41,67,84]
*Pentodon pentandrus* (Schumach. & Thonn.) Vatke	Herb	Roots	Anthelmintic, pneumonia, cough	[74]
*Polysphaeria multiflora* Hiern	Shrub/tree	Leaves	Sore muscles, wounds	[142]
*Psychotria amboniana* K.Schum.	Shrub	Leaves, roots	Convulsions, headache	[127,142]
*Psychotria faucicola* K.Schum.	Shrub	Roots	Diarrhea	[142]
*Psychotria holtzii* (K.Schum.) E.M.A.Petit	Shrub	Leaves	Colds	[142]
*Psychotria kirkii* Hiern	Shrub	Roots	Malaria, meat allergy	[136,140]
*Psychotria lauracea* (K.Schum.) E.M.A.Petit	Shrub/tree	Roots, leaves	Childbirth, given to women after birth	Glover et al. 1966[14,67,142]
*Psychotria orophila* E.M.A.Petit	Shrub/tree		Malaria	[136]
*Psydrax parviflorus* (Afzel.) Bridson	Shrub/tree		Back/joint/bone problems, diarrhea	[41]
*Psydrax schimperianus* (A.Rich.) Bridson	Shrub/tree	Shoots, roots, bark	Breast cancer, diarrhea, wounds, strengthen bones, general body weakness, abdominal pains, backache, joint pains	[117,132,140,141,144,168]
*Pyrostria phyllanthoidea* (Baill.) Bridson	Shrub/tree	Roots, bark	Dizziness, weak bones, bad breadth, retained placenta	[139,140]
*Rhodopentas bussei* (K.Krause) Kårehed & B.Bremer	Herb/shrub	Roots	Venereal diseases	[142]
*Rhodopentas parvifolia* (Hiern) Kårehed & B.Bremer	Shrub	Roots, bark	Vomiting, malaria, constipation in children	[81,129,140]
*Rubia cordifolia* L.	Herb	Roots, leaves, whole plant	Mouth inflammation, gonorrhea, diarrhea, vomiting, pneumonia, cough, whooping cough, skin infection, heartburn, oral thrush, livestock diseases, malaria, upper respiratory tract infections, ringworms, oral infections, anthelmintic, stomachache, hypertension, chest pain, dysentery, urinary disorders	[68,77,86,99,104,109,115,119,120,123,138,140,141,143,152,168]
*Rytigynia acuminatissima* (K.Schum.) Robyns	Shrub/tree	Bark	Arthritis	[154]
*Spermacoce princeae* (K.Schum.) Verdc.	Herb	Roots, leaves	Breast/colorectal/skin cancer, boils	[117,119]
*Tarenna pavettoides* (Harv.) Sim	Shrub/tree	Leaves	Correct fetus during parturition	[144]
*Tennantia sennii* (Chiov.) Verdc. & Bridson	Shrub	Roots	Diarrhea, malaria, impotence and infertility	[51]
*Uncaria africana* G.Don	Shrub/liana	Roots, bark	Stomach disorder, strength	[77]
*Vangueria apiculata* K.Schum.	Shrub/tree	Leaves, roots, twigs	Prolonged menstruation, arthritis, veterinary uses, body swellings, warts, tuberculosis	[66,116,144,154]
*Vangueria infausta* Burch.	Shrub/tree	Roots, leaves, fruits	Snakebites, prenatal care, cough, colds, body pains, anthelmintic, pneumonia (cow, goat, sheep, poultry), stop menses, fever, eye problems	[14,73,74,105,112,116,127,147]
*Vangueria madagascariensis* J.F.Gmel.	Shrub/tree	Leaves, roots, bark, shoots	Mouth infection in babies, stomach complications, brucellosis, cholera, heartburn, loin pains, medicine antidote, pancreatic diseases, prostrate problems, rheumatism, typhoid, arthritis, men infertility, malaria, colic pain, anthelmintic (human, animals)	[41,67,84,86,103,104,109,140,146,154]
*Vangueria volkensii* K.Schum.	Shrub/tree		Malaria, amoeba	[41,124]
**Rutaceae**				
*Calodendrum capense* (L.f.) Thunb.	Tree	Bark	Stomach upsets, emetic, fever, bleeding wounds (human, sheep)	[105,109,145]
*Citrus × aurantiifolia* (Christm.) Swingle	Shrub/tree	Leaves, fruits	Replacement milk for nursing mothers, colds, flu	[108]
*Citrus × aurantium* L.	Shrub/tree	Roots, leaves, fruits, bark	Cough, colds/flu, arthritis, cancer, low libido, nose bleeding, rheumatism, eye ache	[41,146,150]
*Citrus × limon* (L.) Osbeck	Shrub/tree	Fruits, leaves	Malaria, stomachache, flu, colds, ringworm, asthma, back/joint/bone problems, blood cleanser, chest congestions/pains, ear-nose-threat infections, appetizer, rheumatism, typhoid, skin rashes, pneumonia, prostate cancer, infertility, anthelmintic	[14,41,74,94,106,108,143,146,150]
*Clausena anisata* (Willd.) Hook.f. ex Benth.	Shrub/tree	Leaves, roots, bark	Malaria, anthelmintic, stomach problems, prostate, convulsions, cough, colds, mouth infection in babies, emetic, blood cleanser, stroke, whooping cough, body pains, constipation, poisoning, diarrhea, pneumonia, teeth cleansing, protracted labor, retained placenta, postpartum hemorrhage, headache, fever, indigestion, toothache, kidney/heart diseases, livestock diseases, vertigo, abdominal pains, typhoid, chest pain, respiratory problems, measles, chest diseases, tonsillitis, afterbirth pains, menstrual problems	[14,74,81,84,88,99,101,103,104,105,109,111,120,121,127,138,141,142,143,144,146,147,150,168]
*Fagaropsis angolensis* (Engl.) H.M.Gardner	Tree	Leaves, roots, bark	Chest problems, gut problems, induces menopause, amoeba, bone problems, cough, colds, flu, diarrhea, dysentery, livestock diseases, malaria, rheumatism, stomach disorders, typhoid, anthelmintic, joint pains, stomachache, headache, chest problems, STIs, expectorant, abdominal pains, backache	[41,66,74,84,93,103,104,111,116,141,146,147]
*Fagaropsis hildebrandtii* (Engl.) Milne-Redh.	Shrub/tree	Roots, bark, leaves	Morning sickness, women’s infertility, malaria, asthma, ulcers, chronic joint pains, abscesses, arthritis, cough, colds/flu, postpartum hemorrhage, milk letdown, prevent first/second trimester abortion, vomiting, pregnancy anemia, swollen legs and body pains during pregnancy	[41,81,94,102,129,130,139]
*Harrisonia abyssinica* Oliv.	Shub/tree	Bark, leaves, roots	Gonorrhea, syphilis, malaria, arthritis, STIs, stomach pains, body pains, diarrhea, stomachache, pneumonia, infertility, eye ointment, ringworms, blood purifier, cough, chest congestion, helminthiasis, amoeba, abscess, ECF, lumbago, rheumatism, retained placenta, inducing abortion, acaricide, headache, pregnancy problems, typhoid, chest problems, asthma, abdominal pains, back ache/joint ache, wounds, swollen breasts and testicles, boils, pregnancy problems, bubonic plague, anthelmintic, kidney problems, diabetes, menstrual problems	[14,41,66,68,72,74,76,77,79,80,84,90,93,98,100,102,103,105,108,111,112,118,124,127,129,132,133,138,141,142,143,146,147,153]
*Toddalia simplicifolia* Engl.	Shrub/tree	Roots, bark, leaves	Pneumonia, allergy, gonorrhea, diarrhea, malaria, stomachache, weak bones, rib bones, eye problem, colds, flu, joint pains, skin rashes, chest troubles, poisoning, diabetes, cerebral malaria, fevers, hepatitis, fever, oral trash, wounds, typhoid, firm stool, stomach acid, tonic, blood pressure, joint diseases, swollen legs and body pains during pregnancy, edema	[51,67,68,71,77,83,84,96,99,100,103,104,105,111,128,129,136,139,147,149,152]
*Vepris eugeniifolia* (Engl.) I.Verd.	Shrub	Bark leaves	Hepatitis, sore throat, malaria, body pains	[72,77]
*Vepris glomerata* (F.Hoffm.) Engl.	Shrub/tree	Roots	Colds, snakebites	[127]
*Vepris nobili*s (Delile) Mziray	Shrub/tree	Bark, roots, leaves	Toothbrush, brucellosis, aphrodisiac, livestock diseases, malaria, headache, joint pains, colds, pneumonia, anthelmintic, chest pain, arthritis, backache, blood cleanser, invigorant, stimulate immunity, stomachache, labor, back/bone problems, nausea, rheumatism, amoeba, acaricide, asthma, tonic, dizziness, cough, expectorant, abdominal pains, internal body pains/weakness, anthrax	[41,67,84,86,88,89,98,104,109,119,120,133,135,136,140,141,143,145,152]
*Vepris samburuensis* Kokwaro	Shrub/tree		Liver problems	[136]
*Vepris sansibarensis* (Engl.) Mziray	Shrub/tree	Roots	Snakebites, body inflammation, reproductive disorder	[127,150]
*Zanthoxylum asiaticum* (L.) Appelhans, Groppo & J.Wen	Shrub/tree	Leaves, roots, bark, stems, fruits	Abdominal pains, gynecological disorders including infertility, cough, colds, cancer, renal disorders, anthelmintic in cattle, malaria, backpain in women, mouth inflammation, joint pain, boils, chest pains, general body weakness, headache, fever, mouth infection in babies, pneumonia, stomachache, sore throat, tonic, respiratory disorders, TB, urinary tract problems, arthritis, cancer, throat cancer, joint aches, food poisoning, rheumatism, asthma, irregular menstruation, back pains, indigestion, kidney problems, typhoid, amoeba, aphrodisiac, firms stool, abdominal pains, emesis in livestock	[14,67,71,72,79,82,86,87,88,89,98,99,103,107,109,115,118,119,120,123,124,125,129,131,132,134,135,136,140,141,143,144,147,150,152,154,168]
*Zanthoxylum chalybeum* Engl.	Shrub/tree	Roots, bark, leaves, seeds, fruits	STIs, flu/cold, indigestion, throat infection, pain in fallopian tubes, asthma, pneumonia, malaria, ulcers, fever, general body pains, gastric lesions and pain, urinary tract infections, venereal diseases, bleeding gums, chest pains, stomach problems, anthrax, measles, epilepsy, psychotic disorders, oral thrush, emetic, sore throat, livestock diseases, joint pains, cholera, allergies, back/bone problems, constipation, cough, dental problems, gouts, headache, injuries, cuts/wounds, rheumatism, ringworms, TB, ulcers, sores, diabetes mellitus, chest congestion, mouth sores/ulcers, freshens the mouth, anthelmintic, toothache, delayed labor, first trimester abortion, vomiting, pregnancy edema, snakebite, keep gums hard and healthy, amoeba, mouth ulcers, headache, typhoid, stomachache, waterborne diseases, body pains, boils, muscular pains, diarrhea, abdominal pains, epilepsy, peptic ulcers, obesity, reproductive disorders, anthrax, immune booster, cattle cough, swollen legs and body pains during pregnancy, retained placenta, kidney problems, edema	[14,41,51,64,72,74,76,77,79,80,81,84,85,86,88,91,92,96,98,99,100,102,103,106,109,111,112,127,128,129,130,131,132,134,138,139,141,142,143,146,147,148,150,151]
*Zanthoxylum gilletii* (De Wild.) P.G.Waterman	Tree	Bark, leaves, seeds, roots	Asthma, bronchitis, sore throat, colic pain, constipation, hepatomegaly, severe body inflammation, tongue, anthrax, cough, colds, chest problems, malaria, rheumatism, joint aches, skin cancer, scabies, gut problems, buccal/oral problems, headache, cardiac conditions, gout, veterinary uses, asthma, pneumonia, body malaise, fevers, anaplasmosis in cattle, toothache, bleeding gums, snakebites, headache, prostate cancer, dental problems, arthralgia, joint pains, fever, venereal diseases, stomachache, scratches on body, wounds	[14,66,87,98,101,116,117,125,142,143,144,147,168]
*Zanthoxylum holtzianum* (Engl.) P.G.Waterman	Shrub/tree	Leaves, roots, bark	Ulcer, cough	[145]
*Zanthoxylum rubescens* Planch. ex Hook.	Shrub/tree	Bark, leaves	Breast/colorectal/skin/esophageal cancer	[117]
*Zanthoxylum usambarense* (Engl.) Kokwaro	Tree	Bark, leaves, fruits, seeds, roots, stems	Malaria, diarrhea, postpartum weakness, backache, joint pains, emetic, amoeba, arthritis, bone problems, body weakness, brucellosis, cough, colds, flu, headache, rheumatism, STIs, stomach disorders, tonsilitis, typhoid, ulcers, throat infections, respiratory tract infections, malignant catarrhal fever, fever, chest pain, sore throat, livestock medicine, chest pain, pneumonia, toothache, bleeding gums, bad breath, purgative	[41,67,68,77,93,103,104,105,107,113,135,137,138,140,142,168]
**Salicaceae**				
*Casearia battiscombei* R.E.Fr.	Tree	Bark, roots	Joint problems, abdominal pains, infertility, stomachache	[88,120]
*Dovyalis abyssinica* (A.Rich.) Warb.	Shrub/tree	Roots, stems, leaves, fruit, bark	Splenomegaly, arthritis, infertility, indigestion, postpartum weakness, diarrhea, malaria, afterbirth weakness, joint pain, STIs, backache, anthelmintic, stomachache, general body weakness, pneumonia, dirty circulatory system, stomach ulcers, boost immunity, seizures/epilepsy, muscle pains, invigorant, blood cleanser, skin rashes, venereal diseases, bone problems, cough, colds, flu, appetizer, rheumatism, gonorrhea, toothache, chest pains, colic pain in infants, headache, eyes problems, wounds, body pains, boils, heartburn, acidity, stomach ailments, chiggers, aphrodisiac, abdominal pains, typhoid, kidney problems, diabetes	[14,41,84,87,88,89,105,113,131,132,135,138,141,142,146,147,152,168]
*Dovyalis caffra* (Hook.f. & Harv.) Warb. (exotic)	Shrub	Fruits	Lack of appetite, malaria, rheumatism, cough (cow, goat, sheep, poultry, pig, ruminant)	[41,73]
*Dovyalis macrocalyx* (Oliv.) Warb.	Shrub/tree	Roots, leaves, whole plant, fruits	Constipation, peptic ulcers, headache, colds, joint pain, fever, malaria, breast problem, stomach complications, body weakness, seizures/epilepsy, skin rashes, boils, acaricide	[88,109,125,133,144,146]
*Flacourtia indica* (Burm.f.) Merr.	Shrub/tree	Roots, bark, leaves	Measles, chickenpox, dysmenorrhea, STIs, back/joint/bone problems, malaria, pneumonia, anthelmintic, arthritis, hypertension, stomachache, indigestion, asthma, indigestion, diarrhea, gonorrhea, infertility, anti-venom, abdominal pains	[41,74,86,87,99,111,120,132,141,154]
*Ludia mauritiana* J.F.Gmel.	Shrub/tree	Roots	Malaria	[14]
*Oncoba routledgei* Sprague	Shrub/tree	Fruits, flowers	Jiggers, biopesticide	[74]
*Oncoba spinosa* Forssk.	Shrub/tree	Leaves	Skin diseases	[144]
*Scolopia theifolia* Gilg	Shrub/tree	Roots	STIs	[142]
*Scolopia zeyheri* (Nees) Szyszyl.	Shrub/tree	Bark	Gonorrhea	[152]
*Trimeria grandifolia* (Hochst.) Warb.	Shrub/tree	Stem, bark, roots, fruits, leaves	Malaria, brucellosis, postpartum weakness, cough, backache, weak bones, colds, heartburn, STIs, arthritis, gonorrhea, heartburn, toothache, renal disorders, cancer, arthritis, rheumatism, fever, chest pain, joint pains, dysmenorrhea	[67,77,86,104,105,107,113,120,124,135,136,138,140,141,142,145,147,152,154,168]
**Salvadoraceae**				
*Azima tetracantha* Lam.	Shrub	Roots	Stomach disorders	[142]
*Dobera glabra* (Forssk.) Juss. Ex Poir.	Shrub/tree	Roots, branches	Eye problem, amoebiasis, anemia, rheumatism, gouts	[100,132,150]
*Salvadora persica* L.	Shrub/tree	Stems, bark, roots, twigs, leaves	Malaria, flu/cold, colds, stomachache, internal wounds, induce bile release from the gall bladder, eye infections, anthelmintic, constipation, tonic, respiratory infections, pneumonia, abdominal pain, colic pain, joint pains, abscesses, typhoid, blocked nose, fever, abortion, childbirth, retained placenta (cattle, human), ulcers, seizures, mange, trypanosomiasis, brucellosis, anthrax, cleaning the stomach after birth, emetic, purgative, dental caries, toothache, gum diseases, livestock diseases, backache, chest problems, chest pains, ulcers, aphrodisiac, labor pain, obesity, hernia, whitening teeth, rib pain, swollen legs and body pains during pregnancy, eye infections	[14,41,64,68,72,77,80,88,90,91,92,94,96,100,105,107,110,121,130,136,138,139,149,150]
**Santalaceae**				
*Osyridicarpos schimperianus* (Hochst. ex A.Rich.) A.DC.	Shrub		Malaria, swollen breasts	[136]
*Osyris lanceolata* Hochst. & Steud.	Shrub/tree	Stems, bark, leaves, roots, seeds	Ringworms, impotence, fatigue, boost immunity, back/joint/bone problems, cough/colds/flu, AIDS, allergies, back/joint/bone problems, blood purifier, chest problems, general body pains, fever, malaria, pneumonia, prostate cancer, rheumatism, stomach disorders, anthelmintic, abdominal pains in children, diarrhea, gonorrhea, pregnancy problems, swollen breasts, indigestion, excessive bleeding after birth, mosquito repellant, dysentery, typhoid, fever, tonic, swollen udder in cows, purgative, insecticide, cow fever, typhoid in livestock	[14,41,72,77,84,89,99,100,105,107,129,135,136,140,141,142,145,147,168]
*Viscum bagshawei* Rendle	Shrub	Leaves, stems	Enhance sexual performance	[143]
*Viscum fischeri* Engl.	Shrub	Whole plant, branches, twigs	Pneumonia, emetic, purgative, retained afterbirth, chest pains, hypertension, diarrhea, respiratory problems	[67,77,99,168]
*Viscum tuberculatum* A.Rich.	Shrub	Branches, bark	Pneumonia, retained afterbirth, hypertension, diarrhea, respiratory problems	[67,68,99,168]
**Sapindaceae**				
*Allophylus abyssinicus* (Hochst.) Radlk.	Tree	Leaves, roots, bark, fruits	Boils, hunchback, rickets, colds, skin problems, fatigue, boost immunity, headache, heartache, chest pains, childbirth problems, STIs, crooked limps, irregular menstruation, fever, weight loss, edema, toothache	[14,66,88,116,120,144,146,152,168]
*Allophylus pervillei* Blume	Shrub/tree	Roots, stem bark, leaves	Headache, prenatal care, colds, wounds, fibroids, female reproductive disorders	[75,127,150]
*Allophylus rubifolius* (Hochst. ex A.Rich.) Engl.	Shrub/tree	Roots, leaves	Headache, prenatal care	[127]
*Aporrhiza paniculata* Radlk.	Tree	Bark	Tuberculosis	[142]
*Blighia unijugata* Baker	Tree	Stems, roots, bark	Veterinary uses, headache, fever	[112,116,144]
*Cardiospermum corindum* L.	Herb	Bark, roots	Anthelmintic, amoeba, joint pains, malaria, polio, snakebites, stomachache, STI’s	[14,72,77,84,146]
*Cardiospermum halicacabum* L.	Herb	Seeds, roots, leaves	Snakebite, malaria, polio, antidote for arrow poison, eye medicine, rashes, boils, sprained muscles	[136,143,168]
*Deinbollia borbonica* Scheff.	Shrub/tree	Roots	Septic swelling, stomachache, aphrodisiac, diabetes, gastrointestinal complaints	[127,150]
*Deinbollia kilimandscharica* Taub.	Shrub/tree	Fruits	Poisonous	[168]
*Dodonaea viscosa* Jacq.	Shrub/tree	Bark, twigs, leaves, roots	Excessive menstruation, respiratory disorders, back/bone joints problems, diarrhea, stomach disorders, malaria, ECF, chest pain, pneumonia, stimulates milk after birth, respiratory disorders, rheumatic, dysmenorrhea, colds, tonsills in cattle	[14,41,73,77,88,99,104,113,115,132,141,168]
*Haplocoelum foliolosum* (Hiern) Bullock	Shrub/tree	Leaves	Eye lotion	[77]
*Lepisanthes senegalensis* (Poir.) Leenh.	Tree	Fruits, leaves	Poisonous	[142]
*Pappea capensis* Eckl. & Zeyh.	Shrub/tree	Stem, bark, leaves, roots, seeds	Backache, tonic, appetizer, indigestion, infertility, gout, stomachache, stamina, aphrodisiac, typhoid fever, amoebic dysentery, abdominal pain, excessive menstruation, body/muscle building, tuberculosis, rib/joint pains, AIDs, arthritis, bone problems, chest problems, cough, colds, flu, diarrhea, malaria, diabetes, body aches, invigorator, rheumatism, body weakness, postpartum hemorrhage, emetic, cancer, rheumatoid arthritis	[41,67,77,81,84,89,90,91,92,97,102,105,107,113,129,130,135,138,142,147,148,149,154,168]
*Paullinia pinnata* L.	Liana	Leaves, roots	Hiccoughs, acaricide, poisonous, diarrhea, snakebite	[125,133,142,144]
*Zanha africana* (Radlk.) Exell	Tree	Leaves, roots, bark	Miscarriage (bleeding), typhoid fever, abscesses, malaria, pregnancy edema, dog poison, pneumonia, toothache, flu, kidney ailments, allergies	[41,81,84,94,102,129,151]
**Sapotaceae**				
*Aningeria adolfi-friederici* (Engl.) Robyns & Gilbert	Tree	Latex, bark	Backache, stomachache, poisonous	[77,99,120,168]
*Englerophytum oblanceolatum* (S.Moore) T.D.Penn.	Shrub/tree	Fruits	Ulcers in digestive track, boils around belly, scabies	[116,125]
*Manilkara discolor* (Sond.) J.H.Hemsl.	Tree	Bark	Stomach disorders	[77]
*Manilkara mochisia* (Baker) Dubard	Tree	Roots, bark	Cough, colds, snakebites, fever	[100,127,142]
*Manilkara sansibarensis* (Engl.) Dubard	Tree	Bark	Pneumonia, rheumatism, cough	[112,142,150]
*Manilkara sulcata* (Engl.) Dubard	Shrub/tree	Roots	Cough, chest pains, snakebites, stings,diarrhea, ulcers/stomach upsets, foot and mouth disease in cattle	[100,127,134]
*Sideroxylon inerme* L.	Shrub/tree	Roots, bark	Chest pains, cough, disorders in menstruation	[127]
*Synsepalum cerasiferum* (Welw.) T.D.Penn.	Tree	Latex, bark	Colorectal/esophageal cancer, wounds	[117,142]
*Vitellariopsis kirkii* (Baker) Dubard	Shrub/tree	Roots	Stomach ailments	[127]
**Scrophulariaceae**				
*Buddleja polystachya* Fresen.	Shrub	Roots, leaves	Infertility, eye diseases	[99,120,141]
**Simaroubaceae**				
*Brucea antidysenterica* J.F.Mill.	Shrub/tree		Arrow poison, malaria	[136]
**Smilacaceae**				
*Smilax anceps* Willd.	Liana	Shoots	STIs	[144]
**Solanaceae**				
*Capsicum annuum* L. (exotic)	Herb/shrub	Fruits, leaves, stems	Eye problems, cellulitis, diarrhea/dysentery (cow, poultry, goat, sheep, pig), dullness (poultry), Newcastle disease, coccidiosis (poultry), weakness/dullness (cow, goat, sheep, poultry, pig), throat/breast/squamous cell carcinoma, joint problems, acaricide, tumors, constipation, abdominal pains	[73,94,95,117,119,133,139,143,144,150,168]
*Datura metel* L. (exotic)	Herb	Stems, leaves, flowers	Poison, respiratory congestion	[134]
*Datura stramonium* L. (exotic)	Herb	Seeds, leaves, roots, fruits	Colds, mouth inflammation, toothache, asthma, ectoparasites i.e., mites, lice, flea infestation (cow, poultry), tick infestation (cow, poultry, goat, sheep, pig), coccidiosis and Newcastle (poultry), foot rot (cow, goat, sheep, ruminant, pig), body swellings, tonsils, earache, wounds, boils, asthma, fever, poisonous, madness, epilepsy	[73,88,115,138,144,152,168]
*Lycium europaeum* L.	Shrub	Root, leaves, stem	Malaria, rheumatism, breast swelling, headache, backache, respiratory diseases, rib pain, backpain, joint pain, cough	[72,77,137,142]
*Nicotiana tabacum* L. (exotic)	Herb	Leaves, fruits	Eye problems, malnutrition, insecticide, wounds, colds, constipation, pesticide of stored grains, stomach infections, babesiosis, gastroenteritis, chronic cough, gingivitis, candidiasis, glossitis, ectoparasites i.e., mites, lice and fleas’ infestation (cow, poultry), acaricide, expectorant, leech	[68,73,88,96,109,120,133,137,141,146,147,168]
*Physalis lagascae* Roem. & Schult.	Herb	Leaves, whole plant	Stomachache, boils, malaria, facilitate childbirth	[76,119,144]
*Physalis peruviana* L.	Herb	Roots, leaves, fruits	Malaria, postpartum pain, diarrhea, cholera, joint pains, stiff joints, skin allergies, respiratory disorders, tonsillitis, arthritis, anthelmintic, cough, colds, anemia, acidity, chest problems, typhoid, acaricide, mouth ulcers, ear discharge, stomachache, indigestion, constipation, diuretic, asthma, abdominal ailments, headache, hiccups, bloat in cattle/humans	[14,88,89,101,110,113,115,119,122,133,143,146,147,152,154,168]
*Solanum aculeastrum* Dunal	Shrub/tree	Leaves, roots, fruits	Pneumonia, liver diseases, boils at teething, chest problems, malaria, joint pains, infertility in women, diarrhea, gonorrhea, livestock diseases, eye problems, fever, spinal cord problem, colds, toothache, nausea fever, osteomyelitis, backache, rheumatism, pesticide of stored grains, warts, bleeding gums, respiratory symptoms and contagious bovine pleuropneumonia (livestock), skin/breast/cervical cancer, tonsils, wounds, bronchitis, venom, expectorant, dysmenorrhea, immune booster	[14,67,82,87,99,109,113,115,117,131,132,141,142,146,147,152,168]
*Solanum aculeatissimum* Jacq.	Herb/shrub	Roots, leaves	Asthma, cough, colds, eye problem, fever, spinal cord problem, throat problems, wounds	[87,109,136]
*Solanum americanum* Mill.	Herb	Leaves, fruits, roots	STIs, general body problems, chest pains, joint pains, backache, stomachache, diarrhea, gonorrhea, headache, mouth inflammation, toothache, sprains, colic pain in infants, vitamin/mineral deficiency in pregnancy, amoeba, tonsils, anthelmintic, stomach crumps, acidity, indigestion, diabetes, earache, toothache, constipation, typhoid fever, teeth problems, stomachache, fever, throat, wounds, erectile dysfunction (impotence), malaria prevention, respiratory disorders, burns, scalds	[41,76,79,83,84,88,106,115,132,136,143,144,145,147,152,168]
*Solanum anguivi* Lam.	Shrub	Fruits, roots	Skin problems, ringworms, infertility in women, stomachache, poultry (skin rashes, loss of feather)	[73,152]
*Solanum arundo* Mattei	Shrub/tree	Roots	Fever, sore throat, wounds	[77,89]
*Solanum goetzei* Dammer	Herb/shrub	Leaves	Abscesses	[142]
*Solanum hastifolium* Hochst. ex Dunal	Shrub	Roots, leaves	Eye lotion, livestock diseases (anthrax, black quarter), diarrhea, smallpox	[77,121,142]
*Solanum incanum* L.	Herb/shrub	Roots, bark, fruits, leaves	Ear infection/ache, constipation, throat infection, stomachache, malaria, oral hygiene, tonic, sore throat, oral wounds/thrush, diarrhea, respiratory disorders, toothache, ringworms, joint pains/swelling, anthelmintic, amoeba, asthma, back/bone joints problems, brucellosis, chest problems, cough, colds/flu, dental problems, family planning, gastritis, poison/sting/snake/spider bite antidote, pesticide, pimples, prostate cancer, rheumatism, ringworms, skin diseases, TB, fever, chest pain, wounds, skin disorders, respiratory disorders in sheep, arthritis, headache, sprains, rabies, whitlow, emetic, diabetes mellitus, polio, jiggers, warts, fever, ingestion, ectoparasites/acaricide, delayed labor, prevent abortion, vomiting, helminthiasis (cow, poultry, goat, sheep, pig), lumpy skin disease (cow), foot rot (cow, goat, sheep, ruminants, pig), infection of umbilical cord, erectile dysfunction, abdominal/colic pains, backache, liver enlargement, scabies, expectorant, boils, kidney problem, hasten child delivery, typhoid, edema, leprosy, chickenpox, pneumonia, HIV, swollen legs in pregnancy, retained placenta, measles, sheep (colds)	[14,41,51,66,67,68,72,73,76,77,79,81,82,84,85,88,89,90,91,92,96,99,100,101,103,105,106,111,115,119,120,125,128,129,130,132,133,135,136,138,139,141,142,143,144,146,147,148,150,152,154,168]
*Solanum mauense* Bitter	Shrub	Roots, fruits	Anthelmintic, purgative, chest ailments, pneumonia, anthrax (man/animals), malaria, chest pains, teeth problems, flu, stomachache, fever, throat, wounds	[67,89,131,132,136,142,168]
*Solanum mauritianum* Scop. (exotic)	Shrub/tree	Fruits, bark, roots	Pneumonia, cough, asthma, malaria, splenomegaly, colorectal cancer, skin rashes, STIs, poisonous	[87,115,117,144]
*Solanum nakurense* C.H.Wright	Herb/shrub	Fruits	Pneumonia, otitis media	[131,152]
*Solanum nigriviolaceum* Bitter	Shrub	Fruits, roots	Teeth problems, ringworms, rashes, colds, skin infections, joint pain, wounds, chest pain, fever, ear problem, cough, livestock diseases	[67,87,99,109,143]
*Solanum runsoriense* C.H. Wright	Liana		Fever, throat, wounds	[136]
*Solanum schumannianum* Dammer	Shrub		Fever, throat, wounds	[136]
*Solanum taitense* Vatke	Shrub	Roots, leaves, stems	Arthritis, malaria, typhoid, stomachache, respiratory	[92,105]
*Solanum terminale* Forssk.	Shrub/liana	Fruits, roots	Arthritis, toothache, chest congestion, rashes, fever, throat, wounds	[86,132,136,154]
*Solanum tettense* Klotzsch	Shrub/tree	Roots, leaves	Boils, abscesses, diabetes mellitus, fever, typhoid, throat, wounds, threatened miscarriage, stomach pains, retained placenta, abdominal pains, diarrhea	[51,72,77,85,94,123,136,139,141,142]
*Solanum tuberosum* L. (exotic)	Herb		Bites, wounds	[41]
*Solanum zanzibarense* Vatke	Shrub	Roots	Toothache	[142]
*Withania somnifera* (L.) Dunal	Herb/shrub	Roots, bark, whole plant, fruits, stems, leaves	Tonic, backache, joint pains, galactagogue, appetite, livestock diseases, gonorrhea, excessive menstrual flow, postpartum pain, meningitis, cerebral malaria, anthrax, detoxify meat from infected animal, urinary tract, rectal problems, STIs, diarrhea, toothache, joint pains, bone problems, cough/colds/flu, malaria, arthritis, pneumonia, painkiller, stomach upsets, burns, wounds, headache, rheumatism, boils, allergic conditions, retained afterbirth, eye diseases, coagulant, acaricide, amoeba, cancer, purgative, anodyne, anthrax, skin sores/ulcers, gum diseases, abdominal pains, aphrodisiac, gynecological problems, jaundice, hepatitis, stomachache, diabetes, blood pressure	[14,41,67,72,74,77,81,84,85,88,90,91,93,103,104,105,113,115,120,130,132,133,141,142,143,146,147,154,168]
**Stilbaceae**				
*Nuxia congesta* R.Br. ex Fresen.	Shrub/tree	Leaves, roots, bark	Backache, stomachache, joint pains, body weakness, respiratory disorders, indigestion, menstrual pains, astringent, abdominal/colic pains, flu, malaria, typhoid, smallpox, pneumonia, rashes, varicose veins, vomiting, mumps, tonsils, edema, dysmenorrhea, expectorant, colds, cough	[14,88,91,99,109,132,141,145,152]
**Talinaceae**				
*Talinum caffrum* (Thunb.) Eckl. & Zeyh.	Herb	Roots	Prevent abortion, fever	[139]
*Talinum portulacifolium* (Forssk.) Asch. ex Schweinf.	Herb	Leaves, roots	Arthritis, backache, STIs e.g., gonorrhea, wounds, boils	[77,92]
**Theaceae**				
*Camellia sinensis* (L.) Kuntze (exotic)	Shrub/tree	Leaves	Eye problems, stomach disorders, STIs	[14,41,95]
**Thymelaeaceae**				
*Englerodaphne subcordata* (Meisn.) Engl.	Shrub	Roots	Constipation in children, allergy after eating meat, poisonous	[140]
*Gnidia involucrata* Steud. ex A.Rich.	Herb/shrub	Stalks	Expectorant	[141]
*Lasiosiphon glaucus* Fresen.	Shrub/tree	Roots, bark, twigs, leaves	Fibers are said to be poisonous, arrow poison, abdominal pains, back pains, joint ache, insecticide, irregular menstruation	[84,136,141,142,168]
*Lasiosiphon latifolius* (Oliv.) Brenan	Shrub/tree	Roots, leaves, bark	Poisonous, ulcers, evacuation of bowels, toxic to cows, pesticide, snakebites, pesticide for maize storage	[96,99,100,142,145]
*Struthiola thomsonii* Oliv.	Herb/shrub	Roots	Cough, stomachache	[67,99,142]
*Synaptolepis kirkii* Oliv.	Shrub	Fruits, leaves, roots	Anthelmintic, convulsions, wounds, snakebite, female infertility, hydrocele	[127,142,150]
**Typhaceae**				
*Typha domingensis* Pers.	Herb	Roots	Diabetes, diarrhea, impotency, female conditions	[83,168]
*Typha latifolia* L.	Herb	Roots	Diarrhea, impotency, female conditions	[168]
**Urticaceae**				
*Girardinia diversifolia* (Link) Friis	Herb	Roots	Tonic	[77]
*Laportea alatipes* Hook.f.	Herb	Leaves	Vitamin supplement	[14]
*Laportea ovalifolia* (Schumach. & Thonn.) Chew	Herb	Roots, leaves	Excessive menstrual bleeding, retained placenta, diarrhea, stomach problems, gastrointestinal infections, blood booster	[99,144]
*Myrianthus holstii* Engl.	Tree	Leaves, bark, roots	Stomachache	[14]
*Obetia radula* (Baker) Baker ex B.D.Jacks.	Tree	Roots	Barrenness	[142]
*Pouzolzia parasitica* (Forssk.) Schweinf.	Herb	Stems, roots	Burn, livestock diseases	[77]
*Urena lobata* L.	Shrub	Roots	Afterbirth, ease delivery	[120]
*Urera hypselodendron* (Hochst. ex A.Rich.) Wedd.	Liana	Roots, leaves	Stomachache after birth, spider bites, antidote, livestock diseases, pregnancy problems, tonic	[67,142,152]
*Urera sansibarica* Engl.	Liana	Leaves	Leprosy	[127]
*Urtica dioica* L. (exotic)	Herb	Leaves, roots	Diabetes, ulcers, boils, anthelmintic, skin, stomach infections	[105,120,123]
*Urtica massaica* Mildbr.	Herb	Roots, leaves, whole plant	Abdominal pains, stomachache, aphrodisiac, irregular menstruation, poison antidote, infertility, malaria, sprains, headache, typhoid, dirty circulatory system, joint pains, chest pains, mouth inflammation, muscle pains, body weakness, livestock diseases, kidney problem, bruises, arthritis, bone problems, cough, colds, flu, diabetes, tonic, injuries/cuts, wounds, heartburn, hypertension, leg swellings, appetizer, pneumonia, rheumatism, stomach acid, arthritis, adds blood in the body, cancer, diabetes, anemia, skin/uterine/breast cancer, pimples, urinary problems, ulcers, vomiting, leukemia, skin roughness, problems of passing urine, fractures, venereal diseases	[41,83,85,87,90,99,104,107,109,113,115,117,128,132,146,147,152,154,168]
**Velloziaceae**				
*Xerophyta spekei* Baker	Shrub	Whole plant, stems	Burns, dog bites, diabetes, snakebites	[84,94]
**Verbenaceae**				
*Lantana camara* L. (exotic)	Shrub	Leaves, roots, whole plant	Diarrhea, measles, joint pains, amoeba, back/joint/bone problems, cough, colds/flu, dental problems, headache, injuries/cuts/wounds, malaria, wounds, change the sex of expected kids, toxicity, pneumonia (cow, sheep), cough (cow, goat, sheep, poultry, pig, ruminant), sore throat, nose bleeding, breathing problems, acaricide, poison, anthelmintic, hepatitis, rheumatism, toothache, anticoagulant, liver problems, retained placenta, conjunctivitis/eye problems, toothache, runny nose	[14,41,73,76,80,84,86,104,106,108,111,115,119,127,130,133,142,143,145,146,147,150]
*Lantana trifolia* L.	Shrub	Roots, leaves, aerial parts. twigs	Stomachache, malaria, diarrhea in livestock, back/joint/bone problems, cough, colds/flu, fever, pneumonia, hypertension, heartburn, oral thrush, hepatic diseases, glandular disorders, rheumatism, sore throat, livestock joint problems, constipation, hiccups, chest problems, eye injury, anemia, ringworms, boil, mouth ulcers, abdominal/colic pains, enhance milk letdown, abdominal enlargement, general body weakness, astringent, anodyne, expectorant, meningitis, anthelmintic, hepatitis, toothache, anticoagulant, liver problems, sore throat, conjunctivitis, eye problems, kidney problems, urinary tract problems	[41,74,82,86,91,92,99,101,109,115,119,120,122,125,138,140,141,143,145,146,154]
*Lantana ukambensis* (Vatke) Verdc.	Herb	Roots, leaves, whole plant stems	Measles, malaria, smallpox, strength, colds, headache, cough, asthma, medicine for pregnant woman, children skin rashes, runny nose	[14,72,77,147]
*Lantana viburnoides* (Forssk.) Vahl	Shrub	Leaves, whole plant	Aphrodisiac, medicine for a pregnant woman	[77,80]
*Lippia abyssinica* (Otto & A.Dietr.) Cufod.	Shrub	Leaves, roots, stems	Acaricide, conjunctivitis, painful eyes	[122,133]
*Lippia javanica* (Burm.f.) Spreng.	Shrub	Leaves, roots, twigs, stems	Wounds, headache, chest congestion, malaria, migraines, measles, postpartum hemorrhage, children skin rashes, colds/flu, anaplasmosis, amoeba, body weakness, cough, anthrax, abdominal pains, tapeworms, chest complaints, toothache, stomachache, bloody diarrhea, afterbirth pains, insect repellant	[68,77,81,86,91,102,104,115,131,140,141,143,144,168]
*Lippia kituiensis* Vatke	Herb/shrub	Leaves, roots	Respiratory problems, measles, livestock diseases, chronic joint pains, cough, colds, flu, malaria, stomachache, emetic	[14,41,90,105]
*Priva curtisiae* Kobuski	Herb	Leaves	Ophthalmia	[67]
*Verbena bonariensis* L.	Herb	Leaves	Mouth infection in babies	[109]
**Viburnaceae**				
*Sambucus africana* Standl.	Herb	Flowers	Colds	[168]
**Violaceae**				
*Rinorea ilicifolia* (Welw. ex Oliv.) Kuntze	Shrub/tree	Roots	Snakebite antidote	[134]
**Vitaceae**				
*Ampelocissus africana* (Lour.) Merr.	Shrub/liana	Roots	Threatened miscarriage	[141]
*Cayratia ibuensis* (Hook.f.) Suess.	Herb	Leaves	Leprosy	[144]
*Cissus aphyllantha* Gilg	Shrub/liana	Roots	Diarrhea, wounds, burns, boils, swollen legs, body pains and pregnancy, hemorrhage, retained placenta, headache, pneumonia	[51,77,96,139]
*Cissus phymatocarpa* Masinde & L.E.Newton	Herb	Stems	Septic swelling, eardrops	[127]
*Cissus quadrangularis* L.	Herb	Leaves, stems, sap, roots	Cuts, wounds, diarrhea, septic swelling, ear drops, respiratory disorders in cattle, colds, malaria, stomach problems, gastric ulcers, schistosomiasis, neurosis, ECF, rheumatism, epilepsy, TB, asthma, colibacillosis, rib pain, livestock hepatitis, skin disorders, earache, chest pain, pesticide, poisonous, fly infestation, viral, joint diseases, eye infections, skin problems, diarrhea/fever/virus in poultry	[51,67,68,72,76,77,89,100,105,121,127,142,143,149,168]
*Cissus rotundifolia* Vahl	Liana	Leaves, roots, stems, whole plant	Stomachache, septic swelling, ear drops, allergies, swollen joints, spleen problems, bee stings, throat infection, pneumonia, cough, tonic, boils, skin diseases, body pains, cuts, skin inflammation, retained placenta, abdominal pains, septic wounds, poultry diseases (diarrhea, fever, sneezing)	[51,67,71,77,84,88,98,100,127,139,142,143]
*Cissus sylvicola* Masinde & L.E.Newton	Liana	Stems	Septic swelling, ear drops	[127]
*Cyphostemma adenocaule* (Steud. ex A.Rich.) Desc. ex Wild & R.B.Drumm.	Liana	Roots, leaves, bark, whole plant	Stomachache, swollen abdomen, tuberculosis, arthritis, malaria, jiggers, colorectal/breast/skin cancer, syphilis, anthelmintic, pneumonia, acaricide, weight loss	[14,72,76,99,108,117,133,144]
*Cyphostemma bambuseti* (Gilg & M.Brandt) Desc. ex Wild & R.B.Drumm.	Liana		Back/joint problems, cough, colds/flu, HIV/AIDs, malaria, rheumatism, sore throat, STIs, stomach disorders, bone pain, tuberculosis, aphrodisiac	[41,136]
*Cyphostemma cyphopetalum* (Fresen.) Desc. ex Wild & R.B.Drumm.	Herb	Leaves, roots, whole plant	STIs, tonic, galactagogue, stomachache, malaria, dermatitis, eye problems, enlarged glands, pesticide, teeth problems, gonorrhea, retained placenta, itchy skin rash, chickenpox, swollen legs and body pains during pregnancy	[90,91,94,96,99,132,139,141,152]
*Cyphostemma heterotrichum* (Gilg & R.E.Fr.) Desc. ex Wild & R.B.Drumm.	Shrub	Whole plant	Acaricide	[133]
*Cyphostemma kilimandscharicum* (Gilg) Desc. ex Wild & R.B.Drumm.	Herb		Bone pain, tuberculosis	[136]
*Cyphostemma maranguense* (Gilg) Desc.	Herb	Stems	Anaplasmosis, malaria	[115,168]
*Cyphostemma serpens* (Hochst. ex A.Rich.) Desc.	Herb	Leaves, stems, roots, whole plant	Tonic, cervical/skin/breast cancer, acaricide, abortifacient, abscess, boils, gynecological diseases, polio, dermatological problems	[67,91,117,133,143]
*Cyphostemma ukerewense* (Gilg) Desc.	Herb	Leaves	Tonsils	[119]
*Rhoicissus revoilii* Planch.	Liana	Roots, stems, whole plant	Stomachache, cuts, sores, burns, livestock diseases, stomach disorders, strength, acaricide, antiseptic, gastrointestinal problems, general body malaise, malaria, facilitate conception/pregnancy, venereal diseases, leprosy	[67,77,79,98,100,127,133,142,143]
*Rhoicissus tridentata* (L.f.) Wild & R.B.Drumm.	Shrub/liana	Roots, stems, bark, exudate	Wounds, malaria, cuts, sores, snakebites, constipation, stomachache, diarrhea, bilharzia, ear drops, oral cavity infections, arthritis, livestock diseases, heartburn, peptic ulcers, renal disorders, anemia, cleans blood, respiratory infections, fever, menstrual pains, fever, acaricide, anthelmintic, prevent miscarriage, prophylaxis for children/babies diseases, abdominal pains, skin diseases, hydrocele, tapeworms, red/painful eyes, infertility, kidney and bladder complaints, dysmenorrhea, livestock fever	[67,68,86,87,99,101,105,107,123,127,133,140,141,142,144,147,150,154,168]
**Asphodelaceae**				
*Aloe ballyi* Reynolds	Tree	Leaves	Pneumonia, malaria	[84]
*Aloe dawei* A.Berger	Shrub	Leaves, roots	Cathartic, gastrointestinal problems, hoof wounds, mouth diseases, anthraxdermatitis, kidney ailment	[143,144]
*Aloe deserti* A.Berger	Shrub	Leaves	Malaria	[111]
*Aloe elgonica* Bullock	Shrub	Leaves, roots	Malaria, ulcers, urinary tract, acaricide	[120,133]
*Aloe kedongensis* Reynolds	Shrub	Roots, leaves	Abdominal pains in infants, rashes/ringworms, colds, infertility in women, malaria, headache, appetizer, livestock diseases, asthma, pneumonia, gonorrhea, laxative, fever, wounds, anaplasmosis in cattle, chicken diseases, typhoid, skin diseases, ear problems, coccidiosis	[78,84,86,87,98,135,168]
*Aloe kilifiensis* Christian	Herb	Latex	Headache	[127]
*Aloe lateritia* Engl.	Herb	Leaves, juice	Typhoid, laxative, wounds, constipation, malarial skin conditions	[84,168]
*Aloe macrosiphon* Baker	Shrub	Leaves	Malaria	[111]
*Aloe secundiflora* Engl.	Shrub	Inflorescence, roots, leaves, exudate, whole plant	Tonic, ECF, headache, diarrhea, typhoid, edema, HIV/AIDs, STIs, blood cleanser, infertility, respiratory disorders, allergies, obesity, joint pains, abscesses, cellulitis, cholera, ulcers, filariasis, fatigue, scorpion stings, arthritis, rheumatism, eyes, tuberculosis, aching ears, diabetes, malaria, chest pains, conjunctivitis, wounds, ectoparasites, retained afterbirth, prevent abortion, vomiting, pneumonia, chest pains, amoeba, rashes, anthelmintic, stomachache, acne, laxative, constipation, colds, cuts, cough, body weakness, abdominal pains, purgative, indigestion, skin conditions, antiseptic, peptic ulcers, diaphragm swelling, diarrhea in poultry	[14,51,64,68,72,74,80,81,88,90,94,96,99,102,103,104,105,106,111,113,129,130,132,139,146,147,151,152,153,168]
*Aloe vera* (L.) Burm.f. (exotic)	Herb	Leaves, exudate	Malaria, wounds, swelling, boils, laxative, constipation, malarian skin conditions	[74,76,111,168]
*Aloe volkensii* Engl.	Shrub	Leaves, roots, bark	Headache, anthelmintic (livestock), wounds, malaria, colorectal/esophageal/prostate cancer, whooping cough, pneumonia, chest pain, cough, joint pains/swellings, fever, stomachache, diarrhea, skin diseases, hypertension, eye problems, veterinary medicine, sore feet, jiggers	[71,92,99,109,117,127,144,150]
**Zamiaceae**				
*Encephalartos hildebrandtii* A.Braun & C.D.Bouché (exotic)	Tree	Stems	Septic swelling	[127]
**Zingiberaceae**				
*Aframomum angustifolium* (Sonn.) K.Schum.	Herb	Seeds	Anthelmintic	[147]
*Aframomum mala* (K.Schum.) K.Schum.	Herb	Seeds	Anthelmintic	[143]
*Aframomum melegueta* K.Schum. (exotic)	Herb	Seeds	Amoeba	[146]
*Aframomum zambesiacum* (Baker) K.Schum.	Herb	Leaves	Colds	[14]
*Zingiber officinale* Roscoe (exotic)	Herb	Roots	Malaria, sore throat, respiratory diseases, throat infections, internal body pains, flu, colds, fever, indigestion, circulatory stimulant	[108,137,143,168]
**Zygophyllaceae**				
*Balanites aegyptiaca* (L.) Delile	Tree	Roots, bark, fruits, resin	Stomachache, respiratory disorders, itch, bone problem, bilharzia, constipation, cough, colds/flu, headache, kwashiorkor, burns, eyes, malaria, purgative, diarrhea, *Chira*, body/chest/rib pain, rheumatism, blood cleanser, respiratory disorders, edema, heartburn, bilharzia, epigastric pain, expectorant, dysmenorrhea, skin irritation, measles, aphrodisiac, retained placenta, abdominal pains	[41,51,67,71,72,76,77,80,88,91,96,97,99,100,104,106,108,110,138,139,141,142,143,147,148]
*Balanites glabra* Mildbr. & Schltr.	Shrub/tree	Roots, branches, fruits, bark	Induce vomiting during pregnancy or if one has malaria, induce bile release, pneumonia, cowpox, stomach upsets, emetic to clean the stomach, chronic joint pains	[92,105,130,135]
*Balanites pedicellaris* Mildbr. & Schltr.		Roots	Emetic, skin irritations	[100,142]
*Balanites rotundifolia* (Tiegh.) Blatt.	Shrub/tree	Roots, leaves, fruits	Urinary tract infections, gastrointestinal complications (emetic), eye infection, eye lotion, wounds, pesticide	[68,77,91,123,142]
*Balanites wilsoniana* Dawe & Sprague	Tree	Bark, leaves, roots	General body pains, muscle injuries, insomnia, amnesia	[127,150]
*Tribulus terrestris* L.	Herb	Whole plant	Erectile dysfunction, arthritis	[91,132]

## Appendix B

Checklist of food plants in Kenya; introduced species are indicated in brackets as ‘exotic.’

**Family/Species**	**Habit**	**Part Eaten**	**References**
**Acanthaceae**			
*Asystasia gangetica* (L.) T.Anderson	Herb	Leaves (vegetable)	[134,158,159,160,161,166,167]
*Asystasia mysorensis* (Roth) T.Anderson	Herb	Leaves (vegetable)	[159,160,166,167,169]
*Crabbea velutina* S.Moore	Herb	General	[163]
*Crossandra mucronata* Lindau	Herb	Flowers	[13,163]
*Crossandra nilotica* Oliv.	Herb	General	[163]
*Dyschoriste radicans* Nees	Herb	Leaves (vegetable)	[159,160,169]
*Justicia anagalloides* (Nees) T.Anderson	Herb	General	[162]
*Justicia caerulea* Forssk.	Herb	General	[163]
*Justicia calyculata* Deflers	Herb	Leaves (vegetable)	[159,160,163]
*Justicia ladanoides* Lam.	Herb	Leaves (vegetable)	[16,163]
*Justicia matammensis* (Schweinf.) Oliv.	Herb	Leaves (vegetable)	[16,169]
*Justicia odora* (Forssk.) Lam.	Herb	General	[163]
**Achariaceae**			
*Rawsonia lucida* Harv. & Sond.	Tree	Fruits	[13,142]
*Sesuvium portulacastrum* (L.) L.	Shrub/subshrub	Leaves (vegetable)	[134]
**Alismataceae**			
*Burnatia enneandra* Micheli	Herb	Tubers	[156]
**Amaranthaceae**			
*Achyranthes aspera* L.	Herb	Leaves (vegetable)	[159,160,169]
*Amaranthus blitum* L.	Herb	Leaves (vegetable)	[12,134,159,160,166,167]
*Amaranthus caudatus* L.	Herb	Leaves (vegetable)	[134]
*Amaranthus graecizans* L.	Herb	Leaves (vegetable)	[12,13,18,157,159,160,162,163,165,166,167]
*Amaranthus hybridus* L.	Herb	Leaves (vegetable)	[12,16,18,134,159,160,162,166,167,169]
*Amaranthus sparganiocephalus* Thell.	Herb	Leaves (vegetable)	[166,167]
*Amaranthus spinosus* L.	Herb	Leaves (vegetable)	[134,159,160,166,167]
*Amaranthus thunbergii* Moq.	Herb	General	[163]
*Amaranthus tortuosus* Hornem. (exotic)	Herb	Leaves (vegetable)	[165,166]
*Caroxylon dendroides* (Pall.) Tzvelev (exotic)	Shrub/subshrub	Leaves	[13]
*Celosia argentea* L.	Herb	Stems, leaves (vegetable)	[18,134]
*Celosia schweinfurthiana* Schinz	Subshrub	Leaves (vegetable)	[16]
*Celosia trigyna* L.	Herb	Leaves (vegetable)	[169]
*Chenopodium opulifolium* Schrad. ex W.D.J.Koch & Ziz	Herb	Leaves (vegetable)	[13,16,159,160,169]
*Cyathula orthacantha* (Hochst. ex Asch.) Schinz	Herb	General	[163]
*Digera muricata* (L.) Mart.	Herb	Leaves, flowers (vegetable)	[18,163,166,167]
*Ouret lanata* (L.) Kuntze	Subshrub	Leaves (vegetable)	[166,167]
*Psilotrichum sericeum* (J.Koenig ex Roxb.) Dalzell	Herb	Leaves (vegetable)	[161]
*Pupalia lappacea* (L.) Juss.	Herb	General	[163]
*Sericocomopsis hildebrandtii* Schinz	Subshrub/shrub	General	[163]
**Amaryllidaceae**			
*Scadoxus multiflorus* (Martyn) Raf.	Herb	General	[163]
**Anacardiaceae**			
*Anacardium occidentale* L. (exotic)	Tree	Fruits	[158]
*Lannea alata* (Engl.) Engl.	Shrub/tree	Fruits	[100,142,167]
*Lannea edulis* (Sond.) Engl.	Shrub/tree	Fruits	[167]
*Lannea fulva* (Engl.) Engl.	Shrub/tree	Fruits	
*Lannea rivae* (Chiov.) Sacleux	Shrub/tree	Fruits, bark, tubers	[13,100,142,163,165,167]
*Lannea schimperi* (Hochst. ex A.Rich.) Engl.	Shrub/tree	Fruits	[167]
*Lannea schweinfurthii* (Engl.) Engl.	Shrub/tree	Fruits; bark (tea)	[14,100,142,163,167]
*Lannea triphylla* (Hochst. ex A.Rich.) Engl.	Shrub/tree	Fruits, roots; bark (tea)	[13,18,142,156,163,167]
*Ozoroa insignis* Delile	Shrub/tree	Gum	[13,163]
*Pistacia aethiopica* Kokwaro	Tree	Roots (tea)	[142]
*Sclerocarya birrea* (A.Rich.) Hochst.	Tree	Seeds, fruits	[100,134,142,161,163,165,167]
*Sclerocarya gillettii* Kokwaro	Tree	Fruits	[142]
*Searsia crenulata* (A.Rich.) Moffett	Shrub/tree	Fruits	[100]
*Searsia longipes* (Engl.) Moffett	Shrub/tree	Fruits	[100,167]
*Searsia natalensis* (Bernh. ex C.Krauss) F.A.Barkley	Shrub/tree	Fruits; leaves (chewed); leaves, bark (tea)	[14,16,142,162,163,166,167]
*Searsia pyroides* (Burch.) Moffett	Shrub/tree	Fruits; leaves (tea)	[14,16,142,167]
*Searsia quartiniana* (A.Rich.) A.J.Mill.	Shrub/tree	Fruits	[167]
*Searsia ruspolii* (Engl.) Moffett	Shrub/tree	Fruits	[167]
*Searsia tenuinervis* (Engl.) Moffett	Shrub/tree	Fruits; leaves (chewed, vegetable)	[142,166,167]
*Sorindeia madagascariensi*s (Spreng.) DC.	Tree	Fruits	[100,142]
**Annonaceae**			
*Annona senegalensis* Pers.	Shrub/tree	Fruits; bark (chewed)	[16,142,167]
*Annona squamosa* L. (exotic)	Shrub/tree	Fruits	[158]
*Monodora myristica* (Gaertn.) Dunal	Tree	Seeds	[142]
*Uvaria acuminata* Oliv.	Shrub/tree	Fruits	[100,142,167]
*Uvaria denhardtiana* Engl. & Diels	Shrub	Fruits	[142]
*Uvaria kirkii* Oliv. ex Hook.f.	Shrub	Fruits	[142]
*Uvaria lucida* Bojer ex Benth.	Shrub/tree	Fruits	[161]
*Uvaria scheffleri* Diels	Shrub/liana/tree	Fruits	[13,142,163,165,167]
*Uvariodendron anisatum* Verdc.	Shrub/tree	Fruits	[14]
**Apiaceae**			
*Afroligusticum linderi* (C.Norman) P.J.D.Winter	Herb	Roots	[13]
**Apocynaceae**			
*Acokanthera oppositifolia* (Lam.) Codd	Shrub/tree	Fruits	[142,155]
*Acokanthera schimperi* (A.DC.) Schweinf.	Shrub/tree	Fruits	[142,155,167]
*Ancylobothrys petersiana* (Klotzsch) Pierre	Shrub	Fruits	[100,142,155,161]
*Ancylobothrys tayloris* (Stapf) Pichon	Liana	Fruits	[142]
*Baseonema gregorii* Schltr. & Rendle	Herb	Tubers	[13]
*Calotropis procera* (Aiton) W.T.Aiton	Shrub/tree	Seeds	[18]
*Caralluma arachnoidea* (P.R.O.Bally) M.G.Gilbert	Herb	General	[164]
*Carissa bispinosa* (L.) Desf. ex Brenan	Shrub/tree	Fruits	[142]
*Carissa spinarum* L.	Shrub/tree	Fruits, flowers; roots (vegetable)	[13,14,16,18,100,142,155,156,163,165,167]
*Carissa tetramera* (Sacleux) Stapf	Shrub	Fruits, roots	[100,155,158,161]
*Ceropegia ballyana* Bullock	Herb	General	[164]
*Ceropegia johnstonii* (N.E.Br.) Bruyns	Herb	Tubers	[18,156,162]
*Ceropegia stenantha* K.Schum.	Herb	Tubers	[13]
*Ceropegia stenoloba* Hochst. ex Chiov.	Herb	Tubers	[13]
*Ceropegia woodii* Schltr.	Herb	Tubers	[156,163]
*Cynanchum hastifolium* K.Schum.	Herb	Fruits	[13,163]
*Cynanchum insipidum* (E.Mey.) Liede & Khanum	Herb	Fruits; leaves (vegetable)	[13,156,159,160,166]
*Cynanchum viminale* (L.) L.	Herb	Raw stems edible; roots (vegetable)	[156,163,167]
*Desmidorchis retrospiciens* Ehrenb.	Herb	General	[163,164]
*Dictyophleba lucida* (K.Schum.) Pierre	Liana	Fruits	[142,155]
*Echidnopsis dammanniana* E.Dammann & Sprenger	Herb	General	[163]
*Gomphocarpus fruticosus* (L.) W.T.Aiton	Subshrub/shrub	General	[163]
*Hunteria zeylanica* (Retz.) Gardner ex Thwaites	Tree	Leaves (vegetable)	[158]
*Landolphia buchananii* (Hallier f.) Stapf	Shrub/tree	Fruits	[142,155,167]
*Landolphia kirkii* Dyer ex Hook.f.	Liana	Fruits; leaves (vegetable)	[142,155,158,167]
*Leptadenia lanceolata* (Poir.) Goyder	Liana	Leaves (vegetable)	[18,162,166,167]
*Marsdenia schimperi* Decne.	Liana	General	[162]
*Mondia whitei* (Hook.f.) Skeels	Liana	Roots	[155,167]
*Monolluma socotrana* (Balf.f.) Meve & Liede	Herb	Stems	[13,163]
*Orbea dummeri* (N.E.Br.) Bruyns	Herb	General	[163]
*Orbea tubiformis* (E.A.Bruce & P.R.O.Bally) Bruyns	Herb	General	[163]
*Pergularia daemia* (Forssk.) Chiov.	Herb	General	[163]
*Rauvolfia caffra* Sond.	Tree	Stems (ferment beer)	[155]
*Saba comorensis* (Bojer ex A.DC.) Pichon	Liana	Fruits; plant used to make sour milk	[16,142,155,158,161,165,167]
*Schlechterella africana* (Schltr.) K.Schum.	Herb	Tubers	[156]
*Stathmostelma pedunculatum* (Decne.) K.Schum.	Herb	Roots	[156]
*Stathmostelma propinquum* (N.E.Br.) Schltr.	Herb	Tubers	[167]
*Strophanthus mirabilis* Gilg	Shrub	Roots	[142,155]
*Tabernaemontana elegans* Stapf	Shrub/tree	Fruits	[155]
*Vincetoxicum diplostigma* Meve & Liede	Subshrub	General	[163]
*Vincetoxicum fruticulosum* (Decne.) Decne.	Shrub/subshrub	General	[163]
**Aquifoliaceae**			
*Ilex mitis* (L.) Radlk.	Shrub/tree	Roots (vegetable)	[156]
**Araceae**			
*Colocasia esculenta* (L.) Schott (exotic)	Herb	Tubers; leaves (vegetable)	[14,159,160]
*Gonatopus boivinii* (Decne.) Engl.	Herb	Famine food	[161]
*Stylochaeton borumense* N.E.Br.	Herb	General	[163]
**Arecaceae**			
*Areca catechu* L. (exotic)	Tree	Nuts	[163]
*Borassus aethiopum* Mart.	Tree	Fruit	[134,142,167]
*Cocos nucifera* L. (exotic)	Tree	Fruits	[16,158]
*Elaeis guineensis* Jacq.	Tree	Make wine	[142]
*Hyphaene compressa* H.Wendl.	Tree	Germinating seeds; fruit juice drunk; wine tapped stem	[18,161,162,163,165,167]
*Hyphaene coriacea* Gaertn. (exotic)	Shrub/tree	Fruits	[18,167]
*Hyphaene petersiana* Klotzsch ex Mart.	Tree	Fruit	[157]
*Phoenix reclinata* Jacq.	Tree	Fruit	[142,167]
**Aristolochiaceae**			
*Hydnora abyssinica* A.Br.	Herb	Tubers, fruits, buds	[16,18,100,162,163,166,167]
**Asparagaceae**			
*Asparagus buchananii* Baker	Shrub	General	[163]
*Asparagus falcatus* L.	Shrub	General	[164]
*Chlorophytum blepharophyllum* Schweinf. ex Baker	Herb	Tubers	[156]
*Chlorophytum macrophyllum* (A.Rich.) Asch.	Herb	Tubers	[156]
*Dracaena ellenbeckiana* Engl.	Shrub/tree	General	[163]
*Drimia indica* (Roxb.) Jessop	Herb	Tubers	[156,157,163]
*Ornithogalum gracillimum* R.E.Fr.	Herb	Tubers	[156]
**Asphodelaceae**			
*Aloe deserti* A.Berger	Shrub/subshrub	Ferment beer	[167]
*Aloe kedongensis* Reynolds	Shrub	Ferment beer	[167]
*Aloe ngongensis* Christian	Shrub	Ferment beer	[167]
*Aloe secundiflora* Engl.	Herb	Ferment beer; nectar, inflorescence	[167]
*Aloe turkanensis* Christian	Shrub	Roots used to flavor beer	[157,163]
*Aloe vera* (L.) Burm.f. (exotic)	Herb	Leaves	[18]
**Asteraceae**			
*Aspilia kotschyi* (Sch.Bip. ex Hochst.) Oliv.	Herb	Leaves (vegetable)	[159,160]
*Aspilia pluriseta* Schweinf. ex Engl.	Shrub	General	[164]
*Bidens pilosa* L.	Herb	Leaves (vegetable)	[79,159,160,161,163,167,169]
*Cyanthillium cinereum* (L.) H.Rob.	Herb	Leaves (vegetable)	[134,166,167]
*Dicoma tomentosa* Cass.	Herb	General	[163]
*Emilia discifolia* (Oliv.) C.Jeffrey	Herb	General	[163]
*Erigeron bonariensis* L. (exotic)	Herb	General	[163]
*Ethulia conyzoides* L.f.	Herb	Leaves (vegetable)	[134]
*Galinsoga parviflora* Cav. (exotic)	Herb	Leaves (vegetable)	[167]
*Gymnanthemum amygdalinum* (Delile) Sch.Bip.	Shrub/tree	Leaves (vegetable)	[167]
*Kleinia squarrosa* Cufod.	Shrub	General	[164]
*Lagascea mollis* Cav. (exotic)	Herb	Leaves (vegetable)	[159,160]
*Launaea cornuta* (Hochst. ex Oliv. & Hiern) C.Jeffrey	Herb	Leaves (vegetable)	[14,158,159,160,165,166,167]
*Solanecio angulatus* (Vahl) C.Jeffrey	Herb	Fruits	[158]
*Sonchus oleraceus* L. (exotic)	Herb	Leaves (vegetable)	[134,167]
*Sonchus schweinfurthii* Oliv. & Hiern	Herb	Leaves (vegetable)	[16,169]
*Vernonia popeana* C.Jeffrey	Herb	General	[163]
**Basellaceae**			
*Basella alba* L.	Herb	Leaves (vegetable)	[12,16,159,160,166,167,169]
**Bignoniaceae**			
*Kigelia africana* (Lam.) Benth.	Tree	Fruit (ferment beer)	[14,16,134,142,157,163,165,167]
**Boraginaceae**			
*Cordia africana* Lam.	Shrub/tree	Fruits	[13,142]
*Cordia crenata* Delile	Shrub/tree	Fruits	[142,157,163]
*Cordia monoica* Roxb.	Shrub/tree	Fruits	[142,163,164,167]
*Cordia quercifolia* Klotzsch	Shrub/tree	Fruits	[164]
*Cordia sinensis* Lam.	Shrub/tree	Fruits; fruits (make beer)	[18,100,142,156,157,162,163,164,167]
*Cordia somaliensis* Baker	Shrub/tree	Fruits	[142]
*Ehretia janjalle* Verdc.	Shrub	Fruits	[142]
*Euploca ovalifolia* (Forssk.) Diane & Hilger	Herb/subshrub	General	[163]
*Heliotropium longiflorum* (A.DC.) Jaub. & Spach	Subshrub	General	[163]
*Heliotropium zeylanicum* (Burm.f.) Lam.	Subshrub	General	[163]
*Trichodesma zeylanicum* (Burm.f.) R.Br.	Herb/subshrub	Leaves (vegetable)	[159,160]
**Brassicaceae**			
*Brassica carinata* A.Braun (exotic)	Herb	Leaves (vegetable)	[166]
*Brassica juncea* (L.) Czern.	Herb	Leaves (vegetable)	[160]
*Erucastrum arabicum* Fisch. & C.A.Mey.	Herb	Leaves (vegetable)	[159,160,166,167]
**Burseraceae**			
*Boswellia neglecta* S.Moore	Shrub/tree	Gum/resin, fruits; bark (tea)	[13,100,157,163,164,167]
*Commiphora africana* (A.Rich.) Engl.	Shrub/tree	Shoots, bark, roots, gum	[13,18,100,156,163,164,167]
*Commiphora baluensis* Engl.	Tree	Fruits, shoots	[13]
*Commiphora edulis* subsp. *boiviniana* (Engl.) J.B.Gillett	Shrub/tree	Fruits, shoots	[13,163,164]
*Commiphora eminii* subsp. *zimmermannii* (Engl.) J.B.Gillett	Tree	Fruits edible	[14]
*Commiphora hildebrandtii* Engl.	Shrub/tree	Roots chewed when thirsty	[156]
*Commiphora kataf* (Forssk.) Engl.	Shrub/tree	Fruits, shoots	[13]
*Commiphora kua* (R.Br. ex Royle) Vollesen	Shrub/tree	Stems, roots (chewed when thirsty)	[156,157,164]
*Commiphora mollis* (Oliv.) Engl.	Shrub/tree	Fruits, shoots, gum	[13]
*Commiphora myrrha* (T.Nees) Engl.	Shrub/tree	General	[163]
*Commiphora rostrata* Engl.	Shrub/tree	Leaves, bark, seeds; leaves (vegetables); bark (tea)	[18,142,157,166]
*Commiphora samharensis* Schweinf.	Shrub/tree	General	[163]
*Commiphora sarandensis* subsp. *moyaleensis* J.B.Gillett	Shrub	Root swellings chewed when thirsty	[142]
*Commiphora schimperi* (O.Berg) Engl.	Shrub/tree	Shoots, roots, gum; bark (tea)	[13,18,156,157,163,167]
**Cactaceae**			
*Opuntia ficus-indica* (L.) Mill. (exotic)	Shrub/tree	Fruits	[165]
*Opuntia humifusa* (Raf.) Raf. (exotic)	Subshrub	Fruits	[161]
**Campanulaceae**			
*Cyphia glandulifera* Hochst. ex A.Rich.	Herb	Tubers; leaves (vegetable)	[13,166,167]
*Lobelia fervens* Thunb.	Herb	Whole plant (vegetable)	[134]
**Canellaceae**			
*Warburgia ugandensis* Sprague	Tree	Bark, fruits	[18,142]
**Cannabaceae**			
*Celtis africana* Burm.f.	Tree	Fruits	[13]
*Trema orientale* (L.) Blume	Shrub/tree	Fruits	[142]
**Capparaceae**			
*Boscia coriacea* Graells	Shrub/tree	Fruits, cotyledons; twigs (flavor milk)	[18,100,142,157,162,163,164,167]
*Boscia integrifolia* J.St.-Hil.	Shrub/tree	Bark (tea; roots) eaten like potatoes.	[100,157,163]
*Boscia mazzocchii* (Chiov.) Fici & Kers	Shrub/tree	Fruits	[142]
*Cadaba farinosa* Forssk.	Shrub/tree	General	[163,164]
*Capparis erythrocarpos* Isert	Shrub	Fruits	[16]
*Capparis fascicularis* DC.	Shrub/tree	General	[163]
*Capparis tomentosa* Lam.	Shrub/tree	Fruits	[100,163]
*Crateva adansonii* DC.	Shrub/tree	Seeds	[142]
*Elaeodendron schlechterianum* (Loes.) Loes.	Shrub/tree	Fruits	[100]
*Elaeodendron schweinfurthianum* (Loes.) Loes.	Shrub/tree	Fruits	[100,142]
*Gymnosporia heterophylla* (Eckl. & Zeyh.) Loes.	Shrub	Fruits; roots (vegetable)	[100,142]
*Gymnosporia mossambicensis* (Klotzsch) Loes.	Shrub	Fruits	[100]
*Maerua angolensis* DC.	Shrub/tree	General	[164]
*Maerua denhardtiorum* Gilg		Fruits/nuts	[13,142]
*Maerua edulis* (Gilg & Gilg-Ben.) DeWolf	Herb	Fruits; roots used in soup/porridge; seeds, roots (water sweetener)	[13,16,18,100,142,162,164,165,167]
*Maerua kirkii* (Oliv.) F.White	Shrub/tree	Nuts	[13]
*Maerua subcordata* (Gilg) DeWolf	Shrub	Roots (chewed to quench thirst, boiled in broth, food); fruits	[156,157,163]
*Maerua triphylla* A.Rich.	Shrub/tree	Roots (porridge); fruits	[100]
*Maytenus acuminata* (L.f.) Loes.	Shrub/tree	Roots used in soup	[142]
*Maytenus undata* (Thunb.) Blakelock	Shrub/tree	Bark (tonic)	[142]
*Mystroxylon aethiopicum* (Thunb.) Loes.	Tree	Fruits	[16,100,142]
*Salacia stuhlmanniana* Loes.	Shrub/tree	Fruits	[100]
*Thilachium africanum* Lour.	Shrub/tree	Fruits, roots (porridge)	[13,100,134,142,156,161]
*Thilachium thomasii* Gilg	Shrub/tree	Fruits, roots (porridge)	[100,167]
**Caricaceae**			
*Carica papaya* L. (exotic)	Tree	Leaves (vegetable)	[160]
**Caryophyllaceae**			
*Pollichia campestris* Aiton	Subshrub	Fruits	[13]
**Celastraceae**			
*Catha edulis* (Vahl) Endl.	Shrub/tree	Leaves, shoots (stimulants)	[142,167]
*Gymnosporia senegalensis* (Lam.) Loes.	Shrub/tree	Fruits, leaves, roots (vegetable)	[18,100,156]
*Salacia elegans* Welw. ex Oliv.	Liana	Seed/fruit	[134,142]
*Salacia erecta* (G.Don) Walp.	Liana	Fruit	[167]
*Salacia madagascariensis* (Lam.) DC.	Liana	Fruits	[142,167]
**Chrysobalanaceae**			
*Hirtella zanzibarica* Oliv.	Tree	Seed/fruit	[134,142]
*Parinari curatellifolia* Planch. ex Benth.	Tree	Fruits	[134,142,167]
**Cleomaceae**			
*Cleome allamanii* Chiov.	Herb	General	[163]
*Cleome angustifolia* Forssk.	Herb	General	[163,164]
*Cleome brachycarpa* Vahl ex DC.	Herb	General	[164]
*Cleome gynandra* L.	Herb	Leaves (vegetable)	[12,16,18,159,160,163,166,167,169]
*Cleome hirta* (Klotzsch) Oliv.	Herb	Leaves (vegetable)	[159,160,166]
*Cleome monophylla* L.	Herb	Leaves (vegetable)	[166]
*Cleome strigosa* (Bojer) Oliv.	Herb	Leaves (vegetable)	[134]
**Clusiaceae**			
*Garcinia buchananii* Baker	Shrub/tree	Fruit	[142]
*Garcinia livingstonei* T.Anderson	Tree	Seed/fruit	[127,134,142,167]
**Colchicaceae**			
*Colchicum melanthioides* (Willd.) J.C.Manning & Vinn. (exotic)	Herb	Corms	[156]
**Combretaceae**			
*Combretum aculeatum* Vent.	Shrub/tree	Seeds, leaves, fruits	[18,100,157,163,165]
*Combretum constrictum* (Benth.) M.A.Lawson	Shrub	General	[164]
*Combretum molle* R.Br. ex G.Don	Shrub/tree	General	[163]
*Terminalia brownii* Fresen.	Tree	Fruits	[142,163]
*Terminalia catappa* L.	Tree	Fruits	[142]
*Terminalia spinosa* Engl.	Tree	Bark (tea)	[157,163]
**Commelinaceae**			
*Commelina africana* L.	Herb	Leaves (vegetable)	[16,159,160,166,167]
*Commelina benghalensis* L.	Herb	Leaves (vegetable)	[16,159,160,165,166,167]
*Commelina forskaolii* Vahl	Herb	Leaves (vegetable)	[163,166]
*Commelina imberbis* Ehrenb. ex Hassk.	Herb	Leaves (vegetable)	[166]
**Connaraceae**			
*Rourea minor* (Gaertn.) Alston	Shrub/liana	Seed aril	[142]
**Convolvulaceae**			
*Ipomoea aquatica* Forssk.	Herb	Leaves (vegetable)	[166,167]
*Ipomoea batatas* (L.) Lam. (exotic)	Herb	Leaves (vegetable)	[160]
*Ipomoea biflora* (L.) Pers.	Herb	Tubers; leaves (vegetable)	[156,159,160,163]
*Ipomoea donaldsonii* Rendle	Shrub	General	[164]
*Ipomoea elythrocephala* Hallier f.	Herb	Roots	[13]
*Ipomoea hildebrandtii* Vatke	Shrub/subshrub	General	[163]
*Ipomoea kituiensis* Vatke	Shrub	General	[164]
*Ipomoea lapathifolia* Hallier f.	Herb	Tubers	[167]
*Ipomoea longituba* Hallier f.	Shrub	Tubers	[13,156,167]
*Ipomoea mombassana* Vatke	Herb	Leaves (vegetable)	[18,162,165,166,167]
*Ipomoea obscura* (L.) Ker Gawl.	Herb	General	[164]
*Ipomoea oenotherae* (Vatke) Hallier f.	Herb	Tubers	[13,16,156,167]
*Ipomoea pes-caprae* (L.) R.Br.	Herb	Leaves (vegetable)	[134]
*Ipomoea spathulata* Hallier f.	Shrub	Roots used in soup	[142,163,164]
*Jacquemontia tamnifolia* (L.) Griseb.	Subshrub	Leaves (vegetable)	[134]
*Seddera latifolia* Hochst. & Steud.	Subshrub	General	[163]
**Crassulaceae**			
*Kalanchoe lanceolata* (Forssk.) Pers.	Herb	General	[164]
**Cucurbitaceae**			
*Citrullus lanatus* (Thunb.) Matsum. & Nakai	Herb	Fruit, leaves, seeds	[158,162,167]
*Coccinia adoensis* (Hochst. ex A.Rich.) Cogn.	Herb	Leaves (vegetable)	[16,114,169]
*Coccinia grandiflora* Cogn.	Herb	Roots	[13]
*Coccinia grandis* (L.) Voigt	Herb	Fruits; leaves (vegetable)	[18,157,159,160,162,163,166,167]
*Coccinia trilobata* (Cogn.) C.Jeffrey	Herb	Fruits edible, leafy vegetable	[114,167]
*Cucumis aculeatus* Cogn.	Herb	Leaves (vegetable)	[114,163]
*Cucumis dipsaceus* Ehrenb. ex Spach	Herb	Leaves (vegetable)	[18,114,157,162,163,164,166,167]
*Cucumis engleri* (Gilg) Ghebret. & Thulin	Herb	General	[163]
*Cucumis ficifolius* A.Rich.	Herb	Leaves (vegetable)	[166]
*Cucumis melo* L. (exotic)	Herb	Fruits (vegetable)	[16,134]
*Cucumis prophetarum* L.	Herb	Fruits	[114]
*Cucumis pustulatus* Naudin ex Hook.f.	Herb	General	[162,163,164]
*Cucurbita maxima* Duchesne (exotic)	Herb	Leaves (vegetable)	[160]
*Diplocyclos palmatus* (L.) C.Jeffrey	Herb	Leaves (vegetable)	[114]
*Kedrostis foetidissima* (Jacq.) Cogn.	Herb	Leaves (vegetable)	[114,163]
*Kedrostis gijef* (Forssk. ex J.F.Gmel.) C.Jeffrey	Liana	Leaves (vegetable)	[114,162,166]
*Kedrostis pseudogijef* (Gilg) C.Jeffrey	Liana	Fruits; leaves (vegetable)	[166,167]
*Lagenaria siceraria* (Molina) Standl.	Herb	Fruits, seeds; leaves (vegetable)	[134,166,167]
*Lagenaria sphaerica* (Sond.) Naudin	Herb	Leaves (vegetable)	[114]
*Momordica calantha* Gilg	Herb	Fruits	[114]
*Momordica charantia* L.	Herb	Fruits; leaves (vegetable)	[134]
*Momordica foetida* Schumach.	Herb	Fruits	[114]
*Momordica friesiorum* (Harms) C.Jeffrey	Herb	General	[163]
*Momordica rostrata* Zimm.	Herb	Fruits; leaves (vegetable, tea)	[114,163,167]
*Momordica spinosa* (Gilg) Chiov.	Shrub	Fruits	[142]
*Momordica trifoliolata* Hook.f.	Herb	Fruits	[157,163]
*Peponium vogelii* (Hook.f.) Engl.	Herb	Fruits	[13,114]
*Zehneria anomala* C.Jeffrey	Herb	Fruits	[13]
**Cyperaceae**			
*Cyperus alatus* subsp. *albus* (Nees) Lye	Herb	Bulbs	[13]
*Cyperus amauropus* Steud.	Herb	General	[163]
*Cyperus blysmoides* Hochst. ex C.B.Clarke	Herb	Roots/bulbs	[156,165,167]
*Cyperus comosipes* Mattf. & Kük. (exotic)	Herb	Roots	[13]
*Cyperus rotundus* L.	Herb	General	[163]
*Cyperus rubicundus* Vahl	Herb	General	[163]
**Dichapetalaceae**			
*Dichapetalum zenkeri* Engl.	Shrub/tree	Fruits	[127]
**Didiereaceae**			
*Calyptrotheca somalensis* Gilg	Shrub	Root juice (quench thirst)	[142]
**Dilleniaceae**			
*Tetracera litoralis* Gilg	Shrub/liana	Fruits	[158]
**Dioscoreaceae**			
*Dioscorea bulbifera* L. (exotic)	Herb	Roots/tubers	[167]
*Dioscorea cayenensis* subsp. *rotundata* (Poir.) J.Miège (exotic)	Herb	Roots/tubers	[14]
*Dioscorea dumetorum* (Kunth) Pax	Herb	Tubers; leaves (vegetable)	[127,134,158,167]
*Dioscorea minutiflora* Engl. (exotic)	Herb	Tubers	[167]
*Dioscorea schimperiana* Hochst. ex Kunth	Herb	Bulbs	[156]
**Ebenaceae**			
*Diospyros bussei* Gürke	Tree	Fruits	[127]
*Diospyros consolatae* Chiov.	Shrub/tree	Fruits	[100]
*Diospyros mespiliformis* Hochst. ex A.DC.	Tree	Fruits/seeds	[165,167]
*Diospyros natalensis* (Harv.) Brenan	Shrub/tree	Fruits	[127]
*Diospyros squarrosa* Klotzsch	Shrub/tree	Fruits	[127]
*Diospyros wajirensis* F.White	Shrub/tree	Fruits	[142]
*Euclea divinorum* Hiern	Shrub/tree	Fruits	[100,167]
*Euclea racemosa* L.	Shrub/tree	Fruits	[100]
**Euphorbiaceae**			
*Acalypha indica* L.	Herb	Leaves (vegetable)	[157]
*Acalypha volkensii* Pax	Shrub/subshrub	Leaves (vegetable)	[16,169]
*Croton dichogamus* Pax	Shrub/tree	Roots used in soup	[142,156]
*Croton pseudopulchellus* Pax	Shrub/tree	Stem smoke (flavor milk)	[127]
*Erythrococca bongensis* Pax	Shrub/tree	Leaves (vegetable)	[16,159,160,169]
*Erythrococca fischeri* Pax	Shrub/tree	General	[163]
*Erythrococca kirkii* (Müll.Arg.) Prain	Shrub/tree	Leaves (vegetable)	[142]
*Euphorbia bussei* Pax	Tree	Roasted roots	[100]
*Euphorbia candelabrum* Welw.	Tree	Roasted roots	[100]
*Euphorbia scheffleri* Pax	Shrub/tree	Meat tenderizer	[142]
*Givotia gosai* Radcl.-Sm.	Tree	Fruits	[142]
*Manihot esculenta* Crantz (exotic)	Shrub/tree	Leaves (vegetable)	[160]
*Suregada zanzibariensis* Baill.	Shrub	Fruits	[158]
*Tragia impedita* Prain	Shrub	Fruits	[165]
**Fabaceae**			
*Albizia adianthifolia* (Schumach.) W.Wight	Tree	Leaves (vegetable)	[158]
*Albizia amara* (Roxb.) Boivin	Tree	Gum; stems used in soup	[163,167]
*Albizia anthelmintica* (A.Rich.) Brongn.	Shrub/tree	Leaves (vegetable)	[158]
*Astragalus atropilosulus* (Hochst.) Bunge	Subshrub	Roots chewed	[156]
*Cajanus cajan* (L.) Huth	Shrub	Seeds, fruits, leaves	[16,158,167]
*Clitoria ternatea* L.	Subshrub	Leaves (vegetable)	[165]
*Craibia brownii* Dunn	Tree	Seeds	[142]
*Craibia laurentii* (De Wild.) De Wild.	Shrub/tree	Bean	[13]
*Crotalaria brevidens* Benth.	Herb/subshrub	Leaves (vegetable)	[16,160,166,167,169]
*Crotalaria lanceolata* E.Mey.	Herb	Leaves (vegetable)	[159]
*Crotalaria ochroleuca* G.Don	Herb/subshrub	Leaves (vegetable)	[159,160,166,167]
*Crotalaria polysperma* Kotschy	Herb	General	[163]
*Crotalaria pycnostachya* Benth.	Herb	Leaves (vegetable)	[159]
*Delonix elata* (L.) Gamble	Shrub/tree	General	[163,164]
*Dialium holtzii* Harms	Tree	Fruits	[167]
*Dialium orientale* Baker f.	Shrub/tree	Fruits	[142,158,167]
*Entada leptostachya* Harms	Liana	Stem water drunk	[100]
*Eriosema shirense* Baker f.	Herb	Tubers	[167]
*Erythrina abyssinica* Lam.	Tree	Roots (tea)	[142]
*Faidherbia albida* (Delile) A.Chev.	Shrub/tree	General	[163]
*Indigofera lupatana* Baker f.	Shrub	Leaves (vegetable)	[165]
*Indigofera schimperi* Jaub. & Spach	Shrub	Tubers	[18]
*Indigofera spinosa* Forssk.	Shrub/subshrub	General	[164]
*Lablab purpureus* (L.) Sweet	Herb	Leaves (vegetable); beans	[13,166,167]
*Macrotyloma maranguense* (Taub.) Verdc.	Herb	Roots	[156]
*Mucuna gigantea* (Willd.) DC.	Shrub	Bean	[13,142]
*Ormocarpum kirkii* S.Moore	Shrub/tree	Leaves (vegetable)	[158,164]
*Ormocarpum trachycarpum* (Taub.) Harms	Shrub/tree	General	[164]
*Parkia filicoidea* Welw. ex Oliv.	Tree	Fruit	[142]
*Phaseolus vulgaris* L. (exotic)	Herb	Leaves (vegetable)	[160]
*Philenoptera eriocalyx* (Harms) Schrire	Shrub/tree		[163]
*Piliostigma thonningii* (Schumach.) Milne-Redh.	Shrub/tree	General	[134,165,167]
*Prosopis juliflora* (Sw.) DC. (exotic)	Shrub/tree	General	[162]
*Senegalia mellifera* (Benth.) Seigler & Ebinger	Shrub/tree	Gum	[142,157,162,163]
*Senegalia polyacantha* (Willd.) Seigler & Ebinger	Tree	Gum	[142]
*Senegalia senegal* (L.) Britton	Shrub/tree	Gum, fruits	[13,18,142,157,162,163,164,165,167]
*Senna bicapsularis* (L.) Roxb. (exotic)	Shrub	Leaves (vegetable)	[16]
*Senna didymobotrya* (Fresen.) H.S.Irwin & Barneby	Shrub/tree	Leaves (vegetable)	[165]
*Senna italica* Mill.	Herb/subshrub	General	[163]
*Senna obtusifolia* (L.) H.S.Irwin & Barneby	Herb/subshrub	General	[163]
*Senna occidentalis* (L.) Link (exotic)	Herb	Leaves (vegetable)	[16,158,159,160,163,169]
*Senna singueana* (Delile) Lock	Shrub/tree	Fruit	[142]
*Tamarindus indica* L.	Tree	Fruit, leaves, seeds; fruit (food spice, vinegar substitute)	[14,16,18,134,142,158,161,162,163,165,166,167]
*Tephrosia nubica* (Boiss.) Baker	Subshrub	General	[163]
*Tephrosia pumila* (Lam.) Pers.	Herb	Leaves (vegetable)	[16]
*Tephrosia villosa* subsp. *ehrenbergiana* (Schweinf.) Brummitt	Herb	General	[163]
*Tylosema fassoglense* (Kotschy ex Schweinf.) Torre & Hillc.	Herb	Fruit/seeds	[167]
*Vachellia bussei* (Harms ex Y.Sjöstedt) Kyal. & Boatwr.	Tree	Bark (tea)	[100]
*Vachellia drepanolobium* (Harms ex Y.Sjöstedt) P.J.H.Hurter	Shrub/tree	Galls, fruits, inner bark	[13,142,167]
*Vachellia elatior* (Brenan) Kyal. & Boatwr.	Tree	Bark (tea)	[18,157,163]
*Vachellia etbaica* (Schweinf.) Kyal. & Boatwr.	Shrub/tree	Gum	[157,163]
*Vachellia gerrardii* (Benth.) P.J.H.Hurter	Shrub/tree	Bark used in soup	[142]
*Vachellia hockii* (De Wild.) Seigler & Ebinger	Shrub/tree	Bark	[142,167]
*Vachellia horrida* (L.) Kyal. & Boatwr.	Shrub/tree	Galls	[13]
*Vachellia kirkii* (Oliv.) Kyal. & Boatwr.	Shrub/tree	Bark (tea)	[142]
*Vachellia nilotica* (L.) P.J.H.Hurter & Mabb.	Shrub/tree	Pods; pod, bark (used in tea)	[100,163,167]
*Vachellia oerfota* (Forssk.) Kyal. & Boatwr.		General	[162]
*Vachellia paolii* (Chiov.) Kyal. & Boatwr.	Shrub	Gum	[142]
*Vachellia reficiens* (Wawra & Peyr.) Kyal. & Boatwr.	Shrub/tree	Bark/gum	[13,18,142,162,164]
*Vachellia seyal* (Delile) P.J.H.Hurter	Shrub/tree	Gum; bark (tea)	[142,162,163,167]
*Vachellia sieberiana* (DC.) Kyal. & Boatwr.	Tree	Gum	[142]
*Vachellia tortilis* (Forssk.) Galasso & Banfi	Shrub/tree	Fruits/seeds, gum	[13,18,142,157,162,163,164,167]
*Vachellia xanthophloea* (Benth.) Banfi & Galasso	Tree	Gum	[142]
*Vatovaea pseudolablab* (Harms) J.B.Gillett	Herb	Fruits, flowers leaves (vegetables); tubers	[13,18,142,156,157,162,163,166,167]
*Vigna friesiorum* Harms	Herb	Tubers	[167]
*Vigna frutescens* A.Rich.	Herb	Roots	[13,156]
*Vigna luteola* (Jacq.) Benth.	Herb	Leaves (vegetable)	[159,160]
*Vigna membranacea* A.Rich.	Herb	Leaves (vegetable); roots	[13,156,163,165,166,167]
*Vigna radiata* (L.) R.Wilczek	Herb	General	[163]
*Vigna schimperi* Baker	Herb	Leaves (vegetable)	[159]
*Vigna subterranea* (L.) Verdc.	Herb	Seeds edible	[167]
*Vigna unguiculata* (L.) Walp.	Herb	Leaves (vegetable); seeds	[16,158,159,160,163,166,167,169]
*Vigna vexillata* (L.) A.Rich.	Herb	Tubers chewed when thirsty	[156]
*Wajira grahamiana* (Wight & Arn.) Thulin & Lavin	Herb	Roots	[156]
*Wajira praecox* (Verdc.) Thulin & Lavin	Herb	Roots	[13]
**Geraniaceae**			
*Pelargonium quinquelobatum* Hochst. ex A.Rich.	Herb	Leaves (vegetable)	[13]
**Hydrocharitaceae**			
*Ottelia ulvifolia* (Planch.) Walp.	Herb	Leaves (vegetable)	[159,169]
**Hypericaceae**			
*Harungana madagascariensis* Lam. ex Poir.	Shrub/tree	Fruits	[14,134]
**Hypoxidaceae**			
*Hypoxis angustifolia* Lam.	Herb	Corms	[156]
*Hypoxis urceolata* Nel	Herb	Corms	[156]
**Icacinaceae**			
*Pyrenacantha kaurabassana* Baill.	Herb	Leaves (vegetable)	[165]
**Kirkiaceae**			
*Kirkia tenuifolia* Engl.	Shrub/tree	Bark chewed when thirsty	[142]
**Lamiaceae**			
*Hoslundia opposita* Vahl	Shrub	Fruits, leaves (tea)	[13,16,100,142,161,163,167]
*Leucas glabrata* (Vahl) Sm.	Subshrub	Leaves (vegetable)	[158,163,164]
*Leucas jamesii* Baker	shrub	General	[164]
*Leucas urticifolia* (Vahl) Sm.	Herb	General	[163]
*Mesosphaerum pectinatum* (L.) Kuntze	Herb/subshrub	General	[163]
*Ocimum americanum* L.	Herb/subshrub	Seeds/leaves	[134]
*Ocimum basilicum* L.	Herb/subshrub	Leaves (flavor tea)	[167]
*Ocimum gratissimum* L.	Subshrub	Leaves (flavor tea)	[167]
*Ocimum kilimandscharicum* Gürke	Shrub	Leaves (flavor tea)	[142,167]
*Premna chrysoclada* (Bojer) Gürke	Shrub/tree	Fruits	[134,142]
*Premna oligotricha* Baker	Shrub	Fruits	[13]
*Premna resinosa* (Hochst.) Schauer	Shrub	Fruits/seeds	[13,134,142,163]
*Vitex doniana* Sweet	Tree	Fruits	[142,163,167]
*Vitex ferruginea* Schumach. & Thonn.	Shrub/tree	Fruits	[142,167]
*Vitex fischeri* Gürke	Shrub/tree	Fruits	[142]
*Vitex mombassae* Vatke	Shrub/tree	Fruits/seeds	[134,142,161,167]
*Vitex payos* (Lour.) Merr.	Shrub/tree	Fruits	[14,142,161,165,167]
*Vitex strickeri* Vatke & Hildebrandt	Shrub/tree	Fruits	[142]
**Limeaceae**			
*Limeum viscosum* var. *kenyense* Friedrich	Herb	General	[163]
**Loganiaceae**			
*Strychnos bredemeyeri* (Schult.) Sprague & Sandwith	Liana	Tubers	[167]
*Strychnos decussata* (Pappe) Gilg	Shrub/tree	Fruits	[100,142]
*Strychnos henningsii* Gilg	Shrub/tree	Fruits (ferment beer)	[167]
*Strychnos innocua* Delile	Shrub/tree	Fruits	[142,167]
*Strychnos madagascariensis* Poir.	Tree	Fruits	[142,158,165,167]
*Strychnos spinosa* Lam.	Shrub/tree	Fruits	[167]
**Loranthaceae**			
*Oncocalyx fischeri* (Engl.) M.G.Gilbert	Shrub	General	[163]
*Oncocalyx ugogensis* (Engl.) Wiens & Polhill	Shrub	General	[163]
**Lythraceae**			
*Sonneratia alba* Sm.	Tree	Fruits	[142]
*Lawsonia inermis* L.	Shrub/tree	General	[163]
**Malpighiaceae**			
*Acridocarpus zanzibaricus* A.Juss.	Shrub	Leaves (vegetable)	[158]
**Malvaceae**			
*Abutilon fruticosum* Guill. & Perr.	Herb/subshrub	General	[163]
*Abutilon hirtum* (Lam.) Sweet	Herb/subshrub	General	[163]
*Abutilon mauritianum* (Jacq.) Medik.	Shrub	General	[163]
*Adansonia digitata* L.	Tree	Seeds; leaves, shoots, root tips, germinating seeds (vegetables); seed pulp (curdle milk, flavor food)	[14,100,134,142,156,161,165,166,167]
*Corchorus olitorius* L.	Herb	Leaves (vegetable)	[16,18,158,159,160,161,166,167]
*Corchorus tridens* L.	Herb	Leaves (vegetable)	[158,166]
*Corchorus trilocularis* L.	Herb	Leaves (vegetable)	[16,162,166,167,169]
*Grewia arborea* (Forssk.) Lam.	Shrub/tree	Fruits	[142]
*Grewia capitellata* Bojer	Shrub/tree	Fruits	[142]
*Grewia forbesii* Harv. ex Mast.	Shrub/tree	Fruits	[100]
*Grewia kakothamnos* K.Schum.	Shrub	Fruits	[13]
*Grewia lilacina* K.Schum.	Shrub/tree	Fruits	[13,142,164]
*Grewia mollis* Juss.	Shrub/tree	Fruits	[18,142,162]
*Grewia nematopus* K.Schum.	Shrub	Fruits	[100]
*Grewia penicillata* Chiov.	Shrub	Fruits	[142]
*Grewia plagiophylla* K.Schum.	Shrub/tree	Fruits	[100,161]
*Grewia similis* K.Schum.	Shrub/tree	Fruits	[13,16,100,142,165]
*Grewia tembensis* Fresen.	Shrub	Fruits	[13,142,163,167]
*Grewia tenax* (Forssk.) Fiori	Shrub/tree	Fruits	[13,18,142,157,162,163,164,167]
*Grewia tephrodermis* K.Schum.	Shrub/tree	Fruits	[13,100,142,157,163,164,165,167]
*Grewia trichocarpa* Hochst. ex A.Rich.	Shrub/tree	Fruits	[13,16,163]
*Grewia truncata* Mast.	Shrub/tree	Fruits	[100]
*Grewia villosa* Willd.	Shrub	Fruits	[13,14,18,100,134,157,162,163,165,167]
*Hibiscus calyphyllus* Cav.	Shrub	Leaves (vegetable)	[159]
*Hibiscus greenwayi* Baker f.	Shrub/tree	Stems	[13]
*Hibiscus micranthus* L.f.	Shrub	General	[163]
*Hibiscus palmatus* Forssk.	Herb	General	[163]
*Hibiscus vitifolius* L.	Herb/shrub	General	[163]
*Melhania ovata* (Cav.) Spreng.	Herb/subshrub	General	[163]
*Sida acuta* Burm.f.	Herb/subshrub	Leaves (vegetable)	[16,159,160]
*Sida rhombifolia* L.	Herb/subshrub	Leaves (vegetable)	[159,160]
*Sterculia africana* (Lour.) Fiori	Tree	Fruits	[18,100,162,164,165]
*Sterculia rhynchocarpa* K.Schum.	Shrub/tree	Fruits	[157,163]
*Sterculia stenocarpa* H.J.P.Winkl.	Tree	Fruits, leaves	[13,14,142,163]
*Thespesia danis* Oliv.	Shrub/tree	Fruits	[134,158,161]
*Thespesia garckeana* F.Hoffm.	Shrub/tree	Fruits	[14,142,165,167]
*Triumfetta annua* L.	Herb/subshrub	Leaves (vegetable)	[134]
**Meliaceae**			
*Trichilia emetica* Vahl	Tree	Fruits	[157,163]
**Menispermaceae**			
*Chasmanthera dependens* Hochst.	Liana	Roots, stems	[13,142,156,163]
*Cissampelos mucronata* A.Rich.	Liana	General	[163]
*Cocculus hirsutus* (L.) W.Theob.	Liana	General	[162]
*Hyalosepalum caffrum* (Miers) Troupin	Liana	Stems	[13]
**Moraceae**			
*Dorstenia hildebrandtii* var. *schlechteri* (Engl.) Hijman	Herb	Tubers	[13]
*Ficus amadiensis* De Wild.	Tree	Fruits	[142]
*Ficus capreifolia* Delile	Tree	Fruits	[142]
*Ficus glumosa* Delile	Tree	Fruits	[13,142]
*Ficus ingens* (Miq.) Miq.	Tree	Fruits	[100]
*Ficus lutea* Vahl	Tree	General	[163]
*Ficus natalensis* Hochst.	Tree	Fruits	[13,167]
*Ficus populifolia* Vahl	Tree	Fruits	[142]
*Ficus salicifolia* Vahl	Shrub/tree	Fruits	[142]
*Ficus sur* Forssk.	Tree	Fruits	[13,16,100,142,163,165,167]
*Ficus sycomorus* L.	Tree	Fruits	[13,14,18,100,142,157,162,164,166,167]
*Ficus thonningii* Blume	Tree	Fruits	[14,100,142,163,166]
*Ficus vallis-choudae* Delile	Tree	Fruits	[167]
*Ficus vasta* Forssk.	Tree	Fruits	[142]
**Moringaceae**			
*Moringa oleifera* Lam.	Tree	Root powder (flavor); seeds, leaves (vegetable)	[134,142,158,166,167]
*Moringa stenopetala* (Baker f.) Cufod.	Tree	Leaf/seeds (vegetable); seeds (purify water)	[167]
**Myrtaceae**			
*Psidium guajava* L. (exotic)	Tree	Fruits	[18]
*Syzygium cordatum* Hochst. ex Krauss	Shrub/tree	Leaves (tea); fruits	[14,142,167]
*Syzygium cumini* (L.) Skeels	Tree	Fruits	[16]
*Syzygium guineense* (Willd.) DC.	Tree	Fruits	[14,167]
**Nyctaginaceae**			
*Boerhavia diffusa* L.	Herb	Leaves (vegetable)	[159,160]
**Nymphaeaceae**			
*Nymphaea lotus* L.	Herb	Tubers	[156,157,163]
*Nymphaea nouchali* var. *caerulea* (Savigny) Verdc.	Herb	Roots, fruits, seeds, flowers	[134,156,167]
**Ochnaceae**			
*Ochna atropurpurea* DC.	Shrub/tree	Seeds/fruit	[134,161]
*Ochna inermis* (Forssk.) Schweinf.	Shrub/tree	General	[163]
**Olacaceae**			
*Strombosia scheffleri* Engl.	Tree	Seeds	[142]
*Ximenia americana* L.	Shrub/tree	Fruits/seeds	[13,14,16,18,100,134,161,162,163,165,167]
*Ximenia caffra* Sond.	Shrub/tree	Fruits	[13,14,142,163]
**Oleaceae**			
*Jasminum abyssinicum* Hochst. ex DC.	Liana	Roots used in soup	[156]
*Olea capensis* subsp. *macrocarpa* (C.H.Wright) I.Verd.	Shrub/tree	Fruits	[142]
*Olea europaea* L.	Shrub/tree	Fruits	[142]
*Olea welwitschii* (Knobl.) Gilg & G.Schellenb.	Tree	Fruits	[142]
**Onagraceae**			
*Ludwigia adscendens* (L.) H.Hara	Subshrub	Leaves (vegetable)	[16,159,160,169]
**Opiliaceae**			
*Opilia amentacea* Roxb.	Liana	Fruits	[100,142]
*Opilia campestris* Engl.	Shrub/tree	Fruits	[13,142]
*Pentarhopalopilia umbellulata* (Baill.) Hiepko	Shrub	Fruits	[142]
**Orobanchaceae**			
*Ghikaea speciosa* (Rendle) Diels	Herb	General	[163]
*Pseudosopubia hildebrandtii* (Vatke) Engl.	Herb	General	[163]
**Oxalidaceae**			
*Oxalis latifolia* Kunth	Herb	Leaves (vegetable)	[159,160]
**Pandanaceae**			
*Pandanus rabaiensis* Rendle.	Tree	Fruits	[142]
**Passifloraceae**			
*Adenia ellenbeckii* Harms	Herb	General	[163]
*Adenia venenata* Forssk.	Shrub	Fruits	[142]
*Adenia volkensii* Harms	Herb/shrub	Fruit, seeds, leaves	[18,157,162,163]
*Basananthe hanningtoniana* (Mast.) J.J.de Wilde	Herb	General	[163]
**Pedaliaceae**			
*Sesamum angustifolium* (Oliv.) Engl.	Herb	Leaves (vegetable)	[16,160,166,169]
*Sesamum calycinum* Welw.	Herb	Leaves (vegetable)	[159,167]
*Sesamum indicum* L.	Herb	Seeds; leaves (vegetable)	[158,159,160,167]
**Peraceae**			
*Clutia abyssinica* Jaub. & Spach	Shrub/tree	Roots used in soup	[156]
**Phyllanthaceae**			
*Antidesma venosum* E.Mey. ex Tul.	Shrub/tree	Fruits/seeds	[134,142,167]
*Bridelia cathartica* Bertol.	Shrub/tree	Fruits; leaves (vegetable)	[142,158,161]
*Bridelia micrantha* (Hochst.) Baill.	Tree	Fruits	[16,100]
*Bridelia scleroneura* Müll.Arg.	Shrub/tree	Fruit	[142]
*Bridelia taitensis* Vatke & Pax	Shrub/tree	Fruits	[14,100,142,165,167]
*Flueggea virosa* (Roxb. ex Willd.) Royle	Shrub/tree	Fruits	[13,16,134,142,157,163,165,167]
**Piperaceae**			
*Piper capense* L.f.	Shrub/tree	Roots (vegetable)	[156]
**Pittosporaceae**			
*Pittosporum viridiflorum* Sims	Tree	Fruits	[142]
**Poaceae**			
*Cenchrus americanus* (L.) Morrone (exotic)	Herb	Seeds	[18,167]
*Cymbopogon citratus* (DC.) Stapf (exotic)	Herb	Leaves	[16]
*Cymbopogon giganteus* Chiov.	Herb	General	[163]
*Dactyloctenium aegyptium* (L.) Willd.	Herb	Grain	[163,167]
*Dactyloctenium geminatum* Hack.	Herb	Rhizomes	[40,156]
*Dactyloctenium giganteum* B.S.Fisher & Schweick.	Herb	Grain	[167]
*Echinochloa haploclada* (Stapf) Stapf	Herb	General	[163]
*Eleusine coracana* (L.) Gaertn. (exotic)	Herb	Grain	[163,167]
*Eragrostis cilianensis* (All.) Vignolo ex Janch.	Herb	General	[163]
*Eragrostis tef* (Zuccagni) Trotter	Herb	Grain	[167]
*Hyparrhenia cymbaria* (L.) Stapf	Herb	Leaves (vegetable)	[16]
*Sorghum bicolor* (L.) Moench (exotic)	Herb	Tubers, grains	[18,163,167]
*Sporobolus macranthelus* Chiov.	Herb	General	[163]
*Urochloa deflexa* (Schumach.) H.Scholz	Herb	General	[163]
**Polygalaceae**			
*Carpolobia goetzei* Gürke	Shrub/tree	Seed/fruit	[134,142]
**Polygonaceae**			
*Oxygonum salicifolium* Dammer	Herb	Leaves (vegetable)	[166,167]
*Oxygonum sinuatum* (Hochst. & Steud. ex Meisn.) Dammer	Herb	Leaves (vegetable)	[13,16,159,160,163,165,166,167,169]
*Persicaria senegalensis* (Meisn.) Soják	Herb	Leaves (vegetable)	[159,160]
*Rumex abyssinicus* Jacq.	Herb	Leaves (vegetable)	[167]
*Rumex nepalensis* Spreng.	Herb	Leaves (vegetable)	[12,166]
*Rumex usambarensis* (Engl.) Dammer	Shrub/subshrub	Leaves (vegetable); stems, leaves (chewed)	[12,142,166,167]
**Portulacaceae**			
*Portulaca oleracea* L.	Herb	Vegetable, salad; grain (flour)	[162,166,167]
*Portulaca quadrifida* L.	Herb	Vegetable, salad; grain (flour)	[16,159,160,163,166,167,169]
**Primulaceae**			
*Maesa lanceolata* Forssk.	Tree	Fruit	[142]
*Myrsine africana* L.	Shrub/tree	Bark, seeds	[18]
**Putranjivaceae**			
*Drypetes gerrardii* Hutch.	Tree	Fruits	[13]
**Rhamnaceae**			
*Phyllogeiton discolor* (Klotzsch) Herzog	Shrub/tree	Fruits, gum	[13,14,18,100,142,157,162,163,165,167]
*Rhamnus prinoides* L’Hér.	Shrub/tree	Roots boiled in soup	[156]
*Scutia myrtina* (Burm.f.) Kurz	Shrub/tree	Fruits; roots boiled in soup	[16,142,167]
*Ziziphus abyssinica* Hochst. ex A.Rich.	Shrub/tree	Fruits	[100,142,167].
*Ziziphus jujuba* Mill.	Shrub/tree	Fruits	[18]
*Ziziphus mauritiana* Lam.	Tree	Fruits/seeds	[134,142,157,158,162,163,167]
*Ziziphus mucronata* Willd.	Shrub/tree	Fruits	[13,14,100,142,163,164,165,167]
*Ziziphus pubescens* Oliv.	Shrub/tree	Fruits	[142,161]
**Rosaceae**			
*Rubus apetalus* Poir.	Shrub	Fruits	[142,167]
*Rubus niveus* Thunb.	Shrub	Fruits	[167]
*Rubus pinnatus* Willd.	Liana	Fruits	[14,142,167]
*Rubus rigidus* Sm.	Shrub	Fruits	[13]
*Rubus scheffleri* Engl.	Shrub	Fruits	[167]
*Rubus steudneri* Schweinf.	Shrub/liana	Fruits	[142]
*Rubus volkensii* Engl.	Shrub	Fruits	[167]
**Rubiaceae**			
*Afrocanthium keniense* (Bullock) Lantz	Tree	Fruits	[142]
*Afrocanthium kilifiense* (Bridson) Lantz	Tree	Fruits	[142]
*Afrocanthium lactescens* (Hiern) Lantz	Tree	Fruits	[142,163,167]
*Afrocanthium pseudoverticillatum* (S.Moore) Lantz	Shrub/tree	Fruits	[142]
*Bullockia dyscritos* (Bullock) Razafim., Lantz & B.Bremer	Tree	Fruits	[142]
*Bullockia fadenii* (Bridson) Razafim., Lantz & B.Bremer	Shrub/tree	Fruits	[142]
*Bullockia mombazensis* (Baill.) Razafim., Lantz & B.Bremer	Shrub/tree	Fruits	[100,142]
*Bullockia pseudosetiflora* (Bridson) Razafim., Lantz & B.Bremer	Shrub	Fruits	[18,142]
*Bullockia setiflora* (Hiern) Razafim., Lantz & B.Bremer	Shrub/tree	Fruits	[13,142,163]
*Canthium glaucum* Hiern	Shrub/tree	Fruits	[142,167]
*Canthium oligocarpum* Hiern	Shrub/tree	Fruits	[142]
*Coffea arabica* L.	Shrub/tree	Seeds (coffee)	[14,167]
*Conostomium quadrangulare* (Rendle) Cufod.	Herb	Nectar	[157,163]
*Coptosperma graveolens* (S.Moore) Degreef	Shrub/tree	Fruits	[134]
*Coptosperma littorale* (Hiern) Degreef	Shrub/tree	Seed/fruit	[134]
*Cremaspora triflora* (Thonn.) K.Schum.	Shrub/tree	Fruits	[142]
*Eumachia parviflora* (R.D.Good) Razafim. & C.M.Taylor (exotic)	Shrub	Fruits	[142]
*Gardenia ternifolia* Schumach. & Thonn.	Shrub/tree	General	[163]
*Heinsia crinita* (Wennberg) G.Taylor	Shrub/tree	Fruits	[142,158,161]
*Keetia gueinzii* (Sond.) Bridson	Shrub/tree	Fruits	[16,142]
*Keetia zanzibarica* (Klotzsch) Bridson	Shrub	Fruits	[142]
*Lamprothamnus zanguebaricus* Hiern	Shrub/tree	Fruits	[142,161]
*Meyna tetraphylla* (Schweinf. ex Hiern) Robyns	Shrub/tree	Fruits	[14,100,162,163,165,167]
*Mussaenda arcuata* Poir.	Shrub/tree	Fruits	[142]
*Pavetta crassipes* K.Schum.	Shrub/tree	Fruits	[142]
*Pavetta gardeniifolia* Hochst. ex A.Rich.	Shrub/tree	Fruits	[13]
*Pentanisia schweinfurthii* Hiern	Herb	General	[163]
*Polysphaeria multiflora* Hiern	Shrub/tree	Fruits	[142]
*Polysphaeria parvifolia* Hiern	Shrub/tree	Fruits	[161]
*Pyrostria bibracteata* (Baker) Cavaco	Shrub/tree	Fruits	[142]
*Pyrostria phyllanthoidea* (Baill.) Bridson	Shrub/tree	Fruits	[100,142]
*Rothmannia fischeri* (K.Schum.) Bullock ex Oberm.	Tree	Fruits	[142]
*Rytigynia uhligii* (K.Schum. & K.Krause) Verdc.	Shrub/tree	Fruits	[142]
*Tennantia sennii* (Chiov.) Verdc. & Bridson	Shrub	Fruits	[100,142,165]
*Uncaria africana* G.Don	Liana	Fruits	[13]
*Vangueria apiculata* K.Schum.	Shrub/tree	Fruits	[142,167]
*Vangueria infausta* Burch.	Shrub/tree	Fruits	[142,161,167]
*Vangueria loranthifolia* K.Schum.	Shrub/tree	Fruits	[13]
*Vangueria madagascariensis* J.F.Gmel.	Shrub/tree	Fruits	[13,14,100,142,158,163,165,167]
*Vangueria schumanniana* (Robyns) Lantz	Shrub/tree	Fruits	[167]
*Vangueria volkensii* K.Schum.	Shrub/tree	Fruits	[142,167]
**Rutaceae**			
*Harrisonia abyssinica* Oliv.	Shrub/tree	Fruits, roots	[100,142,163]
*Vepris glomerata* (F.Hoffm.) Engl.	Shrub/tree	Fruits	[142]
*Vepris nobilis* (Delile) Mziray	Tree	Fruits	[142]
*Vepris uguenensis* Engl.	Shrub	General	[163]
*Zanthoxylum asiaticum* (L.) Appelhans, Groppo & J.Wen	Shrub/tree	Fruits (food flavor)	[134]
*Zanthoxylum chalybeum* Engl.	Shrub/tree	Fruits; leaves, bark, seeds (flavor tea, soup)	[18,100,134,161,163,167]
*Zanthoxylum holtzianum* (Engl.) P.G.Waterman	Shrub/tree	Fruits	[158]
*Zanthoxylum usambarense* (Engl.) Kokwaro	Tree	Leaves/bark (flavor tea/soup)	[167]
**Salicaceae**			
*Dovyalis abyssinica* (A.Rich.) Warb.	Shrub/tree	Fruits	[13,16,142,167]
*Dovyalis macrocalyx* (Oliv.) Warb.	Shrub/tree	Fruits	[167]
*Flacourtia indica* (Burm.f.) Merr.	Shrub/tree	Seed/fruits	[18,134,142,161,163,167]
*Oncoba spinosa* Forssk.	Shrub/tree	Fruit	[142]
*Scolopia zeyheri* (Nees) Szyszyl.	Shrub/tree	Fruits	[142]
**Salvadoraceae**			
*Dobera glabra* (Forssk.) Juss. ex Poir.	Shrub/tree	Fruits	[18,100,142,157,162,163,167]
*Dobera loranthifolia* (Warb.) Harms	Tree	Fruits	[142]
*Salvadora persica* L.	Shrub/tree	Fruits/roots	[13,18,142,157,162,163,164,167]
**Santalaceae**			
*Osyris lanceolata* Hochst. & Steud.	Shrub	Fruits	[142]
**Sapindaceae**			
*Allophylus africanus* var. *griseotomentosus* (Gilg) Verdc.	Shrub/tree	Fruits	[13]
*Allophylus ferrugineus* Taub.	Shrub/tree	Fruits	[142]
*Allophylus rubifolius* (Hochst. ex A.Rich.) Engl.	Shrub/tree	Fruits	[100]
*Chytranthus obliquinervis* Radlk. ex Engl.	Shrub/tree	Fruits	[142,158]
*Deinbollia borbonica* Scheff.	Shrub/tree	Fruits	[134,142,161]
*Deinbollia kilimandscharica* Taub.	Shrub/tree	Fruits	[142,165]
*Haplocoelum foliolosum* (Hiern) Bullock	Shrub/tree	Fruits	[13,142]
*Haplocoelum inoploeum* Radlk.	Shrub/tree	Fruits; leaves (vegetable)	[142,158]
*Lecaniodiscus fraxinifolius* Baker	Tree	Fruits	[100,161]
*Lepisanthes senegalensis* (Poir.) Leenh.	Tree	Fruits	[16]
*Pappea capensis* Eckl. & Zeyh.	Tree	Fruits/seeds; bark (tea)	[16,100,142,163,167]
**Sapotaceae**			
*Aningeria adolfi-friederici* (Engl.) Robyns & Gilbert	Tree	Seed oil	[142]
*Englerophytum natalense* (Sond.) T.D.Penn.	Tree	Fruits	[142]
*Englerophytum oblanceolatum* (S.Moore) T.D.Penn.	Tree	Fruit	[142]
*Gambeya albida* (G.Don) Aubrév. & Pellegr.	Tree	Fruit	[142]
*Inhambanella henriquesii* (Engl. & Warb.) Dubard	Tree	Fruits	[142]
*Malacantha alnifolia* (Baker) Pierre	Tree	Fruits	[142]
*Manilkara butugi* Chiov.	Tree	Fruits	[142]
*Manilkara discolor* (Sond.) J.H.Hemsl.	Tree	Fruits	[13,142]
*Manilkara mochisia* (Baker) Dubard	Tree	Fruits	[100,142,158,161,163,167]
*Manilkara sansibarensis* (Engl.) Dubard	Shrub/tree	Fruits	[142,167]
*Manilkara sulcata* (Engl.) Dubard	Tree	Fruits	[100,142,161,167]
*Mimusops bagshawei* S.Moore	Tree	Fruits	[142]
*Mimusops kummel* Bruce ex A.DC.	Tree	Fruits	[16,142,163,167]
*Mimusops obtusifolia* Lam.	Shrub/tree	Fruits	[100,142,161,167]
*Mimusops somalensis* Chiov.	Tree	Fruits	[161,167]
*Sideroxylon inerme* L.	Shrub/tree	Fruits	[142]
*Synsepalum brevipes* (Baker) T.D.Penn.	Tree	Fruits	[142,167]
*Synsepalum cerasiferum* (Welw.) T.D.Penn.	Tree	Fruits	[142]
*Synsepalum msolo* (Engl.) T.D.Penn.	Tree	Fruits	[142,167]
**Solanaceae**			
*Capsicum frutescens* L. (exotic)	Shrub	Leaves (vegetable)	[160]
*Lycium shawii* Roem. & Schult.	Shrub/tree	General	[162,163]
*Nicandra physalodes* (L.) Gaertn.	Herb	Leaves (vegetable)	[161]
*Physalis angulata* L.	Herb	Leaves (vegetable)	[169]
*Physalis lagascae* Roem. & Schult.	Herb	Leaves (vegetable)	[159]
*Physalis peruviana* L.	Herb	Fruits	[16]
*Solanum aculeastrum* Dunal	Shrub/tree	Fruits	[142]
*Solanum nigrum* L	Herb	Leaves (vegetable)	[12,13,16,157,158,159,160,161,163,166,169]
*Solanum coagulans* Forssk.	Subshrub	Seeds (coagulate milk)	[157,163,164]
*Solanum incanum* L.	Shrub/subshrub	General	[164]
*Solanum lycopersicum* L. (exotic)	Herb	Leaves (vegetable)	[160]
*Solanum villosum* Mill.	Herb/shrub	Leaves (vegetable)	[162]
**Talinaceae**			
*Talinum caffrum* (Thunb.) Eckl. & Zeyh.	Herb	Leaves (vegetable)	[161]
*Talinum portulacifolium* (Forssk.) Asch. ex Schweinf.	Herb	Leaves (vegetable)	[13,16,134,159,160,161,164,169]
**Urticaceae**			
*Forsskaolea viridis* Ehrenb. ex Webb	Subshrub	Leaves (vegetable)	[157]
*Laportea alatipes* Hook.f.	Herb	Leaves	[14]
*Myrianthus holstii* Engl.	Tree	Fruits	[14,142,167]
*Pilea johnstonii* Oliv.	Subshrub	Leaves (vegetable)	[12]
*Urtica massaica* Mildbr.	Herb	Leaves (vegetable)	[12,166,167]
**Verbenaceae**			
*Lantana camara* L. (exotic)	Shrub	Fruits	[16,161,165,167]
*Lantana trifolia* L.	Shrub	Fruits; leaves (vegetable)	[16,142,165,167]
*Lantana ukambensis* (Vatke) Verdc.	Shrub	Fruits	[142,163,167]
*Lantana viburnoides* (Forssk.) Vahl	Subshrub/shrub	Fruits	[167]
*Lippia carviodora* Meikle	Shrub	Leaves (tea)	[142,164,167]
*Lippia javanica* (Burm.f.) Spreng.	Shrub	Fruits edible; leaves used in tea	[167]
*Lippia kituiensis* Vatke	Shrub	Fruits edible; leaves used in tea	[14,142,167]
**Vitaceae**			
*Cayratia ibuensis* (Hook.f.) Suess.	Herb	Roots	[156]
*Cissus aphyllantha* Gilg	Shrub	Fruits	[142]
*Cissus quadrangularis* L.	Liana	General	[164]
*Cissus rotundifolia* Vahl	Shrub	Fruits; roots used in soup	[16,142,156,162,163]
*Cyphostemma adenocaule* (Steud. ex A.Rich.) Desc. ex Wild & R.B.Drumm.	Herb	Fruits; leaves (vegetable)	[158,163,165]
*Cyphostemma bambuseti* (Gilg & M.Brandt) Desc. ex Wild & R.B.Drumm.	Herb	General	[163]
*Cyphostemma cyphopetalum* (Fresen.) Desc. ex Wild & R.B.Drumm.	Herb	Leaves (vegetable)	[157,159,160,163,169]
*Cyphostemma serpens* (Hochst. ex A.Rich.) Desc.	Herb	Leaves (vegetable)	[13,16,169]
**Zingiberaceae**			
*Aframomum angustifolium* (Sonn.) K.Schum.	Herb	Seed/fruits	[134]
*Aframomum mala* (K.Schum.) K.Schum.	Herb	Fruits	[16]
*Zingiber officinale* Roscoe (exotic)	Herb	Root	[16]
**Zygophyllaceae**			
*Balanites aegyptiaca* (L.) Delile	Tree	Fruit, seed oil, gum; leaf/shoots (vegetable)	[14,16,18,100,142,162,163,165,166,167]
*Balanites glabra* Mildbr. & Schltr.	Shrub/tree	Fruits	[142,164]
*Balanites pedicellaris* Mildbr. & Schltr.	Tree	Fruits, cotyledons	[18,100,142,162,163,164,167]
*Balanites rotundifolia* (Tiegh.) Blatt.	Shrub/tree	Fruits, cotyledons; fruit (flavor milk)	[13,18,142,157,162,163,167]
*Tribulus cistoides* L.	Herb	Leaves	[18,162]
*Tribulus terrestris* L.	Herb	Leaves (vegetable)	[159,160]
